# Supplementary material for: Differential effects of propofol and ketamine on critical brain dynamics
Source: PLoS Comput Biol. 2020 Dec 21;16(12):e1008418. doi: 10.1371/journal.pcbi.1008418 (PMC7785236; doi:10.1371/journal.pcbi.1008418)
Supplement: S1 Scripts — (TAR.GZ) [file pcbi.1008418.s002.tar.gz › varley_2020_scripts/scripts/cython/avalanches.html]

Cython: avalanches.pyx


Generated by Cython 0.28.5

Yellow lines hint at Python interaction.  
Click on a line that starts with a "`+`" to see the C code that Cython generated for it.

Raw output: avalanches.c

```
+001: #!/usr/bin/env python3
```

```
  __pyx_t_2 = __Pyx_PyDict_NewPresized(0); if (unlikely(!__pyx_t_2)) __PYX_ERR(0, 1, __pyx_L1_error)
  __Pyx_GOTREF(__pyx_t_2);
  if (PyDict_SetItem(__pyx_d, __pyx_n_s_test, __pyx_t_2) < 0) __PYX_ERR(0, 1, __pyx_L1_error)
  __Pyx_DECREF(__pyx_t_2); __pyx_t_2 = 0;
```

```
 002: # -*- coding: utf-8 -*-
```

```
 003: """
```

```
 004: Created on Thu Jan 17 14:10:01 2019
```

```
 005:
```

```
 006: @author: thosvarley
```

```
 007:
```

```
 008: """
```

```
+009: import numpy as np
```

```
  __pyx_t_1 = __Pyx_Import(__pyx_n_s_numpy, 0, -1); if (unlikely(!__pyx_t_1)) __PYX_ERR(0, 9, __pyx_L1_error)
  __Pyx_GOTREF(__pyx_t_1);
  if (PyDict_SetItem(__pyx_d, __pyx_n_s_np, __pyx_t_1) < 0) __PYX_ERR(0, 9, __pyx_L1_error)
  __Pyx_DECREF(__pyx_t_1); __pyx_t_1 = 0;
```

```
 010: cimport numpy as np
```

```
+011: import matplotlib.pyplot as plt
```

```
  __pyx_t_1 = PyList_New(1); if (unlikely(!__pyx_t_1)) __PYX_ERR(0, 11, __pyx_L1_error)
  __Pyx_GOTREF(__pyx_t_1);
  __Pyx_INCREF(__pyx_n_s__38);
  __Pyx_GIVEREF(__pyx_n_s__38);
  PyList_SET_ITEM(__pyx_t_1, 0, __pyx_n_s__38);
  __pyx_t_2 = __Pyx_Import(__pyx_n_s_matplotlib_pyplot, __pyx_t_1, -1); if (unlikely(!__pyx_t_2)) __PYX_ERR(0, 11, __pyx_L1_error)
  __Pyx_GOTREF(__pyx_t_2);
  __Pyx_DECREF(__pyx_t_1); __pyx_t_1 = 0;
  if (PyDict_SetItem(__pyx_d, __pyx_n_s_plt, __pyx_t_2) < 0) __PYX_ERR(0, 11, __pyx_L1_error)
  __Pyx_DECREF(__pyx_t_2); __pyx_t_2 = 0;
```

```
 012: #from matplotlib import rc
```

```
 013: #rc("text", usetex=True)
```

```
 014:
```

```
+015: from collections import Counter
```

```
  __pyx_t_2 = PyList_New(1); if (unlikely(!__pyx_t_2)) __PYX_ERR(0, 15, __pyx_L1_error)
  __Pyx_GOTREF(__pyx_t_2);
  __Pyx_INCREF(__pyx_n_s_Counter);
  __Pyx_GIVEREF(__pyx_n_s_Counter);
  PyList_SET_ITEM(__pyx_t_2, 0, __pyx_n_s_Counter);
  __pyx_t_1 = __Pyx_Import(__pyx_n_s_collections, __pyx_t_2, -1); if (unlikely(!__pyx_t_1)) __PYX_ERR(0, 15, __pyx_L1_error)
  __Pyx_GOTREF(__pyx_t_1);
  __Pyx_DECREF(__pyx_t_2); __pyx_t_2 = 0;
  __pyx_t_2 = __Pyx_ImportFrom(__pyx_t_1, __pyx_n_s_Counter); if (unlikely(!__pyx_t_2)) __PYX_ERR(0, 15, __pyx_L1_error)
  __Pyx_GOTREF(__pyx_t_2);
  if (PyDict_SetItem(__pyx_d, __pyx_n_s_Counter, __pyx_t_2) < 0) __PYX_ERR(0, 15, __pyx_L1_error)
  __Pyx_DECREF(__pyx_t_2); __pyx_t_2 = 0;
  __Pyx_DECREF(__pyx_t_1); __pyx_t_1 = 0;
```

```
+016: from copy import deepcopy
```

```
  __pyx_t_1 = PyList_New(1); if (unlikely(!__pyx_t_1)) __PYX_ERR(0, 16, __pyx_L1_error)
  __Pyx_GOTREF(__pyx_t_1);
  __Pyx_INCREF(__pyx_n_s_deepcopy);
  __Pyx_GIVEREF(__pyx_n_s_deepcopy);
  PyList_SET_ITEM(__pyx_t_1, 0, __pyx_n_s_deepcopy);
  __pyx_t_2 = __Pyx_Import(__pyx_n_s_copy, __pyx_t_1, -1); if (unlikely(!__pyx_t_2)) __PYX_ERR(0, 16, __pyx_L1_error)
  __Pyx_GOTREF(__pyx_t_2);
  __Pyx_DECREF(__pyx_t_1); __pyx_t_1 = 0;
  __pyx_t_1 = __Pyx_ImportFrom(__pyx_t_2, __pyx_n_s_deepcopy); if (unlikely(!__pyx_t_1)) __PYX_ERR(0, 16, __pyx_L1_error)
  __Pyx_GOTREF(__pyx_t_1);
  if (PyDict_SetItem(__pyx_d, __pyx_n_s_deepcopy, __pyx_t_1) < 0) __PYX_ERR(0, 16, __pyx_L1_error)
  __Pyx_DECREF(__pyx_t_1); __pyx_t_1 = 0;
  __Pyx_DECREF(__pyx_t_2); __pyx_t_2 = 0;
```

```
+017: from mne.preprocessing.peak_finder import peak_finder
```

```
  __pyx_t_2 = PyList_New(1); if (unlikely(!__pyx_t_2)) __PYX_ERR(0, 17, __pyx_L1_error)
  __Pyx_GOTREF(__pyx_t_2);
  __Pyx_INCREF(__pyx_n_s_peak_finder);
  __Pyx_GIVEREF(__pyx_n_s_peak_finder);
  PyList_SET_ITEM(__pyx_t_2, 0, __pyx_n_s_peak_finder);
  __pyx_t_1 = __Pyx_Import(__pyx_n_s_mne_preprocessing_peak_finder, __pyx_t_2, -1); if (unlikely(!__pyx_t_1)) __PYX_ERR(0, 17, __pyx_L1_error)
  __Pyx_GOTREF(__pyx_t_1);
  __Pyx_DECREF(__pyx_t_2); __pyx_t_2 = 0;
  __pyx_t_2 = __Pyx_ImportFrom(__pyx_t_1, __pyx_n_s_peak_finder); if (unlikely(!__pyx_t_2)) __PYX_ERR(0, 17, __pyx_L1_error)
  __Pyx_GOTREF(__pyx_t_2);
  if (PyDict_SetItem(__pyx_d, __pyx_n_s_peak_finder, __pyx_t_2) < 0) __PYX_ERR(0, 17, __pyx_L1_error)
  __Pyx_DECREF(__pyx_t_2); __pyx_t_2 = 0;
  __Pyx_DECREF(__pyx_t_1); __pyx_t_1 = 0;
```

```
+018: from scipy.stats import linregress, pearsonr
```

```
  __pyx_t_1 = PyList_New(2); if (unlikely(!__pyx_t_1)) __PYX_ERR(0, 18, __pyx_L1_error)
  __Pyx_GOTREF(__pyx_t_1);
  __Pyx_INCREF(__pyx_n_s_linregress);
  __Pyx_GIVEREF(__pyx_n_s_linregress);
  PyList_SET_ITEM(__pyx_t_1, 0, __pyx_n_s_linregress);
  __Pyx_INCREF(__pyx_n_s_pearsonr);
  __Pyx_GIVEREF(__pyx_n_s_pearsonr);
  PyList_SET_ITEM(__pyx_t_1, 1, __pyx_n_s_pearsonr);
  __pyx_t_2 = __Pyx_Import(__pyx_n_s_scipy_stats, __pyx_t_1, -1); if (unlikely(!__pyx_t_2)) __PYX_ERR(0, 18, __pyx_L1_error)
  __Pyx_GOTREF(__pyx_t_2);
  __Pyx_DECREF(__pyx_t_1); __pyx_t_1 = 0;
  __pyx_t_1 = __Pyx_ImportFrom(__pyx_t_2, __pyx_n_s_linregress); if (unlikely(!__pyx_t_1)) __PYX_ERR(0, 18, __pyx_L1_error)
  __Pyx_GOTREF(__pyx_t_1);
  if (PyDict_SetItem(__pyx_d, __pyx_n_s_linregress, __pyx_t_1) < 0) __PYX_ERR(0, 18, __pyx_L1_error)
  __Pyx_DECREF(__pyx_t_1); __pyx_t_1 = 0;
  __pyx_t_1 = __Pyx_ImportFrom(__pyx_t_2, __pyx_n_s_pearsonr); if (unlikely(!__pyx_t_1)) __PYX_ERR(0, 18, __pyx_L1_error)
  __Pyx_GOTREF(__pyx_t_1);
  if (PyDict_SetItem(__pyx_d, __pyx_n_s_pearsonr, __pyx_t_1) < 0) __PYX_ERR(0, 18, __pyx_L1_error)
  __Pyx_DECREF(__pyx_t_1); __pyx_t_1 = 0;
  __Pyx_DECREF(__pyx_t_2); __pyx_t_2 = 0;
```

```
 019:
```

```
 020: cimport cython
```

```
 021: from libc.math cimport log10, floor
```

```
 022:
```

```
 023: @cython.boundscheck(False)
```

```
 024: @cython.wraparound(False)
```

```
+025: cdef int total_raster_sum(int[:,:] X):
```

```
static int __pyx_f_10avalanches_total_raster_sum(__Pyx_memviewslice __pyx_v_X) {
  int __pyx_v_i;
  int __pyx_v_j;
  int __pyx_v_s;
  int __pyx_v_N0;
  int __pyx_v_N1;
  int __pyx_r;
  __Pyx_RefNannyDeclarations
  __Pyx_RefNannySetupContext("total_raster_sum", 0);
/* … */
  /* function exit code */
  __pyx_L0:;
  __Pyx_RefNannyFinishContext();
  return __pyx_r;
}
```

```
 026:     cdef int i, j
```

```
+027:     cdef int s = 0
```

```
  __pyx_v_s = 0;
```

```
+028:     cdef int N0 = X.shape[0]
```

```
  __pyx_v_N0 = (__pyx_v_X.shape[0]);
```

```
+029:     cdef int N1 = X.shape[1]
```

```
  __pyx_v_N1 = (__pyx_v_X.shape[1]);
```

```
 030:
```

```
+031:     for i in range(N0):
```

```
  __pyx_t_1 = __pyx_v_N0;
  __pyx_t_2 = __pyx_t_1;
  for (__pyx_t_3 = 0; __pyx_t_3 < __pyx_t_2; __pyx_t_3+=1) {
    __pyx_v_i = __pyx_t_3;
```

```
+032:         for j in range(N1):
```

```
    __pyx_t_4 = __pyx_v_N1;
    __pyx_t_5 = __pyx_t_4;
    for (__pyx_t_6 = 0; __pyx_t_6 < __pyx_t_5; __pyx_t_6+=1) {
      __pyx_v_j = __pyx_t_6;
```

```
+033:             s += X[i][j]
```

```
      __pyx_t_7 = __pyx_v_i;
      __pyx_t_8 = __pyx_v_j;
      __pyx_v_s = (__pyx_v_s + (*((int *) ( /* dim=1 */ (( /* dim=0 */ (__pyx_v_X.data + __pyx_t_7 * __pyx_v_X.strides[0]) ) + __pyx_t_8 * __pyx_v_X.strides[1]) ))));
    }
  }
```

```
 034:
```

```
+035:     return s
```

```
  __pyx_r = __pyx_v_s;
  goto __pyx_L0;
```

```
 036:
```

```
 037: @cython.boundscheck(False)
```

```
 038: @cython.wraparound(False)
```

```
+039: cdef int number_active_channels(int[:,:] X):
```

```
static int __pyx_f_10avalanches_number_active_channels(__Pyx_memviewslice __pyx_v_X) {
  int __pyx_v_i;
  int __pyx_v_j;
  int __pyx_v_s;
  int __pyx_v_N0;
  int __pyx_v_N1;
  int __pyx_v_t;
  int __pyx_r;
  __Pyx_RefNannyDeclarations
  __Pyx_RefNannySetupContext("number_active_channels", 0);
/* … */
  /* function exit code */
  __pyx_L0:;
  __Pyx_RefNannyFinishContext();
  return __pyx_r;
}
```

```
 040:     cdef int i, j
```

```
+041:     cdef int s = 0
```

```
  __pyx_v_s = 0;
```

```
+042:     cdef int N0 = X.shape[0]
```

```
  __pyx_v_N0 = (__pyx_v_X.shape[0]);
```

```
+043:     cdef int N1 = X.shape[1]
```

```
  __pyx_v_N1 = (__pyx_v_X.shape[1]);
```

```
+044:     cdef int t = 0
```

```
  __pyx_v_t = 0;
```

```
 045:
```

```
+046:     for i in range(N0):
```

```
  __pyx_t_1 = __pyx_v_N0;
  __pyx_t_2 = __pyx_t_1;
  for (__pyx_t_3 = 0; __pyx_t_3 < __pyx_t_2; __pyx_t_3+=1) {
    __pyx_v_i = __pyx_t_3;
```

```
+047:         t = 0
```

```
    __pyx_v_t = 0;
```

```
+048:         for j in range(N1):
```

```
    __pyx_t_4 = __pyx_v_N1;
    __pyx_t_5 = __pyx_t_4;
    for (__pyx_t_6 = 0; __pyx_t_6 < __pyx_t_5; __pyx_t_6+=1) {
      __pyx_v_j = __pyx_t_6;
```

```
+049:             t += X[i][j]
```

```
      __pyx_t_7 = __pyx_v_i;
      __pyx_t_8 = __pyx_v_j;
      __pyx_v_t = (__pyx_v_t + (*((int *) ( /* dim=1 */ (( /* dim=0 */ (__pyx_v_X.data + __pyx_t_7 * __pyx_v_X.strides[0]) ) + __pyx_t_8 * __pyx_v_X.strides[1]) ))));
    }
```

```
+050:         if t != 0:
```

```
    __pyx_t_9 = ((__pyx_v_t != 0) != 0);
    if (__pyx_t_9) {
/* … */
    }
  }
```

```
+051:             s += 1
```

```
      __pyx_v_s = (__pyx_v_s + 1);
```

```
 052:
```

```
+053:     return s
```

```
  __pyx_r = __pyx_v_s;
  goto __pyx_L0;
```

```
 054:
```

```
 055: @cython.boundscheck(False)
```

```
 056: @cython.wraparound(False)
```

```
 057: @cython.cdivision(True)
```

```
+058: def normalize(double[:,:] X):
```

```
/* Python wrapper */
static PyObject *__pyx_pw_10avalanches_1normalize(PyObject *__pyx_self, PyObject *__pyx_arg_X); /*proto*/
static char __pyx_doc_10avalanches_normalize[] = "\n    Normalizes a 2D array by subtracting out the mean of each row and dividing by the standard deviation. \n    Normalizes the array in-place. \n    \n    Arguments:\n        \n        X:\n            A 2-dimensional Numpy array (dtype must be \"double\")\n    \n    Returns:\n        \n        X: \n            The original array, normalized in-place. \n    ";
static PyMethodDef __pyx_mdef_10avalanches_1normalize = {"normalize", (PyCFunction)__pyx_pw_10avalanches_1normalize, METH_O, __pyx_doc_10avalanches_normalize};
static PyObject *__pyx_pw_10avalanches_1normalize(PyObject *__pyx_self, PyObject *__pyx_arg_X) {
  __Pyx_memviewslice __pyx_v_X = { 0, 0, { 0 }, { 0 }, { 0 } };
  PyObject *__pyx_r = 0;
  __Pyx_RefNannyDeclarations
  __Pyx_RefNannySetupContext("normalize (wrapper)", 0);
  assert(__pyx_arg_X); {
    __pyx_v_X = __Pyx_PyObject_to_MemoryviewSlice_dsds_double(__pyx_arg_X, PyBUF_WRITABLE); if (unlikely(!__pyx_v_X.memview)) __PYX_ERR(0, 58, __pyx_L3_error)
  }
  goto __pyx_L4_argument_unpacking_done;
  __pyx_L3_error:;
  __Pyx_AddTraceback("avalanches.normalize", __pyx_clineno, __pyx_lineno, __pyx_filename);
  __Pyx_RefNannyFinishContext();
  return NULL;
  __pyx_L4_argument_unpacking_done:;
  __pyx_r = __pyx_pf_10avalanches_normalize(__pyx_self, __pyx_v_X);

  /* function exit code */
  __Pyx_RefNannyFinishContext();
  return __pyx_r;
}

static PyObject *__pyx_pf_10avalanches_normalize(CYTHON_UNUSED PyObject *__pyx_self, __Pyx_memviewslice __pyx_v_X) {
  __Pyx_memviewslice __pyx_v_means = { 0, 0, { 0 }, { 0 }, { 0 } };
  __Pyx_memviewslice __pyx_v_sds = { 0, 0, { 0 }, { 0 }, { 0 } };
  int __pyx_v_N0;
  int __pyx_v_N1;
  int __pyx_v_i;
  int __pyx_v_j;
  PyObject *__pyx_r = NULL;
  __Pyx_RefNannyDeclarations
  __Pyx_RefNannySetupContext("normalize", 0);
/* … */
  /* function exit code */
  __pyx_L1_error:;
  __Pyx_XDECREF(__pyx_t_1);
  __Pyx_XDECREF(__pyx_t_2);
  __Pyx_XDECREF(__pyx_t_3);
  __Pyx_XDECREF(__pyx_t_4);
  __PYX_XDEC_MEMVIEW(&__pyx_t_5, 1);
  __Pyx_AddTraceback("avalanches.normalize", __pyx_clineno, __pyx_lineno, __pyx_filename);
  __pyx_r = NULL;
  __pyx_L0:;
  __PYX_XDEC_MEMVIEW(&__pyx_v_X, 1);
  __PYX_XDEC_MEMVIEW(&__pyx_v_means, 1);
  __PYX_XDEC_MEMVIEW(&__pyx_v_sds, 1);
  __Pyx_XGIVEREF(__pyx_r);
  __Pyx_RefNannyFinishContext();
  return __pyx_r;
}
/* … */
  __pyx_tuple__39 = PyTuple_Pack(8, __pyx_n_s_X, __pyx_n_s_X, __pyx_n_s_means, __pyx_n_s_sds, __pyx_n_s_N0, __pyx_n_s_N1, __pyx_n_s_i, __pyx_n_s_j); if (unlikely(!__pyx_tuple__39)) __PYX_ERR(0, 58, __pyx_L1_error)
  __Pyx_GOTREF(__pyx_tuple__39);
  __Pyx_GIVEREF(__pyx_tuple__39);
/* … */
  __pyx_t_2 = PyCFunction_NewEx(&__pyx_mdef_10avalanches_1normalize, NULL, __pyx_n_s_avalanches); if (unlikely(!__pyx_t_2)) __PYX_ERR(0, 58, __pyx_L1_error)
  __Pyx_GOTREF(__pyx_t_2);
  if (PyDict_SetItem(__pyx_d, __pyx_n_s_normalize, __pyx_t_2) < 0) __PYX_ERR(0, 58, __pyx_L1_error)
  __Pyx_DECREF(__pyx_t_2); __pyx_t_2 = 0;
  __pyx_codeobj__40 = (PyObject*)__Pyx_PyCode_New(1, 0, 8, 0, CO_OPTIMIZED|CO_NEWLOCALS, __pyx_empty_bytes, __pyx_empty_tuple, __pyx_empty_tuple, __pyx_tuple__39, __pyx_empty_tuple, __pyx_empty_tuple, __pyx_kp_s_avalanches_pyx, __pyx_n_s_normalize, 58, __pyx_empty_bytes); if (unlikely(!__pyx_codeobj__40)) __PYX_ERR(0, 58, __pyx_L1_error)
```

```
 059:     """
```

```
 060:     Normalizes a 2D array by subtracting out the mean of each row and dividing by the standard deviation.
```

```
 061:     Normalizes the array in-place.
```

```
 062:
```

```
 063:     Arguments:
```

```
 064:
```

```
 065:         X:
```

```
 066:             A 2-dimensional Numpy array (dtype must be "double")
```

```
 067:
```

```
 068:     Returns:
```

```
 069:
```

```
 070:         X:
```

```
 071:             The original array, normalized in-place.
```

```
 072:     """
```

```
+073:     cdef double[:] means = np.mean(X, axis = 1)
```

```
  __pyx_t_1 = __Pyx_GetModuleGlobalName(__pyx_n_s_np); if (unlikely(!__pyx_t_1)) __PYX_ERR(0, 73, __pyx_L1_error)
  __Pyx_GOTREF(__pyx_t_1);
  __pyx_t_2 = __Pyx_PyObject_GetAttrStr(__pyx_t_1, __pyx_n_s_mean); if (unlikely(!__pyx_t_2)) __PYX_ERR(0, 73, __pyx_L1_error)
  __Pyx_GOTREF(__pyx_t_2);
  __Pyx_DECREF(__pyx_t_1); __pyx_t_1 = 0;
  __pyx_t_1 = __pyx_memoryview_fromslice(__pyx_v_X, 2, (PyObject *(*)(char *)) __pyx_memview_get_double, (int (*)(char *, PyObject *)) __pyx_memview_set_double, 0);; if (unlikely(!__pyx_t_1)) __PYX_ERR(0, 73, __pyx_L1_error)
  __Pyx_GOTREF(__pyx_t_1);
  __pyx_t_3 = PyTuple_New(1); if (unlikely(!__pyx_t_3)) __PYX_ERR(0, 73, __pyx_L1_error)
  __Pyx_GOTREF(__pyx_t_3);
  __Pyx_GIVEREF(__pyx_t_1);
  PyTuple_SET_ITEM(__pyx_t_3, 0, __pyx_t_1);
  __pyx_t_1 = 0;
  __pyx_t_1 = __Pyx_PyDict_NewPresized(1); if (unlikely(!__pyx_t_1)) __PYX_ERR(0, 73, __pyx_L1_error)
  __Pyx_GOTREF(__pyx_t_1);
  if (PyDict_SetItem(__pyx_t_1, __pyx_n_s_axis, __pyx_int_1) < 0) __PYX_ERR(0, 73, __pyx_L1_error)
  __pyx_t_4 = __Pyx_PyObject_Call(__pyx_t_2, __pyx_t_3, __pyx_t_1); if (unlikely(!__pyx_t_4)) __PYX_ERR(0, 73, __pyx_L1_error)
  __Pyx_GOTREF(__pyx_t_4);
  __Pyx_DECREF(__pyx_t_2); __pyx_t_2 = 0;
  __Pyx_DECREF(__pyx_t_3); __pyx_t_3 = 0;
  __Pyx_DECREF(__pyx_t_1); __pyx_t_1 = 0;
  __pyx_t_5 = __Pyx_PyObject_to_MemoryviewSlice_ds_double(__pyx_t_4, PyBUF_WRITABLE); if (unlikely(!__pyx_t_5.memview)) __PYX_ERR(0, 73, __pyx_L1_error)
  __Pyx_DECREF(__pyx_t_4); __pyx_t_4 = 0;
  __pyx_v_means = __pyx_t_5;
  __pyx_t_5.memview = NULL;
  __pyx_t_5.data = NULL;
```

```
+074:     cdef double[:] sds = np.std(X, axis = 1)
```

```
  __pyx_t_4 = __Pyx_GetModuleGlobalName(__pyx_n_s_np); if (unlikely(!__pyx_t_4)) __PYX_ERR(0, 74, __pyx_L1_error)
  __Pyx_GOTREF(__pyx_t_4);
  __pyx_t_1 = __Pyx_PyObject_GetAttrStr(__pyx_t_4, __pyx_n_s_std); if (unlikely(!__pyx_t_1)) __PYX_ERR(0, 74, __pyx_L1_error)
  __Pyx_GOTREF(__pyx_t_1);
  __Pyx_DECREF(__pyx_t_4); __pyx_t_4 = 0;
  __pyx_t_4 = __pyx_memoryview_fromslice(__pyx_v_X, 2, (PyObject *(*)(char *)) __pyx_memview_get_double, (int (*)(char *, PyObject *)) __pyx_memview_set_double, 0);; if (unlikely(!__pyx_t_4)) __PYX_ERR(0, 74, __pyx_L1_error)
  __Pyx_GOTREF(__pyx_t_4);
  __pyx_t_3 = PyTuple_New(1); if (unlikely(!__pyx_t_3)) __PYX_ERR(0, 74, __pyx_L1_error)
  __Pyx_GOTREF(__pyx_t_3);
  __Pyx_GIVEREF(__pyx_t_4);
  PyTuple_SET_ITEM(__pyx_t_3, 0, __pyx_t_4);
  __pyx_t_4 = 0;
  __pyx_t_4 = __Pyx_PyDict_NewPresized(1); if (unlikely(!__pyx_t_4)) __PYX_ERR(0, 74, __pyx_L1_error)
  __Pyx_GOTREF(__pyx_t_4);
  if (PyDict_SetItem(__pyx_t_4, __pyx_n_s_axis, __pyx_int_1) < 0) __PYX_ERR(0, 74, __pyx_L1_error)
  __pyx_t_2 = __Pyx_PyObject_Call(__pyx_t_1, __pyx_t_3, __pyx_t_4); if (unlikely(!__pyx_t_2)) __PYX_ERR(0, 74, __pyx_L1_error)
  __Pyx_GOTREF(__pyx_t_2);
  __Pyx_DECREF(__pyx_t_1); __pyx_t_1 = 0;
  __Pyx_DECREF(__pyx_t_3); __pyx_t_3 = 0;
  __Pyx_DECREF(__pyx_t_4); __pyx_t_4 = 0;
  __pyx_t_5 = __Pyx_PyObject_to_MemoryviewSlice_ds_double(__pyx_t_2, PyBUF_WRITABLE); if (unlikely(!__pyx_t_5.memview)) __PYX_ERR(0, 74, __pyx_L1_error)
  __Pyx_DECREF(__pyx_t_2); __pyx_t_2 = 0;
  __pyx_v_sds = __pyx_t_5;
  __pyx_t_5.memview = NULL;
  __pyx_t_5.data = NULL;
```

```
+075:     cdef int N0 = X.shape[0]
```

```
  __pyx_v_N0 = (__pyx_v_X.shape[0]);
```

```
+076:     cdef int N1 = X.shape[1]
```

```
  __pyx_v_N1 = (__pyx_v_X.shape[1]);
```

```
 077:     cdef int i, j
```

```
 078:
```

```
+079:     for i in range(N0):
```

```
  __pyx_t_6 = __pyx_v_N0;
  __pyx_t_7 = __pyx_t_6;
  for (__pyx_t_8 = 0; __pyx_t_8 < __pyx_t_7; __pyx_t_8+=1) {
    __pyx_v_i = __pyx_t_8;
```

```
+080:         for j in range(N1):
```

```
    __pyx_t_9 = __pyx_v_N1;
    __pyx_t_10 = __pyx_t_9;
    for (__pyx_t_11 = 0; __pyx_t_11 < __pyx_t_10; __pyx_t_11+=1) {
      __pyx_v_j = __pyx_t_11;
```

```
+081:             X[i][j] = (X[i][j] - means[i])/sds[i]
```

```
      __pyx_t_12 = __pyx_v_i;
      __pyx_t_13 = __pyx_v_j;
      __pyx_t_14 = __pyx_v_i;
      __pyx_t_15 = __pyx_v_i;
      __pyx_t_16 = __pyx_v_i;
      __pyx_t_17 = __pyx_v_j;
      *((double *) ( /* dim=1 */ (( /* dim=0 */ (__pyx_v_X.data + __pyx_t_16 * __pyx_v_X.strides[0]) ) + __pyx_t_17 * __pyx_v_X.strides[1]) )) = (((*((double *) ( /* dim=1 */ (( /* dim=0 */ (__pyx_v_X.data + __pyx_t_12 * __pyx_v_X.strides[0]) ) + __pyx_t_13 * __pyx_v_X.strides[1]) ))) - (*((double *) ( /* dim=0 */ (__pyx_v_means.data + __pyx_t_14 * __pyx_v_means.strides[0]) )))) / (*((double *) ( /* dim=0 */ (__pyx_v_sds.data + __pyx_t_15 * __pyx_v_sds.strides[0]) ))));
    }
  }
```

```
 082:
```

```
+083:     return X
```

```
  __Pyx_XDECREF(__pyx_r);
  __pyx_t_2 = __pyx_memoryview_fromslice(__pyx_v_X, 2, (PyObject *(*)(char *)) __pyx_memview_get_double, (int (*)(char *, PyObject *)) __pyx_memview_set_double, 0);; if (unlikely(!__pyx_t_2)) __PYX_ERR(0, 83, __pyx_L1_error)
  __Pyx_GOTREF(__pyx_t_2);
  __pyx_r = __pyx_t_2;
  __pyx_t_2 = 0;
  goto __pyx_L0;
```

```
 084:
```

```
 085: @cython.boundscheck(False)
```

```
 086: @cython.wraparound(False)
```

```
+087: def simple_raster(double[:,:] X, double threshold):
```

```
/* Python wrapper */
static PyObject *__pyx_pw_10avalanches_3simple_raster(PyObject *__pyx_self, PyObject *__pyx_args, PyObject *__pyx_kwds); /*proto*/
static char __pyx_doc_10avalanches_2simple_raster[] = "\n    The simplest algorithm for identifying \"events.\"\n    Places a 1 every time the time-series crosses a threshold (n * standard devation). \n    \n    Arguments:\n        \n        X:\n            A 2-dimensional Numpy array (dtype must be \"double\"). \n        Threshold:\n            The number of standard deviations the time-series must exceed to be counted as an event. \n    \n    Returns:\n        \n        Raster:\n            A binarized copy of the original array. \n            Places a 1 everywhere the time-series crosses the threshold, 0 otherwise. \n    ";
static PyMethodDef __pyx_mdef_10avalanches_3simple_raster = {"simple_raster", (PyCFunction)__pyx_pw_10avalanches_3simple_raster, METH_VARARGS|METH_KEYWORDS, __pyx_doc_10avalanches_2simple_raster};
static PyObject *__pyx_pw_10avalanches_3simple_raster(PyObject *__pyx_self, PyObject *__pyx_args, PyObject *__pyx_kwds) {
  __Pyx_memviewslice __pyx_v_X = { 0, 0, { 0 }, { 0 }, { 0 } };
  double __pyx_v_threshold;
  PyObject *__pyx_r = 0;
  __Pyx_RefNannyDeclarations
  __Pyx_RefNannySetupContext("simple_raster (wrapper)", 0);
  {
    static PyObject **__pyx_pyargnames[] = {&__pyx_n_s_X,&__pyx_n_s_threshold,0};
    PyObject* values[2] = {0,0};
    if (unlikely(__pyx_kwds)) {
      Py_ssize_t kw_args;
      const Py_ssize_t pos_args = PyTuple_GET_SIZE(__pyx_args);
      switch (pos_args) {
        case  2: values[1] = PyTuple_GET_ITEM(__pyx_args, 1);
        CYTHON_FALLTHROUGH;
        case  1: values[0] = PyTuple_GET_ITEM(__pyx_args, 0);
        CYTHON_FALLTHROUGH;
        case  0: break;
        default: goto __pyx_L5_argtuple_error;
      }
      kw_args = PyDict_Size(__pyx_kwds);
      switch (pos_args) {
        case  0:
        if (likely((values[0] = __Pyx_PyDict_GetItemStr(__pyx_kwds, __pyx_n_s_X)) != 0)) kw_args--;
        else goto __pyx_L5_argtuple_error;
        CYTHON_FALLTHROUGH;
        case  1:
        if (likely((values[1] = __Pyx_PyDict_GetItemStr(__pyx_kwds, __pyx_n_s_threshold)) != 0)) kw_args--;
        else {
          __Pyx_RaiseArgtupleInvalid("simple_raster", 1, 2, 2, 1); __PYX_ERR(0, 87, __pyx_L3_error)
        }
      }
      if (unlikely(kw_args > 0)) {
        if (unlikely(__Pyx_ParseOptionalKeywords(__pyx_kwds, __pyx_pyargnames, 0, values, pos_args, "simple_raster") < 0)) __PYX_ERR(0, 87, __pyx_L3_error)
      }
    } else if (PyTuple_GET_SIZE(__pyx_args) != 2) {
      goto __pyx_L5_argtuple_error;
    } else {
      values[0] = PyTuple_GET_ITEM(__pyx_args, 0);
      values[1] = PyTuple_GET_ITEM(__pyx_args, 1);
    }
    __pyx_v_X = __Pyx_PyObject_to_MemoryviewSlice_dsds_double(values[0], PyBUF_WRITABLE); if (unlikely(!__pyx_v_X.memview)) __PYX_ERR(0, 87, __pyx_L3_error)
    __pyx_v_threshold = __pyx_PyFloat_AsDouble(values[1]); if (unlikely((__pyx_v_threshold == (double)-1) && PyErr_Occurred())) __PYX_ERR(0, 87, __pyx_L3_error)
  }
  goto __pyx_L4_argument_unpacking_done;
  __pyx_L5_argtuple_error:;
  __Pyx_RaiseArgtupleInvalid("simple_raster", 1, 2, 2, PyTuple_GET_SIZE(__pyx_args)); __PYX_ERR(0, 87, __pyx_L3_error)
  __pyx_L3_error:;
  __Pyx_AddTraceback("avalanches.simple_raster", __pyx_clineno, __pyx_lineno, __pyx_filename);
  __Pyx_RefNannyFinishContext();
  return NULL;
  __pyx_L4_argument_unpacking_done:;
  __pyx_r = __pyx_pf_10avalanches_2simple_raster(__pyx_self, __pyx_v_X, __pyx_v_threshold);

  /* function exit code */
  __Pyx_RefNannyFinishContext();
  return __pyx_r;
}

static PyObject *__pyx_pf_10avalanches_2simple_raster(CYTHON_UNUSED PyObject *__pyx_self, __Pyx_memviewslice __pyx_v_X, double __pyx_v_threshold) {
  __Pyx_memviewslice __pyx_v_sd = { 0, 0, { 0 }, { 0 }, { 0 } };
  __Pyx_memviewslice __pyx_v_means = { 0, 0, { 0 }, { 0 }, { 0 } };
  int __pyx_v_N0;
  int __pyx_v_N1;
  __Pyx_memviewslice __pyx_v_raster = { 0, 0, { 0 }, { 0 }, { 0 } };
  int __pyx_v_i;
  int __pyx_v_j;
  PyObject *__pyx_r = NULL;
  __Pyx_RefNannyDeclarations
  __Pyx_RefNannySetupContext("simple_raster", 0);
/* … */
  /* function exit code */
  __pyx_L1_error:;
  __Pyx_XDECREF(__pyx_t_1);
  __Pyx_XDECREF(__pyx_t_2);
  __Pyx_XDECREF(__pyx_t_3);
  __Pyx_XDECREF(__pyx_t_4);
  __PYX_XDEC_MEMVIEW(&__pyx_t_5, 1);
  __Pyx_XDECREF(__pyx_t_6);
  __PYX_XDEC_MEMVIEW(&__pyx_t_7, 1);
  __Pyx_AddTraceback("avalanches.simple_raster", __pyx_clineno, __pyx_lineno, __pyx_filename);
  __pyx_r = NULL;
  __pyx_L0:;
  __PYX_XDEC_MEMVIEW(&__pyx_v_sd, 1);
  __PYX_XDEC_MEMVIEW(&__pyx_v_means, 1);
  __PYX_XDEC_MEMVIEW(&__pyx_v_raster, 1);
  __PYX_XDEC_MEMVIEW(&__pyx_v_X, 1);
  __Pyx_XGIVEREF(__pyx_r);
  __Pyx_RefNannyFinishContext();
  return __pyx_r;
}
/* … */
  __pyx_tuple__41 = PyTuple_Pack(9, __pyx_n_s_X, __pyx_n_s_threshold, __pyx_n_s_sd, __pyx_n_s_means, __pyx_n_s_N0, __pyx_n_s_N1, __pyx_n_s_raster, __pyx_n_s_i, __pyx_n_s_j); if (unlikely(!__pyx_tuple__41)) __PYX_ERR(0, 87, __pyx_L1_error)
  __Pyx_GOTREF(__pyx_tuple__41);
  __Pyx_GIVEREF(__pyx_tuple__41);
/* … */
  __pyx_t_2 = PyCFunction_NewEx(&__pyx_mdef_10avalanches_3simple_raster, NULL, __pyx_n_s_avalanches); if (unlikely(!__pyx_t_2)) __PYX_ERR(0, 87, __pyx_L1_error)
  __Pyx_GOTREF(__pyx_t_2);
  if (PyDict_SetItem(__pyx_d, __pyx_n_s_simple_raster, __pyx_t_2) < 0) __PYX_ERR(0, 87, __pyx_L1_error)
  __Pyx_DECREF(__pyx_t_2); __pyx_t_2 = 0;
  __pyx_codeobj__42 = (PyObject*)__Pyx_PyCode_New(2, 0, 9, 0, CO_OPTIMIZED|CO_NEWLOCALS, __pyx_empty_bytes, __pyx_empty_tuple, __pyx_empty_tuple, __pyx_tuple__41, __pyx_empty_tuple, __pyx_empty_tuple, __pyx_kp_s_avalanches_pyx, __pyx_n_s_simple_raster, 87, __pyx_empty_bytes); if (unlikely(!__pyx_codeobj__42)) __PYX_ERR(0, 87, __pyx_L1_error)
```

```
 088:     """
```

```
 089:     The simplest algorithm for identifying "events."
```

```
 090:     Places a 1 every time the time-series crosses a threshold (n * standard devation).
```

```
 091:
```

```
 092:     Arguments:
```

```
 093:
```

```
 094:         X:
```

```
 095:             A 2-dimensional Numpy array (dtype must be "double").
```

```
 096:         Threshold:
```

```
 097:             The number of standard deviations the time-series must exceed to be counted as an event.
```

```
 098:
```

```
 099:     Returns:
```

```
 100:
```

```
 101:         Raster:
```

```
 102:             A binarized copy of the original array.
```

```
 103:             Places a 1 everywhere the time-series crosses the threshold, 0 otherwise.
```

```
 104:     """
```

```
+105:     cdef double[:] sd = np.std(X, axis = 1)
```

```
  __pyx_t_1 = __Pyx_GetModuleGlobalName(__pyx_n_s_np); if (unlikely(!__pyx_t_1)) __PYX_ERR(0, 105, __pyx_L1_error)
  __Pyx_GOTREF(__pyx_t_1);
  __pyx_t_2 = __Pyx_PyObject_GetAttrStr(__pyx_t_1, __pyx_n_s_std); if (unlikely(!__pyx_t_2)) __PYX_ERR(0, 105, __pyx_L1_error)
  __Pyx_GOTREF(__pyx_t_2);
  __Pyx_DECREF(__pyx_t_1); __pyx_t_1 = 0;
  __pyx_t_1 = __pyx_memoryview_fromslice(__pyx_v_X, 2, (PyObject *(*)(char *)) __pyx_memview_get_double, (int (*)(char *, PyObject *)) __pyx_memview_set_double, 0);; if (unlikely(!__pyx_t_1)) __PYX_ERR(0, 105, __pyx_L1_error)
  __Pyx_GOTREF(__pyx_t_1);
  __pyx_t_3 = PyTuple_New(1); if (unlikely(!__pyx_t_3)) __PYX_ERR(0, 105, __pyx_L1_error)
  __Pyx_GOTREF(__pyx_t_3);
  __Pyx_GIVEREF(__pyx_t_1);
  PyTuple_SET_ITEM(__pyx_t_3, 0, __pyx_t_1);
  __pyx_t_1 = 0;
  __pyx_t_1 = __Pyx_PyDict_NewPresized(1); if (unlikely(!__pyx_t_1)) __PYX_ERR(0, 105, __pyx_L1_error)
  __Pyx_GOTREF(__pyx_t_1);
  if (PyDict_SetItem(__pyx_t_1, __pyx_n_s_axis, __pyx_int_1) < 0) __PYX_ERR(0, 105, __pyx_L1_error)
  __pyx_t_4 = __Pyx_PyObject_Call(__pyx_t_2, __pyx_t_3, __pyx_t_1); if (unlikely(!__pyx_t_4)) __PYX_ERR(0, 105, __pyx_L1_error)
  __Pyx_GOTREF(__pyx_t_4);
  __Pyx_DECREF(__pyx_t_2); __pyx_t_2 = 0;
  __Pyx_DECREF(__pyx_t_3); __pyx_t_3 = 0;
  __Pyx_DECREF(__pyx_t_1); __pyx_t_1 = 0;
  __pyx_t_5 = __Pyx_PyObject_to_MemoryviewSlice_ds_double(__pyx_t_4, PyBUF_WRITABLE); if (unlikely(!__pyx_t_5.memview)) __PYX_ERR(0, 105, __pyx_L1_error)
  __Pyx_DECREF(__pyx_t_4); __pyx_t_4 = 0;
  __pyx_v_sd = __pyx_t_5;
  __pyx_t_5.memview = NULL;
  __pyx_t_5.data = NULL;
```

```
+106:     cdef double[:] means = np.means(X, axis = 1)
```

```
  __pyx_t_4 = __Pyx_GetModuleGlobalName(__pyx_n_s_np); if (unlikely(!__pyx_t_4)) __PYX_ERR(0, 106, __pyx_L1_error)
  __Pyx_GOTREF(__pyx_t_4);
  __pyx_t_1 = __Pyx_PyObject_GetAttrStr(__pyx_t_4, __pyx_n_s_means); if (unlikely(!__pyx_t_1)) __PYX_ERR(0, 106, __pyx_L1_error)
  __Pyx_GOTREF(__pyx_t_1);
  __Pyx_DECREF(__pyx_t_4); __pyx_t_4 = 0;
  __pyx_t_4 = __pyx_memoryview_fromslice(__pyx_v_X, 2, (PyObject *(*)(char *)) __pyx_memview_get_double, (int (*)(char *, PyObject *)) __pyx_memview_set_double, 0);; if (unlikely(!__pyx_t_4)) __PYX_ERR(0, 106, __pyx_L1_error)
  __Pyx_GOTREF(__pyx_t_4);
  __pyx_t_3 = PyTuple_New(1); if (unlikely(!__pyx_t_3)) __PYX_ERR(0, 106, __pyx_L1_error)
  __Pyx_GOTREF(__pyx_t_3);
  __Pyx_GIVEREF(__pyx_t_4);
  PyTuple_SET_ITEM(__pyx_t_3, 0, __pyx_t_4);
  __pyx_t_4 = 0;
  __pyx_t_4 = __Pyx_PyDict_NewPresized(1); if (unlikely(!__pyx_t_4)) __PYX_ERR(0, 106, __pyx_L1_error)
  __Pyx_GOTREF(__pyx_t_4);
  if (PyDict_SetItem(__pyx_t_4, __pyx_n_s_axis, __pyx_int_1) < 0) __PYX_ERR(0, 106, __pyx_L1_error)
  __pyx_t_2 = __Pyx_PyObject_Call(__pyx_t_1, __pyx_t_3, __pyx_t_4); if (unlikely(!__pyx_t_2)) __PYX_ERR(0, 106, __pyx_L1_error)
  __Pyx_GOTREF(__pyx_t_2);
  __Pyx_DECREF(__pyx_t_1); __pyx_t_1 = 0;
  __Pyx_DECREF(__pyx_t_3); __pyx_t_3 = 0;
  __Pyx_DECREF(__pyx_t_4); __pyx_t_4 = 0;
  __pyx_t_5 = __Pyx_PyObject_to_MemoryviewSlice_ds_double(__pyx_t_2, PyBUF_WRITABLE); if (unlikely(!__pyx_t_5.memview)) __PYX_ERR(0, 106, __pyx_L1_error)
  __Pyx_DECREF(__pyx_t_2); __pyx_t_2 = 0;
  __pyx_v_means = __pyx_t_5;
  __pyx_t_5.memview = NULL;
  __pyx_t_5.data = NULL;
```

```
+107:     cdef int N0 = X.shape[0]
```

```
  __pyx_v_N0 = (__pyx_v_X.shape[0]);
```

```
+108:     cdef int N1 = X.shape[1]
```

```
  __pyx_v_N1 = (__pyx_v_X.shape[1]);
```

```
+109:     cdef double[:,:] raster = np.zeros((N0, N1))
```

```
  __pyx_t_4 = __Pyx_GetModuleGlobalName(__pyx_n_s_np); if (unlikely(!__pyx_t_4)) __PYX_ERR(0, 109, __pyx_L1_error)
  __Pyx_GOTREF(__pyx_t_4);
  __pyx_t_3 = __Pyx_PyObject_GetAttrStr(__pyx_t_4, __pyx_n_s_zeros); if (unlikely(!__pyx_t_3)) __PYX_ERR(0, 109, __pyx_L1_error)
  __Pyx_GOTREF(__pyx_t_3);
  __Pyx_DECREF(__pyx_t_4); __pyx_t_4 = 0;
  __pyx_t_4 = __Pyx_PyInt_From_int(__pyx_v_N0); if (unlikely(!__pyx_t_4)) __PYX_ERR(0, 109, __pyx_L1_error)
  __Pyx_GOTREF(__pyx_t_4);
  __pyx_t_1 = __Pyx_PyInt_From_int(__pyx_v_N1); if (unlikely(!__pyx_t_1)) __PYX_ERR(0, 109, __pyx_L1_error)
  __Pyx_GOTREF(__pyx_t_1);
  __pyx_t_6 = PyTuple_New(2); if (unlikely(!__pyx_t_6)) __PYX_ERR(0, 109, __pyx_L1_error)
  __Pyx_GOTREF(__pyx_t_6);
  __Pyx_GIVEREF(__pyx_t_4);
  PyTuple_SET_ITEM(__pyx_t_6, 0, __pyx_t_4);
  __Pyx_GIVEREF(__pyx_t_1);
  PyTuple_SET_ITEM(__pyx_t_6, 1, __pyx_t_1);
  __pyx_t_4 = 0;
  __pyx_t_1 = 0;
  __pyx_t_1 = NULL;
  if (CYTHON_UNPACK_METHODS && unlikely(PyMethod_Check(__pyx_t_3))) {
    __pyx_t_1 = PyMethod_GET_SELF(__pyx_t_3);
    if (likely(__pyx_t_1)) {
      PyObject* function = PyMethod_GET_FUNCTION(__pyx_t_3);
      __Pyx_INCREF(__pyx_t_1);
      __Pyx_INCREF(function);
      __Pyx_DECREF_SET(__pyx_t_3, function);
    }
  }
  if (!__pyx_t_1) {
    __pyx_t_2 = __Pyx_PyObject_CallOneArg(__pyx_t_3, __pyx_t_6); if (unlikely(!__pyx_t_2)) __PYX_ERR(0, 109, __pyx_L1_error)
    __Pyx_DECREF(__pyx_t_6); __pyx_t_6 = 0;
    __Pyx_GOTREF(__pyx_t_2);
  } else {
    #if CYTHON_FAST_PYCALL
    if (PyFunction_Check(__pyx_t_3)) {
      PyObject *__pyx_temp[2] = {__pyx_t_1, __pyx_t_6};
      __pyx_t_2 = __Pyx_PyFunction_FastCall(__pyx_t_3, __pyx_temp+1-1, 1+1); if (unlikely(!__pyx_t_2)) __PYX_ERR(0, 109, __pyx_L1_error)
      __Pyx_XDECREF(__pyx_t_1); __pyx_t_1 = 0;
      __Pyx_GOTREF(__pyx_t_2);
      __Pyx_DECREF(__pyx_t_6); __pyx_t_6 = 0;
    } else
    #endif
    #if CYTHON_FAST_PYCCALL
    if (__Pyx_PyFastCFunction_Check(__pyx_t_3)) {
      PyObject *__pyx_temp[2] = {__pyx_t_1, __pyx_t_6};
      __pyx_t_2 = __Pyx_PyCFunction_FastCall(__pyx_t_3, __pyx_temp+1-1, 1+1); if (unlikely(!__pyx_t_2)) __PYX_ERR(0, 109, __pyx_L1_error)
      __Pyx_XDECREF(__pyx_t_1); __pyx_t_1 = 0;
      __Pyx_GOTREF(__pyx_t_2);
      __Pyx_DECREF(__pyx_t_6); __pyx_t_6 = 0;
    } else
    #endif
    {
      __pyx_t_4 = PyTuple_New(1+1); if (unlikely(!__pyx_t_4)) __PYX_ERR(0, 109, __pyx_L1_error)
      __Pyx_GOTREF(__pyx_t_4);
      __Pyx_GIVEREF(__pyx_t_1); PyTuple_SET_ITEM(__pyx_t_4, 0, __pyx_t_1); __pyx_t_1 = NULL;
      __Pyx_GIVEREF(__pyx_t_6);
      PyTuple_SET_ITEM(__pyx_t_4, 0+1, __pyx_t_6);
      __pyx_t_6 = 0;
      __pyx_t_2 = __Pyx_PyObject_Call(__pyx_t_3, __pyx_t_4, NULL); if (unlikely(!__pyx_t_2)) __PYX_ERR(0, 109, __pyx_L1_error)
      __Pyx_GOTREF(__pyx_t_2);
      __Pyx_DECREF(__pyx_t_4); __pyx_t_4 = 0;
    }
  }
  __Pyx_DECREF(__pyx_t_3); __pyx_t_3 = 0;
  __pyx_t_7 = __Pyx_PyObject_to_MemoryviewSlice_dsds_double(__pyx_t_2, PyBUF_WRITABLE); if (unlikely(!__pyx_t_7.memview)) __PYX_ERR(0, 109, __pyx_L1_error)
  __Pyx_DECREF(__pyx_t_2); __pyx_t_2 = 0;
  __pyx_v_raster = __pyx_t_7;
  __pyx_t_7.memview = NULL;
  __pyx_t_7.data = NULL;
```

```
 110:     cdef int i, j
```

```
 111:
```

```
+112:     for i in range(N0):
```

```
  __pyx_t_8 = __pyx_v_N0;
  __pyx_t_9 = __pyx_t_8;
  for (__pyx_t_10 = 0; __pyx_t_10 < __pyx_t_9; __pyx_t_10+=1) {
    __pyx_v_i = __pyx_t_10;
```

```
+113:         j = 1
```

```
    __pyx_v_j = 1;
```

```
+114:         while j < N1:
```

```
    while (1) {
      __pyx_t_11 = ((__pyx_v_j < __pyx_v_N1) != 0);
      if (!__pyx_t_11) break;
```

```
 115:             # The positive extreme case:
```

```
+116:             if X[i][j] >= threshold * (means[i] + sd[i]) and X[i][j-1] < means[i] + (threshold * sd[i]):
```

```
      __pyx_t_12 = __pyx_v_i;
      __pyx_t_13 = __pyx_v_j;
      __pyx_t_14 = __pyx_v_i;
      __pyx_t_15 = __pyx_v_i;
      __pyx_t_16 = (((*((double *) ( /* dim=1 */ (( /* dim=0 */ (__pyx_v_X.data + __pyx_t_12 * __pyx_v_X.strides[0]) ) + __pyx_t_13 * __pyx_v_X.strides[1]) ))) >= (__pyx_v_threshold * ((*((double *) ( /* dim=0 */ (__pyx_v_means.data + __pyx_t_14 * __pyx_v_means.strides[0]) ))) + (*((double *) ( /* dim=0 */ (__pyx_v_sd.data + __pyx_t_15 * __pyx_v_sd.strides[0]) )))))) != 0);
      if (__pyx_t_16) {
      } else {
        __pyx_t_11 = __pyx_t_16;
        goto __pyx_L8_bool_binop_done;
      }
      __pyx_t_17 = __pyx_v_i;
      __pyx_t_18 = (__pyx_v_j - 1);
      __pyx_t_19 = __pyx_v_i;
      __pyx_t_20 = __pyx_v_i;
      __pyx_t_16 = (((*((double *) ( /* dim=1 */ (( /* dim=0 */ (__pyx_v_X.data + __pyx_t_17 * __pyx_v_X.strides[0]) ) + __pyx_t_18 * __pyx_v_X.strides[1]) ))) < ((*((double *) ( /* dim=0 */ (__pyx_v_means.data + __pyx_t_19 * __pyx_v_means.strides[0]) ))) + (__pyx_v_threshold * (*((double *) ( /* dim=0 */ (__pyx_v_sd.data + __pyx_t_20 * __pyx_v_sd.strides[0]) )))))) != 0);
      __pyx_t_11 = __pyx_t_16;
      __pyx_L8_bool_binop_done:;
      if (__pyx_t_11) {
/* … */
        goto __pyx_L7;
      }
```

```
+117:                 raster[i][j] = 1
```

```
        __pyx_t_21 = __pyx_v_i;
        __pyx_t_22 = __pyx_v_j;
        *((double *) ( /* dim=1 */ (( /* dim=0 */ (__pyx_v_raster.data + __pyx_t_21 * __pyx_v_raster.strides[0]) ) + __pyx_t_22 * __pyx_v_raster.strides[1]) )) = 1.0;
```

```
+118:                 j += 1
```

```
        __pyx_v_j = (__pyx_v_j + 1);
```

```
 119:
```

```
 120:             #The negative extreme case:
```

```
+121:             elif X[i][j] <= -1 * threshold * (means[i] + sd[i]) and X[i][j-1] > -1 * (means[i] + (threshold * sd[i])):
```

```
      __pyx_t_23 = __pyx_v_i;
      __pyx_t_24 = __pyx_v_j;
      __pyx_t_25 = __pyx_v_i;
      __pyx_t_26 = __pyx_v_i;
      __pyx_t_16 = (((*((double *) ( /* dim=1 */ (( /* dim=0 */ (__pyx_v_X.data + __pyx_t_23 * __pyx_v_X.strides[0]) ) + __pyx_t_24 * __pyx_v_X.strides[1]) ))) <= ((-1.0 * __pyx_v_threshold) * ((*((double *) ( /* dim=0 */ (__pyx_v_means.data + __pyx_t_25 * __pyx_v_means.strides[0]) ))) + (*((double *) ( /* dim=0 */ (__pyx_v_sd.data + __pyx_t_26 * __pyx_v_sd.strides[0]) )))))) != 0);
      if (__pyx_t_16) {
      } else {
        __pyx_t_11 = __pyx_t_16;
        goto __pyx_L10_bool_binop_done;
      }
      __pyx_t_27 = __pyx_v_i;
      __pyx_t_28 = (__pyx_v_j - 1);
      __pyx_t_29 = __pyx_v_i;
      __pyx_t_30 = __pyx_v_i;
      __pyx_t_16 = (((*((double *) ( /* dim=1 */ (( /* dim=0 */ (__pyx_v_X.data + __pyx_t_27 * __pyx_v_X.strides[0]) ) + __pyx_t_28 * __pyx_v_X.strides[1]) ))) > (-1.0 * ((*((double *) ( /* dim=0 */ (__pyx_v_means.data + __pyx_t_29 * __pyx_v_means.strides[0]) ))) + (__pyx_v_threshold * (*((double *) ( /* dim=0 */ (__pyx_v_sd.data + __pyx_t_30 * __pyx_v_sd.strides[0]) ))))))) != 0);
      __pyx_t_11 = __pyx_t_16;
      __pyx_L10_bool_binop_done:;
      if (__pyx_t_11) {
/* … */
        goto __pyx_L7;
      }
```

```
+122:                 raster[i][j] = 1
```

```
        __pyx_t_31 = __pyx_v_i;
        __pyx_t_32 = __pyx_v_j;
        *((double *) ( /* dim=1 */ (( /* dim=0 */ (__pyx_v_raster.data + __pyx_t_31 * __pyx_v_raster.strides[0]) ) + __pyx_t_32 * __pyx_v_raster.strides[1]) )) = 1.0;
```

```
+123:                 j += 1
```

```
        __pyx_v_j = (__pyx_v_j + 1);
```

```
 124:
```

```
 125:             #If the value X[i][j] does not break the threshold.
```

```
 126:             else:
```

```
+127:                 j += 1
```

```
      /*else*/ {
        __pyx_v_j = (__pyx_v_j + 1);
      }
      __pyx_L7:;
    }
  }
```

```
 128:
```

```
+129:     return raster
```

```
  __Pyx_XDECREF(__pyx_r);
  __pyx_t_2 = __pyx_memoryview_fromslice(__pyx_v_raster, 2, (PyObject *(*)(char *)) __pyx_memview_get_double, (int (*)(char *, PyObject *)) __pyx_memview_set_double, 0);; if (unlikely(!__pyx_t_2)) __PYX_ERR(0, 129, __pyx_L1_error)
  __Pyx_GOTREF(__pyx_t_2);
  __pyx_r = __pyx_t_2;
  __pyx_t_2 = 0;
  goto __pyx_L0;
```

```
 130:
```

```
 131: @cython.boundscheck(False)
```

```
 132: @cython.wraparound(False)
```

```
+133: def max_raster(double[:,:] X, double threshold):
```

```
/* Python wrapper */
static PyObject *__pyx_pw_10avalanches_5max_raster(PyObject *__pyx_self, PyObject *__pyx_args, PyObject *__pyx_kwds); /*proto*/
static PyMethodDef __pyx_mdef_10avalanches_5max_raster = {"max_raster", (PyCFunction)__pyx_pw_10avalanches_5max_raster, METH_VARARGS|METH_KEYWORDS, 0};
static PyObject *__pyx_pw_10avalanches_5max_raster(PyObject *__pyx_self, PyObject *__pyx_args, PyObject *__pyx_kwds) {
  __Pyx_memviewslice __pyx_v_X = { 0, 0, { 0 }, { 0 }, { 0 } };
  double __pyx_v_threshold;
  PyObject *__pyx_r = 0;
  __Pyx_RefNannyDeclarations
  __Pyx_RefNannySetupContext("max_raster (wrapper)", 0);
  {
    static PyObject **__pyx_pyargnames[] = {&__pyx_n_s_X,&__pyx_n_s_threshold,0};
    PyObject* values[2] = {0,0};
    if (unlikely(__pyx_kwds)) {
      Py_ssize_t kw_args;
      const Py_ssize_t pos_args = PyTuple_GET_SIZE(__pyx_args);
      switch (pos_args) {
        case  2: values[1] = PyTuple_GET_ITEM(__pyx_args, 1);
        CYTHON_FALLTHROUGH;
        case  1: values[0] = PyTuple_GET_ITEM(__pyx_args, 0);
        CYTHON_FALLTHROUGH;
        case  0: break;
        default: goto __pyx_L5_argtuple_error;
      }
      kw_args = PyDict_Size(__pyx_kwds);
      switch (pos_args) {
        case  0:
        if (likely((values[0] = __Pyx_PyDict_GetItemStr(__pyx_kwds, __pyx_n_s_X)) != 0)) kw_args--;
        else goto __pyx_L5_argtuple_error;
        CYTHON_FALLTHROUGH;
        case  1:
        if (likely((values[1] = __Pyx_PyDict_GetItemStr(__pyx_kwds, __pyx_n_s_threshold)) != 0)) kw_args--;
        else {
          __Pyx_RaiseArgtupleInvalid("max_raster", 1, 2, 2, 1); __PYX_ERR(0, 133, __pyx_L3_error)
        }
      }
      if (unlikely(kw_args > 0)) {
        if (unlikely(__Pyx_ParseOptionalKeywords(__pyx_kwds, __pyx_pyargnames, 0, values, pos_args, "max_raster") < 0)) __PYX_ERR(0, 133, __pyx_L3_error)
      }
    } else if (PyTuple_GET_SIZE(__pyx_args) != 2) {
      goto __pyx_L5_argtuple_error;
    } else {
      values[0] = PyTuple_GET_ITEM(__pyx_args, 0);
      values[1] = PyTuple_GET_ITEM(__pyx_args, 1);
    }
    __pyx_v_X = __Pyx_PyObject_to_MemoryviewSlice_dsds_double(values[0], PyBUF_WRITABLE); if (unlikely(!__pyx_v_X.memview)) __PYX_ERR(0, 133, __pyx_L3_error)
    __pyx_v_threshold = __pyx_PyFloat_AsDouble(values[1]); if (unlikely((__pyx_v_threshold == (double)-1) && PyErr_Occurred())) __PYX_ERR(0, 133, __pyx_L3_error)
  }
  goto __pyx_L4_argument_unpacking_done;
  __pyx_L5_argtuple_error:;
  __Pyx_RaiseArgtupleInvalid("max_raster", 1, 2, 2, PyTuple_GET_SIZE(__pyx_args)); __PYX_ERR(0, 133, __pyx_L3_error)
  __pyx_L3_error:;
  __Pyx_AddTraceback("avalanches.max_raster", __pyx_clineno, __pyx_lineno, __pyx_filename);
  __Pyx_RefNannyFinishContext();
  return NULL;
  __pyx_L4_argument_unpacking_done:;
  __pyx_r = __pyx_pf_10avalanches_4max_raster(__pyx_self, __pyx_v_X, __pyx_v_threshold);

  /* function exit code */
  __Pyx_RefNannyFinishContext();
  return __pyx_r;
}

static PyObject *__pyx_pf_10avalanches_4max_raster(CYTHON_UNUSED PyObject *__pyx_self, __Pyx_memviewslice __pyx_v_X, double __pyx_v_threshold) {
  __Pyx_memviewslice __pyx_v_sd = { 0, 0, { 0 }, { 0 }, { 0 } };
  __Pyx_memviewslice __pyx_v_means = { 0, 0, { 0 }, { 0 }, { 0 } };
  int __pyx_v_N0;
  int __pyx_v_N1;
  __Pyx_memviewslice __pyx_v_raster = { 0, 0, { 0 }, { 0 }, { 0 } };
  int __pyx_v_i;
  int __pyx_v_j;
  int __pyx_v_Nmax;
  int __pyx_v_Nmin;
  __Pyx_memviewslice __pyx_v_relmin = { 0, 0, { 0 }, { 0 }, { 0 } };
  __Pyx_memviewslice __pyx_v_relmax = { 0, 0, { 0 }, { 0 }, { 0 } };
  PyObject *__pyx_r = NULL;
  __Pyx_RefNannyDeclarations
  __Pyx_RefNannySetupContext("max_raster", 0);
/* … */
  /* function exit code */
  __pyx_L1_error:;
  __Pyx_XDECREF(__pyx_t_1);
  __Pyx_XDECREF(__pyx_t_2);
  __Pyx_XDECREF(__pyx_t_3);
  __Pyx_XDECREF(__pyx_t_4);
  __PYX_XDEC_MEMVIEW(&__pyx_t_5, 1);
  __Pyx_XDECREF(__pyx_t_6);
  __PYX_XDEC_MEMVIEW(&__pyx_t_7, 1);
  __PYX_XDEC_MEMVIEW(&__pyx_t_11, 1);
  __Pyx_AddTraceback("avalanches.max_raster", __pyx_clineno, __pyx_lineno, __pyx_filename);
  __pyx_r = NULL;
  __pyx_L0:;
  __PYX_XDEC_MEMVIEW(&__pyx_v_sd, 1);
  __PYX_XDEC_MEMVIEW(&__pyx_v_means, 1);
  __PYX_XDEC_MEMVIEW(&__pyx_v_raster, 1);
  __PYX_XDEC_MEMVIEW(&__pyx_v_relmin, 1);
  __PYX_XDEC_MEMVIEW(&__pyx_v_relmax, 1);
  __PYX_XDEC_MEMVIEW(&__pyx_v_X, 1);
  __Pyx_XGIVEREF(__pyx_r);
  __Pyx_RefNannyFinishContext();
  return __pyx_r;
}
/* … */
  __pyx_tuple__43 = PyTuple_Pack(13, __pyx_n_s_X, __pyx_n_s_threshold, __pyx_n_s_sd, __pyx_n_s_means, __pyx_n_s_N0, __pyx_n_s_N1, __pyx_n_s_raster, __pyx_n_s_i, __pyx_n_s_j, __pyx_n_s_Nmax, __pyx_n_s_Nmin, __pyx_n_s_relmin, __pyx_n_s_relmax); if (unlikely(!__pyx_tuple__43)) __PYX_ERR(0, 133, __pyx_L1_error)
  __Pyx_GOTREF(__pyx_tuple__43);
  __Pyx_GIVEREF(__pyx_tuple__43);
/* … */
  __pyx_t_2 = PyCFunction_NewEx(&__pyx_mdef_10avalanches_5max_raster, NULL, __pyx_n_s_avalanches); if (unlikely(!__pyx_t_2)) __PYX_ERR(0, 133, __pyx_L1_error)
  __Pyx_GOTREF(__pyx_t_2);
  if (PyDict_SetItem(__pyx_d, __pyx_n_s_max_raster, __pyx_t_2) < 0) __PYX_ERR(0, 133, __pyx_L1_error)
  __Pyx_DECREF(__pyx_t_2); __pyx_t_2 = 0;
  __pyx_codeobj__44 = (PyObject*)__Pyx_PyCode_New(2, 0, 13, 0, CO_OPTIMIZED|CO_NEWLOCALS, __pyx_empty_bytes, __pyx_empty_tuple, __pyx_empty_tuple, __pyx_tuple__43, __pyx_empty_tuple, __pyx_empty_tuple, __pyx_kp_s_avalanches_pyx, __pyx_n_s_max_raster, 133, __pyx_empty_bytes); if (unlikely(!__pyx_codeobj__44)) __PYX_ERR(0, 133, __pyx_L1_error)
```

```
+134:     cdef double[:] sd = np.std(X, axis = 1)
```

```
  __pyx_t_1 = __Pyx_GetModuleGlobalName(__pyx_n_s_np); if (unlikely(!__pyx_t_1)) __PYX_ERR(0, 134, __pyx_L1_error)
  __Pyx_GOTREF(__pyx_t_1);
  __pyx_t_2 = __Pyx_PyObject_GetAttrStr(__pyx_t_1, __pyx_n_s_std); if (unlikely(!__pyx_t_2)) __PYX_ERR(0, 134, __pyx_L1_error)
  __Pyx_GOTREF(__pyx_t_2);
  __Pyx_DECREF(__pyx_t_1); __pyx_t_1 = 0;
  __pyx_t_1 = __pyx_memoryview_fromslice(__pyx_v_X, 2, (PyObject *(*)(char *)) __pyx_memview_get_double, (int (*)(char *, PyObject *)) __pyx_memview_set_double, 0);; if (unlikely(!__pyx_t_1)) __PYX_ERR(0, 134, __pyx_L1_error)
  __Pyx_GOTREF(__pyx_t_1);
  __pyx_t_3 = PyTuple_New(1); if (unlikely(!__pyx_t_3)) __PYX_ERR(0, 134, __pyx_L1_error)
  __Pyx_GOTREF(__pyx_t_3);
  __Pyx_GIVEREF(__pyx_t_1);
  PyTuple_SET_ITEM(__pyx_t_3, 0, __pyx_t_1);
  __pyx_t_1 = 0;
  __pyx_t_1 = __Pyx_PyDict_NewPresized(1); if (unlikely(!__pyx_t_1)) __PYX_ERR(0, 134, __pyx_L1_error)
  __Pyx_GOTREF(__pyx_t_1);
  if (PyDict_SetItem(__pyx_t_1, __pyx_n_s_axis, __pyx_int_1) < 0) __PYX_ERR(0, 134, __pyx_L1_error)
  __pyx_t_4 = __Pyx_PyObject_Call(__pyx_t_2, __pyx_t_3, __pyx_t_1); if (unlikely(!__pyx_t_4)) __PYX_ERR(0, 134, __pyx_L1_error)
  __Pyx_GOTREF(__pyx_t_4);
  __Pyx_DECREF(__pyx_t_2); __pyx_t_2 = 0;
  __Pyx_DECREF(__pyx_t_3); __pyx_t_3 = 0;
  __Pyx_DECREF(__pyx_t_1); __pyx_t_1 = 0;
  __pyx_t_5 = __Pyx_PyObject_to_MemoryviewSlice_ds_double(__pyx_t_4, PyBUF_WRITABLE); if (unlikely(!__pyx_t_5.memview)) __PYX_ERR(0, 134, __pyx_L1_error)
  __Pyx_DECREF(__pyx_t_4); __pyx_t_4 = 0;
  __pyx_v_sd = __pyx_t_5;
  __pyx_t_5.memview = NULL;
  __pyx_t_5.data = NULL;
```

```
+135:     cdef double[:] means = np.mean(X, axis = 1)
```

```
  __pyx_t_4 = __Pyx_GetModuleGlobalName(__pyx_n_s_np); if (unlikely(!__pyx_t_4)) __PYX_ERR(0, 135, __pyx_L1_error)
  __Pyx_GOTREF(__pyx_t_4);
  __pyx_t_1 = __Pyx_PyObject_GetAttrStr(__pyx_t_4, __pyx_n_s_mean); if (unlikely(!__pyx_t_1)) __PYX_ERR(0, 135, __pyx_L1_error)
  __Pyx_GOTREF(__pyx_t_1);
  __Pyx_DECREF(__pyx_t_4); __pyx_t_4 = 0;
  __pyx_t_4 = __pyx_memoryview_fromslice(__pyx_v_X, 2, (PyObject *(*)(char *)) __pyx_memview_get_double, (int (*)(char *, PyObject *)) __pyx_memview_set_double, 0);; if (unlikely(!__pyx_t_4)) __PYX_ERR(0, 135, __pyx_L1_error)
  __Pyx_GOTREF(__pyx_t_4);
  __pyx_t_3 = PyTuple_New(1); if (unlikely(!__pyx_t_3)) __PYX_ERR(0, 135, __pyx_L1_error)
  __Pyx_GOTREF(__pyx_t_3);
  __Pyx_GIVEREF(__pyx_t_4);
  PyTuple_SET_ITEM(__pyx_t_3, 0, __pyx_t_4);
  __pyx_t_4 = 0;
  __pyx_t_4 = __Pyx_PyDict_NewPresized(1); if (unlikely(!__pyx_t_4)) __PYX_ERR(0, 135, __pyx_L1_error)
  __Pyx_GOTREF(__pyx_t_4);
  if (PyDict_SetItem(__pyx_t_4, __pyx_n_s_axis, __pyx_int_1) < 0) __PYX_ERR(0, 135, __pyx_L1_error)
  __pyx_t_2 = __Pyx_PyObject_Call(__pyx_t_1, __pyx_t_3, __pyx_t_4); if (unlikely(!__pyx_t_2)) __PYX_ERR(0, 135, __pyx_L1_error)
  __Pyx_GOTREF(__pyx_t_2);
  __Pyx_DECREF(__pyx_t_1); __pyx_t_1 = 0;
  __Pyx_DECREF(__pyx_t_3); __pyx_t_3 = 0;
  __Pyx_DECREF(__pyx_t_4); __pyx_t_4 = 0;
  __pyx_t_5 = __Pyx_PyObject_to_MemoryviewSlice_ds_double(__pyx_t_2, PyBUF_WRITABLE); if (unlikely(!__pyx_t_5.memview)) __PYX_ERR(0, 135, __pyx_L1_error)
  __Pyx_DECREF(__pyx_t_2); __pyx_t_2 = 0;
  __pyx_v_means = __pyx_t_5;
  __pyx_t_5.memview = NULL;
  __pyx_t_5.data = NULL;
```

```
+136:     cdef int N0 = X.shape[0]
```

```
  __pyx_v_N0 = (__pyx_v_X.shape[0]);
```

```
+137:     cdef int N1 = X.shape[1]
```

```
  __pyx_v_N1 = (__pyx_v_X.shape[1]);
```

```
+138:     cdef double[:,:] raster = np.zeros((N0, N1))
```

```
  __pyx_t_4 = __Pyx_GetModuleGlobalName(__pyx_n_s_np); if (unlikely(!__pyx_t_4)) __PYX_ERR(0, 138, __pyx_L1_error)
  __Pyx_GOTREF(__pyx_t_4);
  __pyx_t_3 = __Pyx_PyObject_GetAttrStr(__pyx_t_4, __pyx_n_s_zeros); if (unlikely(!__pyx_t_3)) __PYX_ERR(0, 138, __pyx_L1_error)
  __Pyx_GOTREF(__pyx_t_3);
  __Pyx_DECREF(__pyx_t_4); __pyx_t_4 = 0;
  __pyx_t_4 = __Pyx_PyInt_From_int(__pyx_v_N0); if (unlikely(!__pyx_t_4)) __PYX_ERR(0, 138, __pyx_L1_error)
  __Pyx_GOTREF(__pyx_t_4);
  __pyx_t_1 = __Pyx_PyInt_From_int(__pyx_v_N1); if (unlikely(!__pyx_t_1)) __PYX_ERR(0, 138, __pyx_L1_error)
  __Pyx_GOTREF(__pyx_t_1);
  __pyx_t_6 = PyTuple_New(2); if (unlikely(!__pyx_t_6)) __PYX_ERR(0, 138, __pyx_L1_error)
  __Pyx_GOTREF(__pyx_t_6);
  __Pyx_GIVEREF(__pyx_t_4);
  PyTuple_SET_ITEM(__pyx_t_6, 0, __pyx_t_4);
  __Pyx_GIVEREF(__pyx_t_1);
  PyTuple_SET_ITEM(__pyx_t_6, 1, __pyx_t_1);
  __pyx_t_4 = 0;
  __pyx_t_1 = 0;
  __pyx_t_1 = NULL;
  if (CYTHON_UNPACK_METHODS && unlikely(PyMethod_Check(__pyx_t_3))) {
    __pyx_t_1 = PyMethod_GET_SELF(__pyx_t_3);
    if (likely(__pyx_t_1)) {
      PyObject* function = PyMethod_GET_FUNCTION(__pyx_t_3);
      __Pyx_INCREF(__pyx_t_1);
      __Pyx_INCREF(function);
      __Pyx_DECREF_SET(__pyx_t_3, function);
    }
  }
  if (!__pyx_t_1) {
    __pyx_t_2 = __Pyx_PyObject_CallOneArg(__pyx_t_3, __pyx_t_6); if (unlikely(!__pyx_t_2)) __PYX_ERR(0, 138, __pyx_L1_error)
    __Pyx_DECREF(__pyx_t_6); __pyx_t_6 = 0;
    __Pyx_GOTREF(__pyx_t_2);
  } else {
    #if CYTHON_FAST_PYCALL
    if (PyFunction_Check(__pyx_t_3)) {
      PyObject *__pyx_temp[2] = {__pyx_t_1, __pyx_t_6};
      __pyx_t_2 = __Pyx_PyFunction_FastCall(__pyx_t_3, __pyx_temp+1-1, 1+1); if (unlikely(!__pyx_t_2)) __PYX_ERR(0, 138, __pyx_L1_error)
      __Pyx_XDECREF(__pyx_t_1); __pyx_t_1 = 0;
      __Pyx_GOTREF(__pyx_t_2);
      __Pyx_DECREF(__pyx_t_6); __pyx_t_6 = 0;
    } else
    #endif
    #if CYTHON_FAST_PYCCALL
    if (__Pyx_PyFastCFunction_Check(__pyx_t_3)) {
      PyObject *__pyx_temp[2] = {__pyx_t_1, __pyx_t_6};
      __pyx_t_2 = __Pyx_PyCFunction_FastCall(__pyx_t_3, __pyx_temp+1-1, 1+1); if (unlikely(!__pyx_t_2)) __PYX_ERR(0, 138, __pyx_L1_error)
      __Pyx_XDECREF(__pyx_t_1); __pyx_t_1 = 0;
      __Pyx_GOTREF(__pyx_t_2);
      __Pyx_DECREF(__pyx_t_6); __pyx_t_6 = 0;
    } else
    #endif
    {
      __pyx_t_4 = PyTuple_New(1+1); if (unlikely(!__pyx_t_4)) __PYX_ERR(0, 138, __pyx_L1_error)
      __Pyx_GOTREF(__pyx_t_4);
      __Pyx_GIVEREF(__pyx_t_1); PyTuple_SET_ITEM(__pyx_t_4, 0, __pyx_t_1); __pyx_t_1 = NULL;
      __Pyx_GIVEREF(__pyx_t_6);
      PyTuple_SET_ITEM(__pyx_t_4, 0+1, __pyx_t_6);
      __pyx_t_6 = 0;
      __pyx_t_2 = __Pyx_PyObject_Call(__pyx_t_3, __pyx_t_4, NULL); if (unlikely(!__pyx_t_2)) __PYX_ERR(0, 138, __pyx_L1_error)
      __Pyx_GOTREF(__pyx_t_2);
      __Pyx_DECREF(__pyx_t_4); __pyx_t_4 = 0;
    }
  }
  __Pyx_DECREF(__pyx_t_3); __pyx_t_3 = 0;
  __pyx_t_7 = __Pyx_PyObject_to_MemoryviewSlice_dsds_double(__pyx_t_2, PyBUF_WRITABLE); if (unlikely(!__pyx_t_7.memview)) __PYX_ERR(0, 138, __pyx_L1_error)
  __Pyx_DECREF(__pyx_t_2); __pyx_t_2 = 0;
  __pyx_v_raster = __pyx_t_7;
  __pyx_t_7.memview = NULL;
  __pyx_t_7.data = NULL;
```

```
 139:     cdef int i, j, Nmax, Nmin
```

```
 140:     cdef int[:] relmin, relmax
```

```
 141:
```

```
+142:     for i in range(N0):
```

```
  __pyx_t_8 = __pyx_v_N0;
  __pyx_t_9 = __pyx_t_8;
  for (__pyx_t_10 = 0; __pyx_t_10 < __pyx_t_9; __pyx_t_10+=1) {
    __pyx_v_i = __pyx_t_10;
```

```
+143:         relmax = peak_finder(X[i], extrema = 1)[0].astype("int32")
```

```
    __pyx_t_2 = __Pyx_GetModuleGlobalName(__pyx_n_s_peak_finder); if (unlikely(!__pyx_t_2)) __PYX_ERR(0, 143, __pyx_L1_error)
    __Pyx_GOTREF(__pyx_t_2);
    __pyx_t_5.data = __pyx_v_X.data;
    __pyx_t_5.memview = __pyx_v_X.memview;
    __PYX_INC_MEMVIEW(&__pyx_t_5, 0);
    {
    Py_ssize_t __pyx_tmp_idx = __pyx_v_i;
    Py_ssize_t __pyx_tmp_shape = __pyx_v_X.shape[0];
    Py_ssize_t __pyx_tmp_stride = __pyx_v_X.strides[0];
    if (0 && (__pyx_tmp_idx < 0))
        __pyx_tmp_idx += __pyx_tmp_shape;
    if (0 && (__pyx_tmp_idx < 0 || __pyx_tmp_idx >= __pyx_tmp_shape)) {
        PyErr_SetString(PyExc_IndexError, "Index out of bounds (axis 0)");
        __PYX_ERR(0, 143, __pyx_L1_error)
    }
        __pyx_t_5.data += __pyx_tmp_idx * __pyx_tmp_stride;
}

__pyx_t_5.shape[0] = __pyx_v_X.shape[1];
__pyx_t_5.strides[0] = __pyx_v_X.strides[1];
    __pyx_t_5.suboffsets[0] = -1;

__pyx_t_3 = __pyx_memoryview_fromslice(__pyx_t_5, 1, (PyObject *(*)(char *)) __pyx_memview_get_double, (int (*)(char *, PyObject *)) __pyx_memview_set_double, 0);; if (unlikely(!__pyx_t_3)) __PYX_ERR(0, 143, __pyx_L1_error)
    __Pyx_GOTREF(__pyx_t_3);
    __PYX_XDEC_MEMVIEW(&__pyx_t_5, 1);
    __pyx_t_5.memview = NULL;
    __pyx_t_5.data = NULL;
    __pyx_t_4 = PyTuple_New(1); if (unlikely(!__pyx_t_4)) __PYX_ERR(0, 143, __pyx_L1_error)
    __Pyx_GOTREF(__pyx_t_4);
    __Pyx_GIVEREF(__pyx_t_3);
    PyTuple_SET_ITEM(__pyx_t_4, 0, __pyx_t_3);
    __pyx_t_3 = 0;
    __pyx_t_3 = __Pyx_PyDict_NewPresized(1); if (unlikely(!__pyx_t_3)) __PYX_ERR(0, 143, __pyx_L1_error)
    __Pyx_GOTREF(__pyx_t_3);
    if (PyDict_SetItem(__pyx_t_3, __pyx_n_s_extrema, __pyx_int_1) < 0) __PYX_ERR(0, 143, __pyx_L1_error)
    __pyx_t_6 = __Pyx_PyObject_Call(__pyx_t_2, __pyx_t_4, __pyx_t_3); if (unlikely(!__pyx_t_6)) __PYX_ERR(0, 143, __pyx_L1_error)
    __Pyx_GOTREF(__pyx_t_6);
    __Pyx_DECREF(__pyx_t_2); __pyx_t_2 = 0;
    __Pyx_DECREF(__pyx_t_4); __pyx_t_4 = 0;
    __Pyx_DECREF(__pyx_t_3); __pyx_t_3 = 0;
    __pyx_t_3 = __Pyx_GetItemInt(__pyx_t_6, 0, long, 1, __Pyx_PyInt_From_long, 0, 0, 0); if (unlikely(!__pyx_t_3)) __PYX_ERR(0, 143, __pyx_L1_error)
    __Pyx_GOTREF(__pyx_t_3);
    __Pyx_DECREF(__pyx_t_6); __pyx_t_6 = 0;
    __pyx_t_6 = __Pyx_PyObject_GetAttrStr(__pyx_t_3, __pyx_n_s_astype); if (unlikely(!__pyx_t_6)) __PYX_ERR(0, 143, __pyx_L1_error)
    __Pyx_GOTREF(__pyx_t_6);
    __Pyx_DECREF(__pyx_t_3); __pyx_t_3 = 0;
    __pyx_t_3 = __Pyx_PyObject_Call(__pyx_t_6, __pyx_tuple_, NULL); if (unlikely(!__pyx_t_3)) __PYX_ERR(0, 143, __pyx_L1_error)
    __Pyx_GOTREF(__pyx_t_3);
    __Pyx_DECREF(__pyx_t_6); __pyx_t_6 = 0;
    __pyx_t_11 = __Pyx_PyObject_to_MemoryviewSlice_ds_int(__pyx_t_3, PyBUF_WRITABLE); if (unlikely(!__pyx_t_11.memview)) __PYX_ERR(0, 143, __pyx_L1_error)
    __Pyx_DECREF(__pyx_t_3); __pyx_t_3 = 0;
    __PYX_XDEC_MEMVIEW(&__pyx_v_relmax, 1);
    __pyx_v_relmax = __pyx_t_11;
    __pyx_t_11.memview = NULL;
    __pyx_t_11.data = NULL;
/* … */
  __pyx_tuple_ = PyTuple_Pack(1, __pyx_n_s_int32); if (unlikely(!__pyx_tuple_)) __PYX_ERR(0, 143, __pyx_L1_error)
  __Pyx_GOTREF(__pyx_tuple_);
  __Pyx_GIVEREF(__pyx_tuple_);
```

```
+144:         relmin = peak_finder(X[i], extrema = -1)[0].astype("int32")
```

```
    __pyx_t_3 = __Pyx_GetModuleGlobalName(__pyx_n_s_peak_finder); if (unlikely(!__pyx_t_3)) __PYX_ERR(0, 144, __pyx_L1_error)
    __Pyx_GOTREF(__pyx_t_3);
    __pyx_t_5.data = __pyx_v_X.data;
    __pyx_t_5.memview = __pyx_v_X.memview;
    __PYX_INC_MEMVIEW(&__pyx_t_5, 0);
    {
    Py_ssize_t __pyx_tmp_idx = __pyx_v_i;
    Py_ssize_t __pyx_tmp_shape = __pyx_v_X.shape[0];
    Py_ssize_t __pyx_tmp_stride = __pyx_v_X.strides[0];
    if (0 && (__pyx_tmp_idx < 0))
        __pyx_tmp_idx += __pyx_tmp_shape;
    if (0 && (__pyx_tmp_idx < 0 || __pyx_tmp_idx >= __pyx_tmp_shape)) {
        PyErr_SetString(PyExc_IndexError, "Index out of bounds (axis 0)");
        __PYX_ERR(0, 144, __pyx_L1_error)
    }
        __pyx_t_5.data += __pyx_tmp_idx * __pyx_tmp_stride;
}

__pyx_t_5.shape[0] = __pyx_v_X.shape[1];
__pyx_t_5.strides[0] = __pyx_v_X.strides[1];
    __pyx_t_5.suboffsets[0] = -1;

__pyx_t_6 = __pyx_memoryview_fromslice(__pyx_t_5, 1, (PyObject *(*)(char *)) __pyx_memview_get_double, (int (*)(char *, PyObject *)) __pyx_memview_set_double, 0);; if (unlikely(!__pyx_t_6)) __PYX_ERR(0, 144, __pyx_L1_error)
    __Pyx_GOTREF(__pyx_t_6);
    __PYX_XDEC_MEMVIEW(&__pyx_t_5, 1);
    __pyx_t_5.memview = NULL;
    __pyx_t_5.data = NULL;
    __pyx_t_4 = PyTuple_New(1); if (unlikely(!__pyx_t_4)) __PYX_ERR(0, 144, __pyx_L1_error)
    __Pyx_GOTREF(__pyx_t_4);
    __Pyx_GIVEREF(__pyx_t_6);
    PyTuple_SET_ITEM(__pyx_t_4, 0, __pyx_t_6);
    __pyx_t_6 = 0;
    __pyx_t_6 = __Pyx_PyDict_NewPresized(1); if (unlikely(!__pyx_t_6)) __PYX_ERR(0, 144, __pyx_L1_error)
    __Pyx_GOTREF(__pyx_t_6);
    if (PyDict_SetItem(__pyx_t_6, __pyx_n_s_extrema, __pyx_int_neg_1) < 0) __PYX_ERR(0, 144, __pyx_L1_error)
    __pyx_t_2 = __Pyx_PyObject_Call(__pyx_t_3, __pyx_t_4, __pyx_t_6); if (unlikely(!__pyx_t_2)) __PYX_ERR(0, 144, __pyx_L1_error)
    __Pyx_GOTREF(__pyx_t_2);
    __Pyx_DECREF(__pyx_t_3); __pyx_t_3 = 0;
    __Pyx_DECREF(__pyx_t_4); __pyx_t_4 = 0;
    __Pyx_DECREF(__pyx_t_6); __pyx_t_6 = 0;
    __pyx_t_6 = __Pyx_GetItemInt(__pyx_t_2, 0, long, 1, __Pyx_PyInt_From_long, 0, 0, 0); if (unlikely(!__pyx_t_6)) __PYX_ERR(0, 144, __pyx_L1_error)
    __Pyx_GOTREF(__pyx_t_6);
    __Pyx_DECREF(__pyx_t_2); __pyx_t_2 = 0;
    __pyx_t_2 = __Pyx_PyObject_GetAttrStr(__pyx_t_6, __pyx_n_s_astype); if (unlikely(!__pyx_t_2)) __PYX_ERR(0, 144, __pyx_L1_error)
    __Pyx_GOTREF(__pyx_t_2);
    __Pyx_DECREF(__pyx_t_6); __pyx_t_6 = 0;
    __pyx_t_6 = __Pyx_PyObject_Call(__pyx_t_2, __pyx_tuple__2, NULL); if (unlikely(!__pyx_t_6)) __PYX_ERR(0, 144, __pyx_L1_error)
    __Pyx_GOTREF(__pyx_t_6);
    __Pyx_DECREF(__pyx_t_2); __pyx_t_2 = 0;
    __pyx_t_11 = __Pyx_PyObject_to_MemoryviewSlice_ds_int(__pyx_t_6, PyBUF_WRITABLE); if (unlikely(!__pyx_t_11.memview)) __PYX_ERR(0, 144, __pyx_L1_error)
    __Pyx_DECREF(__pyx_t_6); __pyx_t_6 = 0;
    __PYX_XDEC_MEMVIEW(&__pyx_v_relmin, 1);
    __pyx_v_relmin = __pyx_t_11;
    __pyx_t_11.memview = NULL;
    __pyx_t_11.data = NULL;
/* … */
  __pyx_tuple__2 = PyTuple_Pack(1, __pyx_n_s_int32); if (unlikely(!__pyx_tuple__2)) __PYX_ERR(0, 144, __pyx_L1_error)
  __Pyx_GOTREF(__pyx_tuple__2);
  __Pyx_GIVEREF(__pyx_tuple__2);
```

```
 145:
```

```
+146:         Nmax = relmax.shape[0]
```

```
    __pyx_v_Nmax = (__pyx_v_relmax.shape[0]);
```

```
+147:         Nmin = relmin.shape[0]
```

```
    __pyx_v_Nmin = (__pyx_v_relmin.shape[0]);
```

```
 148:
```

```
+149:         for j in range(Nmax):
```

```
    __pyx_t_12 = __pyx_v_Nmax;
    __pyx_t_13 = __pyx_t_12;
    for (__pyx_t_14 = 0; __pyx_t_14 < __pyx_t_13; __pyx_t_14+=1) {
      __pyx_v_j = __pyx_t_14;
```

```
+150:             if X[i][relmax[j]] >= means[i] + (threshold * sd[i]):
```

```
      __pyx_t_15 = __pyx_v_j;
      __pyx_t_16 = __pyx_v_i;
      __pyx_t_17 = (*((int *) ( /* dim=0 */ (__pyx_v_relmax.data + __pyx_t_15 * __pyx_v_relmax.strides[0]) )));
      __pyx_t_18 = __pyx_v_i;
      __pyx_t_19 = __pyx_v_i;
      __pyx_t_20 = (((*((double *) ( /* dim=1 */ (( /* dim=0 */ (__pyx_v_X.data + __pyx_t_16 * __pyx_v_X.strides[0]) ) + __pyx_t_17 * __pyx_v_X.strides[1]) ))) >= ((*((double *) ( /* dim=0 */ (__pyx_v_means.data + __pyx_t_18 * __pyx_v_means.strides[0]) ))) + (__pyx_v_threshold * (*((double *) ( /* dim=0 */ (__pyx_v_sd.data + __pyx_t_19 * __pyx_v_sd.strides[0]) )))))) != 0);
      if (__pyx_t_20) {
/* … */
      }
    }
```

```
+151:                 raster[i][relmax[j]] = 1
```

```
        __pyx_t_21 = __pyx_v_j;
        __pyx_t_22 = __pyx_v_i;
        __pyx_t_23 = (*((int *) ( /* dim=0 */ (__pyx_v_relmax.data + __pyx_t_21 * __pyx_v_relmax.strides[0]) )));
        *((double *) ( /* dim=1 */ (( /* dim=0 */ (__pyx_v_raster.data + __pyx_t_22 * __pyx_v_raster.strides[0]) ) + __pyx_t_23 * __pyx_v_raster.strides[1]) )) = 1.0;
```

```
+152:         for j in range(Nmin):
```

```
    __pyx_t_12 = __pyx_v_Nmin;
    __pyx_t_13 = __pyx_t_12;
    for (__pyx_t_14 = 0; __pyx_t_14 < __pyx_t_13; __pyx_t_14+=1) {
      __pyx_v_j = __pyx_t_14;
```

```
+153:             if X[i][relmin[j]] <= -1 * (means[i] + (threshold * sd[i])):
```

```
      __pyx_t_24 = __pyx_v_j;
      __pyx_t_25 = __pyx_v_i;
      __pyx_t_26 = (*((int *) ( /* dim=0 */ (__pyx_v_relmin.data + __pyx_t_24 * __pyx_v_relmin.strides[0]) )));
      __pyx_t_27 = __pyx_v_i;
      __pyx_t_28 = __pyx_v_i;
      __pyx_t_20 = (((*((double *) ( /* dim=1 */ (( /* dim=0 */ (__pyx_v_X.data + __pyx_t_25 * __pyx_v_X.strides[0]) ) + __pyx_t_26 * __pyx_v_X.strides[1]) ))) <= (-1.0 * ((*((double *) ( /* dim=0 */ (__pyx_v_means.data + __pyx_t_27 * __pyx_v_means.strides[0]) ))) + (__pyx_v_threshold * (*((double *) ( /* dim=0 */ (__pyx_v_sd.data + __pyx_t_28 * __pyx_v_sd.strides[0]) ))))))) != 0);
      if (__pyx_t_20) {
/* … */
      }
    }
  }
```

```
+154:                 raster[i][relmin[j]] = 1
```

```
        __pyx_t_29 = __pyx_v_j;
        __pyx_t_30 = __pyx_v_i;
        __pyx_t_31 = (*((int *) ( /* dim=0 */ (__pyx_v_relmin.data + __pyx_t_29 * __pyx_v_relmin.strides[0]) )));
        *((double *) ( /* dim=1 */ (( /* dim=0 */ (__pyx_v_raster.data + __pyx_t_30 * __pyx_v_raster.strides[0]) ) + __pyx_t_31 * __pyx_v_raster.strides[1]) )) = 1.0;
```

```
 155:
```

```
+156:     return raster
```

```
  __Pyx_XDECREF(__pyx_r);
  __pyx_t_6 = __pyx_memoryview_fromslice(__pyx_v_raster, 2, (PyObject *(*)(char *)) __pyx_memview_get_double, (int (*)(char *, PyObject *)) __pyx_memview_set_double, 0);; if (unlikely(!__pyx_t_6)) __PYX_ERR(0, 156, __pyx_L1_error)
  __Pyx_GOTREF(__pyx_t_6);
  __pyx_r = __pyx_t_6;
  __pyx_t_6 = 0;
  goto __pyx_L0;
```

```
 157:
```

```
 158: @cython.boundscheck(False)
```

```
 159: @cython.wraparound(False)
```

```
+160: def coherence_raster(double[:,:] X, double threshold, double R):
```

```
/* Python wrapper */
static PyObject *__pyx_pw_10avalanches_7coherence_raster(PyObject *__pyx_self, PyObject *__pyx_args, PyObject *__pyx_kwds); /*proto*/
static PyMethodDef __pyx_mdef_10avalanches_7coherence_raster = {"coherence_raster", (PyCFunction)__pyx_pw_10avalanches_7coherence_raster, METH_VARARGS|METH_KEYWORDS, 0};
static PyObject *__pyx_pw_10avalanches_7coherence_raster(PyObject *__pyx_self, PyObject *__pyx_args, PyObject *__pyx_kwds) {
  __Pyx_memviewslice __pyx_v_X = { 0, 0, { 0 }, { 0 }, { 0 } };
  double __pyx_v_threshold;
  double __pyx_v_R;
  PyObject *__pyx_r = 0;
  __Pyx_RefNannyDeclarations
  __Pyx_RefNannySetupContext("coherence_raster (wrapper)", 0);
  {
    static PyObject **__pyx_pyargnames[] = {&__pyx_n_s_X,&__pyx_n_s_threshold,&__pyx_n_s_R,0};
    PyObject* values[3] = {0,0,0};
    if (unlikely(__pyx_kwds)) {
      Py_ssize_t kw_args;
      const Py_ssize_t pos_args = PyTuple_GET_SIZE(__pyx_args);
      switch (pos_args) {
        case  3: values[2] = PyTuple_GET_ITEM(__pyx_args, 2);
        CYTHON_FALLTHROUGH;
        case  2: values[1] = PyTuple_GET_ITEM(__pyx_args, 1);
        CYTHON_FALLTHROUGH;
        case  1: values[0] = PyTuple_GET_ITEM(__pyx_args, 0);
        CYTHON_FALLTHROUGH;
        case  0: break;
        default: goto __pyx_L5_argtuple_error;
      }
      kw_args = PyDict_Size(__pyx_kwds);
      switch (pos_args) {
        case  0:
        if (likely((values[0] = __Pyx_PyDict_GetItemStr(__pyx_kwds, __pyx_n_s_X)) != 0)) kw_args--;
        else goto __pyx_L5_argtuple_error;
        CYTHON_FALLTHROUGH;
        case  1:
        if (likely((values[1] = __Pyx_PyDict_GetItemStr(__pyx_kwds, __pyx_n_s_threshold)) != 0)) kw_args--;
        else {
          __Pyx_RaiseArgtupleInvalid("coherence_raster", 1, 3, 3, 1); __PYX_ERR(0, 160, __pyx_L3_error)
        }
        CYTHON_FALLTHROUGH;
        case  2:
        if (likely((values[2] = __Pyx_PyDict_GetItemStr(__pyx_kwds, __pyx_n_s_R)) != 0)) kw_args--;
        else {
          __Pyx_RaiseArgtupleInvalid("coherence_raster", 1, 3, 3, 2); __PYX_ERR(0, 160, __pyx_L3_error)
        }
      }
      if (unlikely(kw_args > 0)) {
        if (unlikely(__Pyx_ParseOptionalKeywords(__pyx_kwds, __pyx_pyargnames, 0, values, pos_args, "coherence_raster") < 0)) __PYX_ERR(0, 160, __pyx_L3_error)
      }
    } else if (PyTuple_GET_SIZE(__pyx_args) != 3) {
      goto __pyx_L5_argtuple_error;
    } else {
      values[0] = PyTuple_GET_ITEM(__pyx_args, 0);
      values[1] = PyTuple_GET_ITEM(__pyx_args, 1);
      values[2] = PyTuple_GET_ITEM(__pyx_args, 2);
    }
    __pyx_v_X = __Pyx_PyObject_to_MemoryviewSlice_dsds_double(values[0], PyBUF_WRITABLE); if (unlikely(!__pyx_v_X.memview)) __PYX_ERR(0, 160, __pyx_L3_error)
    __pyx_v_threshold = __pyx_PyFloat_AsDouble(values[1]); if (unlikely((__pyx_v_threshold == (double)-1) && PyErr_Occurred())) __PYX_ERR(0, 160, __pyx_L3_error)
    __pyx_v_R = __pyx_PyFloat_AsDouble(values[2]); if (unlikely((__pyx_v_R == (double)-1) && PyErr_Occurred())) __PYX_ERR(0, 160, __pyx_L3_error)
  }
  goto __pyx_L4_argument_unpacking_done;
  __pyx_L5_argtuple_error:;
  __Pyx_RaiseArgtupleInvalid("coherence_raster", 1, 3, 3, PyTuple_GET_SIZE(__pyx_args)); __PYX_ERR(0, 160, __pyx_L3_error)
  __pyx_L3_error:;
  __Pyx_AddTraceback("avalanches.coherence_raster", __pyx_clineno, __pyx_lineno, __pyx_filename);
  __Pyx_RefNannyFinishContext();
  return NULL;
  __pyx_L4_argument_unpacking_done:;
  __pyx_r = __pyx_pf_10avalanches_6coherence_raster(__pyx_self, __pyx_v_X, __pyx_v_threshold, __pyx_v_R);

  /* function exit code */
  __Pyx_RefNannyFinishContext();
  return __pyx_r;
}

static PyObject *__pyx_pf_10avalanches_6coherence_raster(CYTHON_UNUSED PyObject *__pyx_self, __Pyx_memviewslice __pyx_v_X, double __pyx_v_threshold, double __pyx_v_R) {
  int __pyx_v_N0;
  int __pyx_v_N1;
  __Pyx_memviewslice __pyx_v_sd = { 0, 0, { 0 }, { 0 }, { 0 } };
  __Pyx_memviewslice __pyx_v_means = { 0, 0, { 0 }, { 0 }, { 0 } };
  __Pyx_memviewslice __pyx_v_raster = { 0, 0, { 0 }, { 0 }, { 0 } };
  PyObject *__pyx_v_found_peaks = 0;
  int __pyx_v_idx;
  int __pyx_v_i;
  int __pyx_v_j;
  int __pyx_v_x;
  int __pyx_v_argmx;
  double __pyx_v_mx;
  __Pyx_memviewslice __pyx_v_sl_i = { 0, 0, { 0 }, { 0 }, { 0 } };
  __Pyx_memviewslice __pyx_v_sl_k = { 0, 0, { 0 }, { 0 }, { 0 } };
  Py_ssize_t __pyx_v_Nsl_i;
  int __pyx_v_k;
  Py_ssize_t __pyx_v_Nsl_k;
  PyObject *__pyx_r = NULL;
  __Pyx_RefNannyDeclarations
  __Pyx_RefNannySetupContext("coherence_raster", 0);
/* … */
  /* function exit code */
  __pyx_L1_error:;
  __Pyx_XDECREF(__pyx_t_1);
  __Pyx_XDECREF(__pyx_t_2);
  __Pyx_XDECREF(__pyx_t_3);
  __Pyx_XDECREF(__pyx_t_4);
  __PYX_XDEC_MEMVIEW(&__pyx_t_5, 1);
  __Pyx_XDECREF(__pyx_t_6);
  __PYX_XDEC_MEMVIEW(&__pyx_t_7, 1);
  __Pyx_XDECREF(__pyx_t_42);
  __Pyx_AddTraceback("avalanches.coherence_raster", __pyx_clineno, __pyx_lineno, __pyx_filename);
  __pyx_r = NULL;
  __pyx_L0:;
  __PYX_XDEC_MEMVIEW(&__pyx_v_sd, 1);
  __PYX_XDEC_MEMVIEW(&__pyx_v_means, 1);
  __PYX_XDEC_MEMVIEW(&__pyx_v_raster, 1);
  __Pyx_XDECREF(__pyx_v_found_peaks);
  __PYX_XDEC_MEMVIEW(&__pyx_v_sl_i, 1);
  __PYX_XDEC_MEMVIEW(&__pyx_v_sl_k, 1);
  __PYX_XDEC_MEMVIEW(&__pyx_v_X, 1);
  __Pyx_XGIVEREF(__pyx_r);
  __Pyx_RefNannyFinishContext();
  return __pyx_r;
}
/* … */
  __pyx_tuple__45 = PyTuple_Pack(20, __pyx_n_s_X, __pyx_n_s_threshold, __pyx_n_s_R, __pyx_n_s_N0, __pyx_n_s_N1, __pyx_n_s_sd, __pyx_n_s_means, __pyx_n_s_raster, __pyx_n_s_found_peaks, __pyx_n_s_idx, __pyx_n_s_i, __pyx_n_s_j, __pyx_n_s_x, __pyx_n_s_argmx, __pyx_n_s_mx, __pyx_n_s_sl_i, __pyx_n_s_sl_k, __pyx_n_s_Nsl_i, __pyx_n_s_k, __pyx_n_s_Nsl_k); if (unlikely(!__pyx_tuple__45)) __PYX_ERR(0, 160, __pyx_L1_error)
  __Pyx_GOTREF(__pyx_tuple__45);
  __Pyx_GIVEREF(__pyx_tuple__45);
/* … */
  __pyx_t_2 = PyCFunction_NewEx(&__pyx_mdef_10avalanches_7coherence_raster, NULL, __pyx_n_s_avalanches); if (unlikely(!__pyx_t_2)) __PYX_ERR(0, 160, __pyx_L1_error)
  __Pyx_GOTREF(__pyx_t_2);
  if (PyDict_SetItem(__pyx_d, __pyx_n_s_coherence_raster, __pyx_t_2) < 0) __PYX_ERR(0, 160, __pyx_L1_error)
  __Pyx_DECREF(__pyx_t_2); __pyx_t_2 = 0;
  __pyx_codeobj__46 = (PyObject*)__Pyx_PyCode_New(3, 0, 20, 0, CO_OPTIMIZED|CO_NEWLOCALS, __pyx_empty_bytes, __pyx_empty_tuple, __pyx_empty_tuple, __pyx_tuple__45, __pyx_empty_tuple, __pyx_empty_tuple, __pyx_kp_s_avalanches_pyx, __pyx_n_s_coherence_raster, 160, __pyx_empty_bytes); if (unlikely(!__pyx_codeobj__46)) __PYX_ERR(0, 160, __pyx_L1_error)
```

```
+161:     cdef int N0 = X.shape[0]
```

```
  __pyx_v_N0 = (__pyx_v_X.shape[0]);
```

```
+162:     cdef int N1 = X.shape[1]
```

```
  __pyx_v_N1 = (__pyx_v_X.shape[1]);
```

```
+163:     cdef double[:] sd = np.std(X, axis = 1)
```

```
  __pyx_t_1 = __Pyx_GetModuleGlobalName(__pyx_n_s_np); if (unlikely(!__pyx_t_1)) __PYX_ERR(0, 163, __pyx_L1_error)
  __Pyx_GOTREF(__pyx_t_1);
  __pyx_t_2 = __Pyx_PyObject_GetAttrStr(__pyx_t_1, __pyx_n_s_std); if (unlikely(!__pyx_t_2)) __PYX_ERR(0, 163, __pyx_L1_error)
  __Pyx_GOTREF(__pyx_t_2);
  __Pyx_DECREF(__pyx_t_1); __pyx_t_1 = 0;
  __pyx_t_1 = __pyx_memoryview_fromslice(__pyx_v_X, 2, (PyObject *(*)(char *)) __pyx_memview_get_double, (int (*)(char *, PyObject *)) __pyx_memview_set_double, 0);; if (unlikely(!__pyx_t_1)) __PYX_ERR(0, 163, __pyx_L1_error)
  __Pyx_GOTREF(__pyx_t_1);
  __pyx_t_3 = PyTuple_New(1); if (unlikely(!__pyx_t_3)) __PYX_ERR(0, 163, __pyx_L1_error)
  __Pyx_GOTREF(__pyx_t_3);
  __Pyx_GIVEREF(__pyx_t_1);
  PyTuple_SET_ITEM(__pyx_t_3, 0, __pyx_t_1);
  __pyx_t_1 = 0;
  __pyx_t_1 = __Pyx_PyDict_NewPresized(1); if (unlikely(!__pyx_t_1)) __PYX_ERR(0, 163, __pyx_L1_error)
  __Pyx_GOTREF(__pyx_t_1);
  if (PyDict_SetItem(__pyx_t_1, __pyx_n_s_axis, __pyx_int_1) < 0) __PYX_ERR(0, 163, __pyx_L1_error)
  __pyx_t_4 = __Pyx_PyObject_Call(__pyx_t_2, __pyx_t_3, __pyx_t_1); if (unlikely(!__pyx_t_4)) __PYX_ERR(0, 163, __pyx_L1_error)
  __Pyx_GOTREF(__pyx_t_4);
  __Pyx_DECREF(__pyx_t_2); __pyx_t_2 = 0;
  __Pyx_DECREF(__pyx_t_3); __pyx_t_3 = 0;
  __Pyx_DECREF(__pyx_t_1); __pyx_t_1 = 0;
  __pyx_t_5 = __Pyx_PyObject_to_MemoryviewSlice_ds_double(__pyx_t_4, PyBUF_WRITABLE); if (unlikely(!__pyx_t_5.memview)) __PYX_ERR(0, 163, __pyx_L1_error)
  __Pyx_DECREF(__pyx_t_4); __pyx_t_4 = 0;
  __pyx_v_sd = __pyx_t_5;
  __pyx_t_5.memview = NULL;
  __pyx_t_5.data = NULL;
```

```
+164:     cdef double[:] means = np.mean(X, axis = 1)
```

```
  __pyx_t_4 = __Pyx_GetModuleGlobalName(__pyx_n_s_np); if (unlikely(!__pyx_t_4)) __PYX_ERR(0, 164, __pyx_L1_error)
  __Pyx_GOTREF(__pyx_t_4);
  __pyx_t_1 = __Pyx_PyObject_GetAttrStr(__pyx_t_4, __pyx_n_s_mean); if (unlikely(!__pyx_t_1)) __PYX_ERR(0, 164, __pyx_L1_error)
  __Pyx_GOTREF(__pyx_t_1);
  __Pyx_DECREF(__pyx_t_4); __pyx_t_4 = 0;
  __pyx_t_4 = __pyx_memoryview_fromslice(__pyx_v_X, 2, (PyObject *(*)(char *)) __pyx_memview_get_double, (int (*)(char *, PyObject *)) __pyx_memview_set_double, 0);; if (unlikely(!__pyx_t_4)) __PYX_ERR(0, 164, __pyx_L1_error)
  __Pyx_GOTREF(__pyx_t_4);
  __pyx_t_3 = PyTuple_New(1); if (unlikely(!__pyx_t_3)) __PYX_ERR(0, 164, __pyx_L1_error)
  __Pyx_GOTREF(__pyx_t_3);
  __Pyx_GIVEREF(__pyx_t_4);
  PyTuple_SET_ITEM(__pyx_t_3, 0, __pyx_t_4);
  __pyx_t_4 = 0;
  __pyx_t_4 = __Pyx_PyDict_NewPresized(1); if (unlikely(!__pyx_t_4)) __PYX_ERR(0, 164, __pyx_L1_error)
  __Pyx_GOTREF(__pyx_t_4);
  if (PyDict_SetItem(__pyx_t_4, __pyx_n_s_axis, __pyx_int_1) < 0) __PYX_ERR(0, 164, __pyx_L1_error)
  __pyx_t_2 = __Pyx_PyObject_Call(__pyx_t_1, __pyx_t_3, __pyx_t_4); if (unlikely(!__pyx_t_2)) __PYX_ERR(0, 164, __pyx_L1_error)
  __Pyx_GOTREF(__pyx_t_2);
  __Pyx_DECREF(__pyx_t_1); __pyx_t_1 = 0;
  __Pyx_DECREF(__pyx_t_3); __pyx_t_3 = 0;
  __Pyx_DECREF(__pyx_t_4); __pyx_t_4 = 0;
  __pyx_t_5 = __Pyx_PyObject_to_MemoryviewSlice_ds_double(__pyx_t_2, PyBUF_WRITABLE); if (unlikely(!__pyx_t_5.memview)) __PYX_ERR(0, 164, __pyx_L1_error)
  __Pyx_DECREF(__pyx_t_2); __pyx_t_2 = 0;
  __pyx_v_means = __pyx_t_5;
  __pyx_t_5.memview = NULL;
  __pyx_t_5.data = NULL;
```

```
+165:     cdef double[:,:] raster = np.zeros((N0, N1))
```

```
  __pyx_t_4 = __Pyx_GetModuleGlobalName(__pyx_n_s_np); if (unlikely(!__pyx_t_4)) __PYX_ERR(0, 165, __pyx_L1_error)
  __Pyx_GOTREF(__pyx_t_4);
  __pyx_t_3 = __Pyx_PyObject_GetAttrStr(__pyx_t_4, __pyx_n_s_zeros); if (unlikely(!__pyx_t_3)) __PYX_ERR(0, 165, __pyx_L1_error)
  __Pyx_GOTREF(__pyx_t_3);
  __Pyx_DECREF(__pyx_t_4); __pyx_t_4 = 0;
  __pyx_t_4 = __Pyx_PyInt_From_int(__pyx_v_N0); if (unlikely(!__pyx_t_4)) __PYX_ERR(0, 165, __pyx_L1_error)
  __Pyx_GOTREF(__pyx_t_4);
  __pyx_t_1 = __Pyx_PyInt_From_int(__pyx_v_N1); if (unlikely(!__pyx_t_1)) __PYX_ERR(0, 165, __pyx_L1_error)
  __Pyx_GOTREF(__pyx_t_1);
  __pyx_t_6 = PyTuple_New(2); if (unlikely(!__pyx_t_6)) __PYX_ERR(0, 165, __pyx_L1_error)
  __Pyx_GOTREF(__pyx_t_6);
  __Pyx_GIVEREF(__pyx_t_4);
  PyTuple_SET_ITEM(__pyx_t_6, 0, __pyx_t_4);
  __Pyx_GIVEREF(__pyx_t_1);
  PyTuple_SET_ITEM(__pyx_t_6, 1, __pyx_t_1);
  __pyx_t_4 = 0;
  __pyx_t_1 = 0;
  __pyx_t_1 = NULL;
  if (CYTHON_UNPACK_METHODS && unlikely(PyMethod_Check(__pyx_t_3))) {
    __pyx_t_1 = PyMethod_GET_SELF(__pyx_t_3);
    if (likely(__pyx_t_1)) {
      PyObject* function = PyMethod_GET_FUNCTION(__pyx_t_3);
      __Pyx_INCREF(__pyx_t_1);
      __Pyx_INCREF(function);
      __Pyx_DECREF_SET(__pyx_t_3, function);
    }
  }
  if (!__pyx_t_1) {
    __pyx_t_2 = __Pyx_PyObject_CallOneArg(__pyx_t_3, __pyx_t_6); if (unlikely(!__pyx_t_2)) __PYX_ERR(0, 165, __pyx_L1_error)
    __Pyx_DECREF(__pyx_t_6); __pyx_t_6 = 0;
    __Pyx_GOTREF(__pyx_t_2);
  } else {
    #if CYTHON_FAST_PYCALL
    if (PyFunction_Check(__pyx_t_3)) {
      PyObject *__pyx_temp[2] = {__pyx_t_1, __pyx_t_6};
      __pyx_t_2 = __Pyx_PyFunction_FastCall(__pyx_t_3, __pyx_temp+1-1, 1+1); if (unlikely(!__pyx_t_2)) __PYX_ERR(0, 165, __pyx_L1_error)
      __Pyx_XDECREF(__pyx_t_1); __pyx_t_1 = 0;
      __Pyx_GOTREF(__pyx_t_2);
      __Pyx_DECREF(__pyx_t_6); __pyx_t_6 = 0;
    } else
    #endif
    #if CYTHON_FAST_PYCCALL
    if (__Pyx_PyFastCFunction_Check(__pyx_t_3)) {
      PyObject *__pyx_temp[2] = {__pyx_t_1, __pyx_t_6};
      __pyx_t_2 = __Pyx_PyCFunction_FastCall(__pyx_t_3, __pyx_temp+1-1, 1+1); if (unlikely(!__pyx_t_2)) __PYX_ERR(0, 165, __pyx_L1_error)
      __Pyx_XDECREF(__pyx_t_1); __pyx_t_1 = 0;
      __Pyx_GOTREF(__pyx_t_2);
      __Pyx_DECREF(__pyx_t_6); __pyx_t_6 = 0;
    } else
    #endif
    {
      __pyx_t_4 = PyTuple_New(1+1); if (unlikely(!__pyx_t_4)) __PYX_ERR(0, 165, __pyx_L1_error)
      __Pyx_GOTREF(__pyx_t_4);
      __Pyx_GIVEREF(__pyx_t_1); PyTuple_SET_ITEM(__pyx_t_4, 0, __pyx_t_1); __pyx_t_1 = NULL;
      __Pyx_GIVEREF(__pyx_t_6);
      PyTuple_SET_ITEM(__pyx_t_4, 0+1, __pyx_t_6);
      __pyx_t_6 = 0;
      __pyx_t_2 = __Pyx_PyObject_Call(__pyx_t_3, __pyx_t_4, NULL); if (unlikely(!__pyx_t_2)) __PYX_ERR(0, 165, __pyx_L1_error)
      __Pyx_GOTREF(__pyx_t_2);
      __Pyx_DECREF(__pyx_t_4); __pyx_t_4 = 0;
    }
  }
  __Pyx_DECREF(__pyx_t_3); __pyx_t_3 = 0;
  __pyx_t_7 = __Pyx_PyObject_to_MemoryviewSlice_dsds_double(__pyx_t_2, PyBUF_WRITABLE); if (unlikely(!__pyx_t_7.memview)) __PYX_ERR(0, 165, __pyx_L1_error)
  __Pyx_DECREF(__pyx_t_2); __pyx_t_2 = 0;
  __pyx_v_raster = __pyx_t_7;
  __pyx_t_7.memview = NULL;
  __pyx_t_7.data = NULL;
```

```
+166:     cdef set found_peaks = set()
```

```
  __pyx_t_2 = PySet_New(0); if (unlikely(!__pyx_t_2)) __PYX_ERR(0, 166, __pyx_L1_error)
  __Pyx_GOTREF(__pyx_t_2);
  __pyx_v_found_peaks = ((PyObject*)__pyx_t_2);
  __pyx_t_2 = 0;
```

```
 167:     cdef int idx, i, j, x, argmx
```

```
 168:     cdef double mx
```

```
 169:     cdef double[:] sl_i, sl_k
```

```
 170:     #cdef list ranges = []
```

```
 171:
```

```
 172:
```

```
+173:     for i in range(N0): #For each channel
```

```
  __pyx_t_8 = __pyx_v_N0;
  __pyx_t_9 = __pyx_t_8;
  for (__pyx_t_10 = 0; __pyx_t_10 < __pyx_t_9; __pyx_t_10+=1) {
    __pyx_v_i = __pyx_t_10;
```

```
+174:         idx = 0 #Begin at 0
```

```
    __pyx_v_idx = 0;
```

```
+175:         for j in range(N1-1):
```

```
    __pyx_t_11 = (__pyx_v_N1 - 1);
    __pyx_t_12 = __pyx_t_11;
    for (__pyx_t_13 = 0; __pyx_t_13 < __pyx_t_12; __pyx_t_13+=1) {
      __pyx_v_j = __pyx_t_13;
```

```
+176:             if (X[i][j] > means[i] and X[i][j+1] < means[i]) or (X[i][j] < means[i] and X[i][j+1] > means[i]): #Every time the trace crosses the mean
```

```
      __pyx_t_15 = __pyx_v_i;
      __pyx_t_16 = __pyx_v_j;
      __pyx_t_17 = __pyx_v_i;
      __pyx_t_18 = (((*((double *) ( /* dim=1 */ (( /* dim=0 */ (__pyx_v_X.data + __pyx_t_15 * __pyx_v_X.strides[0]) ) + __pyx_t_16 * __pyx_v_X.strides[1]) ))) > (*((double *) ( /* dim=0 */ (__pyx_v_means.data + __pyx_t_17 * __pyx_v_means.strides[0]) )))) != 0);
      if (!__pyx_t_18) {
        goto __pyx_L9_next_or;
      } else {
      }
      __pyx_t_19 = __pyx_v_i;
      __pyx_t_20 = (__pyx_v_j + 1);
      __pyx_t_21 = __pyx_v_i;
      __pyx_t_18 = (((*((double *) ( /* dim=1 */ (( /* dim=0 */ (__pyx_v_X.data + __pyx_t_19 * __pyx_v_X.strides[0]) ) + __pyx_t_20 * __pyx_v_X.strides[1]) ))) < (*((double *) ( /* dim=0 */ (__pyx_v_means.data + __pyx_t_21 * __pyx_v_means.strides[0]) )))) != 0);
      if (!__pyx_t_18) {
      } else {
        __pyx_t_14 = __pyx_t_18;
        goto __pyx_L8_bool_binop_done;
      }
      __pyx_L9_next_or:;
      __pyx_t_22 = __pyx_v_i;
      __pyx_t_23 = __pyx_v_j;
      __pyx_t_24 = __pyx_v_i;
      __pyx_t_18 = (((*((double *) ( /* dim=1 */ (( /* dim=0 */ (__pyx_v_X.data + __pyx_t_22 * __pyx_v_X.strides[0]) ) + __pyx_t_23 * __pyx_v_X.strides[1]) ))) < (*((double *) ( /* dim=0 */ (__pyx_v_means.data + __pyx_t_24 * __pyx_v_means.strides[0]) )))) != 0);
      if (__pyx_t_18) {
      } else {
        __pyx_t_14 = __pyx_t_18;
        goto __pyx_L8_bool_binop_done;
      }
      __pyx_t_25 = __pyx_v_i;
      __pyx_t_26 = (__pyx_v_j + 1);
      __pyx_t_27 = __pyx_v_i;
      __pyx_t_18 = (((*((double *) ( /* dim=1 */ (( /* dim=0 */ (__pyx_v_X.data + __pyx_t_25 * __pyx_v_X.strides[0]) ) + __pyx_t_26 * __pyx_v_X.strides[1]) ))) > (*((double *) ( /* dim=0 */ (__pyx_v_means.data + __pyx_t_27 * __pyx_v_means.strides[0]) )))) != 0);
      __pyx_t_14 = __pyx_t_18;
      __pyx_L8_bool_binop_done:;
      if (__pyx_t_14) {
/* … */
      }
    }
  }
```

```
+177:                 sl_i = X[i][idx:j] #Takes a slice from idx to wherever in the trace it has gotten to.
```

```
        __pyx_t_5.data = __pyx_v_X.data;
        __pyx_t_5.memview = __pyx_v_X.memview;
        __PYX_INC_MEMVIEW(&__pyx_t_5, 0);
        {
    Py_ssize_t __pyx_tmp_idx = __pyx_v_i;
    Py_ssize_t __pyx_tmp_shape = __pyx_v_X.shape[0];
    Py_ssize_t __pyx_tmp_stride = __pyx_v_X.strides[0];
    if (0 && (__pyx_tmp_idx < 0))
        __pyx_tmp_idx += __pyx_tmp_shape;
    if (0 && (__pyx_tmp_idx < 0 || __pyx_tmp_idx >= __pyx_tmp_shape)) {
        PyErr_SetString(PyExc_IndexError, "Index out of bounds (axis 0)");
        __PYX_ERR(0, 177, __pyx_L1_error)
    }
        __pyx_t_5.data += __pyx_tmp_idx * __pyx_tmp_stride;
}

__pyx_t_28 = -1;
        if (unlikely(__pyx_memoryview_slice_memviewslice(
    &__pyx_t_5,
    __pyx_v_X.shape[1], __pyx_v_X.strides[1], __pyx_v_X.suboffsets[1],
    1,
    0,
    &__pyx_t_28,
    __pyx_v_idx,
    __pyx_v_j,
    0,
    1,
    1,
    0,
    1) < 0))
{
    __PYX_ERR(0, 177, __pyx_L1_error)
}

__PYX_XDEC_MEMVIEW(&__pyx_v_sl_i, 1);
        __pyx_v_sl_i = __pyx_t_5;
        __pyx_t_5.memview = NULL;
        __pyx_t_5.data = NULL;
```

```
 178:                 #ranges.append((idx, j, sl_i))
```

```
 179:
```

```
 180:                 #Finding the max and argmax of the slice
```

```
+181:                 mx, argmx = 0, 0
```

```
        __pyx_t_29 = 0.0;
        __pyx_t_28 = 0;
        __pyx_v_mx = __pyx_t_29;
        __pyx_v_argmx = __pyx_t_28;
```

```
+182:                 Nsl_i = sl_i.shape[0]
```

```
        __pyx_v_Nsl_i = (__pyx_v_sl_i.shape[0]);
```

```
+183:                 for x in range(Nsl_i):
```

```
        __pyx_t_30 = __pyx_v_Nsl_i;
        __pyx_t_31 = __pyx_t_30;
        for (__pyx_t_28 = 0; __pyx_t_28 < __pyx_t_31; __pyx_t_28+=1) {
          __pyx_v_x = __pyx_t_28;
```

```
+184:                     if abs(sl_i[x]) > mx:
```

```
          __pyx_t_32 = __pyx_v_x;
          __pyx_t_14 = ((fabs((*((double *) ( /* dim=0 */ (__pyx_v_sl_i.data + __pyx_t_32 * __pyx_v_sl_i.strides[0]) )))) > __pyx_v_mx) != 0);
          if (__pyx_t_14) {
/* … */
          }
        }
```

```
+185:                         mx = abs(sl_i[x])
```

```
            __pyx_t_33 = __pyx_v_x;
            __pyx_v_mx = fabs((*((double *) ( /* dim=0 */ (__pyx_v_sl_i.data + __pyx_t_33 * __pyx_v_sl_i.strides[0]) ))));
```

```
+186:                         argmx = x
```

```
            __pyx_v_argmx = __pyx_v_x;
```

```
 187:
```

```
+188:                 if mx >= means[i] + (threshold * sd[i]): #If the max of the slice is greater than the threshold
```

```
        __pyx_t_34 = __pyx_v_i;
        __pyx_t_35 = __pyx_v_i;
        __pyx_t_14 = ((__pyx_v_mx >= ((*((double *) ( /* dim=0 */ (__pyx_v_means.data + __pyx_t_34 * __pyx_v_means.strides[0]) ))) + (__pyx_v_threshold * (*((double *) ( /* dim=0 */ (__pyx_v_sd.data + __pyx_t_35 * __pyx_v_sd.strides[0]) )))))) != 0);
        if (__pyx_t_14) {
/* … */
        }
```

```
+189:                     if (i, idx + argmx) not in found_peaks: #If that peak has not been previously found
```

```
          __pyx_t_2 = __Pyx_PyInt_From_int(__pyx_v_i); if (unlikely(!__pyx_t_2)) __PYX_ERR(0, 189, __pyx_L1_error)
          __Pyx_GOTREF(__pyx_t_2);
          __pyx_t_3 = __Pyx_PyInt_From_int((__pyx_v_idx + __pyx_v_argmx)); if (unlikely(!__pyx_t_3)) __PYX_ERR(0, 189, __pyx_L1_error)
          __Pyx_GOTREF(__pyx_t_3);
          __pyx_t_4 = PyTuple_New(2); if (unlikely(!__pyx_t_4)) __PYX_ERR(0, 189, __pyx_L1_error)
          __Pyx_GOTREF(__pyx_t_4);
          __Pyx_GIVEREF(__pyx_t_2);
          PyTuple_SET_ITEM(__pyx_t_4, 0, __pyx_t_2);
          __Pyx_GIVEREF(__pyx_t_3);
          PyTuple_SET_ITEM(__pyx_t_4, 1, __pyx_t_3);
          __pyx_t_2 = 0;
          __pyx_t_3 = 0;
          __pyx_t_14 = (__Pyx_PySet_ContainsTF(__pyx_t_4, __pyx_v_found_peaks, Py_NE)); if (unlikely(__pyx_t_14 < 0)) __PYX_ERR(0, 189, __pyx_L1_error)
          __Pyx_DECREF(__pyx_t_4); __pyx_t_4 = 0;
          __pyx_t_18 = (__pyx_t_14 != 0);
          if (__pyx_t_18) {
/* … */
          }
```

```
+190:                         raster[i][idx + argmx] = 1
```

```
            __pyx_t_36 = __pyx_v_i;
            __pyx_t_37 = (__pyx_v_idx + __pyx_v_argmx);
            *((double *) ( /* dim=1 */ (( /* dim=0 */ (__pyx_v_raster.data + __pyx_t_36 * __pyx_v_raster.strides[0]) ) + __pyx_t_37 * __pyx_v_raster.strides[1]) )) = 1.0;
```

```
+191:                         found_peaks.add((i, idx + argmx))
```

```
            __pyx_t_4 = __Pyx_PyInt_From_int(__pyx_v_i); if (unlikely(!__pyx_t_4)) __PYX_ERR(0, 191, __pyx_L1_error)
            __Pyx_GOTREF(__pyx_t_4);
            __pyx_t_3 = __Pyx_PyInt_From_int((__pyx_v_idx + __pyx_v_argmx)); if (unlikely(!__pyx_t_3)) __PYX_ERR(0, 191, __pyx_L1_error)
            __Pyx_GOTREF(__pyx_t_3);
            __pyx_t_2 = PyTuple_New(2); if (unlikely(!__pyx_t_2)) __PYX_ERR(0, 191, __pyx_L1_error)
            __Pyx_GOTREF(__pyx_t_2);
            __Pyx_GIVEREF(__pyx_t_4);
            PyTuple_SET_ITEM(__pyx_t_2, 0, __pyx_t_4);
            __Pyx_GIVEREF(__pyx_t_3);
            PyTuple_SET_ITEM(__pyx_t_2, 1, __pyx_t_3);
            __pyx_t_4 = 0;
            __pyx_t_3 = 0;
            __pyx_t_38 = PySet_Add(__pyx_v_found_peaks, __pyx_t_2); if (unlikely(__pyx_t_38 == ((int)-1))) __PYX_ERR(0, 191, __pyx_L1_error)
            __Pyx_DECREF(__pyx_t_2); __pyx_t_2 = 0;
```

```
 192:
```

```
+193:                     for k in range(N0):
```

```
          __pyx_t_28 = __pyx_v_N0;
          __pyx_t_39 = __pyx_t_28;
          for (__pyx_t_40 = 0; __pyx_t_40 < __pyx_t_39; __pyx_t_40+=1) {
            __pyx_v_k = __pyx_t_40;
```

```
+194:                         if k != i:
```

```
            __pyx_t_18 = ((__pyx_v_k != __pyx_v_i) != 0);
            if (__pyx_t_18) {
/* … */
            }
          }
```

```
+195:                             sl_k = X[k][idx:j]
```

```
              __pyx_t_5.data = __pyx_v_X.data;
              __pyx_t_5.memview = __pyx_v_X.memview;
              __PYX_INC_MEMVIEW(&__pyx_t_5, 0);
              {
    Py_ssize_t __pyx_tmp_idx = __pyx_v_k;
    Py_ssize_t __pyx_tmp_shape = __pyx_v_X.shape[0];
    Py_ssize_t __pyx_tmp_stride = __pyx_v_X.strides[0];
    if (0 && (__pyx_tmp_idx < 0))
        __pyx_tmp_idx += __pyx_tmp_shape;
    if (0 && (__pyx_tmp_idx < 0 || __pyx_tmp_idx >= __pyx_tmp_shape)) {
        PyErr_SetString(PyExc_IndexError, "Index out of bounds (axis 0)");
        __PYX_ERR(0, 195, __pyx_L1_error)
    }
        __pyx_t_5.data += __pyx_tmp_idx * __pyx_tmp_stride;
}

__pyx_t_41 = -1;
              if (unlikely(__pyx_memoryview_slice_memviewslice(
    &__pyx_t_5,
    __pyx_v_X.shape[1], __pyx_v_X.strides[1], __pyx_v_X.suboffsets[1],
    1,
    0,
    &__pyx_t_41,
    __pyx_v_idx,
    __pyx_v_j,
    0,
    1,
    1,
    0,
    1) < 0))
{
    __PYX_ERR(0, 195, __pyx_L1_error)
}

__PYX_XDEC_MEMVIEW(&__pyx_v_sl_k, 1);
              __pyx_v_sl_k = __pyx_t_5;
              __pyx_t_5.memview = NULL;
              __pyx_t_5.data = NULL;
```

```
 196:
```

```
+197:                             if pearsonr(sl_i, sl_k)[0] > R:
```

```
              __pyx_t_3 = __Pyx_GetModuleGlobalName(__pyx_n_s_pearsonr); if (unlikely(!__pyx_t_3)) __PYX_ERR(0, 197, __pyx_L1_error)
              __Pyx_GOTREF(__pyx_t_3);
              __pyx_t_4 = __pyx_memoryview_fromslice(__pyx_v_sl_i, 1, (PyObject *(*)(char *)) __pyx_memview_get_double, (int (*)(char *, PyObject *)) __pyx_memview_set_double, 0);; if (unlikely(!__pyx_t_4)) __PYX_ERR(0, 197, __pyx_L1_error)
              __Pyx_GOTREF(__pyx_t_4);
              __pyx_t_6 = __pyx_memoryview_fromslice(__pyx_v_sl_k, 1, (PyObject *(*)(char *)) __pyx_memview_get_double, (int (*)(char *, PyObject *)) __pyx_memview_set_double, 0);; if (unlikely(!__pyx_t_6)) __PYX_ERR(0, 197, __pyx_L1_error)
              __Pyx_GOTREF(__pyx_t_6);
              __pyx_t_1 = NULL;
              __pyx_t_41 = 0;
              if (CYTHON_UNPACK_METHODS && unlikely(PyMethod_Check(__pyx_t_3))) {
                __pyx_t_1 = PyMethod_GET_SELF(__pyx_t_3);
                if (likely(__pyx_t_1)) {
                  PyObject* function = PyMethod_GET_FUNCTION(__pyx_t_3);
                  __Pyx_INCREF(__pyx_t_1);
                  __Pyx_INCREF(function);
                  __Pyx_DECREF_SET(__pyx_t_3, function);
                  __pyx_t_41 = 1;
                }
              }
              #if CYTHON_FAST_PYCALL
              if (PyFunction_Check(__pyx_t_3)) {
                PyObject *__pyx_temp[3] = {__pyx_t_1, __pyx_t_4, __pyx_t_6};
                __pyx_t_2 = __Pyx_PyFunction_FastCall(__pyx_t_3, __pyx_temp+1-__pyx_t_41, 2+__pyx_t_41); if (unlikely(!__pyx_t_2)) __PYX_ERR(0, 197, __pyx_L1_error)
                __Pyx_XDECREF(__pyx_t_1); __pyx_t_1 = 0;
                __Pyx_GOTREF(__pyx_t_2);
                __Pyx_DECREF(__pyx_t_4); __pyx_t_4 = 0;
                __Pyx_DECREF(__pyx_t_6); __pyx_t_6 = 0;
              } else
              #endif
              #if CYTHON_FAST_PYCCALL
              if (__Pyx_PyFastCFunction_Check(__pyx_t_3)) {
                PyObject *__pyx_temp[3] = {__pyx_t_1, __pyx_t_4, __pyx_t_6};
                __pyx_t_2 = __Pyx_PyCFunction_FastCall(__pyx_t_3, __pyx_temp+1-__pyx_t_41, 2+__pyx_t_41); if (unlikely(!__pyx_t_2)) __PYX_ERR(0, 197, __pyx_L1_error)
                __Pyx_XDECREF(__pyx_t_1); __pyx_t_1 = 0;
                __Pyx_GOTREF(__pyx_t_2);
                __Pyx_DECREF(__pyx_t_4); __pyx_t_4 = 0;
                __Pyx_DECREF(__pyx_t_6); __pyx_t_6 = 0;
              } else
              #endif
              {
                __pyx_t_42 = PyTuple_New(2+__pyx_t_41); if (unlikely(!__pyx_t_42)) __PYX_ERR(0, 197, __pyx_L1_error)
                __Pyx_GOTREF(__pyx_t_42);
                if (__pyx_t_1) {
                  __Pyx_GIVEREF(__pyx_t_1); PyTuple_SET_ITEM(__pyx_t_42, 0, __pyx_t_1); __pyx_t_1 = NULL;
                }
                __Pyx_GIVEREF(__pyx_t_4);
                PyTuple_SET_ITEM(__pyx_t_42, 0+__pyx_t_41, __pyx_t_4);
                __Pyx_GIVEREF(__pyx_t_6);
                PyTuple_SET_ITEM(__pyx_t_42, 1+__pyx_t_41, __pyx_t_6);
                __pyx_t_4 = 0;
                __pyx_t_6 = 0;
                __pyx_t_2 = __Pyx_PyObject_Call(__pyx_t_3, __pyx_t_42, NULL); if (unlikely(!__pyx_t_2)) __PYX_ERR(0, 197, __pyx_L1_error)
                __Pyx_GOTREF(__pyx_t_2);
                __Pyx_DECREF(__pyx_t_42); __pyx_t_42 = 0;
              }
              __Pyx_DECREF(__pyx_t_3); __pyx_t_3 = 0;
              __pyx_t_3 = __Pyx_GetItemInt(__pyx_t_2, 0, long, 1, __Pyx_PyInt_From_long, 0, 0, 0); if (unlikely(!__pyx_t_3)) __PYX_ERR(0, 197, __pyx_L1_error)
              __Pyx_GOTREF(__pyx_t_3);
              __Pyx_DECREF(__pyx_t_2); __pyx_t_2 = 0;
              __pyx_t_2 = PyFloat_FromDouble(__pyx_v_R); if (unlikely(!__pyx_t_2)) __PYX_ERR(0, 197, __pyx_L1_error)
              __Pyx_GOTREF(__pyx_t_2);
              __pyx_t_42 = PyObject_RichCompare(__pyx_t_3, __pyx_t_2, Py_GT); __Pyx_XGOTREF(__pyx_t_42); if (unlikely(!__pyx_t_42)) __PYX_ERR(0, 197, __pyx_L1_error)
              __Pyx_DECREF(__pyx_t_3); __pyx_t_3 = 0;
              __Pyx_DECREF(__pyx_t_2); __pyx_t_2 = 0;
              __pyx_t_18 = __Pyx_PyObject_IsTrue(__pyx_t_42); if (unlikely(__pyx_t_18 < 0)) __PYX_ERR(0, 197, __pyx_L1_error)
              __Pyx_DECREF(__pyx_t_42); __pyx_t_42 = 0;
              if (__pyx_t_18) {
/* … */
              }
```

```
+198:                                 Nsl_k = sl_k.shape[0]
```

```
                __pyx_v_Nsl_k = (__pyx_v_sl_k.shape[0]);
```

```
+199:                                 mx, argmx = 0, 0
```

```
                __pyx_t_29 = 0.0;
                __pyx_t_41 = 0;
                __pyx_v_mx = __pyx_t_29;
                __pyx_v_argmx = __pyx_t_41;
```

```
+200:                                 for x in range(Nsl_k):
```

```
                __pyx_t_30 = __pyx_v_Nsl_k;
                __pyx_t_31 = __pyx_t_30;
                for (__pyx_t_41 = 0; __pyx_t_41 < __pyx_t_31; __pyx_t_41+=1) {
                  __pyx_v_x = __pyx_t_41;
```

```
+201:                                     if abs(sl_k[x]) > mx:
```

```
                  __pyx_t_43 = __pyx_v_x;
                  __pyx_t_18 = ((fabs((*((double *) ( /* dim=0 */ (__pyx_v_sl_k.data + __pyx_t_43 * __pyx_v_sl_k.strides[0]) )))) > __pyx_v_mx) != 0);
                  if (__pyx_t_18) {
/* … */
                  }
                }
```

```
+202:                                         mx = abs(sl_k[x])
```

```
                    __pyx_t_44 = __pyx_v_x;
                    __pyx_v_mx = fabs((*((double *) ( /* dim=0 */ (__pyx_v_sl_k.data + __pyx_t_44 * __pyx_v_sl_k.strides[0]) ))));
```

```
+203:                                         argmx = x
```

```
                    __pyx_v_argmx = __pyx_v_x;
```

```
 204:
```

```
+205:                                 if (k, idx + argmx) not in found_peaks:
```

```
                __pyx_t_42 = __Pyx_PyInt_From_int(__pyx_v_k); if (unlikely(!__pyx_t_42)) __PYX_ERR(0, 205, __pyx_L1_error)
                __Pyx_GOTREF(__pyx_t_42);
                __pyx_t_2 = __Pyx_PyInt_From_int((__pyx_v_idx + __pyx_v_argmx)); if (unlikely(!__pyx_t_2)) __PYX_ERR(0, 205, __pyx_L1_error)
                __Pyx_GOTREF(__pyx_t_2);
                __pyx_t_3 = PyTuple_New(2); if (unlikely(!__pyx_t_3)) __PYX_ERR(0, 205, __pyx_L1_error)
                __Pyx_GOTREF(__pyx_t_3);
                __Pyx_GIVEREF(__pyx_t_42);
                PyTuple_SET_ITEM(__pyx_t_3, 0, __pyx_t_42);
                __Pyx_GIVEREF(__pyx_t_2);
                PyTuple_SET_ITEM(__pyx_t_3, 1, __pyx_t_2);
                __pyx_t_42 = 0;
                __pyx_t_2 = 0;
                __pyx_t_18 = (__Pyx_PySet_ContainsTF(__pyx_t_3, __pyx_v_found_peaks, Py_NE)); if (unlikely(__pyx_t_18 < 0)) __PYX_ERR(0, 205, __pyx_L1_error)
                __Pyx_DECREF(__pyx_t_3); __pyx_t_3 = 0;
                __pyx_t_14 = (__pyx_t_18 != 0);
                if (__pyx_t_14) {
/* … */
                }
```

```
+206:                                     raster[k][idx + argmx] = 1
```

```
                  __pyx_t_45 = __pyx_v_k;
                  __pyx_t_46 = (__pyx_v_idx + __pyx_v_argmx);
                  *((double *) ( /* dim=1 */ (( /* dim=0 */ (__pyx_v_raster.data + __pyx_t_45 * __pyx_v_raster.strides[0]) ) + __pyx_t_46 * __pyx_v_raster.strides[1]) )) = 1.0;
```

```
+207:                                     found_peaks.add((k, idx + argmx))
```

```
                  __pyx_t_3 = __Pyx_PyInt_From_int(__pyx_v_k); if (unlikely(!__pyx_t_3)) __PYX_ERR(0, 207, __pyx_L1_error)
                  __Pyx_GOTREF(__pyx_t_3);
                  __pyx_t_2 = __Pyx_PyInt_From_int((__pyx_v_idx + __pyx_v_argmx)); if (unlikely(!__pyx_t_2)) __PYX_ERR(0, 207, __pyx_L1_error)
                  __Pyx_GOTREF(__pyx_t_2);
                  __pyx_t_42 = PyTuple_New(2); if (unlikely(!__pyx_t_42)) __PYX_ERR(0, 207, __pyx_L1_error)
                  __Pyx_GOTREF(__pyx_t_42);
                  __Pyx_GIVEREF(__pyx_t_3);
                  PyTuple_SET_ITEM(__pyx_t_42, 0, __pyx_t_3);
                  __Pyx_GIVEREF(__pyx_t_2);
                  PyTuple_SET_ITEM(__pyx_t_42, 1, __pyx_t_2);
                  __pyx_t_3 = 0;
                  __pyx_t_2 = 0;
                  __pyx_t_38 = PySet_Add(__pyx_v_found_peaks, __pyx_t_42); if (unlikely(__pyx_t_38 == ((int)-1))) __PYX_ERR(0, 207, __pyx_L1_error)
                  __Pyx_DECREF(__pyx_t_42); __pyx_t_42 = 0;
```

```
 208:
```

```
+209:                 idx = j
```

```
        __pyx_v_idx = __pyx_v_j;
```

```
 210:
```

```
+211:     return raster
```

```
  __Pyx_XDECREF(__pyx_r);
  __pyx_t_42 = __pyx_memoryview_fromslice(__pyx_v_raster, 2, (PyObject *(*)(char *)) __pyx_memview_get_double, (int (*)(char *, PyObject *)) __pyx_memview_set_double, 0);; if (unlikely(!__pyx_t_42)) __PYX_ERR(0, 211, __pyx_L1_error)
  __Pyx_GOTREF(__pyx_t_42);
  __pyx_r = __pyx_t_42;
  __pyx_t_42 = 0;
  goto __pyx_L0;
```

```
 212:
```

```
 213:
```

```
 214: @cython.wraparound(False)
```

```
 215: @cython.boundscheck(False)
```

```
 216: @cython.cdivision(True)
```

```
+217: def avalanches(int[:,:] X, int bin_size, int min_length = 0, int min_frequency = 1):
```

```
/* Python wrapper */
static PyObject *__pyx_pw_10avalanches_9avalanches(PyObject *__pyx_self, PyObject *__pyx_args, PyObject *__pyx_kwds); /*proto*/
static char __pyx_doc_10avalanches_8avalanches[] = "\n    For a raster of data, returns the size and length of each avalanche, and how many times each channel participates in avalanches.\n    \n    Arguments:\n        X:\n            A 2-dimensional Numpy array (dtype must be \"int32\")\n        bin_size:\n            The size of the bin. \n    \n    Returns:\n        Avalanche_sizes: \n            The number of channels that participates in a given avalanche.\n        Avalanche_lengths:\n            The number of bins each avalanche persists for.\n        Channel_avalanches:\n            The number of times each channel participates in an avalanche. \n    \n    To do:\n        Get the size of an avalanche over time, for shape-collapse. \n    ";
static PyMethodDef __pyx_mdef_10avalanches_9avalanches = {"avalanches", (PyCFunction)__pyx_pw_10avalanches_9avalanches, METH_VARARGS|METH_KEYWORDS, __pyx_doc_10avalanches_8avalanches};
static PyObject *__pyx_pw_10avalanches_9avalanches(PyObject *__pyx_self, PyObject *__pyx_args, PyObject *__pyx_kwds) {
  __Pyx_memviewslice __pyx_v_X = { 0, 0, { 0 }, { 0 }, { 0 } };
  int __pyx_v_bin_size;
  int __pyx_v_min_length;
  int __pyx_v_min_frequency;
  PyObject *__pyx_r = 0;
  __Pyx_RefNannyDeclarations
  __Pyx_RefNannySetupContext("avalanches (wrapper)", 0);
  {
    static PyObject **__pyx_pyargnames[] = {&__pyx_n_s_X,&__pyx_n_s_bin_size,&__pyx_n_s_min_length,&__pyx_n_s_min_frequency,0};
    PyObject* values[4] = {0,0,0,0};
    if (unlikely(__pyx_kwds)) {
      Py_ssize_t kw_args;
      const Py_ssize_t pos_args = PyTuple_GET_SIZE(__pyx_args);
      switch (pos_args) {
        case  4: values[3] = PyTuple_GET_ITEM(__pyx_args, 3);
        CYTHON_FALLTHROUGH;
        case  3: values[2] = PyTuple_GET_ITEM(__pyx_args, 2);
        CYTHON_FALLTHROUGH;
        case  2: values[1] = PyTuple_GET_ITEM(__pyx_args, 1);
        CYTHON_FALLTHROUGH;
        case  1: values[0] = PyTuple_GET_ITEM(__pyx_args, 0);
        CYTHON_FALLTHROUGH;
        case  0: break;
        default: goto __pyx_L5_argtuple_error;
      }
      kw_args = PyDict_Size(__pyx_kwds);
      switch (pos_args) {
        case  0:
        if (likely((values[0] = __Pyx_PyDict_GetItemStr(__pyx_kwds, __pyx_n_s_X)) != 0)) kw_args--;
        else goto __pyx_L5_argtuple_error;
        CYTHON_FALLTHROUGH;
        case  1:
        if (likely((values[1] = __Pyx_PyDict_GetItemStr(__pyx_kwds, __pyx_n_s_bin_size)) != 0)) kw_args--;
        else {
          __Pyx_RaiseArgtupleInvalid("avalanches", 0, 2, 4, 1); __PYX_ERR(0, 217, __pyx_L3_error)
        }
        CYTHON_FALLTHROUGH;
        case  2:
        if (kw_args > 0) {
          PyObject* value = __Pyx_PyDict_GetItemStr(__pyx_kwds, __pyx_n_s_min_length);
          if (value) { values[2] = value; kw_args--; }
        }
        CYTHON_FALLTHROUGH;
        case  3:
        if (kw_args > 0) {
          PyObject* value = __Pyx_PyDict_GetItemStr(__pyx_kwds, __pyx_n_s_min_frequency);
          if (value) { values[3] = value; kw_args--; }
        }
      }
      if (unlikely(kw_args > 0)) {
        if (unlikely(__Pyx_ParseOptionalKeywords(__pyx_kwds, __pyx_pyargnames, 0, values, pos_args, "avalanches") < 0)) __PYX_ERR(0, 217, __pyx_L3_error)
      }
    } else {
      switch (PyTuple_GET_SIZE(__pyx_args)) {
        case  4: values[3] = PyTuple_GET_ITEM(__pyx_args, 3);
        CYTHON_FALLTHROUGH;
        case  3: values[2] = PyTuple_GET_ITEM(__pyx_args, 2);
        CYTHON_FALLTHROUGH;
        case  2: values[1] = PyTuple_GET_ITEM(__pyx_args, 1);
        values[0] = PyTuple_GET_ITEM(__pyx_args, 0);
        break;
        default: goto __pyx_L5_argtuple_error;
      }
    }
    __pyx_v_X = __Pyx_PyObject_to_MemoryviewSlice_dsds_int(values[0], PyBUF_WRITABLE); if (unlikely(!__pyx_v_X.memview)) __PYX_ERR(0, 217, __pyx_L3_error)
    __pyx_v_bin_size = __Pyx_PyInt_As_int(values[1]); if (unlikely((__pyx_v_bin_size == (int)-1) && PyErr_Occurred())) __PYX_ERR(0, 217, __pyx_L3_error)
    if (values[2]) {
      __pyx_v_min_length = __Pyx_PyInt_As_int(values[2]); if (unlikely((__pyx_v_min_length == (int)-1) && PyErr_Occurred())) __PYX_ERR(0, 217, __pyx_L3_error)
    } else {
      __pyx_v_min_length = ((int)0);
    }
    if (values[3]) {
      __pyx_v_min_frequency = __Pyx_PyInt_As_int(values[3]); if (unlikely((__pyx_v_min_frequency == (int)-1) && PyErr_Occurred())) __PYX_ERR(0, 217, __pyx_L3_error)
    } else {
      __pyx_v_min_frequency = ((int)1);
    }
  }
  goto __pyx_L4_argument_unpacking_done;
  __pyx_L5_argtuple_error:;
  __Pyx_RaiseArgtupleInvalid("avalanches", 0, 2, 4, PyTuple_GET_SIZE(__pyx_args)); __PYX_ERR(0, 217, __pyx_L3_error)
  __pyx_L3_error:;
  __Pyx_AddTraceback("avalanches.avalanches", __pyx_clineno, __pyx_lineno, __pyx_filename);
  __Pyx_RefNannyFinishContext();
  return NULL;
  __pyx_L4_argument_unpacking_done:;
  __pyx_r = __pyx_pf_10avalanches_8avalanches(__pyx_self, __pyx_v_X, __pyx_v_bin_size, __pyx_v_min_length, __pyx_v_min_frequency);

  /* function exit code */
  __Pyx_RefNannyFinishContext();
  return __pyx_r;
}

static PyObject *__pyx_pf_10avalanches_8avalanches(CYTHON_UNUSED PyObject *__pyx_self, __Pyx_memviewslice __pyx_v_X, int __pyx_v_bin_size, int __pyx_v_min_length, int __pyx_v_min_frequency) {
  int __pyx_v_N0;
  int __pyx_v_N1;
  int __pyx_v_num_bins;
  __Pyx_memviewslice __pyx_v_bins = { 0, 0, { 0 }, { 0 }, { 0 } };
  __Pyx_memviewslice __pyx_v_bin_events = { 0, 0, { 0 }, { 0 }, { 0 } };
  int __pyx_v_on;
  PyObject *__pyx_v_starts = 0;
  PyObject *__pyx_v_stops = 0;
  int __pyx_v_i;
  __Pyx_memviewslice __pyx_v_window = { 0, 0, { 0 }, { 0 }, { 0 } };
  __Pyx_memviewslice __pyx_v_Z = { 0, 0, { 0 }, { 0 }, { 0 } };
  PyObject *__pyx_v_Z_where_list = 0;
  __Pyx_memviewslice __pyx_v_Z_where = { 0, 0, { 0 }, { 0 }, { 0 } };
  int __pyx_v_Z_len;
  __Pyx_memviewslice __pyx_v_avalanche_lengths = { 0, 0, { 0 }, { 0 }, { 0 } };
  __Pyx_memviewslice __pyx_v_avalanche_array = { 0, 0, { 0 }, { 0 }, { 0 } };
  __Pyx_memviewslice __pyx_v_avalanche_shapes = { 0, 0, { 0 }, { 0 }, { 0 } };
  int __pyx_v_tup0;
  int __pyx_v_tup1;
  int __pyx_v_N0_avalanche;
  __Pyx_memviewslice __pyx_v_avalanche_sizes = { 0, 0, { 0 }, { 0 }, { 0 } };
  __Pyx_memviewslice __pyx_v_a = { 0, 0, { 0 }, { 0 }, { 0 } };
  int __pyx_v_a0;
  int __pyx_v_j;
  int __pyx_v_size;
  PyObject *__pyx_v_C_lengths = NULL;
  PyObject *__pyx_v_C_sizes = NULL;
  PyObject *__pyx_v_index_list = 0;
  __Pyx_memviewslice __pyx_v_avalanche_lengths_filtered = { 0, 0, { 0 }, { 0 }, { 0 } };
  __Pyx_memviewslice __pyx_v_avalanche_sizes_filtered = { 0, 0, { 0 }, { 0 }, { 0 } };
  __Pyx_memviewslice __pyx_v_avalanche_shapes_filtered = { 0, 0, { 0 }, { 0 }, { 0 } };
  CYTHON_UNUSED __Pyx_memviewslice __pyx_v_avalanche_array_filtered = { 0, 0, { 0 }, { 0 }, { 0 } };
  PyObject *__pyx_v_channel_avalanches = 0;
  PyObject *__pyx_r = NULL;
  __Pyx_RefNannyDeclarations
  __Pyx_RefNannySetupContext("avalanches", 0);
/* … */
  /* function exit code */
  __pyx_L1_error:;
  __Pyx_XDECREF(__pyx_t_1);
  __Pyx_XDECREF(__pyx_t_2);
  __Pyx_XDECREF(__pyx_t_3);
  __Pyx_XDECREF(__pyx_t_4);
  __PYX_XDEC_MEMVIEW(&__pyx_t_5, 1);
  __PYX_XDEC_MEMVIEW(&__pyx_t_9, 1);
  __PYX_XDEC_MEMVIEW(&__pyx_t_26, 1);
  __Pyx_XDECREF(__pyx_t_36);
  __Pyx_AddTraceback("avalanches.avalanches", __pyx_clineno, __pyx_lineno, __pyx_filename);
  __pyx_r = NULL;
  __pyx_L0:;
  __PYX_XDEC_MEMVIEW(&__pyx_v_bins, 1);
  __PYX_XDEC_MEMVIEW(&__pyx_v_bin_events, 1);
  __Pyx_XDECREF(__pyx_v_starts);
  __Pyx_XDECREF(__pyx_v_stops);
  __PYX_XDEC_MEMVIEW(&__pyx_v_window, 1);
  __PYX_XDEC_MEMVIEW(&__pyx_v_Z, 1);
  __Pyx_XDECREF(__pyx_v_Z_where_list);
  __PYX_XDEC_MEMVIEW(&__pyx_v_Z_where, 1);
  __PYX_XDEC_MEMVIEW(&__pyx_v_avalanche_lengths, 1);
  __PYX_XDEC_MEMVIEW(&__pyx_v_avalanche_array, 1);
  __PYX_XDEC_MEMVIEW(&__pyx_v_avalanche_shapes, 1);
  __PYX_XDEC_MEMVIEW(&__pyx_v_avalanche_sizes, 1);
  __PYX_XDEC_MEMVIEW(&__pyx_v_a, 1);
  __Pyx_XDECREF(__pyx_v_C_lengths);
  __Pyx_XDECREF(__pyx_v_C_sizes);
  __Pyx_XDECREF(__pyx_v_index_list);
  __PYX_XDEC_MEMVIEW(&__pyx_v_avalanche_lengths_filtered, 1);
  __PYX_XDEC_MEMVIEW(&__pyx_v_avalanche_sizes_filtered, 1);
  __PYX_XDEC_MEMVIEW(&__pyx_v_avalanche_shapes_filtered, 1);
  __PYX_XDEC_MEMVIEW(&__pyx_v_avalanche_array_filtered, 1);
  __Pyx_XDECREF(__pyx_v_channel_avalanches);
  __PYX_XDEC_MEMVIEW(&__pyx_v_X, 1);
  __Pyx_XGIVEREF(__pyx_r);
  __Pyx_RefNannyFinishContext();
  return __pyx_r;
}
/* … */
  __pyx_tuple__47 = PyTuple_Pack(37, __pyx_n_s_X, __pyx_n_s_bin_size, __pyx_n_s_min_length, __pyx_n_s_min_frequency, __pyx_n_s_N0, __pyx_n_s_N1, __pyx_n_s_num_bins, __pyx_n_s_bins, __pyx_n_s_bin_events, __pyx_n_s_on, __pyx_n_s_starts, __pyx_n_s_stops, __pyx_n_s_i, __pyx_n_s_window, __pyx_n_s_Z, __pyx_n_s_Z_where_list, __pyx_n_s_Z_where, __pyx_n_s_Z_len, __pyx_n_s_avalanche_lengths, __pyx_n_s_avalanche_array, __pyx_n_s_avalanche_shapes, __pyx_n_s_tup0, __pyx_n_s_tup1, __pyx_n_s_N0_avalanche, __pyx_n_s_avalanche_sizes, __pyx_n_s_a, __pyx_n_s_a0, __pyx_n_s_j, __pyx_n_s_size, __pyx_n_s_C_lengths, __pyx_n_s_C_sizes, __pyx_n_s_index_list, __pyx_n_s_avalanche_lengths_filtered, __pyx_n_s_avalanche_sizes_filtered, __pyx_n_s_avalanche_shapes_filtered, __pyx_n_s_avalanche_array_filtered, __pyx_n_s_channel_avalanches); if (unlikely(!__pyx_tuple__47)) __PYX_ERR(0, 217, __pyx_L1_error)
  __Pyx_GOTREF(__pyx_tuple__47);
  __Pyx_GIVEREF(__pyx_tuple__47);
/* … */
  __pyx_t_2 = PyCFunction_NewEx(&__pyx_mdef_10avalanches_9avalanches, NULL, __pyx_n_s_avalanches); if (unlikely(!__pyx_t_2)) __PYX_ERR(0, 217, __pyx_L1_error)
  __Pyx_GOTREF(__pyx_t_2);
  if (PyDict_SetItem(__pyx_d, __pyx_n_s_avalanches, __pyx_t_2) < 0) __PYX_ERR(0, 217, __pyx_L1_error)
  __Pyx_DECREF(__pyx_t_2); __pyx_t_2 = 0;
  __pyx_codeobj__48 = (PyObject*)__Pyx_PyCode_New(4, 0, 37, 0, CO_OPTIMIZED|CO_NEWLOCALS, __pyx_empty_bytes, __pyx_empty_tuple, __pyx_empty_tuple, __pyx_tuple__47, __pyx_empty_tuple, __pyx_empty_tuple, __pyx_kp_s_avalanches_pyx, __pyx_n_s_avalanches, 217, __pyx_empty_bytes); if (unlikely(!__pyx_codeobj__48)) __PYX_ERR(0, 217, __pyx_L1_error)
```

```
 218:     """
```

```
 219:     For a raster of data, returns the size and length of each avalanche, and how many times each channel participates in avalanches.
```

```
 220:
```

```
 221:     Arguments:
```

```
 222:         X:
```

```
 223:             A 2-dimensional Numpy array (dtype must be "int32")
```

```
 224:         bin_size:
```

```
 225:             The size of the bin.
```

```
 226:
```

```
 227:     Returns:
```

```
 228:         Avalanche_sizes:
```

```
 229:             The number of channels that participates in a given avalanche.
```

```
 230:         Avalanche_lengths:
```

```
 231:             The number of bins each avalanche persists for.
```

```
 232:         Channel_avalanches:
```

```
 233:             The number of times each channel participates in an avalanche.
```

```
 234:
```

```
 235:     To do:
```

```
 236:         Get the size of an avalanche over time, for shape-collapse.
```

```
 237:     """
```

```
 238:
```

```
 239:     # Static typing important variables
```

```
+240:     cdef int N0 = X.shape[0]
```

```
  __pyx_v_N0 = (__pyx_v_X.shape[0]);
```

```
+241:     cdef int N1 = X.shape[1]
```

```
  __pyx_v_N1 = (__pyx_v_X.shape[1]);
```

```
+242:     cdef int num_bins = (N1 - (N1 % bin_size)) // bin_size
```

```
  __pyx_v_num_bins = ((__pyx_v_N1 - (__pyx_v_N1 % __pyx_v_bin_size)) / __pyx_v_bin_size);
```

```
+243:     cdef int[:] bins = np.zeros(num_bins, dtype = "int32")
```

```
  __pyx_t_1 = __Pyx_GetModuleGlobalName(__pyx_n_s_np); if (unlikely(!__pyx_t_1)) __PYX_ERR(0, 243, __pyx_L1_error)
  __Pyx_GOTREF(__pyx_t_1);
  __pyx_t_2 = __Pyx_PyObject_GetAttrStr(__pyx_t_1, __pyx_n_s_zeros); if (unlikely(!__pyx_t_2)) __PYX_ERR(0, 243, __pyx_L1_error)
  __Pyx_GOTREF(__pyx_t_2);
  __Pyx_DECREF(__pyx_t_1); __pyx_t_1 = 0;
  __pyx_t_1 = __Pyx_PyInt_From_int(__pyx_v_num_bins); if (unlikely(!__pyx_t_1)) __PYX_ERR(0, 243, __pyx_L1_error)
  __Pyx_GOTREF(__pyx_t_1);
  __pyx_t_3 = PyTuple_New(1); if (unlikely(!__pyx_t_3)) __PYX_ERR(0, 243, __pyx_L1_error)
  __Pyx_GOTREF(__pyx_t_3);
  __Pyx_GIVEREF(__pyx_t_1);
  PyTuple_SET_ITEM(__pyx_t_3, 0, __pyx_t_1);
  __pyx_t_1 = 0;
  __pyx_t_1 = __Pyx_PyDict_NewPresized(1); if (unlikely(!__pyx_t_1)) __PYX_ERR(0, 243, __pyx_L1_error)
  __Pyx_GOTREF(__pyx_t_1);
  if (PyDict_SetItem(__pyx_t_1, __pyx_n_s_dtype, __pyx_n_s_int32) < 0) __PYX_ERR(0, 243, __pyx_L1_error)
  __pyx_t_4 = __Pyx_PyObject_Call(__pyx_t_2, __pyx_t_3, __pyx_t_1); if (unlikely(!__pyx_t_4)) __PYX_ERR(0, 243, __pyx_L1_error)
  __Pyx_GOTREF(__pyx_t_4);
  __Pyx_DECREF(__pyx_t_2); __pyx_t_2 = 0;
  __Pyx_DECREF(__pyx_t_3); __pyx_t_3 = 0;
  __Pyx_DECREF(__pyx_t_1); __pyx_t_1 = 0;
  __pyx_t_5 = __Pyx_PyObject_to_MemoryviewSlice_ds_int(__pyx_t_4, PyBUF_WRITABLE); if (unlikely(!__pyx_t_5.memview)) __PYX_ERR(0, 243, __pyx_L1_error)
  __Pyx_DECREF(__pyx_t_4); __pyx_t_4 = 0;
  __pyx_v_bins = __pyx_t_5;
  __pyx_t_5.memview = NULL;
  __pyx_t_5.data = NULL;
```

```
+244:     cdef int[:] bin_events = np.zeros(num_bins, dtype = "int32")
```

```
  __pyx_t_4 = __Pyx_GetModuleGlobalName(__pyx_n_s_np); if (unlikely(!__pyx_t_4)) __PYX_ERR(0, 244, __pyx_L1_error)
  __Pyx_GOTREF(__pyx_t_4);
  __pyx_t_1 = __Pyx_PyObject_GetAttrStr(__pyx_t_4, __pyx_n_s_zeros); if (unlikely(!__pyx_t_1)) __PYX_ERR(0, 244, __pyx_L1_error)
  __Pyx_GOTREF(__pyx_t_1);
  __Pyx_DECREF(__pyx_t_4); __pyx_t_4 = 0;
  __pyx_t_4 = __Pyx_PyInt_From_int(__pyx_v_num_bins); if (unlikely(!__pyx_t_4)) __PYX_ERR(0, 244, __pyx_L1_error)
  __Pyx_GOTREF(__pyx_t_4);
  __pyx_t_3 = PyTuple_New(1); if (unlikely(!__pyx_t_3)) __PYX_ERR(0, 244, __pyx_L1_error)
  __Pyx_GOTREF(__pyx_t_3);
  __Pyx_GIVEREF(__pyx_t_4);
  PyTuple_SET_ITEM(__pyx_t_3, 0, __pyx_t_4);
  __pyx_t_4 = 0;
  __pyx_t_4 = __Pyx_PyDict_NewPresized(1); if (unlikely(!__pyx_t_4)) __PYX_ERR(0, 244, __pyx_L1_error)
  __Pyx_GOTREF(__pyx_t_4);
  if (PyDict_SetItem(__pyx_t_4, __pyx_n_s_dtype, __pyx_n_s_int32) < 0) __PYX_ERR(0, 244, __pyx_L1_error)
  __pyx_t_2 = __Pyx_PyObject_Call(__pyx_t_1, __pyx_t_3, __pyx_t_4); if (unlikely(!__pyx_t_2)) __PYX_ERR(0, 244, __pyx_L1_error)
  __Pyx_GOTREF(__pyx_t_2);
  __Pyx_DECREF(__pyx_t_1); __pyx_t_1 = 0;
  __Pyx_DECREF(__pyx_t_3); __pyx_t_3 = 0;
  __Pyx_DECREF(__pyx_t_4); __pyx_t_4 = 0;
  __pyx_t_5 = __Pyx_PyObject_to_MemoryviewSlice_ds_int(__pyx_t_2, PyBUF_WRITABLE); if (unlikely(!__pyx_t_5.memview)) __PYX_ERR(0, 244, __pyx_L1_error)
  __Pyx_DECREF(__pyx_t_2); __pyx_t_2 = 0;
  __pyx_v_bin_events = __pyx_t_5;
  __pyx_t_5.memview = NULL;
  __pyx_t_5.data = NULL;
```

```
+245:     cdef bint on = False
```

```
  __pyx_v_on = 0;
```

```
+246:     cdef list starts = []
```

```
  __pyx_t_2 = PyList_New(0); if (unlikely(!__pyx_t_2)) __PYX_ERR(0, 246, __pyx_L1_error)
  __Pyx_GOTREF(__pyx_t_2);
  __pyx_v_starts = ((PyObject*)__pyx_t_2);
  __pyx_t_2 = 0;
```

```
+247:     cdef list stops = [],
```

```
  __pyx_t_2 = PyList_New(0); if (unlikely(!__pyx_t_2)) __PYX_ERR(0, 247, __pyx_L1_error)
  __Pyx_GOTREF(__pyx_t_2);
  __pyx_v_stops = ((PyObject*)__pyx_t_2);
  __pyx_t_2 = 0;
```

```
 248:     cdef int i
```

```
 249:     cdef int[:,:] window
```

```
 250:
```

```
 251:     #Finding bins where events occur:
```

```
+252:     for i in range(num_bins):
```

```
  __pyx_t_6 = __pyx_v_num_bins;
  __pyx_t_7 = __pyx_t_6;
  for (__pyx_t_8 = 0; __pyx_t_8 < __pyx_t_7; __pyx_t_8+=1) {
    __pyx_v_i = __pyx_t_8;
```

```
+253:         window = X[:, (i*bin_size):((i+1)*bin_size)] #Each window is one bin
```

```
    __pyx_t_9.data = __pyx_v_X.data;
    __pyx_t_9.memview = __pyx_v_X.memview;
    __PYX_INC_MEMVIEW(&__pyx_t_9, 0);
    __pyx_t_9.shape[0] = __pyx_v_X.shape[0];
__pyx_t_9.strides[0] = __pyx_v_X.strides[0];
    __pyx_t_9.suboffsets[0] = -1;

__pyx_t_10 = -1;
    if (unlikely(__pyx_memoryview_slice_memviewslice(
    &__pyx_t_9,
    __pyx_v_X.shape[1], __pyx_v_X.strides[1], __pyx_v_X.suboffsets[1],
    1,
    1,
    &__pyx_t_10,
    (__pyx_v_i * __pyx_v_bin_size),
    ((__pyx_v_i + 1) * __pyx_v_bin_size),
    0,
    1,
    1,
    0,
    1) < 0))
{
    __PYX_ERR(0, 253, __pyx_L1_error)
}

__PYX_XDEC_MEMVIEW(&__pyx_v_window, 1);
    __pyx_v_window = __pyx_t_9;
    __pyx_t_9.memview = NULL;
    __pyx_t_9.data = NULL;
```

```
+254:         if total_raster_sum(window) != 0: #If an event occurs:
```

```
    __pyx_t_11 = ((__pyx_f_10avalanches_total_raster_sum(__pyx_v_window) != 0) != 0);
    if (__pyx_t_11) {
/* … */
    }
  }
```

```
+255:             bins[i] = 1 #That bin is "on"
```

```
      __pyx_t_12 = __pyx_v_i;
      *((int *) ( /* dim=0 */ (__pyx_v_bins.data + __pyx_t_12 * __pyx_v_bins.strides[0]) )) = 1;
```

```
+256:             bin_events[i] = number_active_channels(window)
```

```
      __pyx_t_13 = __pyx_v_i;
      *((int *) ( /* dim=0 */ (__pyx_v_bin_events.data + __pyx_t_13 * __pyx_v_bin_events.strides[0]) )) = __pyx_f_10avalanches_number_active_channels(__pyx_v_window);
```

```
 257:
```

```
+258:     for i in range(num_bins):
```

```
  __pyx_t_6 = __pyx_v_num_bins;
  __pyx_t_7 = __pyx_t_6;
  for (__pyx_t_8 = 0; __pyx_t_8 < __pyx_t_7; __pyx_t_8+=1) {
    __pyx_v_i = __pyx_t_8;
```

```
+259:         if bins[i] == 1 and bins[i-1] == 0: #If it is is the start of an avlanche
```

```
    __pyx_t_14 = __pyx_v_i;
    __pyx_t_15 = (((*((int *) ( /* dim=0 */ (__pyx_v_bins.data + __pyx_t_14 * __pyx_v_bins.strides[0]) ))) == 1) != 0);
    if (__pyx_t_15) {
    } else {
      __pyx_t_11 = __pyx_t_15;
      goto __pyx_L9_bool_binop_done;
    }
    __pyx_t_16 = (__pyx_v_i - 1);
    __pyx_t_15 = (((*((int *) ( /* dim=0 */ (__pyx_v_bins.data + __pyx_t_16 * __pyx_v_bins.strides[0]) ))) == 0) != 0);
    __pyx_t_11 = __pyx_t_15;
    __pyx_L9_bool_binop_done:;
    if (__pyx_t_11) {
/* … */
      goto __pyx_L8;
    }
```

```
+260:             starts.append(i) #Note start time.
```

```
      __pyx_t_2 = __Pyx_PyInt_From_int(__pyx_v_i); if (unlikely(!__pyx_t_2)) __PYX_ERR(0, 260, __pyx_L1_error)
      __Pyx_GOTREF(__pyx_t_2);
      __pyx_t_17 = __Pyx_PyList_Append(__pyx_v_starts, __pyx_t_2); if (unlikely(__pyx_t_17 == ((int)-1))) __PYX_ERR(0, 260, __pyx_L1_error)
      __Pyx_DECREF(__pyx_t_2); __pyx_t_2 = 0;
```

```
+261:             on = True
```

```
      __pyx_v_on = 1;
```

```
+262:         elif bins[i] == 0 and bins[i-1] == 1 and on == True: #If it is the end of an avalanche:
```

```
    __pyx_t_18 = __pyx_v_i;
    __pyx_t_15 = (((*((int *) ( /* dim=0 */ (__pyx_v_bins.data + __pyx_t_18 * __pyx_v_bins.strides[0]) ))) == 0) != 0);
    if (__pyx_t_15) {
    } else {
      __pyx_t_11 = __pyx_t_15;
      goto __pyx_L11_bool_binop_done;
    }
    __pyx_t_19 = (__pyx_v_i - 1);
    __pyx_t_15 = (((*((int *) ( /* dim=0 */ (__pyx_v_bins.data + __pyx_t_19 * __pyx_v_bins.strides[0]) ))) == 1) != 0);
    if (__pyx_t_15) {
    } else {
      __pyx_t_11 = __pyx_t_15;
      goto __pyx_L11_bool_binop_done;
    }
    __pyx_t_15 = ((__pyx_v_on == 1) != 0);
    __pyx_t_11 = __pyx_t_15;
    __pyx_L11_bool_binop_done:;
    if (__pyx_t_11) {
/* … */
    }
    __pyx_L8:;
  }
```

```
+263:             stops.append(i) #Note end time.
```

```
      __pyx_t_2 = __Pyx_PyInt_From_int(__pyx_v_i); if (unlikely(!__pyx_t_2)) __PYX_ERR(0, 263, __pyx_L1_error)
      __Pyx_GOTREF(__pyx_t_2);
      __pyx_t_17 = __Pyx_PyList_Append(__pyx_v_stops, __pyx_t_2); if (unlikely(__pyx_t_17 == ((int)-1))) __PYX_ERR(0, 263, __pyx_L1_error)
      __Pyx_DECREF(__pyx_t_2); __pyx_t_2 = 0;
```

```
 264:
```

```
+265:     cdef int[:,:] Z = np.array(list(zip(starts, stops)), dtype = "int32") #Creates a list of tuples with each avalanches start and stop time.
```

```
  __pyx_t_2 = __Pyx_GetModuleGlobalName(__pyx_n_s_np); if (unlikely(!__pyx_t_2)) __PYX_ERR(0, 265, __pyx_L1_error)
  __Pyx_GOTREF(__pyx_t_2);
  __pyx_t_4 = __Pyx_PyObject_GetAttrStr(__pyx_t_2, __pyx_n_s_array); if (unlikely(!__pyx_t_4)) __PYX_ERR(0, 265, __pyx_L1_error)
  __Pyx_GOTREF(__pyx_t_4);
  __Pyx_DECREF(__pyx_t_2); __pyx_t_2 = 0;
  __pyx_t_2 = PyTuple_New(2); if (unlikely(!__pyx_t_2)) __PYX_ERR(0, 265, __pyx_L1_error)
  __Pyx_GOTREF(__pyx_t_2);
  __Pyx_INCREF(__pyx_v_starts);
  __Pyx_GIVEREF(__pyx_v_starts);
  PyTuple_SET_ITEM(__pyx_t_2, 0, __pyx_v_starts);
  __Pyx_INCREF(__pyx_v_stops);
  __Pyx_GIVEREF(__pyx_v_stops);
  PyTuple_SET_ITEM(__pyx_t_2, 1, __pyx_v_stops);
  __pyx_t_3 = __Pyx_PyObject_Call(__pyx_builtin_zip, __pyx_t_2, NULL); if (unlikely(!__pyx_t_3)) __PYX_ERR(0, 265, __pyx_L1_error)
  __Pyx_GOTREF(__pyx_t_3);
  __Pyx_DECREF(__pyx_t_2); __pyx_t_2 = 0;
  __pyx_t_2 = PySequence_List(__pyx_t_3); if (unlikely(!__pyx_t_2)) __PYX_ERR(0, 265, __pyx_L1_error)
  __Pyx_GOTREF(__pyx_t_2);
  __Pyx_DECREF(__pyx_t_3); __pyx_t_3 = 0;
  __pyx_t_3 = PyTuple_New(1); if (unlikely(!__pyx_t_3)) __PYX_ERR(0, 265, __pyx_L1_error)
  __Pyx_GOTREF(__pyx_t_3);
  __Pyx_GIVEREF(__pyx_t_2);
  PyTuple_SET_ITEM(__pyx_t_3, 0, __pyx_t_2);
  __pyx_t_2 = 0;
  __pyx_t_2 = __Pyx_PyDict_NewPresized(1); if (unlikely(!__pyx_t_2)) __PYX_ERR(0, 265, __pyx_L1_error)
  __Pyx_GOTREF(__pyx_t_2);
  if (PyDict_SetItem(__pyx_t_2, __pyx_n_s_dtype, __pyx_n_s_int32) < 0) __PYX_ERR(0, 265, __pyx_L1_error)
  __pyx_t_1 = __Pyx_PyObject_Call(__pyx_t_4, __pyx_t_3, __pyx_t_2); if (unlikely(!__pyx_t_1)) __PYX_ERR(0, 265, __pyx_L1_error)
  __Pyx_GOTREF(__pyx_t_1);
  __Pyx_DECREF(__pyx_t_4); __pyx_t_4 = 0;
  __Pyx_DECREF(__pyx_t_3); __pyx_t_3 = 0;
  __Pyx_DECREF(__pyx_t_2); __pyx_t_2 = 0;
  __pyx_t_9 = __Pyx_PyObject_to_MemoryviewSlice_dsds_int(__pyx_t_1, PyBUF_WRITABLE); if (unlikely(!__pyx_t_9.memview)) __PYX_ERR(0, 265, __pyx_L1_error)
  __Pyx_DECREF(__pyx_t_1); __pyx_t_1 = 0;
  __pyx_v_Z = __pyx_t_9;
  __pyx_t_9.memview = NULL;
  __pyx_t_9.data = NULL;
```

```
+266:     cdef list Z_where_list = []
```

```
  __pyx_t_1 = PyList_New(0); if (unlikely(!__pyx_t_1)) __PYX_ERR(0, 266, __pyx_L1_error)
  __Pyx_GOTREF(__pyx_t_1);
  __pyx_v_Z_where_list = ((PyObject*)__pyx_t_1);
  __pyx_t_1 = 0;
```

```
 267:
```

```
+268:     for i in range(Z.shape[0]):
```

```
  __pyx_t_20 = (__pyx_v_Z.shape[0]);
  __pyx_t_21 = __pyx_t_20;
  for (__pyx_t_6 = 0; __pyx_t_6 < __pyx_t_21; __pyx_t_6+=1) {
    __pyx_v_i = __pyx_t_6;
```

```
+269:         if Z[i][1] - Z[i][0] >= min_length:
```

```
    __pyx_t_22 = __pyx_v_i;
    __pyx_t_23 = 1;
    __pyx_t_24 = __pyx_v_i;
    __pyx_t_25 = 0;
    __pyx_t_11 = ((((*((int *) ( /* dim=1 */ (( /* dim=0 */ (__pyx_v_Z.data + __pyx_t_22 * __pyx_v_Z.strides[0]) ) + __pyx_t_23 * __pyx_v_Z.strides[1]) ))) - (*((int *) ( /* dim=1 */ (( /* dim=0 */ (__pyx_v_Z.data + __pyx_t_24 * __pyx_v_Z.strides[0]) ) + __pyx_t_25 * __pyx_v_Z.strides[1]) )))) >= __pyx_v_min_length) != 0);
    if (__pyx_t_11) {
/* … */
    }
  }
```

```
+270:             Z_where_list.append(i)
```

```
      __pyx_t_1 = __Pyx_PyInt_From_int(__pyx_v_i); if (unlikely(!__pyx_t_1)) __PYX_ERR(0, 270, __pyx_L1_error)
      __Pyx_GOTREF(__pyx_t_1);
      __pyx_t_17 = __Pyx_PyList_Append(__pyx_v_Z_where_list, __pyx_t_1); if (unlikely(__pyx_t_17 == ((int)-1))) __PYX_ERR(0, 270, __pyx_L1_error)
      __Pyx_DECREF(__pyx_t_1); __pyx_t_1 = 0;
```

```
 271:
```

```
+272:     cdef int[:] Z_where = np.array(Z_where_list, dtype = "int32")
```

```
  __pyx_t_1 = __Pyx_GetModuleGlobalName(__pyx_n_s_np); if (unlikely(!__pyx_t_1)) __PYX_ERR(0, 272, __pyx_L1_error)
  __Pyx_GOTREF(__pyx_t_1);
  __pyx_t_2 = __Pyx_PyObject_GetAttrStr(__pyx_t_1, __pyx_n_s_array); if (unlikely(!__pyx_t_2)) __PYX_ERR(0, 272, __pyx_L1_error)
  __Pyx_GOTREF(__pyx_t_2);
  __Pyx_DECREF(__pyx_t_1); __pyx_t_1 = 0;
  __pyx_t_1 = PyTuple_New(1); if (unlikely(!__pyx_t_1)) __PYX_ERR(0, 272, __pyx_L1_error)
  __Pyx_GOTREF(__pyx_t_1);
  __Pyx_INCREF(__pyx_v_Z_where_list);
  __Pyx_GIVEREF(__pyx_v_Z_where_list);
  PyTuple_SET_ITEM(__pyx_t_1, 0, __pyx_v_Z_where_list);
  __pyx_t_3 = __Pyx_PyDict_NewPresized(1); if (unlikely(!__pyx_t_3)) __PYX_ERR(0, 272, __pyx_L1_error)
  __Pyx_GOTREF(__pyx_t_3);
  if (PyDict_SetItem(__pyx_t_3, __pyx_n_s_dtype, __pyx_n_s_int32) < 0) __PYX_ERR(0, 272, __pyx_L1_error)
  __pyx_t_4 = __Pyx_PyObject_Call(__pyx_t_2, __pyx_t_1, __pyx_t_3); if (unlikely(!__pyx_t_4)) __PYX_ERR(0, 272, __pyx_L1_error)
  __Pyx_GOTREF(__pyx_t_4);
  __Pyx_DECREF(__pyx_t_2); __pyx_t_2 = 0;
  __Pyx_DECREF(__pyx_t_1); __pyx_t_1 = 0;
  __Pyx_DECREF(__pyx_t_3); __pyx_t_3 = 0;
  __pyx_t_5 = __Pyx_PyObject_to_MemoryviewSlice_ds_int(__pyx_t_4, PyBUF_WRITABLE); if (unlikely(!__pyx_t_5.memview)) __PYX_ERR(0, 272, __pyx_L1_error)
  __Pyx_DECREF(__pyx_t_4); __pyx_t_4 = 0;
  __pyx_v_Z_where = __pyx_t_5;
  __pyx_t_5.memview = NULL;
  __pyx_t_5.data = NULL;
```

```
+273:     cdef int Z_len = Z_where.shape[0] #The number of avalanches total
```

```
  __pyx_v_Z_len = (__pyx_v_Z_where.shape[0]);
```

```
 274:
```

```
+275:     cdef int[:] avalanche_lengths = np.zeros(Z_len, dtype = "int32")
```

```
  __pyx_t_4 = __Pyx_GetModuleGlobalName(__pyx_n_s_np); if (unlikely(!__pyx_t_4)) __PYX_ERR(0, 275, __pyx_L1_error)
  __Pyx_GOTREF(__pyx_t_4);
  __pyx_t_3 = __Pyx_PyObject_GetAttrStr(__pyx_t_4, __pyx_n_s_zeros); if (unlikely(!__pyx_t_3)) __PYX_ERR(0, 275, __pyx_L1_error)
  __Pyx_GOTREF(__pyx_t_3);
  __Pyx_DECREF(__pyx_t_4); __pyx_t_4 = 0;
  __pyx_t_4 = __Pyx_PyInt_From_int(__pyx_v_Z_len); if (unlikely(!__pyx_t_4)) __PYX_ERR(0, 275, __pyx_L1_error)
  __Pyx_GOTREF(__pyx_t_4);
  __pyx_t_1 = PyTuple_New(1); if (unlikely(!__pyx_t_1)) __PYX_ERR(0, 275, __pyx_L1_error)
  __Pyx_GOTREF(__pyx_t_1);
  __Pyx_GIVEREF(__pyx_t_4);
  PyTuple_SET_ITEM(__pyx_t_1, 0, __pyx_t_4);
  __pyx_t_4 = 0;
  __pyx_t_4 = __Pyx_PyDict_NewPresized(1); if (unlikely(!__pyx_t_4)) __PYX_ERR(0, 275, __pyx_L1_error)
  __Pyx_GOTREF(__pyx_t_4);
  if (PyDict_SetItem(__pyx_t_4, __pyx_n_s_dtype, __pyx_n_s_int32) < 0) __PYX_ERR(0, 275, __pyx_L1_error)
  __pyx_t_2 = __Pyx_PyObject_Call(__pyx_t_3, __pyx_t_1, __pyx_t_4); if (unlikely(!__pyx_t_2)) __PYX_ERR(0, 275, __pyx_L1_error)
  __Pyx_GOTREF(__pyx_t_2);
  __Pyx_DECREF(__pyx_t_3); __pyx_t_3 = 0;
  __Pyx_DECREF(__pyx_t_1); __pyx_t_1 = 0;
  __Pyx_DECREF(__pyx_t_4); __pyx_t_4 = 0;
  __pyx_t_5 = __Pyx_PyObject_to_MemoryviewSlice_ds_int(__pyx_t_2, PyBUF_WRITABLE); if (unlikely(!__pyx_t_5.memview)) __PYX_ERR(0, 275, __pyx_L1_error)
  __Pyx_DECREF(__pyx_t_2); __pyx_t_2 = 0;
  __pyx_v_avalanche_lengths = __pyx_t_5;
  __pyx_t_5.memview = NULL;
  __pyx_t_5.data = NULL;
```

```
+276:     cdef object[:] avalanche_array = np.zeros(Z_len, dtype = object)
```

```
  __pyx_t_2 = __Pyx_GetModuleGlobalName(__pyx_n_s_np); if (unlikely(!__pyx_t_2)) __PYX_ERR(0, 276, __pyx_L1_error)
  __Pyx_GOTREF(__pyx_t_2);
  __pyx_t_4 = __Pyx_PyObject_GetAttrStr(__pyx_t_2, __pyx_n_s_zeros); if (unlikely(!__pyx_t_4)) __PYX_ERR(0, 276, __pyx_L1_error)
  __Pyx_GOTREF(__pyx_t_4);
  __Pyx_DECREF(__pyx_t_2); __pyx_t_2 = 0;
  __pyx_t_2 = __Pyx_PyInt_From_int(__pyx_v_Z_len); if (unlikely(!__pyx_t_2)) __PYX_ERR(0, 276, __pyx_L1_error)
  __Pyx_GOTREF(__pyx_t_2);
  __pyx_t_1 = PyTuple_New(1); if (unlikely(!__pyx_t_1)) __PYX_ERR(0, 276, __pyx_L1_error)
  __Pyx_GOTREF(__pyx_t_1);
  __Pyx_GIVEREF(__pyx_t_2);
  PyTuple_SET_ITEM(__pyx_t_1, 0, __pyx_t_2);
  __pyx_t_2 = 0;
  __pyx_t_2 = __Pyx_PyDict_NewPresized(1); if (unlikely(!__pyx_t_2)) __PYX_ERR(0, 276, __pyx_L1_error)
  __Pyx_GOTREF(__pyx_t_2);
  if (PyDict_SetItem(__pyx_t_2, __pyx_n_s_dtype, __pyx_builtin_object) < 0) __PYX_ERR(0, 276, __pyx_L1_error)
  __pyx_t_3 = __Pyx_PyObject_Call(__pyx_t_4, __pyx_t_1, __pyx_t_2); if (unlikely(!__pyx_t_3)) __PYX_ERR(0, 276, __pyx_L1_error)
  __Pyx_GOTREF(__pyx_t_3);
  __Pyx_DECREF(__pyx_t_4); __pyx_t_4 = 0;
  __Pyx_DECREF(__pyx_t_1); __pyx_t_1 = 0;
  __Pyx_DECREF(__pyx_t_2); __pyx_t_2 = 0;
  __pyx_t_26 = __Pyx_PyObject_to_MemoryviewSlice_ds_object(__pyx_t_3, PyBUF_WRITABLE); if (unlikely(!__pyx_t_26.memview)) __PYX_ERR(0, 276, __pyx_L1_error)
  __Pyx_DECREF(__pyx_t_3); __pyx_t_3 = 0;
  __pyx_v_avalanche_array = __pyx_t_26;
  __pyx_t_26.memview = NULL;
  __pyx_t_26.data = NULL;
```

```
+277:     cdef object[:] avalanche_shapes = np.zeros(Z_len, dtype = object)
```

```
  __pyx_t_3 = __Pyx_GetModuleGlobalName(__pyx_n_s_np); if (unlikely(!__pyx_t_3)) __PYX_ERR(0, 277, __pyx_L1_error)
  __Pyx_GOTREF(__pyx_t_3);
  __pyx_t_2 = __Pyx_PyObject_GetAttrStr(__pyx_t_3, __pyx_n_s_zeros); if (unlikely(!__pyx_t_2)) __PYX_ERR(0, 277, __pyx_L1_error)
  __Pyx_GOTREF(__pyx_t_2);
  __Pyx_DECREF(__pyx_t_3); __pyx_t_3 = 0;
  __pyx_t_3 = __Pyx_PyInt_From_int(__pyx_v_Z_len); if (unlikely(!__pyx_t_3)) __PYX_ERR(0, 277, __pyx_L1_error)
  __Pyx_GOTREF(__pyx_t_3);
  __pyx_t_1 = PyTuple_New(1); if (unlikely(!__pyx_t_1)) __PYX_ERR(0, 277, __pyx_L1_error)
  __Pyx_GOTREF(__pyx_t_1);
  __Pyx_GIVEREF(__pyx_t_3);
  PyTuple_SET_ITEM(__pyx_t_1, 0, __pyx_t_3);
  __pyx_t_3 = 0;
  __pyx_t_3 = __Pyx_PyDict_NewPresized(1); if (unlikely(!__pyx_t_3)) __PYX_ERR(0, 277, __pyx_L1_error)
  __Pyx_GOTREF(__pyx_t_3);
  if (PyDict_SetItem(__pyx_t_3, __pyx_n_s_dtype, __pyx_builtin_object) < 0) __PYX_ERR(0, 277, __pyx_L1_error)
  __pyx_t_4 = __Pyx_PyObject_Call(__pyx_t_2, __pyx_t_1, __pyx_t_3); if (unlikely(!__pyx_t_4)) __PYX_ERR(0, 277, __pyx_L1_error)
  __Pyx_GOTREF(__pyx_t_4);
  __Pyx_DECREF(__pyx_t_2); __pyx_t_2 = 0;
  __Pyx_DECREF(__pyx_t_1); __pyx_t_1 = 0;
  __Pyx_DECREF(__pyx_t_3); __pyx_t_3 = 0;
  __pyx_t_26 = __Pyx_PyObject_to_MemoryviewSlice_ds_object(__pyx_t_4, PyBUF_WRITABLE); if (unlikely(!__pyx_t_26.memview)) __PYX_ERR(0, 277, __pyx_L1_error)
  __Pyx_DECREF(__pyx_t_4); __pyx_t_4 = 0;
  __pyx_v_avalanche_shapes = __pyx_t_26;
  __pyx_t_26.memview = NULL;
  __pyx_t_26.data = NULL;
```

```
 278:
```

```
 279:     cdef int tup0, tup1
```

```
 280:
```

```
+281:     for i in range(Z_len):
```

```
  __pyx_t_6 = __pyx_v_Z_len;
  __pyx_t_7 = __pyx_t_6;
  for (__pyx_t_8 = 0; __pyx_t_8 < __pyx_t_7; __pyx_t_8+=1) {
    __pyx_v_i = __pyx_t_8;
```

```
+282:         tup0 = Z[Z_where[i]][0]
```

```
    __pyx_t_27 = __pyx_v_i;
    __pyx_t_28 = (*((int *) ( /* dim=0 */ (__pyx_v_Z_where.data + __pyx_t_27 * __pyx_v_Z_where.strides[0]) )));
    __pyx_t_29 = 0;
    __pyx_v_tup0 = (*((int *) ( /* dim=1 */ (( /* dim=0 */ (__pyx_v_Z.data + __pyx_t_28 * __pyx_v_Z.strides[0]) ) + __pyx_t_29 * __pyx_v_Z.strides[1]) )));
```

```
+283:         tup1 = Z[Z_where[i]][1]
```

```
    __pyx_t_30 = __pyx_v_i;
    __pyx_t_31 = (*((int *) ( /* dim=0 */ (__pyx_v_Z_where.data + __pyx_t_30 * __pyx_v_Z_where.strides[0]) )));
    __pyx_t_32 = 1;
    __pyx_v_tup1 = (*((int *) ( /* dim=1 */ (( /* dim=0 */ (__pyx_v_Z.data + __pyx_t_31 * __pyx_v_Z.strides[0]) ) + __pyx_t_32 * __pyx_v_Z.strides[1]) )));
```

```
+284:         avalanche_lengths[i] = tup1 - tup0
```

```
    __pyx_t_33 = __pyx_v_i;
    *((int *) ( /* dim=0 */ (__pyx_v_avalanche_lengths.data + __pyx_t_33 * __pyx_v_avalanche_lengths.strides[0]) )) = (__pyx_v_tup1 - __pyx_v_tup0);
```

```
+285:         avalanche_array[i] = X[:,bin_size*tup0:bin_size*tup1] #Grabbing each avalanche from the original array
```

```
    __pyx_t_9.data = __pyx_v_X.data;
    __pyx_t_9.memview = __pyx_v_X.memview;
    __PYX_INC_MEMVIEW(&__pyx_t_9, 0);
    __pyx_t_9.shape[0] = __pyx_v_X.shape[0];
__pyx_t_9.strides[0] = __pyx_v_X.strides[0];
    __pyx_t_9.suboffsets[0] = -1;

__pyx_t_10 = -1;
    if (unlikely(__pyx_memoryview_slice_memviewslice(
    &__pyx_t_9,
    __pyx_v_X.shape[1], __pyx_v_X.strides[1], __pyx_v_X.suboffsets[1],
    1,
    1,
    &__pyx_t_10,
    (__pyx_v_bin_size * __pyx_v_tup0),
    (__pyx_v_bin_size * __pyx_v_tup1),
    0,
    1,
    1,
    0,
    1) < 0))
{
    __PYX_ERR(0, 285, __pyx_L1_error)
}

__pyx_t_4 = __pyx_memoryview_fromslice(__pyx_t_9, 2, (PyObject *(*)(char *)) __pyx_memview_get_int, (int (*)(char *, PyObject *)) __pyx_memview_set_int, 0);; if (unlikely(!__pyx_t_4)) __PYX_ERR(0, 285, __pyx_L1_error)
    __Pyx_GOTREF(__pyx_t_4);
    __PYX_XDEC_MEMVIEW(&__pyx_t_9, 1);
    __pyx_t_9.memview = NULL;
    __pyx_t_9.data = NULL;
    __pyx_t_34 = __pyx_v_i;
    __pyx_t_35 = ((PyObject * *) ( /* dim=0 */ (__pyx_v_avalanche_array.data + __pyx_t_34 * __pyx_v_avalanche_array.strides[0]) ));
    __Pyx_GOTREF(*__pyx_t_35);
    __Pyx_INCREF(__pyx_t_4); __Pyx_DECREF(*__pyx_t_35);
    *__pyx_t_35 = __pyx_t_4;
    __Pyx_GIVEREF(*__pyx_t_35);
    __Pyx_DECREF(__pyx_t_4); __pyx_t_4 = 0;
```

```
 286:         #avalanche_lengths[i] = bin_size*(tup1 - tup0) #The length is recorded as the number of bins, times the length of each bin.
```

```
 287:         #This makes avalanches easy to convert to seconds, so long as you know the sampling rate of the original data.
```

```
+288:         avalanche_shapes[i] = np.array(bin_events[tup0:tup1])
```

```
    __pyx_t_3 = __Pyx_GetModuleGlobalName(__pyx_n_s_np); if (unlikely(!__pyx_t_3)) __PYX_ERR(0, 288, __pyx_L1_error)
    __Pyx_GOTREF(__pyx_t_3);
    __pyx_t_1 = __Pyx_PyObject_GetAttrStr(__pyx_t_3, __pyx_n_s_array); if (unlikely(!__pyx_t_1)) __PYX_ERR(0, 288, __pyx_L1_error)
    __Pyx_GOTREF(__pyx_t_1);
    __Pyx_DECREF(__pyx_t_3); __pyx_t_3 = 0;
    __pyx_t_5.data = __pyx_v_bin_events.data;
    __pyx_t_5.memview = __pyx_v_bin_events.memview;
    __PYX_INC_MEMVIEW(&__pyx_t_5, 0);
    __pyx_t_10 = -1;
    if (unlikely(__pyx_memoryview_slice_memviewslice(
    &__pyx_t_5,
    __pyx_v_bin_events.shape[0], __pyx_v_bin_events.strides[0], __pyx_v_bin_events.suboffsets[0],
    0,
    0,
    &__pyx_t_10,
    __pyx_v_tup0,
    __pyx_v_tup1,
    0,
    1,
    1,
    0,
    1) < 0))
{
    __PYX_ERR(0, 288, __pyx_L1_error)
}

__pyx_t_3 = __pyx_memoryview_fromslice(__pyx_t_5, 1, (PyObject *(*)(char *)) __pyx_memview_get_int, (int (*)(char *, PyObject *)) __pyx_memview_set_int, 0);; if (unlikely(!__pyx_t_3)) __PYX_ERR(0, 288, __pyx_L1_error)
    __Pyx_GOTREF(__pyx_t_3);
    __PYX_XDEC_MEMVIEW(&__pyx_t_5, 1);
    __pyx_t_5.memview = NULL;
    __pyx_t_5.data = NULL;
    __pyx_t_2 = NULL;
    if (CYTHON_UNPACK_METHODS && unlikely(PyMethod_Check(__pyx_t_1))) {
      __pyx_t_2 = PyMethod_GET_SELF(__pyx_t_1);
      if (likely(__pyx_t_2)) {
        PyObject* function = PyMethod_GET_FUNCTION(__pyx_t_1);
        __Pyx_INCREF(__pyx_t_2);
        __Pyx_INCREF(function);
        __Pyx_DECREF_SET(__pyx_t_1, function);
      }
    }
    if (!__pyx_t_2) {
      __pyx_t_4 = __Pyx_PyObject_CallOneArg(__pyx_t_1, __pyx_t_3); if (unlikely(!__pyx_t_4)) __PYX_ERR(0, 288, __pyx_L1_error)
      __Pyx_DECREF(__pyx_t_3); __pyx_t_3 = 0;
      __Pyx_GOTREF(__pyx_t_4);
    } else {
      #if CYTHON_FAST_PYCALL
      if (PyFunction_Check(__pyx_t_1)) {
        PyObject *__pyx_temp[2] = {__pyx_t_2, __pyx_t_3};
        __pyx_t_4 = __Pyx_PyFunction_FastCall(__pyx_t_1, __pyx_temp+1-1, 1+1); if (unlikely(!__pyx_t_4)) __PYX_ERR(0, 288, __pyx_L1_error)
        __Pyx_XDECREF(__pyx_t_2); __pyx_t_2 = 0;
        __Pyx_GOTREF(__pyx_t_4);
        __Pyx_DECREF(__pyx_t_3); __pyx_t_3 = 0;
      } else
      #endif
      #if CYTHON_FAST_PYCCALL
      if (__Pyx_PyFastCFunction_Check(__pyx_t_1)) {
        PyObject *__pyx_temp[2] = {__pyx_t_2, __pyx_t_3};
        __pyx_t_4 = __Pyx_PyCFunction_FastCall(__pyx_t_1, __pyx_temp+1-1, 1+1); if (unlikely(!__pyx_t_4)) __PYX_ERR(0, 288, __pyx_L1_error)
        __Pyx_XDECREF(__pyx_t_2); __pyx_t_2 = 0;
        __Pyx_GOTREF(__pyx_t_4);
        __Pyx_DECREF(__pyx_t_3); __pyx_t_3 = 0;
      } else
      #endif
      {
        __pyx_t_36 = PyTuple_New(1+1); if (unlikely(!__pyx_t_36)) __PYX_ERR(0, 288, __pyx_L1_error)
        __Pyx_GOTREF(__pyx_t_36);
        __Pyx_GIVEREF(__pyx_t_2); PyTuple_SET_ITEM(__pyx_t_36, 0, __pyx_t_2); __pyx_t_2 = NULL;
        __Pyx_GIVEREF(__pyx_t_3);
        PyTuple_SET_ITEM(__pyx_t_36, 0+1, __pyx_t_3);
        __pyx_t_3 = 0;
        __pyx_t_4 = __Pyx_PyObject_Call(__pyx_t_1, __pyx_t_36, NULL); if (unlikely(!__pyx_t_4)) __PYX_ERR(0, 288, __pyx_L1_error)
        __Pyx_GOTREF(__pyx_t_4);
        __Pyx_DECREF(__pyx_t_36); __pyx_t_36 = 0;
      }
    }
    __Pyx_DECREF(__pyx_t_1); __pyx_t_1 = 0;
    __pyx_t_37 = __pyx_v_i;
    __pyx_t_35 = ((PyObject * *) ( /* dim=0 */ (__pyx_v_avalanche_shapes.data + __pyx_t_37 * __pyx_v_avalanche_shapes.strides[0]) ));
    __Pyx_GOTREF(*__pyx_t_35);
    __Pyx_INCREF(__pyx_t_4); __Pyx_DECREF(*__pyx_t_35);
    *__pyx_t_35 = __pyx_t_4;
    __Pyx_GIVEREF(*__pyx_t_35);
    __Pyx_DECREF(__pyx_t_4); __pyx_t_4 = 0;
  }
```

```
 289:
```

```
+290:     cdef int N0_avalanche = avalanche_array.shape[0]
```

```
  __pyx_v_N0_avalanche = (__pyx_v_avalanche_array.shape[0]);
```

```
+291:     cdef int[:] avalanche_sizes = np.zeros(Z_len, dtype = "int32")
```

```
  __pyx_t_4 = __Pyx_GetModuleGlobalName(__pyx_n_s_np); if (unlikely(!__pyx_t_4)) __PYX_ERR(0, 291, __pyx_L1_error)
  __Pyx_GOTREF(__pyx_t_4);
  __pyx_t_1 = __Pyx_PyObject_GetAttrStr(__pyx_t_4, __pyx_n_s_zeros); if (unlikely(!__pyx_t_1)) __PYX_ERR(0, 291, __pyx_L1_error)
  __Pyx_GOTREF(__pyx_t_1);
  __Pyx_DECREF(__pyx_t_4); __pyx_t_4 = 0;
  __pyx_t_4 = __Pyx_PyInt_From_int(__pyx_v_Z_len); if (unlikely(!__pyx_t_4)) __PYX_ERR(0, 291, __pyx_L1_error)
  __Pyx_GOTREF(__pyx_t_4);
  __pyx_t_36 = PyTuple_New(1); if (unlikely(!__pyx_t_36)) __PYX_ERR(0, 291, __pyx_L1_error)
  __Pyx_GOTREF(__pyx_t_36);
  __Pyx_GIVEREF(__pyx_t_4);
  PyTuple_SET_ITEM(__pyx_t_36, 0, __pyx_t_4);
  __pyx_t_4 = 0;
  __pyx_t_4 = __Pyx_PyDict_NewPresized(1); if (unlikely(!__pyx_t_4)) __PYX_ERR(0, 291, __pyx_L1_error)
  __Pyx_GOTREF(__pyx_t_4);
  if (PyDict_SetItem(__pyx_t_4, __pyx_n_s_dtype, __pyx_n_s_int32) < 0) __PYX_ERR(0, 291, __pyx_L1_error)
  __pyx_t_3 = __Pyx_PyObject_Call(__pyx_t_1, __pyx_t_36, __pyx_t_4); if (unlikely(!__pyx_t_3)) __PYX_ERR(0, 291, __pyx_L1_error)
  __Pyx_GOTREF(__pyx_t_3);
  __Pyx_DECREF(__pyx_t_1); __pyx_t_1 = 0;
  __Pyx_DECREF(__pyx_t_36); __pyx_t_36 = 0;
  __Pyx_DECREF(__pyx_t_4); __pyx_t_4 = 0;
  __pyx_t_5 = __Pyx_PyObject_to_MemoryviewSlice_ds_int(__pyx_t_3, PyBUF_WRITABLE); if (unlikely(!__pyx_t_5.memview)) __PYX_ERR(0, 291, __pyx_L1_error)
  __Pyx_DECREF(__pyx_t_3); __pyx_t_3 = 0;
  __pyx_v_avalanche_sizes = __pyx_t_5;
  __pyx_t_5.memview = NULL;
  __pyx_t_5.data = NULL;
```

```
 292:
```

```
 293:     cdef int[:,:] a
```

```
 294:     cdef int a0, j
```

```
+295:     cdef int size = 0
```

```
  __pyx_v_size = 0;
```

```
+296:     for i in range(N0_avalanche):
```

```
  __pyx_t_6 = __pyx_v_N0_avalanche;
  __pyx_t_7 = __pyx_t_6;
  for (__pyx_t_8 = 0; __pyx_t_8 < __pyx_t_7; __pyx_t_8+=1) {
    __pyx_v_i = __pyx_t_8;
```

```
+297:         a = avalanche_array[i]
```

```
    __pyx_t_38 = __pyx_v_i;
    __pyx_t_3 = (PyObject *) *((PyObject * *) ( /* dim=0 */ (__pyx_v_avalanche_array.data + __pyx_t_38 * __pyx_v_avalanche_array.strides[0]) ));
    __Pyx_INCREF((PyObject*)__pyx_t_3);
    __pyx_t_9 = __Pyx_PyObject_to_MemoryviewSlice_dsds_int(__pyx_t_3, PyBUF_WRITABLE); if (unlikely(!__pyx_t_9.memview)) __PYX_ERR(0, 297, __pyx_L1_error)
    __Pyx_DECREF(__pyx_t_3); __pyx_t_3 = 0;
    __PYX_XDEC_MEMVIEW(&__pyx_v_a, 1);
    __pyx_v_a = __pyx_t_9;
    __pyx_t_9.memview = NULL;
    __pyx_t_9.data = NULL;
```

```
+298:         a0 = a.shape[0]
```

```
    __pyx_v_a0 = (__pyx_v_a.shape[0]);
```

```
 299:
```

```
+300:         size = 0
```

```
    __pyx_v_size = 0;
```

```
 301:
```

```
+302:         for j in range(a0):
```

```
    __pyx_t_10 = __pyx_v_a0;
    __pyx_t_39 = __pyx_t_10;
    for (__pyx_t_40 = 0; __pyx_t_40 < __pyx_t_39; __pyx_t_40+=1) {
      __pyx_v_j = __pyx_t_40;
```

```
+303:             if sum(a[j]) >= 1:
```

```
      __pyx_t_5.data = __pyx_v_a.data;
      __pyx_t_5.memview = __pyx_v_a.memview;
      __PYX_INC_MEMVIEW(&__pyx_t_5, 0);
      {
    Py_ssize_t __pyx_tmp_idx = __pyx_v_j;
    Py_ssize_t __pyx_tmp_shape = __pyx_v_a.shape[0];
    Py_ssize_t __pyx_tmp_stride = __pyx_v_a.strides[0];
    if (0 && (__pyx_tmp_idx < 0))
        __pyx_tmp_idx += __pyx_tmp_shape;
    if (0 && (__pyx_tmp_idx < 0 || __pyx_tmp_idx >= __pyx_tmp_shape)) {
        PyErr_SetString(PyExc_IndexError, "Index out of bounds (axis 0)");
        __PYX_ERR(0, 303, __pyx_L1_error)
    }
        __pyx_t_5.data += __pyx_tmp_idx * __pyx_tmp_stride;
}

__pyx_t_5.shape[0] = __pyx_v_a.shape[1];
__pyx_t_5.strides[0] = __pyx_v_a.strides[1];
    __pyx_t_5.suboffsets[0] = -1;

__pyx_t_3 = __pyx_memoryview_fromslice(__pyx_t_5, 1, (PyObject *(*)(char *)) __pyx_memview_get_int, (int (*)(char *, PyObject *)) __pyx_memview_set_int, 0);; if (unlikely(!__pyx_t_3)) __PYX_ERR(0, 303, __pyx_L1_error)
      __Pyx_GOTREF(__pyx_t_3);
      __PYX_XDEC_MEMVIEW(&__pyx_t_5, 1);
      __pyx_t_5.memview = NULL;
      __pyx_t_5.data = NULL;
      __pyx_t_4 = __Pyx_PyObject_CallOneArg(__pyx_builtin_sum, __pyx_t_3); if (unlikely(!__pyx_t_4)) __PYX_ERR(0, 303, __pyx_L1_error)
      __Pyx_GOTREF(__pyx_t_4);
      __Pyx_DECREF(__pyx_t_3); __pyx_t_3 = 0;
      __pyx_t_3 = PyObject_RichCompare(__pyx_t_4, __pyx_int_1, Py_GE); __Pyx_XGOTREF(__pyx_t_3); if (unlikely(!__pyx_t_3)) __PYX_ERR(0, 303, __pyx_L1_error)
      __Pyx_DECREF(__pyx_t_4); __pyx_t_4 = 0;
      __pyx_t_11 = __Pyx_PyObject_IsTrue(__pyx_t_3); if (unlikely(__pyx_t_11 < 0)) __PYX_ERR(0, 303, __pyx_L1_error)
      __Pyx_DECREF(__pyx_t_3); __pyx_t_3 = 0;
      if (__pyx_t_11) {
/* … */
      }
    }
```

```
+304:                 size += 1
```

```
        __pyx_v_size = (__pyx_v_size + 1);
```

```
 305:
```

```
+306:         avalanche_sizes[i] = size
```

```
    __pyx_t_41 = __pyx_v_i;
    *((int *) ( /* dim=0 */ (__pyx_v_avalanche_sizes.data + __pyx_t_41 * __pyx_v_avalanche_sizes.strides[0]) )) = __pyx_v_size;
  }
```

```
 307:
```

```
+308:     C_lengths = Counter(avalanche_lengths)
```

```
  __pyx_t_4 = __Pyx_GetModuleGlobalName(__pyx_n_s_Counter); if (unlikely(!__pyx_t_4)) __PYX_ERR(0, 308, __pyx_L1_error)
  __Pyx_GOTREF(__pyx_t_4);
  __pyx_t_36 = __pyx_memoryview_fromslice(__pyx_v_avalanche_lengths, 1, (PyObject *(*)(char *)) __pyx_memview_get_int, (int (*)(char *, PyObject *)) __pyx_memview_set_int, 0);; if (unlikely(!__pyx_t_36)) __PYX_ERR(0, 308, __pyx_L1_error)
  __Pyx_GOTREF(__pyx_t_36);
  __pyx_t_1 = NULL;
  if (CYTHON_UNPACK_METHODS && unlikely(PyMethod_Check(__pyx_t_4))) {
    __pyx_t_1 = PyMethod_GET_SELF(__pyx_t_4);
    if (likely(__pyx_t_1)) {
      PyObject* function = PyMethod_GET_FUNCTION(__pyx_t_4);
      __Pyx_INCREF(__pyx_t_1);
      __Pyx_INCREF(function);
      __Pyx_DECREF_SET(__pyx_t_4, function);
    }
  }
  if (!__pyx_t_1) {
    __pyx_t_3 = __Pyx_PyObject_CallOneArg(__pyx_t_4, __pyx_t_36); if (unlikely(!__pyx_t_3)) __PYX_ERR(0, 308, __pyx_L1_error)
    __Pyx_DECREF(__pyx_t_36); __pyx_t_36 = 0;
    __Pyx_GOTREF(__pyx_t_3);
  } else {
    #if CYTHON_FAST_PYCALL
    if (PyFunction_Check(__pyx_t_4)) {
      PyObject *__pyx_temp[2] = {__pyx_t_1, __pyx_t_36};
      __pyx_t_3 = __Pyx_PyFunction_FastCall(__pyx_t_4, __pyx_temp+1-1, 1+1); if (unlikely(!__pyx_t_3)) __PYX_ERR(0, 308, __pyx_L1_error)
      __Pyx_XDECREF(__pyx_t_1); __pyx_t_1 = 0;
      __Pyx_GOTREF(__pyx_t_3);
      __Pyx_DECREF(__pyx_t_36); __pyx_t_36 = 0;
    } else
    #endif
    #if CYTHON_FAST_PYCCALL
    if (__Pyx_PyFastCFunction_Check(__pyx_t_4)) {
      PyObject *__pyx_temp[2] = {__pyx_t_1, __pyx_t_36};
      __pyx_t_3 = __Pyx_PyCFunction_FastCall(__pyx_t_4, __pyx_temp+1-1, 1+1); if (unlikely(!__pyx_t_3)) __PYX_ERR(0, 308, __pyx_L1_error)
      __Pyx_XDECREF(__pyx_t_1); __pyx_t_1 = 0;
      __Pyx_GOTREF(__pyx_t_3);
      __Pyx_DECREF(__pyx_t_36); __pyx_t_36 = 0;
    } else
    #endif
    {
      __pyx_t_2 = PyTuple_New(1+1); if (unlikely(!__pyx_t_2)) __PYX_ERR(0, 308, __pyx_L1_error)
      __Pyx_GOTREF(__pyx_t_2);
      __Pyx_GIVEREF(__pyx_t_1); PyTuple_SET_ITEM(__pyx_t_2, 0, __pyx_t_1); __pyx_t_1 = NULL;
      __Pyx_GIVEREF(__pyx_t_36);
      PyTuple_SET_ITEM(__pyx_t_2, 0+1, __pyx_t_36);
      __pyx_t_36 = 0;
      __pyx_t_3 = __Pyx_PyObject_Call(__pyx_t_4, __pyx_t_2, NULL); if (unlikely(!__pyx_t_3)) __PYX_ERR(0, 308, __pyx_L1_error)
      __Pyx_GOTREF(__pyx_t_3);
      __Pyx_DECREF(__pyx_t_2); __pyx_t_2 = 0;
    }
  }
  __Pyx_DECREF(__pyx_t_4); __pyx_t_4 = 0;
  __pyx_v_C_lengths = __pyx_t_3;
  __pyx_t_3 = 0;
```

```
+309:     C_sizes = Counter(avalanche_sizes)
```

```
  __pyx_t_4 = __Pyx_GetModuleGlobalName(__pyx_n_s_Counter); if (unlikely(!__pyx_t_4)) __PYX_ERR(0, 309, __pyx_L1_error)
  __Pyx_GOTREF(__pyx_t_4);
  __pyx_t_2 = __pyx_memoryview_fromslice(__pyx_v_avalanche_sizes, 1, (PyObject *(*)(char *)) __pyx_memview_get_int, (int (*)(char *, PyObject *)) __pyx_memview_set_int, 0);; if (unlikely(!__pyx_t_2)) __PYX_ERR(0, 309, __pyx_L1_error)
  __Pyx_GOTREF(__pyx_t_2);
  __pyx_t_36 = NULL;
  if (CYTHON_UNPACK_METHODS && unlikely(PyMethod_Check(__pyx_t_4))) {
    __pyx_t_36 = PyMethod_GET_SELF(__pyx_t_4);
    if (likely(__pyx_t_36)) {
      PyObject* function = PyMethod_GET_FUNCTION(__pyx_t_4);
      __Pyx_INCREF(__pyx_t_36);
      __Pyx_INCREF(function);
      __Pyx_DECREF_SET(__pyx_t_4, function);
    }
  }
  if (!__pyx_t_36) {
    __pyx_t_3 = __Pyx_PyObject_CallOneArg(__pyx_t_4, __pyx_t_2); if (unlikely(!__pyx_t_3)) __PYX_ERR(0, 309, __pyx_L1_error)
    __Pyx_DECREF(__pyx_t_2); __pyx_t_2 = 0;
    __Pyx_GOTREF(__pyx_t_3);
  } else {
    #if CYTHON_FAST_PYCALL
    if (PyFunction_Check(__pyx_t_4)) {
      PyObject *__pyx_temp[2] = {__pyx_t_36, __pyx_t_2};
      __pyx_t_3 = __Pyx_PyFunction_FastCall(__pyx_t_4, __pyx_temp+1-1, 1+1); if (unlikely(!__pyx_t_3)) __PYX_ERR(0, 309, __pyx_L1_error)
      __Pyx_XDECREF(__pyx_t_36); __pyx_t_36 = 0;
      __Pyx_GOTREF(__pyx_t_3);
      __Pyx_DECREF(__pyx_t_2); __pyx_t_2 = 0;
    } else
    #endif
    #if CYTHON_FAST_PYCCALL
    if (__Pyx_PyFastCFunction_Check(__pyx_t_4)) {
      PyObject *__pyx_temp[2] = {__pyx_t_36, __pyx_t_2};
      __pyx_t_3 = __Pyx_PyCFunction_FastCall(__pyx_t_4, __pyx_temp+1-1, 1+1); if (unlikely(!__pyx_t_3)) __PYX_ERR(0, 309, __pyx_L1_error)
      __Pyx_XDECREF(__pyx_t_36); __pyx_t_36 = 0;
      __Pyx_GOTREF(__pyx_t_3);
      __Pyx_DECREF(__pyx_t_2); __pyx_t_2 = 0;
    } else
    #endif
    {
      __pyx_t_1 = PyTuple_New(1+1); if (unlikely(!__pyx_t_1)) __PYX_ERR(0, 309, __pyx_L1_error)
      __Pyx_GOTREF(__pyx_t_1);
      __Pyx_GIVEREF(__pyx_t_36); PyTuple_SET_ITEM(__pyx_t_1, 0, __pyx_t_36); __pyx_t_36 = NULL;
      __Pyx_GIVEREF(__pyx_t_2);
      PyTuple_SET_ITEM(__pyx_t_1, 0+1, __pyx_t_2);
      __pyx_t_2 = 0;
      __pyx_t_3 = __Pyx_PyObject_Call(__pyx_t_4, __pyx_t_1, NULL); if (unlikely(!__pyx_t_3)) __PYX_ERR(0, 309, __pyx_L1_error)
      __Pyx_GOTREF(__pyx_t_3);
      __Pyx_DECREF(__pyx_t_1); __pyx_t_1 = 0;
    }
  }
  __Pyx_DECREF(__pyx_t_4); __pyx_t_4 = 0;
  __pyx_v_C_sizes = __pyx_t_3;
  __pyx_t_3 = 0;
```

```
 310:
```

```
+311:     cdef list index_list = []
```

```
  __pyx_t_3 = PyList_New(0); if (unlikely(!__pyx_t_3)) __PYX_ERR(0, 311, __pyx_L1_error)
  __Pyx_GOTREF(__pyx_t_3);
  __pyx_v_index_list = ((PyObject*)__pyx_t_3);
  __pyx_t_3 = 0;
```

```
 312:
```

```
+313:     for i in range(N0_avalanche):
```

```
  __pyx_t_6 = __pyx_v_N0_avalanche;
  __pyx_t_7 = __pyx_t_6;
  for (__pyx_t_8 = 0; __pyx_t_8 < __pyx_t_7; __pyx_t_8+=1) {
    __pyx_v_i = __pyx_t_8;
```

```
+314:         if C_lengths[avalanche_lengths[i]] < min_frequency or C_sizes[avalanche_sizes[i]] < min_frequency:
```

```
    __pyx_t_42 = __pyx_v_i;
    __pyx_t_10 = (*((int *) ( /* dim=0 */ (__pyx_v_avalanche_lengths.data + __pyx_t_42 * __pyx_v_avalanche_lengths.strides[0]) )));
    __pyx_t_3 = __Pyx_GetItemInt(__pyx_v_C_lengths, __pyx_t_10, int, 1, __Pyx_PyInt_From_int, 0, 0, 0); if (unlikely(!__pyx_t_3)) __PYX_ERR(0, 314, __pyx_L1_error)
    __Pyx_GOTREF(__pyx_t_3);
    __pyx_t_4 = __Pyx_PyInt_From_int(__pyx_v_min_frequency); if (unlikely(!__pyx_t_4)) __PYX_ERR(0, 314, __pyx_L1_error)
    __Pyx_GOTREF(__pyx_t_4);
    __pyx_t_1 = PyObject_RichCompare(__pyx_t_3, __pyx_t_4, Py_LT); __Pyx_XGOTREF(__pyx_t_1); if (unlikely(!__pyx_t_1)) __PYX_ERR(0, 314, __pyx_L1_error)
    __Pyx_DECREF(__pyx_t_3); __pyx_t_3 = 0;
    __Pyx_DECREF(__pyx_t_4); __pyx_t_4 = 0;
    __pyx_t_15 = __Pyx_PyObject_IsTrue(__pyx_t_1); if (unlikely(__pyx_t_15 < 0)) __PYX_ERR(0, 314, __pyx_L1_error)
    __Pyx_DECREF(__pyx_t_1); __pyx_t_1 = 0;
    if (!__pyx_t_15) {
    } else {
      __pyx_t_11 = __pyx_t_15;
      goto __pyx_L27_bool_binop_done;
    }
    __pyx_t_43 = __pyx_v_i;
    __pyx_t_10 = (*((int *) ( /* dim=0 */ (__pyx_v_avalanche_sizes.data + __pyx_t_43 * __pyx_v_avalanche_sizes.strides[0]) )));
    __pyx_t_1 = __Pyx_GetItemInt(__pyx_v_C_sizes, __pyx_t_10, int, 1, __Pyx_PyInt_From_int, 0, 0, 0); if (unlikely(!__pyx_t_1)) __PYX_ERR(0, 314, __pyx_L1_error)
    __Pyx_GOTREF(__pyx_t_1);
    __pyx_t_4 = __Pyx_PyInt_From_int(__pyx_v_min_frequency); if (unlikely(!__pyx_t_4)) __PYX_ERR(0, 314, __pyx_L1_error)
    __Pyx_GOTREF(__pyx_t_4);
    __pyx_t_3 = PyObject_RichCompare(__pyx_t_1, __pyx_t_4, Py_LT); __Pyx_XGOTREF(__pyx_t_3); if (unlikely(!__pyx_t_3)) __PYX_ERR(0, 314, __pyx_L1_error)
    __Pyx_DECREF(__pyx_t_1); __pyx_t_1 = 0;
    __Pyx_DECREF(__pyx_t_4); __pyx_t_4 = 0;
    __pyx_t_15 = __Pyx_PyObject_IsTrue(__pyx_t_3); if (unlikely(__pyx_t_15 < 0)) __PYX_ERR(0, 314, __pyx_L1_error)
    __Pyx_DECREF(__pyx_t_3); __pyx_t_3 = 0;
    __pyx_t_11 = __pyx_t_15;
    __pyx_L27_bool_binop_done:;
    if (__pyx_t_11) {
/* … */
    }
  }
```

```
+315:             index_list.append(i)
```

```
      __pyx_t_3 = __Pyx_PyInt_From_int(__pyx_v_i); if (unlikely(!__pyx_t_3)) __PYX_ERR(0, 315, __pyx_L1_error)
      __Pyx_GOTREF(__pyx_t_3);
      __pyx_t_17 = __Pyx_PyList_Append(__pyx_v_index_list, __pyx_t_3); if (unlikely(__pyx_t_17 == ((int)-1))) __PYX_ERR(0, 315, __pyx_L1_error)
      __Pyx_DECREF(__pyx_t_3); __pyx_t_3 = 0;
```

```
 316:
```

```
+317:     cdef int[:] avalanche_lengths_filtered = np.delete(avalanche_lengths, index_list)
```

```
  __pyx_t_4 = __Pyx_GetModuleGlobalName(__pyx_n_s_np); if (unlikely(!__pyx_t_4)) __PYX_ERR(0, 317, __pyx_L1_error)
  __Pyx_GOTREF(__pyx_t_4);
  __pyx_t_1 = __Pyx_PyObject_GetAttrStr(__pyx_t_4, __pyx_n_s_delete); if (unlikely(!__pyx_t_1)) __PYX_ERR(0, 317, __pyx_L1_error)
  __Pyx_GOTREF(__pyx_t_1);
  __Pyx_DECREF(__pyx_t_4); __pyx_t_4 = 0;
  __pyx_t_4 = __pyx_memoryview_fromslice(__pyx_v_avalanche_lengths, 1, (PyObject *(*)(char *)) __pyx_memview_get_int, (int (*)(char *, PyObject *)) __pyx_memview_set_int, 0);; if (unlikely(!__pyx_t_4)) __PYX_ERR(0, 317, __pyx_L1_error)
  __Pyx_GOTREF(__pyx_t_4);
  __pyx_t_2 = NULL;
  __pyx_t_6 = 0;
  if (CYTHON_UNPACK_METHODS && unlikely(PyMethod_Check(__pyx_t_1))) {
    __pyx_t_2 = PyMethod_GET_SELF(__pyx_t_1);
    if (likely(__pyx_t_2)) {
      PyObject* function = PyMethod_GET_FUNCTION(__pyx_t_1);
      __Pyx_INCREF(__pyx_t_2);
      __Pyx_INCREF(function);
      __Pyx_DECREF_SET(__pyx_t_1, function);
      __pyx_t_6 = 1;
    }
  }
  #if CYTHON_FAST_PYCALL
  if (PyFunction_Check(__pyx_t_1)) {
    PyObject *__pyx_temp[3] = {__pyx_t_2, __pyx_t_4, __pyx_v_index_list};
    __pyx_t_3 = __Pyx_PyFunction_FastCall(__pyx_t_1, __pyx_temp+1-__pyx_t_6, 2+__pyx_t_6); if (unlikely(!__pyx_t_3)) __PYX_ERR(0, 317, __pyx_L1_error)
    __Pyx_XDECREF(__pyx_t_2); __pyx_t_2 = 0;
    __Pyx_GOTREF(__pyx_t_3);
    __Pyx_DECREF(__pyx_t_4); __pyx_t_4 = 0;
  } else
  #endif
  #if CYTHON_FAST_PYCCALL
  if (__Pyx_PyFastCFunction_Check(__pyx_t_1)) {
    PyObject *__pyx_temp[3] = {__pyx_t_2, __pyx_t_4, __pyx_v_index_list};
    __pyx_t_3 = __Pyx_PyCFunction_FastCall(__pyx_t_1, __pyx_temp+1-__pyx_t_6, 2+__pyx_t_6); if (unlikely(!__pyx_t_3)) __PYX_ERR(0, 317, __pyx_L1_error)
    __Pyx_XDECREF(__pyx_t_2); __pyx_t_2 = 0;
    __Pyx_GOTREF(__pyx_t_3);
    __Pyx_DECREF(__pyx_t_4); __pyx_t_4 = 0;
  } else
  #endif
  {
    __pyx_t_36 = PyTuple_New(2+__pyx_t_6); if (unlikely(!__pyx_t_36)) __PYX_ERR(0, 317, __pyx_L1_error)
    __Pyx_GOTREF(__pyx_t_36);
    if (__pyx_t_2) {
      __Pyx_GIVEREF(__pyx_t_2); PyTuple_SET_ITEM(__pyx_t_36, 0, __pyx_t_2); __pyx_t_2 = NULL;
    }
    __Pyx_GIVEREF(__pyx_t_4);
    PyTuple_SET_ITEM(__pyx_t_36, 0+__pyx_t_6, __pyx_t_4);
    __Pyx_INCREF(__pyx_v_index_list);
    __Pyx_GIVEREF(__pyx_v_index_list);
    PyTuple_SET_ITEM(__pyx_t_36, 1+__pyx_t_6, __pyx_v_index_list);
    __pyx_t_4 = 0;
    __pyx_t_3 = __Pyx_PyObject_Call(__pyx_t_1, __pyx_t_36, NULL); if (unlikely(!__pyx_t_3)) __PYX_ERR(0, 317, __pyx_L1_error)
    __Pyx_GOTREF(__pyx_t_3);
    __Pyx_DECREF(__pyx_t_36); __pyx_t_36 = 0;
  }
  __Pyx_DECREF(__pyx_t_1); __pyx_t_1 = 0;
  __pyx_t_5 = __Pyx_PyObject_to_MemoryviewSlice_ds_int(__pyx_t_3, PyBUF_WRITABLE); if (unlikely(!__pyx_t_5.memview)) __PYX_ERR(0, 317, __pyx_L1_error)
  __Pyx_DECREF(__pyx_t_3); __pyx_t_3 = 0;
  __pyx_v_avalanche_lengths_filtered = __pyx_t_5;
  __pyx_t_5.memview = NULL;
  __pyx_t_5.data = NULL;
```

```
+318:     cdef int[:] avalanche_sizes_filtered = np.delete(avalanche_sizes, index_list)
```

```
  __pyx_t_1 = __Pyx_GetModuleGlobalName(__pyx_n_s_np); if (unlikely(!__pyx_t_1)) __PYX_ERR(0, 318, __pyx_L1_error)
  __Pyx_GOTREF(__pyx_t_1);
  __pyx_t_36 = __Pyx_PyObject_GetAttrStr(__pyx_t_1, __pyx_n_s_delete); if (unlikely(!__pyx_t_36)) __PYX_ERR(0, 318, __pyx_L1_error)
  __Pyx_GOTREF(__pyx_t_36);
  __Pyx_DECREF(__pyx_t_1); __pyx_t_1 = 0;
  __pyx_t_1 = __pyx_memoryview_fromslice(__pyx_v_avalanche_sizes, 1, (PyObject *(*)(char *)) __pyx_memview_get_int, (int (*)(char *, PyObject *)) __pyx_memview_set_int, 0);; if (unlikely(!__pyx_t_1)) __PYX_ERR(0, 318, __pyx_L1_error)
  __Pyx_GOTREF(__pyx_t_1);
  __pyx_t_4 = NULL;
  __pyx_t_6 = 0;
  if (CYTHON_UNPACK_METHODS && unlikely(PyMethod_Check(__pyx_t_36))) {
    __pyx_t_4 = PyMethod_GET_SELF(__pyx_t_36);
    if (likely(__pyx_t_4)) {
      PyObject* function = PyMethod_GET_FUNCTION(__pyx_t_36);
      __Pyx_INCREF(__pyx_t_4);
      __Pyx_INCREF(function);
      __Pyx_DECREF_SET(__pyx_t_36, function);
      __pyx_t_6 = 1;
    }
  }
  #if CYTHON_FAST_PYCALL
  if (PyFunction_Check(__pyx_t_36)) {
    PyObject *__pyx_temp[3] = {__pyx_t_4, __pyx_t_1, __pyx_v_index_list};
    __pyx_t_3 = __Pyx_PyFunction_FastCall(__pyx_t_36, __pyx_temp+1-__pyx_t_6, 2+__pyx_t_6); if (unlikely(!__pyx_t_3)) __PYX_ERR(0, 318, __pyx_L1_error)
    __Pyx_XDECREF(__pyx_t_4); __pyx_t_4 = 0;
    __Pyx_GOTREF(__pyx_t_3);
    __Pyx_DECREF(__pyx_t_1); __pyx_t_1 = 0;
  } else
  #endif
  #if CYTHON_FAST_PYCCALL
  if (__Pyx_PyFastCFunction_Check(__pyx_t_36)) {
    PyObject *__pyx_temp[3] = {__pyx_t_4, __pyx_t_1, __pyx_v_index_list};
    __pyx_t_3 = __Pyx_PyCFunction_FastCall(__pyx_t_36, __pyx_temp+1-__pyx_t_6, 2+__pyx_t_6); if (unlikely(!__pyx_t_3)) __PYX_ERR(0, 318, __pyx_L1_error)
    __Pyx_XDECREF(__pyx_t_4); __pyx_t_4 = 0;
    __Pyx_GOTREF(__pyx_t_3);
    __Pyx_DECREF(__pyx_t_1); __pyx_t_1 = 0;
  } else
  #endif
  {
    __pyx_t_2 = PyTuple_New(2+__pyx_t_6); if (unlikely(!__pyx_t_2)) __PYX_ERR(0, 318, __pyx_L1_error)
    __Pyx_GOTREF(__pyx_t_2);
    if (__pyx_t_4) {
      __Pyx_GIVEREF(__pyx_t_4); PyTuple_SET_ITEM(__pyx_t_2, 0, __pyx_t_4); __pyx_t_4 = NULL;
    }
    __Pyx_GIVEREF(__pyx_t_1);
    PyTuple_SET_ITEM(__pyx_t_2, 0+__pyx_t_6, __pyx_t_1);
    __Pyx_INCREF(__pyx_v_index_list);
    __Pyx_GIVEREF(__pyx_v_index_list);
    PyTuple_SET_ITEM(__pyx_t_2, 1+__pyx_t_6, __pyx_v_index_list);
    __pyx_t_1 = 0;
    __pyx_t_3 = __Pyx_PyObject_Call(__pyx_t_36, __pyx_t_2, NULL); if (unlikely(!__pyx_t_3)) __PYX_ERR(0, 318, __pyx_L1_error)
    __Pyx_GOTREF(__pyx_t_3);
    __Pyx_DECREF(__pyx_t_2); __pyx_t_2 = 0;
  }
  __Pyx_DECREF(__pyx_t_36); __pyx_t_36 = 0;
  __pyx_t_5 = __Pyx_PyObject_to_MemoryviewSlice_ds_int(__pyx_t_3, PyBUF_WRITABLE); if (unlikely(!__pyx_t_5.memview)) __PYX_ERR(0, 318, __pyx_L1_error)
  __Pyx_DECREF(__pyx_t_3); __pyx_t_3 = 0;
  __pyx_v_avalanche_sizes_filtered = __pyx_t_5;
  __pyx_t_5.memview = NULL;
  __pyx_t_5.data = NULL;
```

```
+319:     cdef object[:] avalanche_shapes_filtered = np.delete(avalanche_shapes, index_list)
```

```
  __pyx_t_36 = __Pyx_GetModuleGlobalName(__pyx_n_s_np); if (unlikely(!__pyx_t_36)) __PYX_ERR(0, 319, __pyx_L1_error)
  __Pyx_GOTREF(__pyx_t_36);
  __pyx_t_2 = __Pyx_PyObject_GetAttrStr(__pyx_t_36, __pyx_n_s_delete); if (unlikely(!__pyx_t_2)) __PYX_ERR(0, 319, __pyx_L1_error)
  __Pyx_GOTREF(__pyx_t_2);
  __Pyx_DECREF(__pyx_t_36); __pyx_t_36 = 0;
  __pyx_t_36 = __pyx_memoryview_fromslice(__pyx_v_avalanche_shapes, 1, (PyObject *(*)(char *)) __pyx_memview_get_object, (int (*)(char *, PyObject *)) __pyx_memview_set_object, 1);; if (unlikely(!__pyx_t_36)) __PYX_ERR(0, 319, __pyx_L1_error)
  __Pyx_GOTREF(__pyx_t_36);
  __pyx_t_1 = NULL;
  __pyx_t_6 = 0;
  if (CYTHON_UNPACK_METHODS && unlikely(PyMethod_Check(__pyx_t_2))) {
    __pyx_t_1 = PyMethod_GET_SELF(__pyx_t_2);
    if (likely(__pyx_t_1)) {
      PyObject* function = PyMethod_GET_FUNCTION(__pyx_t_2);
      __Pyx_INCREF(__pyx_t_1);
      __Pyx_INCREF(function);
      __Pyx_DECREF_SET(__pyx_t_2, function);
      __pyx_t_6 = 1;
    }
  }
  #if CYTHON_FAST_PYCALL
  if (PyFunction_Check(__pyx_t_2)) {
    PyObject *__pyx_temp[3] = {__pyx_t_1, __pyx_t_36, __pyx_v_index_list};
    __pyx_t_3 = __Pyx_PyFunction_FastCall(__pyx_t_2, __pyx_temp+1-__pyx_t_6, 2+__pyx_t_6); if (unlikely(!__pyx_t_3)) __PYX_ERR(0, 319, __pyx_L1_error)
    __Pyx_XDECREF(__pyx_t_1); __pyx_t_1 = 0;
    __Pyx_GOTREF(__pyx_t_3);
    __Pyx_DECREF(__pyx_t_36); __pyx_t_36 = 0;
  } else
  #endif
  #if CYTHON_FAST_PYCCALL
  if (__Pyx_PyFastCFunction_Check(__pyx_t_2)) {
    PyObject *__pyx_temp[3] = {__pyx_t_1, __pyx_t_36, __pyx_v_index_list};
    __pyx_t_3 = __Pyx_PyCFunction_FastCall(__pyx_t_2, __pyx_temp+1-__pyx_t_6, 2+__pyx_t_6); if (unlikely(!__pyx_t_3)) __PYX_ERR(0, 319, __pyx_L1_error)
    __Pyx_XDECREF(__pyx_t_1); __pyx_t_1 = 0;
    __Pyx_GOTREF(__pyx_t_3);
    __Pyx_DECREF(__pyx_t_36); __pyx_t_36 = 0;
  } else
  #endif
  {
    __pyx_t_4 = PyTuple_New(2+__pyx_t_6); if (unlikely(!__pyx_t_4)) __PYX_ERR(0, 319, __pyx_L1_error)
    __Pyx_GOTREF(__pyx_t_4);
    if (__pyx_t_1) {
      __Pyx_GIVEREF(__pyx_t_1); PyTuple_SET_ITEM(__pyx_t_4, 0, __pyx_t_1); __pyx_t_1 = NULL;
    }
    __Pyx_GIVEREF(__pyx_t_36);
    PyTuple_SET_ITEM(__pyx_t_4, 0+__pyx_t_6, __pyx_t_36);
    __Pyx_INCREF(__pyx_v_index_list);
    __Pyx_GIVEREF(__pyx_v_index_list);
    PyTuple_SET_ITEM(__pyx_t_4, 1+__pyx_t_6, __pyx_v_index_list);
    __pyx_t_36 = 0;
    __pyx_t_3 = __Pyx_PyObject_Call(__pyx_t_2, __pyx_t_4, NULL); if (unlikely(!__pyx_t_3)) __PYX_ERR(0, 319, __pyx_L1_error)
    __Pyx_GOTREF(__pyx_t_3);
    __Pyx_DECREF(__pyx_t_4); __pyx_t_4 = 0;
  }
  __Pyx_DECREF(__pyx_t_2); __pyx_t_2 = 0;
  __pyx_t_26 = __Pyx_PyObject_to_MemoryviewSlice_ds_object(__pyx_t_3, PyBUF_WRITABLE); if (unlikely(!__pyx_t_26.memview)) __PYX_ERR(0, 319, __pyx_L1_error)
  __Pyx_DECREF(__pyx_t_3); __pyx_t_3 = 0;
  __pyx_v_avalanche_shapes_filtered = __pyx_t_26;
  __pyx_t_26.memview = NULL;
  __pyx_t_26.data = NULL;
```

```
+320:     cdef object[:] avalanche_array_filtered = np.delete(avalanche_array, index_list)
```

```
  __pyx_t_2 = __Pyx_GetModuleGlobalName(__pyx_n_s_np); if (unlikely(!__pyx_t_2)) __PYX_ERR(0, 320, __pyx_L1_error)
  __Pyx_GOTREF(__pyx_t_2);
  __pyx_t_4 = __Pyx_PyObject_GetAttrStr(__pyx_t_2, __pyx_n_s_delete); if (unlikely(!__pyx_t_4)) __PYX_ERR(0, 320, __pyx_L1_error)
  __Pyx_GOTREF(__pyx_t_4);
  __Pyx_DECREF(__pyx_t_2); __pyx_t_2 = 0;
  __pyx_t_2 = __pyx_memoryview_fromslice(__pyx_v_avalanche_array, 1, (PyObject *(*)(char *)) __pyx_memview_get_object, (int (*)(char *, PyObject *)) __pyx_memview_set_object, 1);; if (unlikely(!__pyx_t_2)) __PYX_ERR(0, 320, __pyx_L1_error)
  __Pyx_GOTREF(__pyx_t_2);
  __pyx_t_36 = NULL;
  __pyx_t_6 = 0;
  if (CYTHON_UNPACK_METHODS && unlikely(PyMethod_Check(__pyx_t_4))) {
    __pyx_t_36 = PyMethod_GET_SELF(__pyx_t_4);
    if (likely(__pyx_t_36)) {
      PyObject* function = PyMethod_GET_FUNCTION(__pyx_t_4);
      __Pyx_INCREF(__pyx_t_36);
      __Pyx_INCREF(function);
      __Pyx_DECREF_SET(__pyx_t_4, function);
      __pyx_t_6 = 1;
    }
  }
  #if CYTHON_FAST_PYCALL
  if (PyFunction_Check(__pyx_t_4)) {
    PyObject *__pyx_temp[3] = {__pyx_t_36, __pyx_t_2, __pyx_v_index_list};
    __pyx_t_3 = __Pyx_PyFunction_FastCall(__pyx_t_4, __pyx_temp+1-__pyx_t_6, 2+__pyx_t_6); if (unlikely(!__pyx_t_3)) __PYX_ERR(0, 320, __pyx_L1_error)
    __Pyx_XDECREF(__pyx_t_36); __pyx_t_36 = 0;
    __Pyx_GOTREF(__pyx_t_3);
    __Pyx_DECREF(__pyx_t_2); __pyx_t_2 = 0;
  } else
  #endif
  #if CYTHON_FAST_PYCCALL
  if (__Pyx_PyFastCFunction_Check(__pyx_t_4)) {
    PyObject *__pyx_temp[3] = {__pyx_t_36, __pyx_t_2, __pyx_v_index_list};
    __pyx_t_3 = __Pyx_PyCFunction_FastCall(__pyx_t_4, __pyx_temp+1-__pyx_t_6, 2+__pyx_t_6); if (unlikely(!__pyx_t_3)) __PYX_ERR(0, 320, __pyx_L1_error)
    __Pyx_XDECREF(__pyx_t_36); __pyx_t_36 = 0;
    __Pyx_GOTREF(__pyx_t_3);
    __Pyx_DECREF(__pyx_t_2); __pyx_t_2 = 0;
  } else
  #endif
  {
    __pyx_t_1 = PyTuple_New(2+__pyx_t_6); if (unlikely(!__pyx_t_1)) __PYX_ERR(0, 320, __pyx_L1_error)
    __Pyx_GOTREF(__pyx_t_1);
    if (__pyx_t_36) {
      __Pyx_GIVEREF(__pyx_t_36); PyTuple_SET_ITEM(__pyx_t_1, 0, __pyx_t_36); __pyx_t_36 = NULL;
    }
    __Pyx_GIVEREF(__pyx_t_2);
    PyTuple_SET_ITEM(__pyx_t_1, 0+__pyx_t_6, __pyx_t_2);
    __Pyx_INCREF(__pyx_v_index_list);
    __Pyx_GIVEREF(__pyx_v_index_list);
    PyTuple_SET_ITEM(__pyx_t_1, 1+__pyx_t_6, __pyx_v_index_list);
    __pyx_t_2 = 0;
    __pyx_t_3 = __Pyx_PyObject_Call(__pyx_t_4, __pyx_t_1, NULL); if (unlikely(!__pyx_t_3)) __PYX_ERR(0, 320, __pyx_L1_error)
    __Pyx_GOTREF(__pyx_t_3);
    __Pyx_DECREF(__pyx_t_1); __pyx_t_1 = 0;
  }
  __Pyx_DECREF(__pyx_t_4); __pyx_t_4 = 0;
  __pyx_t_26 = __Pyx_PyObject_to_MemoryviewSlice_ds_object(__pyx_t_3, PyBUF_WRITABLE); if (unlikely(!__pyx_t_26.memview)) __PYX_ERR(0, 320, __pyx_L1_error)
  __Pyx_DECREF(__pyx_t_3); __pyx_t_3 = 0;
  __pyx_v_avalanche_array_filtered = __pyx_t_26;
  __pyx_t_26.memview = NULL;
  __pyx_t_26.data = NULL;
```

```
 321:
```

```
+322:     cdef dict channel_avalanches = {}
```

```
  __pyx_t_3 = __Pyx_PyDict_NewPresized(0); if (unlikely(!__pyx_t_3)) __PYX_ERR(0, 322, __pyx_L1_error)
  __Pyx_GOTREF(__pyx_t_3);
  __pyx_v_channel_avalanches = ((PyObject*)__pyx_t_3);
  __pyx_t_3 = 0;
```

```
+323:     for i in range(N0):
```

```
  __pyx_t_6 = __pyx_v_N0;
  __pyx_t_7 = __pyx_t_6;
  for (__pyx_t_8 = 0; __pyx_t_8 < __pyx_t_7; __pyx_t_8+=1) {
    __pyx_v_i = __pyx_t_8;
```

```
+324:         channel_avalanches[i] = 0
```

```
    __pyx_t_3 = __Pyx_PyInt_From_int(__pyx_v_i); if (unlikely(!__pyx_t_3)) __PYX_ERR(0, 324, __pyx_L1_error)
    __Pyx_GOTREF(__pyx_t_3);
    if (unlikely(PyDict_SetItem(__pyx_v_channel_avalanches, __pyx_t_3, __pyx_int_0) < 0)) __PYX_ERR(0, 324, __pyx_L1_error)
    __Pyx_DECREF(__pyx_t_3); __pyx_t_3 = 0;
  }
```

```
 325:
```

```
+326:     for i in range(avalanche_lengths_filtered.shape[0]):
```

```
  __pyx_t_20 = (__pyx_v_avalanche_lengths_filtered.shape[0]);
  __pyx_t_21 = __pyx_t_20;
  for (__pyx_t_6 = 0; __pyx_t_6 < __pyx_t_21; __pyx_t_6+=1) {
    __pyx_v_i = __pyx_t_6;
```

```
+327:         a = avalanche_array[i]
```

```
    __pyx_t_44 = __pyx_v_i;
    __pyx_t_3 = (PyObject *) *((PyObject * *) ( /* dim=0 */ (__pyx_v_avalanche_array.data + __pyx_t_44 * __pyx_v_avalanche_array.strides[0]) ));
    __Pyx_INCREF((PyObject*)__pyx_t_3);
    __pyx_t_9 = __Pyx_PyObject_to_MemoryviewSlice_dsds_int(__pyx_t_3, PyBUF_WRITABLE); if (unlikely(!__pyx_t_9.memview)) __PYX_ERR(0, 327, __pyx_L1_error)
    __Pyx_DECREF(__pyx_t_3); __pyx_t_3 = 0;
    __PYX_XDEC_MEMVIEW(&__pyx_v_a, 1);
    __pyx_v_a = __pyx_t_9;
    __pyx_t_9.memview = NULL;
    __pyx_t_9.data = NULL;
```

```
+328:         a0 = a.shape[0]
```

```
    __pyx_v_a0 = (__pyx_v_a.shape[0]);
```

```
 329:
```

```
+330:         for j in range(a0):
```

```
    __pyx_t_7 = __pyx_v_a0;
    __pyx_t_8 = __pyx_t_7;
    for (__pyx_t_10 = 0; __pyx_t_10 < __pyx_t_8; __pyx_t_10+=1) {
      __pyx_v_j = __pyx_t_10;
```

```
+331:             if sum(a[j]) >= 1:
```

```
      __pyx_t_5.data = __pyx_v_a.data;
      __pyx_t_5.memview = __pyx_v_a.memview;
      __PYX_INC_MEMVIEW(&__pyx_t_5, 0);
      {
    Py_ssize_t __pyx_tmp_idx = __pyx_v_j;
    Py_ssize_t __pyx_tmp_shape = __pyx_v_a.shape[0];
    Py_ssize_t __pyx_tmp_stride = __pyx_v_a.strides[0];
    if (0 && (__pyx_tmp_idx < 0))
        __pyx_tmp_idx += __pyx_tmp_shape;
    if (0 && (__pyx_tmp_idx < 0 || __pyx_tmp_idx >= __pyx_tmp_shape)) {
        PyErr_SetString(PyExc_IndexError, "Index out of bounds (axis 0)");
        __PYX_ERR(0, 331, __pyx_L1_error)
    }
        __pyx_t_5.data += __pyx_tmp_idx * __pyx_tmp_stride;
}

__pyx_t_5.shape[0] = __pyx_v_a.shape[1];
__pyx_t_5.strides[0] = __pyx_v_a.strides[1];
    __pyx_t_5.suboffsets[0] = -1;

__pyx_t_3 = __pyx_memoryview_fromslice(__pyx_t_5, 1, (PyObject *(*)(char *)) __pyx_memview_get_int, (int (*)(char *, PyObject *)) __pyx_memview_set_int, 0);; if (unlikely(!__pyx_t_3)) __PYX_ERR(0, 331, __pyx_L1_error)
      __Pyx_GOTREF(__pyx_t_3);
      __PYX_XDEC_MEMVIEW(&__pyx_t_5, 1);
      __pyx_t_5.memview = NULL;
      __pyx_t_5.data = NULL;
      __pyx_t_4 = __Pyx_PyObject_CallOneArg(__pyx_builtin_sum, __pyx_t_3); if (unlikely(!__pyx_t_4)) __PYX_ERR(0, 331, __pyx_L1_error)
      __Pyx_GOTREF(__pyx_t_4);
      __Pyx_DECREF(__pyx_t_3); __pyx_t_3 = 0;
      __pyx_t_3 = PyObject_RichCompare(__pyx_t_4, __pyx_int_1, Py_GE); __Pyx_XGOTREF(__pyx_t_3); if (unlikely(!__pyx_t_3)) __PYX_ERR(0, 331, __pyx_L1_error)
      __Pyx_DECREF(__pyx_t_4); __pyx_t_4 = 0;
      __pyx_t_11 = __Pyx_PyObject_IsTrue(__pyx_t_3); if (unlikely(__pyx_t_11 < 0)) __PYX_ERR(0, 331, __pyx_L1_error)
      __Pyx_DECREF(__pyx_t_3); __pyx_t_3 = 0;
      if (__pyx_t_11) {
/* … */
      }
    }
  }
```

```
+332:                 channel_avalanches[j] += 1
```

```
        __pyx_t_3 = __Pyx_PyInt_From_int(__pyx_v_j); if (unlikely(!__pyx_t_3)) __PYX_ERR(0, 332, __pyx_L1_error)
        __Pyx_GOTREF(__pyx_t_3);
        __pyx_t_4 = __Pyx_PyDict_GetItem(__pyx_v_channel_avalanches, __pyx_t_3); if (unlikely(!__pyx_t_4)) __PYX_ERR(0, 332, __pyx_L1_error)
        __Pyx_GOTREF(__pyx_t_4);
        __pyx_t_1 = __Pyx_PyInt_AddObjC(__pyx_t_4, __pyx_int_1, 1, 1); if (unlikely(!__pyx_t_1)) __PYX_ERR(0, 332, __pyx_L1_error)
        __Pyx_GOTREF(__pyx_t_1);
        __Pyx_DECREF(__pyx_t_4); __pyx_t_4 = 0;
        if (unlikely(PyDict_SetItem(__pyx_v_channel_avalanches, __pyx_t_3, __pyx_t_1) < 0)) __PYX_ERR(0, 332, __pyx_L1_error)
        __Pyx_DECREF(__pyx_t_1); __pyx_t_1 = 0;
        __Pyx_DECREF(__pyx_t_3); __pyx_t_3 = 0;
```

```
 333:
```

```
+334:     return avalanche_sizes_filtered, avalanche_lengths_filtered, avalanche_shapes_filtered, channel_avalanches,
```

```
  __Pyx_XDECREF(__pyx_r);
  __pyx_t_3 = __pyx_memoryview_fromslice(__pyx_v_avalanche_sizes_filtered, 1, (PyObject *(*)(char *)) __pyx_memview_get_int, (int (*)(char *, PyObject *)) __pyx_memview_set_int, 0);; if (unlikely(!__pyx_t_3)) __PYX_ERR(0, 334, __pyx_L1_error)
  __Pyx_GOTREF(__pyx_t_3);
  __pyx_t_1 = __pyx_memoryview_fromslice(__pyx_v_avalanche_lengths_filtered, 1, (PyObject *(*)(char *)) __pyx_memview_get_int, (int (*)(char *, PyObject *)) __pyx_memview_set_int, 0);; if (unlikely(!__pyx_t_1)) __PYX_ERR(0, 334, __pyx_L1_error)
  __Pyx_GOTREF(__pyx_t_1);
  __pyx_t_4 = __pyx_memoryview_fromslice(__pyx_v_avalanche_shapes_filtered, 1, (PyObject *(*)(char *)) __pyx_memview_get_object, (int (*)(char *, PyObject *)) __pyx_memview_set_object, 1);; if (unlikely(!__pyx_t_4)) __PYX_ERR(0, 334, __pyx_L1_error)
  __Pyx_GOTREF(__pyx_t_4);
  __pyx_t_2 = PyTuple_New(4); if (unlikely(!__pyx_t_2)) __PYX_ERR(0, 334, __pyx_L1_error)
  __Pyx_GOTREF(__pyx_t_2);
  __Pyx_GIVEREF(__pyx_t_3);
  PyTuple_SET_ITEM(__pyx_t_2, 0, __pyx_t_3);
  __Pyx_GIVEREF(__pyx_t_1);
  PyTuple_SET_ITEM(__pyx_t_2, 1, __pyx_t_1);
  __Pyx_GIVEREF(__pyx_t_4);
  PyTuple_SET_ITEM(__pyx_t_2, 2, __pyx_t_4);
  __Pyx_INCREF(__pyx_v_channel_avalanches);
  __Pyx_GIVEREF(__pyx_v_channel_avalanches);
  PyTuple_SET_ITEM(__pyx_t_2, 3, __pyx_v_channel_avalanches);
  __pyx_t_3 = 0;
  __pyx_t_1 = 0;
  __pyx_t_4 = 0;
  __pyx_r = __pyx_t_2;
  __pyx_t_2 = 0;
  goto __pyx_L0;
```

```
 335:
```

```
 336:
```

```
 337: @cython.wraparound(False)
```

```
 338: @cython.boundscheck(False)
```

```
 339: @cython.cdivision(True)
```

```
+340: def CCDF(int[:] X, bint plot = False, str xlab = "X", str ylab = "Probability", str title = "CCDF"):
```

```
/* Python wrapper */
static PyObject *__pyx_pw_10avalanches_11CCDF(PyObject *__pyx_self, PyObject *__pyx_args, PyObject *__pyx_kwds); /*proto*/
static PyMethodDef __pyx_mdef_10avalanches_11CCDF = {"CCDF", (PyCFunction)__pyx_pw_10avalanches_11CCDF, METH_VARARGS|METH_KEYWORDS, 0};
static PyObject *__pyx_pw_10avalanches_11CCDF(PyObject *__pyx_self, PyObject *__pyx_args, PyObject *__pyx_kwds) {
  __Pyx_memviewslice __pyx_v_X = { 0, 0, { 0 }, { 0 }, { 0 } };
  int __pyx_v_plot;
  PyObject *__pyx_v_xlab = 0;
  PyObject *__pyx_v_ylab = 0;
  PyObject *__pyx_v_title = 0;
  PyObject *__pyx_r = 0;
  __Pyx_RefNannyDeclarations
  __Pyx_RefNannySetupContext("CCDF (wrapper)", 0);
  {
    static PyObject **__pyx_pyargnames[] = {&__pyx_n_s_X,&__pyx_n_s_plot,&__pyx_n_s_xlab,&__pyx_n_s_ylab,&__pyx_n_s_title,0};
    PyObject* values[5] = {0,0,0,0,0};
    values[2] = ((PyObject*)__pyx_n_s_X);
    values[3] = ((PyObject*)__pyx_n_s_Probability);
    values[4] = ((PyObject*)__pyx_n_s_CCDF);
    if (unlikely(__pyx_kwds)) {
      Py_ssize_t kw_args;
      const Py_ssize_t pos_args = PyTuple_GET_SIZE(__pyx_args);
      switch (pos_args) {
        case  5: values[4] = PyTuple_GET_ITEM(__pyx_args, 4);
        CYTHON_FALLTHROUGH;
        case  4: values[3] = PyTuple_GET_ITEM(__pyx_args, 3);
        CYTHON_FALLTHROUGH;
        case  3: values[2] = PyTuple_GET_ITEM(__pyx_args, 2);
        CYTHON_FALLTHROUGH;
        case  2: values[1] = PyTuple_GET_ITEM(__pyx_args, 1);
        CYTHON_FALLTHROUGH;
        case  1: values[0] = PyTuple_GET_ITEM(__pyx_args, 0);
        CYTHON_FALLTHROUGH;
        case  0: break;
        default: goto __pyx_L5_argtuple_error;
      }
      kw_args = PyDict_Size(__pyx_kwds);
      switch (pos_args) {
        case  0:
        if (likely((values[0] = __Pyx_PyDict_GetItemStr(__pyx_kwds, __pyx_n_s_X)) != 0)) kw_args--;
        else goto __pyx_L5_argtuple_error;
        CYTHON_FALLTHROUGH;
        case  1:
        if (kw_args > 0) {
          PyObject* value = __Pyx_PyDict_GetItemStr(__pyx_kwds, __pyx_n_s_plot);
          if (value) { values[1] = value; kw_args--; }
        }
        CYTHON_FALLTHROUGH;
        case  2:
        if (kw_args > 0) {
          PyObject* value = __Pyx_PyDict_GetItemStr(__pyx_kwds, __pyx_n_s_xlab);
          if (value) { values[2] = value; kw_args--; }
        }
        CYTHON_FALLTHROUGH;
        case  3:
        if (kw_args > 0) {
          PyObject* value = __Pyx_PyDict_GetItemStr(__pyx_kwds, __pyx_n_s_ylab);
          if (value) { values[3] = value; kw_args--; }
        }
        CYTHON_FALLTHROUGH;
        case  4:
        if (kw_args > 0) {
          PyObject* value = __Pyx_PyDict_GetItemStr(__pyx_kwds, __pyx_n_s_title);
          if (value) { values[4] = value; kw_args--; }
        }
      }
      if (unlikely(kw_args > 0)) {
        if (unlikely(__Pyx_ParseOptionalKeywords(__pyx_kwds, __pyx_pyargnames, 0, values, pos_args, "CCDF") < 0)) __PYX_ERR(0, 340, __pyx_L3_error)
      }
    } else {
      switch (PyTuple_GET_SIZE(__pyx_args)) {
        case  5: values[4] = PyTuple_GET_ITEM(__pyx_args, 4);
        CYTHON_FALLTHROUGH;
        case  4: values[3] = PyTuple_GET_ITEM(__pyx_args, 3);
        CYTHON_FALLTHROUGH;
        case  3: values[2] = PyTuple_GET_ITEM(__pyx_args, 2);
        CYTHON_FALLTHROUGH;
        case  2: values[1] = PyTuple_GET_ITEM(__pyx_args, 1);
        CYTHON_FALLTHROUGH;
        case  1: values[0] = PyTuple_GET_ITEM(__pyx_args, 0);
        break;
        default: goto __pyx_L5_argtuple_error;
      }
    }
    __pyx_v_X = __Pyx_PyObject_to_MemoryviewSlice_ds_int(values[0], PyBUF_WRITABLE); if (unlikely(!__pyx_v_X.memview)) __PYX_ERR(0, 340, __pyx_L3_error)
    if (values[1]) {
      __pyx_v_plot = __Pyx_PyObject_IsTrue(values[1]); if (unlikely((__pyx_v_plot == (int)-1) && PyErr_Occurred())) __PYX_ERR(0, 340, __pyx_L3_error)
    } else {
      __pyx_v_plot = ((int)0);
    }
    __pyx_v_xlab = ((PyObject*)values[2]);
    __pyx_v_ylab = ((PyObject*)values[3]);
    __pyx_v_title = ((PyObject*)values[4]);
  }
  goto __pyx_L4_argument_unpacking_done;
  __pyx_L5_argtuple_error:;
  __Pyx_RaiseArgtupleInvalid("CCDF", 0, 1, 5, PyTuple_GET_SIZE(__pyx_args)); __PYX_ERR(0, 340, __pyx_L3_error)
  __pyx_L3_error:;
  __Pyx_AddTraceback("avalanches.CCDF", __pyx_clineno, __pyx_lineno, __pyx_filename);
  __Pyx_RefNannyFinishContext();
  return NULL;
  __pyx_L4_argument_unpacking_done:;
  if (unlikely(!__Pyx_ArgTypeTest(((PyObject *)__pyx_v_xlab), (&PyString_Type), 1, "xlab", 1))) __PYX_ERR(0, 340, __pyx_L1_error)
  if (unlikely(!__Pyx_ArgTypeTest(((PyObject *)__pyx_v_ylab), (&PyString_Type), 1, "ylab", 1))) __PYX_ERR(0, 340, __pyx_L1_error)
  if (unlikely(!__Pyx_ArgTypeTest(((PyObject *)__pyx_v_title), (&PyString_Type), 1, "title", 1))) __PYX_ERR(0, 340, __pyx_L1_error)
  __pyx_r = __pyx_pf_10avalanches_10CCDF(__pyx_self, __pyx_v_X, __pyx_v_plot, __pyx_v_xlab, __pyx_v_ylab, __pyx_v_title);

  /* function exit code */
  goto __pyx_L0;
  __pyx_L1_error:;
  __pyx_r = NULL;
  __pyx_L0:;
  __Pyx_RefNannyFinishContext();
  return __pyx_r;
}

static PyObject *__pyx_pf_10avalanches_10CCDF(CYTHON_UNUSED PyObject *__pyx_self, __Pyx_memviewslice __pyx_v_X, int __pyx_v_plot, PyObject *__pyx_v_xlab, PyObject *__pyx_v_ylab, PyObject *__pyx_v_title) {
  PyObject *__pyx_v_C = NULL;
  int __pyx_v_m;
  int __pyx_v_Nc;
  __Pyx_memviewslice __pyx_v_x = { 0, 0, { 0 }, { 0 }, { 0 } };
  __Pyx_memviewslice __pyx_v_y = { 0, 0, { 0 }, { 0 }, { 0 } };
  int __pyx_v_i;
  double __pyx_v_y_max;
  __Pyx_memviewslice __pyx_v_ccdf = { 0, 0, { 0 }, { 0 }, { 0 } };
  PyObject *__pyx_r = NULL;
  __Pyx_RefNannyDeclarations
  __Pyx_RefNannySetupContext("CCDF", 0);
/* … */
  /* function exit code */
  __pyx_L1_error:;
  __Pyx_XDECREF(__pyx_t_1);
  __Pyx_XDECREF(__pyx_t_2);
  __Pyx_XDECREF(__pyx_t_3);
  __Pyx_XDECREF(__pyx_t_4);
  __Pyx_XDECREF(__pyx_t_5);
  __PYX_XDEC_MEMVIEW(&__pyx_t_9, 1);
  __PYX_XDEC_MEMVIEW(&__pyx_t_10, 1);
  __Pyx_XDECREF(__pyx_t_22);
  __Pyx_AddTraceback("avalanches.CCDF", __pyx_clineno, __pyx_lineno, __pyx_filename);
  __pyx_r = NULL;
  __pyx_L0:;
  __Pyx_XDECREF(__pyx_v_C);
  __PYX_XDEC_MEMVIEW(&__pyx_v_x, 1);
  __PYX_XDEC_MEMVIEW(&__pyx_v_y, 1);
  __PYX_XDEC_MEMVIEW(&__pyx_v_ccdf, 1);
  __PYX_XDEC_MEMVIEW(&__pyx_v_X, 1);
  __Pyx_XGIVEREF(__pyx_r);
  __Pyx_RefNannyFinishContext();
  return __pyx_r;
}
/* … */
  __pyx_tuple__49 = PyTuple_Pack(13, __pyx_n_s_X, __pyx_n_s_plot, __pyx_n_s_xlab, __pyx_n_s_ylab, __pyx_n_s_title, __pyx_n_s_C, __pyx_n_s_m, __pyx_n_s_Nc, __pyx_n_s_x, __pyx_n_s_y, __pyx_n_s_i, __pyx_n_s_y_max, __pyx_n_s_ccdf); if (unlikely(!__pyx_tuple__49)) __PYX_ERR(0, 340, __pyx_L1_error)
  __Pyx_GOTREF(__pyx_tuple__49);
  __Pyx_GIVEREF(__pyx_tuple__49);
/* … */
  __pyx_t_2 = PyCFunction_NewEx(&__pyx_mdef_10avalanches_11CCDF, NULL, __pyx_n_s_avalanches); if (unlikely(!__pyx_t_2)) __PYX_ERR(0, 340, __pyx_L1_error)
  __Pyx_GOTREF(__pyx_t_2);
  if (PyDict_SetItem(__pyx_d, __pyx_n_s_CCDF, __pyx_t_2) < 0) __PYX_ERR(0, 340, __pyx_L1_error)
  __Pyx_DECREF(__pyx_t_2); __pyx_t_2 = 0;
  __pyx_codeobj__50 = (PyObject*)__Pyx_PyCode_New(5, 0, 13, 0, CO_OPTIMIZED|CO_NEWLOCALS, __pyx_empty_bytes, __pyx_empty_tuple, __pyx_empty_tuple, __pyx_tuple__49, __pyx_empty_tuple, __pyx_empty_tuple, __pyx_kp_s_avalanches_pyx, __pyx_n_s_CCDF, 340, __pyx_empty_bytes); if (unlikely(!__pyx_codeobj__50)) __PYX_ERR(0, 340, __pyx_L1_error)
```

```
+341:     C = Counter(X)
```

```
  __pyx_t_2 = __Pyx_GetModuleGlobalName(__pyx_n_s_Counter); if (unlikely(!__pyx_t_2)) __PYX_ERR(0, 341, __pyx_L1_error)
  __Pyx_GOTREF(__pyx_t_2);
  __pyx_t_3 = __pyx_memoryview_fromslice(__pyx_v_X, 1, (PyObject *(*)(char *)) __pyx_memview_get_int, (int (*)(char *, PyObject *)) __pyx_memview_set_int, 0);; if (unlikely(!__pyx_t_3)) __PYX_ERR(0, 341, __pyx_L1_error)
  __Pyx_GOTREF(__pyx_t_3);
  __pyx_t_4 = NULL;
  if (CYTHON_UNPACK_METHODS && unlikely(PyMethod_Check(__pyx_t_2))) {
    __pyx_t_4 = PyMethod_GET_SELF(__pyx_t_2);
    if (likely(__pyx_t_4)) {
      PyObject* function = PyMethod_GET_FUNCTION(__pyx_t_2);
      __Pyx_INCREF(__pyx_t_4);
      __Pyx_INCREF(function);
      __Pyx_DECREF_SET(__pyx_t_2, function);
    }
  }
  if (!__pyx_t_4) {
    __pyx_t_1 = __Pyx_PyObject_CallOneArg(__pyx_t_2, __pyx_t_3); if (unlikely(!__pyx_t_1)) __PYX_ERR(0, 341, __pyx_L1_error)
    __Pyx_DECREF(__pyx_t_3); __pyx_t_3 = 0;
    __Pyx_GOTREF(__pyx_t_1);
  } else {
    #if CYTHON_FAST_PYCALL
    if (PyFunction_Check(__pyx_t_2)) {
      PyObject *__pyx_temp[2] = {__pyx_t_4, __pyx_t_3};
      __pyx_t_1 = __Pyx_PyFunction_FastCall(__pyx_t_2, __pyx_temp+1-1, 1+1); if (unlikely(!__pyx_t_1)) __PYX_ERR(0, 341, __pyx_L1_error)
      __Pyx_XDECREF(__pyx_t_4); __pyx_t_4 = 0;
      __Pyx_GOTREF(__pyx_t_1);
      __Pyx_DECREF(__pyx_t_3); __pyx_t_3 = 0;
    } else
    #endif
    #if CYTHON_FAST_PYCCALL
    if (__Pyx_PyFastCFunction_Check(__pyx_t_2)) {
      PyObject *__pyx_temp[2] = {__pyx_t_4, __pyx_t_3};
      __pyx_t_1 = __Pyx_PyCFunction_FastCall(__pyx_t_2, __pyx_temp+1-1, 1+1); if (unlikely(!__pyx_t_1)) __PYX_ERR(0, 341, __pyx_L1_error)
      __Pyx_XDECREF(__pyx_t_4); __pyx_t_4 = 0;
      __Pyx_GOTREF(__pyx_t_1);
      __Pyx_DECREF(__pyx_t_3); __pyx_t_3 = 0;
    } else
    #endif
    {
      __pyx_t_5 = PyTuple_New(1+1); if (unlikely(!__pyx_t_5)) __PYX_ERR(0, 341, __pyx_L1_error)
      __Pyx_GOTREF(__pyx_t_5);
      __Pyx_GIVEREF(__pyx_t_4); PyTuple_SET_ITEM(__pyx_t_5, 0, __pyx_t_4); __pyx_t_4 = NULL;
      __Pyx_GIVEREF(__pyx_t_3);
      PyTuple_SET_ITEM(__pyx_t_5, 0+1, __pyx_t_3);
      __pyx_t_3 = 0;
      __pyx_t_1 = __Pyx_PyObject_Call(__pyx_t_2, __pyx_t_5, NULL); if (unlikely(!__pyx_t_1)) __PYX_ERR(0, 341, __pyx_L1_error)
      __Pyx_GOTREF(__pyx_t_1);
      __Pyx_DECREF(__pyx_t_5); __pyx_t_5 = 0;
    }
  }
  __Pyx_DECREF(__pyx_t_2); __pyx_t_2 = 0;
  __pyx_v_C = __pyx_t_1;
  __pyx_t_1 = 0;
```

```
+342:     cdef int m = min(X)
```

```
  __pyx_t_1 = __pyx_memoryview_fromslice(__pyx_v_X, 1, (PyObject *(*)(char *)) __pyx_memview_get_int, (int (*)(char *, PyObject *)) __pyx_memview_set_int, 0);; if (unlikely(!__pyx_t_1)) __PYX_ERR(0, 342, __pyx_L1_error)
  __Pyx_GOTREF(__pyx_t_1);
  __pyx_t_2 = __Pyx_PyObject_CallOneArg(__pyx_builtin_min, __pyx_t_1); if (unlikely(!__pyx_t_2)) __PYX_ERR(0, 342, __pyx_L1_error)
  __Pyx_GOTREF(__pyx_t_2);
  __Pyx_DECREF(__pyx_t_1); __pyx_t_1 = 0;
  __pyx_t_6 = __Pyx_PyInt_As_int(__pyx_t_2); if (unlikely((__pyx_t_6 == (int)-1) && PyErr_Occurred())) __PYX_ERR(0, 342, __pyx_L1_error)
  __Pyx_DECREF(__pyx_t_2); __pyx_t_2 = 0;
  __pyx_v_m = __pyx_t_6;
```

```
+343:     cdef int Nc = len(C)
```

```
  __pyx_t_7 = PyObject_Length(__pyx_v_C); if (unlikely(__pyx_t_7 == ((Py_ssize_t)-1))) __PYX_ERR(0, 343, __pyx_L1_error)
  __pyx_v_Nc = __pyx_t_7;
```

```
 344:
```

```
+345:     cdef long[:] x = np.array(sorted(C))
```

```
  __pyx_t_1 = __Pyx_GetModuleGlobalName(__pyx_n_s_np); if (unlikely(!__pyx_t_1)) __PYX_ERR(0, 345, __pyx_L1_error)
  __Pyx_GOTREF(__pyx_t_1);
  __pyx_t_5 = __Pyx_PyObject_GetAttrStr(__pyx_t_1, __pyx_n_s_array); if (unlikely(!__pyx_t_5)) __PYX_ERR(0, 345, __pyx_L1_error)
  __Pyx_GOTREF(__pyx_t_5);
  __Pyx_DECREF(__pyx_t_1); __pyx_t_1 = 0;
  __pyx_t_3 = PySequence_List(__pyx_v_C); if (unlikely(!__pyx_t_3)) __PYX_ERR(0, 345, __pyx_L1_error)
  __Pyx_GOTREF(__pyx_t_3);
  __pyx_t_1 = ((PyObject*)__pyx_t_3);
  __pyx_t_3 = 0;
  __pyx_t_8 = PyList_Sort(__pyx_t_1); if (unlikely(__pyx_t_8 == ((int)-1))) __PYX_ERR(0, 345, __pyx_L1_error)
  __pyx_t_3 = NULL;
  if (CYTHON_UNPACK_METHODS && unlikely(PyMethod_Check(__pyx_t_5))) {
    __pyx_t_3 = PyMethod_GET_SELF(__pyx_t_5);
    if (likely(__pyx_t_3)) {
      PyObject* function = PyMethod_GET_FUNCTION(__pyx_t_5);
      __Pyx_INCREF(__pyx_t_3);
      __Pyx_INCREF(function);
      __Pyx_DECREF_SET(__pyx_t_5, function);
    }
  }
  if (!__pyx_t_3) {
    __pyx_t_2 = __Pyx_PyObject_CallOneArg(__pyx_t_5, __pyx_t_1); if (unlikely(!__pyx_t_2)) __PYX_ERR(0, 345, __pyx_L1_error)
    __Pyx_DECREF(__pyx_t_1); __pyx_t_1 = 0;
    __Pyx_GOTREF(__pyx_t_2);
  } else {
    #if CYTHON_FAST_PYCALL
    if (PyFunction_Check(__pyx_t_5)) {
      PyObject *__pyx_temp[2] = {__pyx_t_3, __pyx_t_1};
      __pyx_t_2 = __Pyx_PyFunction_FastCall(__pyx_t_5, __pyx_temp+1-1, 1+1); if (unlikely(!__pyx_t_2)) __PYX_ERR(0, 345, __pyx_L1_error)
      __Pyx_XDECREF(__pyx_t_3); __pyx_t_3 = 0;
      __Pyx_GOTREF(__pyx_t_2);
      __Pyx_DECREF(__pyx_t_1); __pyx_t_1 = 0;
    } else
    #endif
    #if CYTHON_FAST_PYCCALL
    if (__Pyx_PyFastCFunction_Check(__pyx_t_5)) {
      PyObject *__pyx_temp[2] = {__pyx_t_3, __pyx_t_1};
      __pyx_t_2 = __Pyx_PyCFunction_FastCall(__pyx_t_5, __pyx_temp+1-1, 1+1); if (unlikely(!__pyx_t_2)) __PYX_ERR(0, 345, __pyx_L1_error)
      __Pyx_XDECREF(__pyx_t_3); __pyx_t_3 = 0;
      __Pyx_GOTREF(__pyx_t_2);
      __Pyx_DECREF(__pyx_t_1); __pyx_t_1 = 0;
    } else
    #endif
    {
      __pyx_t_4 = PyTuple_New(1+1); if (unlikely(!__pyx_t_4)) __PYX_ERR(0, 345, __pyx_L1_error)
      __Pyx_GOTREF(__pyx_t_4);
      __Pyx_GIVEREF(__pyx_t_3); PyTuple_SET_ITEM(__pyx_t_4, 0, __pyx_t_3); __pyx_t_3 = NULL;
      __Pyx_GIVEREF(__pyx_t_1);
      PyTuple_SET_ITEM(__pyx_t_4, 0+1, __pyx_t_1);
      __pyx_t_1 = 0;
      __pyx_t_2 = __Pyx_PyObject_Call(__pyx_t_5, __pyx_t_4, NULL); if (unlikely(!__pyx_t_2)) __PYX_ERR(0, 345, __pyx_L1_error)
      __Pyx_GOTREF(__pyx_t_2);
      __Pyx_DECREF(__pyx_t_4); __pyx_t_4 = 0;
    }
  }
  __Pyx_DECREF(__pyx_t_5); __pyx_t_5 = 0;
  __pyx_t_9 = __Pyx_PyObject_to_MemoryviewSlice_ds_long(__pyx_t_2, PyBUF_WRITABLE); if (unlikely(!__pyx_t_9.memview)) __PYX_ERR(0, 345, __pyx_L1_error)
  __Pyx_DECREF(__pyx_t_2); __pyx_t_2 = 0;
  __pyx_v_x = __pyx_t_9;
  __pyx_t_9.memview = NULL;
  __pyx_t_9.data = NULL;
```

```
+346:     cdef double[:] y = np.zeros(Nc)
```

```
  __pyx_t_5 = __Pyx_GetModuleGlobalName(__pyx_n_s_np); if (unlikely(!__pyx_t_5)) __PYX_ERR(0, 346, __pyx_L1_error)
  __Pyx_GOTREF(__pyx_t_5);
  __pyx_t_4 = __Pyx_PyObject_GetAttrStr(__pyx_t_5, __pyx_n_s_zeros); if (unlikely(!__pyx_t_4)) __PYX_ERR(0, 346, __pyx_L1_error)
  __Pyx_GOTREF(__pyx_t_4);
  __Pyx_DECREF(__pyx_t_5); __pyx_t_5 = 0;
  __pyx_t_5 = __Pyx_PyInt_From_int(__pyx_v_Nc); if (unlikely(!__pyx_t_5)) __PYX_ERR(0, 346, __pyx_L1_error)
  __Pyx_GOTREF(__pyx_t_5);
  __pyx_t_1 = NULL;
  if (CYTHON_UNPACK_METHODS && unlikely(PyMethod_Check(__pyx_t_4))) {
    __pyx_t_1 = PyMethod_GET_SELF(__pyx_t_4);
    if (likely(__pyx_t_1)) {
      PyObject* function = PyMethod_GET_FUNCTION(__pyx_t_4);
      __Pyx_INCREF(__pyx_t_1);
      __Pyx_INCREF(function);
      __Pyx_DECREF_SET(__pyx_t_4, function);
    }
  }
  if (!__pyx_t_1) {
    __pyx_t_2 = __Pyx_PyObject_CallOneArg(__pyx_t_4, __pyx_t_5); if (unlikely(!__pyx_t_2)) __PYX_ERR(0, 346, __pyx_L1_error)
    __Pyx_DECREF(__pyx_t_5); __pyx_t_5 = 0;
    __Pyx_GOTREF(__pyx_t_2);
  } else {
    #if CYTHON_FAST_PYCALL
    if (PyFunction_Check(__pyx_t_4)) {
      PyObject *__pyx_temp[2] = {__pyx_t_1, __pyx_t_5};
      __pyx_t_2 = __Pyx_PyFunction_FastCall(__pyx_t_4, __pyx_temp+1-1, 1+1); if (unlikely(!__pyx_t_2)) __PYX_ERR(0, 346, __pyx_L1_error)
      __Pyx_XDECREF(__pyx_t_1); __pyx_t_1 = 0;
      __Pyx_GOTREF(__pyx_t_2);
      __Pyx_DECREF(__pyx_t_5); __pyx_t_5 = 0;
    } else
    #endif
    #if CYTHON_FAST_PYCCALL
    if (__Pyx_PyFastCFunction_Check(__pyx_t_4)) {
      PyObject *__pyx_temp[2] = {__pyx_t_1, __pyx_t_5};
      __pyx_t_2 = __Pyx_PyCFunction_FastCall(__pyx_t_4, __pyx_temp+1-1, 1+1); if (unlikely(!__pyx_t_2)) __PYX_ERR(0, 346, __pyx_L1_error)
      __Pyx_XDECREF(__pyx_t_1); __pyx_t_1 = 0;
      __Pyx_GOTREF(__pyx_t_2);
      __Pyx_DECREF(__pyx_t_5); __pyx_t_5 = 0;
    } else
    #endif
    {
      __pyx_t_3 = PyTuple_New(1+1); if (unlikely(!__pyx_t_3)) __PYX_ERR(0, 346, __pyx_L1_error)
      __Pyx_GOTREF(__pyx_t_3);
      __Pyx_GIVEREF(__pyx_t_1); PyTuple_SET_ITEM(__pyx_t_3, 0, __pyx_t_1); __pyx_t_1 = NULL;
      __Pyx_GIVEREF(__pyx_t_5);
      PyTuple_SET_ITEM(__pyx_t_3, 0+1, __pyx_t_5);
      __pyx_t_5 = 0;
      __pyx_t_2 = __Pyx_PyObject_Call(__pyx_t_4, __pyx_t_3, NULL); if (unlikely(!__pyx_t_2)) __PYX_ERR(0, 346, __pyx_L1_error)
      __Pyx_GOTREF(__pyx_t_2);
      __Pyx_DECREF(__pyx_t_3); __pyx_t_3 = 0;
    }
  }
  __Pyx_DECREF(__pyx_t_4); __pyx_t_4 = 0;
  __pyx_t_10 = __Pyx_PyObject_to_MemoryviewSlice_ds_double(__pyx_t_2, PyBUF_WRITABLE); if (unlikely(!__pyx_t_10.memview)) __PYX_ERR(0, 346, __pyx_L1_error)
  __Pyx_DECREF(__pyx_t_2); __pyx_t_2 = 0;
  __pyx_v_y = __pyx_t_10;
  __pyx_t_10.memview = NULL;
  __pyx_t_10.data = NULL;
```

```
 347:
```

```
 348:     cdef int i
```

```
+349:     for i in range(m,Nc):
```

```
  __pyx_t_6 = __pyx_v_Nc;
  __pyx_t_11 = __pyx_t_6;
  for (__pyx_t_12 = __pyx_v_m; __pyx_t_12 < __pyx_t_11; __pyx_t_12+=1) {
    __pyx_v_i = __pyx_t_12;
```

```
+350:         y[i] = C[x[i]]
```

```
    __pyx_t_13 = __pyx_v_i;
    __pyx_t_14 = (*((long *) ( /* dim=0 */ (__pyx_v_x.data + __pyx_t_13 * __pyx_v_x.strides[0]) )));
    __pyx_t_2 = __Pyx_GetItemInt(__pyx_v_C, __pyx_t_14, long, 1, __Pyx_PyInt_From_long, 0, 0, 0); if (unlikely(!__pyx_t_2)) __PYX_ERR(0, 350, __pyx_L1_error)
    __Pyx_GOTREF(__pyx_t_2);
    __pyx_t_15 = __pyx_PyFloat_AsDouble(__pyx_t_2); if (unlikely((__pyx_t_15 == (double)-1) && PyErr_Occurred())) __PYX_ERR(0, 350, __pyx_L1_error)
    __Pyx_DECREF(__pyx_t_2); __pyx_t_2 = 0;
    __pyx_t_16 = __pyx_v_i;
    *((double *) ( /* dim=0 */ (__pyx_v_y.data + __pyx_t_16 * __pyx_v_y.strides[0]) )) = __pyx_t_15;
  }
```

```
 351:
```

```
+352:     cdef double y_max = sum(y)
```

```
  __pyx_t_2 = __pyx_memoryview_fromslice(__pyx_v_y, 1, (PyObject *(*)(char *)) __pyx_memview_get_double, (int (*)(char *, PyObject *)) __pyx_memview_set_double, 0);; if (unlikely(!__pyx_t_2)) __PYX_ERR(0, 352, __pyx_L1_error)
  __Pyx_GOTREF(__pyx_t_2);
  __pyx_t_4 = __Pyx_PyObject_CallOneArg(__pyx_builtin_sum, __pyx_t_2); if (unlikely(!__pyx_t_4)) __PYX_ERR(0, 352, __pyx_L1_error)
  __Pyx_GOTREF(__pyx_t_4);
  __Pyx_DECREF(__pyx_t_2); __pyx_t_2 = 0;
  __pyx_t_15 = __pyx_PyFloat_AsDouble(__pyx_t_4); if (unlikely((__pyx_t_15 == (double)-1) && PyErr_Occurred())) __PYX_ERR(0, 352, __pyx_L1_error)
  __Pyx_DECREF(__pyx_t_4); __pyx_t_4 = 0;
  __pyx_v_y_max = __pyx_t_15;
```

```
 353:
```

```
+354:     for i in range(m,Nc):
```

```
  __pyx_t_6 = __pyx_v_Nc;
  __pyx_t_11 = __pyx_t_6;
  for (__pyx_t_12 = __pyx_v_m; __pyx_t_12 < __pyx_t_11; __pyx_t_12+=1) {
    __pyx_v_i = __pyx_t_12;
```

```
+355:         y[i] = y[i] / y_max
```

```
    __pyx_t_17 = __pyx_v_i;
    __pyx_t_18 = __pyx_v_i;
    *((double *) ( /* dim=0 */ (__pyx_v_y.data + __pyx_t_18 * __pyx_v_y.strides[0]) )) = ((*((double *) ( /* dim=0 */ (__pyx_v_y.data + __pyx_t_17 * __pyx_v_y.strides[0]) ))) / __pyx_v_y_max);
  }
```

```
 356:
```

```
+357:     cdef double[:] ccdf = np.zeros(Nc)
```

```
  __pyx_t_2 = __Pyx_GetModuleGlobalName(__pyx_n_s_np); if (unlikely(!__pyx_t_2)) __PYX_ERR(0, 357, __pyx_L1_error)
  __Pyx_GOTREF(__pyx_t_2);
  __pyx_t_3 = __Pyx_PyObject_GetAttrStr(__pyx_t_2, __pyx_n_s_zeros); if (unlikely(!__pyx_t_3)) __PYX_ERR(0, 357, __pyx_L1_error)
  __Pyx_GOTREF(__pyx_t_3);
  __Pyx_DECREF(__pyx_t_2); __pyx_t_2 = 0;
  __pyx_t_2 = __Pyx_PyInt_From_int(__pyx_v_Nc); if (unlikely(!__pyx_t_2)) __PYX_ERR(0, 357, __pyx_L1_error)
  __Pyx_GOTREF(__pyx_t_2);
  __pyx_t_5 = NULL;
  if (CYTHON_UNPACK_METHODS && unlikely(PyMethod_Check(__pyx_t_3))) {
    __pyx_t_5 = PyMethod_GET_SELF(__pyx_t_3);
    if (likely(__pyx_t_5)) {
      PyObject* function = PyMethod_GET_FUNCTION(__pyx_t_3);
      __Pyx_INCREF(__pyx_t_5);
      __Pyx_INCREF(function);
      __Pyx_DECREF_SET(__pyx_t_3, function);
    }
  }
  if (!__pyx_t_5) {
    __pyx_t_4 = __Pyx_PyObject_CallOneArg(__pyx_t_3, __pyx_t_2); if (unlikely(!__pyx_t_4)) __PYX_ERR(0, 357, __pyx_L1_error)
    __Pyx_DECREF(__pyx_t_2); __pyx_t_2 = 0;
    __Pyx_GOTREF(__pyx_t_4);
  } else {
    #if CYTHON_FAST_PYCALL
    if (PyFunction_Check(__pyx_t_3)) {
      PyObject *__pyx_temp[2] = {__pyx_t_5, __pyx_t_2};
      __pyx_t_4 = __Pyx_PyFunction_FastCall(__pyx_t_3, __pyx_temp+1-1, 1+1); if (unlikely(!__pyx_t_4)) __PYX_ERR(0, 357, __pyx_L1_error)
      __Pyx_XDECREF(__pyx_t_5); __pyx_t_5 = 0;
      __Pyx_GOTREF(__pyx_t_4);
      __Pyx_DECREF(__pyx_t_2); __pyx_t_2 = 0;
    } else
    #endif
    #if CYTHON_FAST_PYCCALL
    if (__Pyx_PyFastCFunction_Check(__pyx_t_3)) {
      PyObject *__pyx_temp[2] = {__pyx_t_5, __pyx_t_2};
      __pyx_t_4 = __Pyx_PyCFunction_FastCall(__pyx_t_3, __pyx_temp+1-1, 1+1); if (unlikely(!__pyx_t_4)) __PYX_ERR(0, 357, __pyx_L1_error)
      __Pyx_XDECREF(__pyx_t_5); __pyx_t_5 = 0;
      __Pyx_GOTREF(__pyx_t_4);
      __Pyx_DECREF(__pyx_t_2); __pyx_t_2 = 0;
    } else
    #endif
    {
      __pyx_t_1 = PyTuple_New(1+1); if (unlikely(!__pyx_t_1)) __PYX_ERR(0, 357, __pyx_L1_error)
      __Pyx_GOTREF(__pyx_t_1);
      __Pyx_GIVEREF(__pyx_t_5); PyTuple_SET_ITEM(__pyx_t_1, 0, __pyx_t_5); __pyx_t_5 = NULL;
      __Pyx_GIVEREF(__pyx_t_2);
      PyTuple_SET_ITEM(__pyx_t_1, 0+1, __pyx_t_2);
      __pyx_t_2 = 0;
      __pyx_t_4 = __Pyx_PyObject_Call(__pyx_t_3, __pyx_t_1, NULL); if (unlikely(!__pyx_t_4)) __PYX_ERR(0, 357, __pyx_L1_error)
      __Pyx_GOTREF(__pyx_t_4);
      __Pyx_DECREF(__pyx_t_1); __pyx_t_1 = 0;
    }
  }
  __Pyx_DECREF(__pyx_t_3); __pyx_t_3 = 0;
  __pyx_t_10 = __Pyx_PyObject_to_MemoryviewSlice_ds_double(__pyx_t_4, PyBUF_WRITABLE); if (unlikely(!__pyx_t_10.memview)) __PYX_ERR(0, 357, __pyx_L1_error)
  __Pyx_DECREF(__pyx_t_4); __pyx_t_4 = 0;
  __pyx_v_ccdf = __pyx_t_10;
  __pyx_t_10.memview = NULL;
  __pyx_t_10.data = NULL;
```

```
 358:
```

```
+359:     for i in range(m,Nc):
```

```
  __pyx_t_6 = __pyx_v_Nc;
  __pyx_t_11 = __pyx_t_6;
  for (__pyx_t_12 = __pyx_v_m; __pyx_t_12 < __pyx_t_11; __pyx_t_12+=1) {
    __pyx_v_i = __pyx_t_12;
```

```
+360:         ccdf[i] = np.sum(y[i:])
```

```
    __pyx_t_3 = __Pyx_GetModuleGlobalName(__pyx_n_s_np); if (unlikely(!__pyx_t_3)) __PYX_ERR(0, 360, __pyx_L1_error)
    __Pyx_GOTREF(__pyx_t_3);
    __pyx_t_1 = __Pyx_PyObject_GetAttrStr(__pyx_t_3, __pyx_n_s_sum); if (unlikely(!__pyx_t_1)) __PYX_ERR(0, 360, __pyx_L1_error)
    __Pyx_GOTREF(__pyx_t_1);
    __Pyx_DECREF(__pyx_t_3); __pyx_t_3 = 0;
    __pyx_t_10.data = __pyx_v_y.data;
    __pyx_t_10.memview = __pyx_v_y.memview;
    __PYX_INC_MEMVIEW(&__pyx_t_10, 0);
    __pyx_t_19 = -1;
    if (unlikely(__pyx_memoryview_slice_memviewslice(
    &__pyx_t_10,
    __pyx_v_y.shape[0], __pyx_v_y.strides[0], __pyx_v_y.suboffsets[0],
    0,
    0,
    &__pyx_t_19,
    __pyx_v_i,
    0,
    0,
    1,
    0,
    0,
    1) < 0))
{
    __PYX_ERR(0, 360, __pyx_L1_error)
}

__pyx_t_3 = __pyx_memoryview_fromslice(__pyx_t_10, 1, (PyObject *(*)(char *)) __pyx_memview_get_double, (int (*)(char *, PyObject *)) __pyx_memview_set_double, 0);; if (unlikely(!__pyx_t_3)) __PYX_ERR(0, 360, __pyx_L1_error)
    __Pyx_GOTREF(__pyx_t_3);
    __PYX_XDEC_MEMVIEW(&__pyx_t_10, 1);
    __pyx_t_10.memview = NULL;
    __pyx_t_10.data = NULL;
    __pyx_t_2 = NULL;
    if (CYTHON_UNPACK_METHODS && unlikely(PyMethod_Check(__pyx_t_1))) {
      __pyx_t_2 = PyMethod_GET_SELF(__pyx_t_1);
      if (likely(__pyx_t_2)) {
        PyObject* function = PyMethod_GET_FUNCTION(__pyx_t_1);
        __Pyx_INCREF(__pyx_t_2);
        __Pyx_INCREF(function);
        __Pyx_DECREF_SET(__pyx_t_1, function);
      }
    }
    if (!__pyx_t_2) {
      __pyx_t_4 = __Pyx_PyObject_CallOneArg(__pyx_t_1, __pyx_t_3); if (unlikely(!__pyx_t_4)) __PYX_ERR(0, 360, __pyx_L1_error)
      __Pyx_DECREF(__pyx_t_3); __pyx_t_3 = 0;
      __Pyx_GOTREF(__pyx_t_4);
    } else {
      #if CYTHON_FAST_PYCALL
      if (PyFunction_Check(__pyx_t_1)) {
        PyObject *__pyx_temp[2] = {__pyx_t_2, __pyx_t_3};
        __pyx_t_4 = __Pyx_PyFunction_FastCall(__pyx_t_1, __pyx_temp+1-1, 1+1); if (unlikely(!__pyx_t_4)) __PYX_ERR(0, 360, __pyx_L1_error)
        __Pyx_XDECREF(__pyx_t_2); __pyx_t_2 = 0;
        __Pyx_GOTREF(__pyx_t_4);
        __Pyx_DECREF(__pyx_t_3); __pyx_t_3 = 0;
      } else
      #endif
      #if CYTHON_FAST_PYCCALL
      if (__Pyx_PyFastCFunction_Check(__pyx_t_1)) {
        PyObject *__pyx_temp[2] = {__pyx_t_2, __pyx_t_3};
        __pyx_t_4 = __Pyx_PyCFunction_FastCall(__pyx_t_1, __pyx_temp+1-1, 1+1); if (unlikely(!__pyx_t_4)) __PYX_ERR(0, 360, __pyx_L1_error)
        __Pyx_XDECREF(__pyx_t_2); __pyx_t_2 = 0;
        __Pyx_GOTREF(__pyx_t_4);
        __Pyx_DECREF(__pyx_t_3); __pyx_t_3 = 0;
      } else
      #endif
      {
        __pyx_t_5 = PyTuple_New(1+1); if (unlikely(!__pyx_t_5)) __PYX_ERR(0, 360, __pyx_L1_error)
        __Pyx_GOTREF(__pyx_t_5);
        __Pyx_GIVEREF(__pyx_t_2); PyTuple_SET_ITEM(__pyx_t_5, 0, __pyx_t_2); __pyx_t_2 = NULL;
        __Pyx_GIVEREF(__pyx_t_3);
        PyTuple_SET_ITEM(__pyx_t_5, 0+1, __pyx_t_3);
        __pyx_t_3 = 0;
        __pyx_t_4 = __Pyx_PyObject_Call(__pyx_t_1, __pyx_t_5, NULL); if (unlikely(!__pyx_t_4)) __PYX_ERR(0, 360, __pyx_L1_error)
        __Pyx_GOTREF(__pyx_t_4);
        __Pyx_DECREF(__pyx_t_5); __pyx_t_5 = 0;
      }
    }
    __Pyx_DECREF(__pyx_t_1); __pyx_t_1 = 0;
    __pyx_t_15 = __pyx_PyFloat_AsDouble(__pyx_t_4); if (unlikely((__pyx_t_15 == (double)-1) && PyErr_Occurred())) __PYX_ERR(0, 360, __pyx_L1_error)
    __Pyx_DECREF(__pyx_t_4); __pyx_t_4 = 0;
    __pyx_t_20 = __pyx_v_i;
    *((double *) ( /* dim=0 */ (__pyx_v_ccdf.data + __pyx_t_20 * __pyx_v_ccdf.strides[0]) )) = __pyx_t_15;
  }
```

```
 361:
```

```
+362:     if plot == True:
```

```
  __pyx_t_21 = ((__pyx_v_plot == 1) != 0);
  if (__pyx_t_21) {
/* … */
  }
```

```
+363:         plt.subplots()
```

```
    __pyx_t_1 = __Pyx_GetModuleGlobalName(__pyx_n_s_plt); if (unlikely(!__pyx_t_1)) __PYX_ERR(0, 363, __pyx_L1_error)
    __Pyx_GOTREF(__pyx_t_1);
    __pyx_t_5 = __Pyx_PyObject_GetAttrStr(__pyx_t_1, __pyx_n_s_subplots); if (unlikely(!__pyx_t_5)) __PYX_ERR(0, 363, __pyx_L1_error)
    __Pyx_GOTREF(__pyx_t_5);
    __Pyx_DECREF(__pyx_t_1); __pyx_t_1 = 0;
    __pyx_t_1 = NULL;
    if (CYTHON_UNPACK_METHODS && unlikely(PyMethod_Check(__pyx_t_5))) {
      __pyx_t_1 = PyMethod_GET_SELF(__pyx_t_5);
      if (likely(__pyx_t_1)) {
        PyObject* function = PyMethod_GET_FUNCTION(__pyx_t_5);
        __Pyx_INCREF(__pyx_t_1);
        __Pyx_INCREF(function);
        __Pyx_DECREF_SET(__pyx_t_5, function);
      }
    }
    if (__pyx_t_1) {
      __pyx_t_4 = __Pyx_PyObject_CallOneArg(__pyx_t_5, __pyx_t_1); if (unlikely(!__pyx_t_4)) __PYX_ERR(0, 363, __pyx_L1_error)
      __Pyx_DECREF(__pyx_t_1); __pyx_t_1 = 0;
    } else {
      __pyx_t_4 = __Pyx_PyObject_CallNoArg(__pyx_t_5); if (unlikely(!__pyx_t_4)) __PYX_ERR(0, 363, __pyx_L1_error)
    }
    __Pyx_GOTREF(__pyx_t_4);
    __Pyx_DECREF(__pyx_t_5); __pyx_t_5 = 0;
    __Pyx_DECREF(__pyx_t_4); __pyx_t_4 = 0;
```

```
+364:         plt.loglog(x, ccdf, color = "blue")
```

```
    __pyx_t_4 = __Pyx_GetModuleGlobalName(__pyx_n_s_plt); if (unlikely(!__pyx_t_4)) __PYX_ERR(0, 364, __pyx_L1_error)
    __Pyx_GOTREF(__pyx_t_4);
    __pyx_t_5 = __Pyx_PyObject_GetAttrStr(__pyx_t_4, __pyx_n_s_loglog); if (unlikely(!__pyx_t_5)) __PYX_ERR(0, 364, __pyx_L1_error)
    __Pyx_GOTREF(__pyx_t_5);
    __Pyx_DECREF(__pyx_t_4); __pyx_t_4 = 0;
    __pyx_t_4 = __pyx_memoryview_fromslice(__pyx_v_x, 1, (PyObject *(*)(char *)) __pyx_memview_get_long, (int (*)(char *, PyObject *)) __pyx_memview_set_long, 0);; if (unlikely(!__pyx_t_4)) __PYX_ERR(0, 364, __pyx_L1_error)
    __Pyx_GOTREF(__pyx_t_4);
    __pyx_t_1 = __pyx_memoryview_fromslice(__pyx_v_ccdf, 1, (PyObject *(*)(char *)) __pyx_memview_get_double, (int (*)(char *, PyObject *)) __pyx_memview_set_double, 0);; if (unlikely(!__pyx_t_1)) __PYX_ERR(0, 364, __pyx_L1_error)
    __Pyx_GOTREF(__pyx_t_1);
    __pyx_t_3 = PyTuple_New(2); if (unlikely(!__pyx_t_3)) __PYX_ERR(0, 364, __pyx_L1_error)
    __Pyx_GOTREF(__pyx_t_3);
    __Pyx_GIVEREF(__pyx_t_4);
    PyTuple_SET_ITEM(__pyx_t_3, 0, __pyx_t_4);
    __Pyx_GIVEREF(__pyx_t_1);
    PyTuple_SET_ITEM(__pyx_t_3, 1, __pyx_t_1);
    __pyx_t_4 = 0;
    __pyx_t_1 = 0;
    __pyx_t_1 = __Pyx_PyDict_NewPresized(1); if (unlikely(!__pyx_t_1)) __PYX_ERR(0, 364, __pyx_L1_error)
    __Pyx_GOTREF(__pyx_t_1);
    if (PyDict_SetItem(__pyx_t_1, __pyx_n_s_color, __pyx_n_s_blue) < 0) __PYX_ERR(0, 364, __pyx_L1_error)
    __pyx_t_4 = __Pyx_PyObject_Call(__pyx_t_5, __pyx_t_3, __pyx_t_1); if (unlikely(!__pyx_t_4)) __PYX_ERR(0, 364, __pyx_L1_error)
    __Pyx_GOTREF(__pyx_t_4);
    __Pyx_DECREF(__pyx_t_5); __pyx_t_5 = 0;
    __Pyx_DECREF(__pyx_t_3); __pyx_t_3 = 0;
    __Pyx_DECREF(__pyx_t_1); __pyx_t_1 = 0;
    __Pyx_DECREF(__pyx_t_4); __pyx_t_4 = 0;
```

```
+365:         plt.loglog(x, ccdf, "o")
```

```
    __pyx_t_1 = __Pyx_GetModuleGlobalName(__pyx_n_s_plt); if (unlikely(!__pyx_t_1)) __PYX_ERR(0, 365, __pyx_L1_error)
    __Pyx_GOTREF(__pyx_t_1);
    __pyx_t_3 = __Pyx_PyObject_GetAttrStr(__pyx_t_1, __pyx_n_s_loglog); if (unlikely(!__pyx_t_3)) __PYX_ERR(0, 365, __pyx_L1_error)
    __Pyx_GOTREF(__pyx_t_3);
    __Pyx_DECREF(__pyx_t_1); __pyx_t_1 = 0;
    __pyx_t_1 = __pyx_memoryview_fromslice(__pyx_v_x, 1, (PyObject *(*)(char *)) __pyx_memview_get_long, (int (*)(char *, PyObject *)) __pyx_memview_set_long, 0);; if (unlikely(!__pyx_t_1)) __PYX_ERR(0, 365, __pyx_L1_error)
    __Pyx_GOTREF(__pyx_t_1);
    __pyx_t_5 = __pyx_memoryview_fromslice(__pyx_v_ccdf, 1, (PyObject *(*)(char *)) __pyx_memview_get_double, (int (*)(char *, PyObject *)) __pyx_memview_set_double, 0);; if (unlikely(!__pyx_t_5)) __PYX_ERR(0, 365, __pyx_L1_error)
    __Pyx_GOTREF(__pyx_t_5);
    __pyx_t_2 = NULL;
    __pyx_t_6 = 0;
    if (CYTHON_UNPACK_METHODS && unlikely(PyMethod_Check(__pyx_t_3))) {
      __pyx_t_2 = PyMethod_GET_SELF(__pyx_t_3);
      if (likely(__pyx_t_2)) {
        PyObject* function = PyMethod_GET_FUNCTION(__pyx_t_3);
        __Pyx_INCREF(__pyx_t_2);
        __Pyx_INCREF(function);
        __Pyx_DECREF_SET(__pyx_t_3, function);
        __pyx_t_6 = 1;
      }
    }
    #if CYTHON_FAST_PYCALL
    if (PyFunction_Check(__pyx_t_3)) {
      PyObject *__pyx_temp[4] = {__pyx_t_2, __pyx_t_1, __pyx_t_5, __pyx_n_s_o};
      __pyx_t_4 = __Pyx_PyFunction_FastCall(__pyx_t_3, __pyx_temp+1-__pyx_t_6, 3+__pyx_t_6); if (unlikely(!__pyx_t_4)) __PYX_ERR(0, 365, __pyx_L1_error)
      __Pyx_XDECREF(__pyx_t_2); __pyx_t_2 = 0;
      __Pyx_GOTREF(__pyx_t_4);
      __Pyx_DECREF(__pyx_t_1); __pyx_t_1 = 0;
      __Pyx_DECREF(__pyx_t_5); __pyx_t_5 = 0;
    } else
    #endif
    #if CYTHON_FAST_PYCCALL
    if (__Pyx_PyFastCFunction_Check(__pyx_t_3)) {
      PyObject *__pyx_temp[4] = {__pyx_t_2, __pyx_t_1, __pyx_t_5, __pyx_n_s_o};
      __pyx_t_4 = __Pyx_PyCFunction_FastCall(__pyx_t_3, __pyx_temp+1-__pyx_t_6, 3+__pyx_t_6); if (unlikely(!__pyx_t_4)) __PYX_ERR(0, 365, __pyx_L1_error)
      __Pyx_XDECREF(__pyx_t_2); __pyx_t_2 = 0;
      __Pyx_GOTREF(__pyx_t_4);
      __Pyx_DECREF(__pyx_t_1); __pyx_t_1 = 0;
      __Pyx_DECREF(__pyx_t_5); __pyx_t_5 = 0;
    } else
    #endif
    {
      __pyx_t_22 = PyTuple_New(3+__pyx_t_6); if (unlikely(!__pyx_t_22)) __PYX_ERR(0, 365, __pyx_L1_error)
      __Pyx_GOTREF(__pyx_t_22);
      if (__pyx_t_2) {
        __Pyx_GIVEREF(__pyx_t_2); PyTuple_SET_ITEM(__pyx_t_22, 0, __pyx_t_2); __pyx_t_2 = NULL;
      }
      __Pyx_GIVEREF(__pyx_t_1);
      PyTuple_SET_ITEM(__pyx_t_22, 0+__pyx_t_6, __pyx_t_1);
      __Pyx_GIVEREF(__pyx_t_5);
      PyTuple_SET_ITEM(__pyx_t_22, 1+__pyx_t_6, __pyx_t_5);
      __Pyx_INCREF(__pyx_n_s_o);
      __Pyx_GIVEREF(__pyx_n_s_o);
      PyTuple_SET_ITEM(__pyx_t_22, 2+__pyx_t_6, __pyx_n_s_o);
      __pyx_t_1 = 0;
      __pyx_t_5 = 0;
      __pyx_t_4 = __Pyx_PyObject_Call(__pyx_t_3, __pyx_t_22, NULL); if (unlikely(!__pyx_t_4)) __PYX_ERR(0, 365, __pyx_L1_error)
      __Pyx_GOTREF(__pyx_t_4);
      __Pyx_DECREF(__pyx_t_22); __pyx_t_22 = 0;
    }
    __Pyx_DECREF(__pyx_t_3); __pyx_t_3 = 0;
    __Pyx_DECREF(__pyx_t_4); __pyx_t_4 = 0;
```

```
+366:         plt.xlabel(xlab)
```

```
    __pyx_t_3 = __Pyx_GetModuleGlobalName(__pyx_n_s_plt); if (unlikely(!__pyx_t_3)) __PYX_ERR(0, 366, __pyx_L1_error)
    __Pyx_GOTREF(__pyx_t_3);
    __pyx_t_22 = __Pyx_PyObject_GetAttrStr(__pyx_t_3, __pyx_n_s_xlabel); if (unlikely(!__pyx_t_22)) __PYX_ERR(0, 366, __pyx_L1_error)
    __Pyx_GOTREF(__pyx_t_22);
    __Pyx_DECREF(__pyx_t_3); __pyx_t_3 = 0;
    __pyx_t_3 = NULL;
    if (CYTHON_UNPACK_METHODS && unlikely(PyMethod_Check(__pyx_t_22))) {
      __pyx_t_3 = PyMethod_GET_SELF(__pyx_t_22);
      if (likely(__pyx_t_3)) {
        PyObject* function = PyMethod_GET_FUNCTION(__pyx_t_22);
        __Pyx_INCREF(__pyx_t_3);
        __Pyx_INCREF(function);
        __Pyx_DECREF_SET(__pyx_t_22, function);
      }
    }
    if (!__pyx_t_3) {
      __pyx_t_4 = __Pyx_PyObject_CallOneArg(__pyx_t_22, __pyx_v_xlab); if (unlikely(!__pyx_t_4)) __PYX_ERR(0, 366, __pyx_L1_error)
      __Pyx_GOTREF(__pyx_t_4);
    } else {
      #if CYTHON_FAST_PYCALL
      if (PyFunction_Check(__pyx_t_22)) {
        PyObject *__pyx_temp[2] = {__pyx_t_3, __pyx_v_xlab};
        __pyx_t_4 = __Pyx_PyFunction_FastCall(__pyx_t_22, __pyx_temp+1-1, 1+1); if (unlikely(!__pyx_t_4)) __PYX_ERR(0, 366, __pyx_L1_error)
        __Pyx_XDECREF(__pyx_t_3); __pyx_t_3 = 0;
        __Pyx_GOTREF(__pyx_t_4);
      } else
      #endif
      #if CYTHON_FAST_PYCCALL
      if (__Pyx_PyFastCFunction_Check(__pyx_t_22)) {
        PyObject *__pyx_temp[2] = {__pyx_t_3, __pyx_v_xlab};
        __pyx_t_4 = __Pyx_PyCFunction_FastCall(__pyx_t_22, __pyx_temp+1-1, 1+1); if (unlikely(!__pyx_t_4)) __PYX_ERR(0, 366, __pyx_L1_error)
        __Pyx_XDECREF(__pyx_t_3); __pyx_t_3 = 0;
        __Pyx_GOTREF(__pyx_t_4);
      } else
      #endif
      {
        __pyx_t_5 = PyTuple_New(1+1); if (unlikely(!__pyx_t_5)) __PYX_ERR(0, 366, __pyx_L1_error)
        __Pyx_GOTREF(__pyx_t_5);
        __Pyx_GIVEREF(__pyx_t_3); PyTuple_SET_ITEM(__pyx_t_5, 0, __pyx_t_3); __pyx_t_3 = NULL;
        __Pyx_INCREF(__pyx_v_xlab);
        __Pyx_GIVEREF(__pyx_v_xlab);
        PyTuple_SET_ITEM(__pyx_t_5, 0+1, __pyx_v_xlab);
        __pyx_t_4 = __Pyx_PyObject_Call(__pyx_t_22, __pyx_t_5, NULL); if (unlikely(!__pyx_t_4)) __PYX_ERR(0, 366, __pyx_L1_error)
        __Pyx_GOTREF(__pyx_t_4);
        __Pyx_DECREF(__pyx_t_5); __pyx_t_5 = 0;
      }
    }
    __Pyx_DECREF(__pyx_t_22); __pyx_t_22 = 0;
    __Pyx_DECREF(__pyx_t_4); __pyx_t_4 = 0;
```

```
+367:         plt.ylabel(ylab)
```

```
    __pyx_t_22 = __Pyx_GetModuleGlobalName(__pyx_n_s_plt); if (unlikely(!__pyx_t_22)) __PYX_ERR(0, 367, __pyx_L1_error)
    __Pyx_GOTREF(__pyx_t_22);
    __pyx_t_5 = __Pyx_PyObject_GetAttrStr(__pyx_t_22, __pyx_n_s_ylabel); if (unlikely(!__pyx_t_5)) __PYX_ERR(0, 367, __pyx_L1_error)
    __Pyx_GOTREF(__pyx_t_5);
    __Pyx_DECREF(__pyx_t_22); __pyx_t_22 = 0;
    __pyx_t_22 = NULL;
    if (CYTHON_UNPACK_METHODS && unlikely(PyMethod_Check(__pyx_t_5))) {
      __pyx_t_22 = PyMethod_GET_SELF(__pyx_t_5);
      if (likely(__pyx_t_22)) {
        PyObject* function = PyMethod_GET_FUNCTION(__pyx_t_5);
        __Pyx_INCREF(__pyx_t_22);
        __Pyx_INCREF(function);
        __Pyx_DECREF_SET(__pyx_t_5, function);
      }
    }
    if (!__pyx_t_22) {
      __pyx_t_4 = __Pyx_PyObject_CallOneArg(__pyx_t_5, __pyx_v_ylab); if (unlikely(!__pyx_t_4)) __PYX_ERR(0, 367, __pyx_L1_error)
      __Pyx_GOTREF(__pyx_t_4);
    } else {
      #if CYTHON_FAST_PYCALL
      if (PyFunction_Check(__pyx_t_5)) {
        PyObject *__pyx_temp[2] = {__pyx_t_22, __pyx_v_ylab};
        __pyx_t_4 = __Pyx_PyFunction_FastCall(__pyx_t_5, __pyx_temp+1-1, 1+1); if (unlikely(!__pyx_t_4)) __PYX_ERR(0, 367, __pyx_L1_error)
        __Pyx_XDECREF(__pyx_t_22); __pyx_t_22 = 0;
        __Pyx_GOTREF(__pyx_t_4);
      } else
      #endif
      #if CYTHON_FAST_PYCCALL
      if (__Pyx_PyFastCFunction_Check(__pyx_t_5)) {
        PyObject *__pyx_temp[2] = {__pyx_t_22, __pyx_v_ylab};
        __pyx_t_4 = __Pyx_PyCFunction_FastCall(__pyx_t_5, __pyx_temp+1-1, 1+1); if (unlikely(!__pyx_t_4)) __PYX_ERR(0, 367, __pyx_L1_error)
        __Pyx_XDECREF(__pyx_t_22); __pyx_t_22 = 0;
        __Pyx_GOTREF(__pyx_t_4);
      } else
      #endif
      {
        __pyx_t_3 = PyTuple_New(1+1); if (unlikely(!__pyx_t_3)) __PYX_ERR(0, 367, __pyx_L1_error)
        __Pyx_GOTREF(__pyx_t_3);
        __Pyx_GIVEREF(__pyx_t_22); PyTuple_SET_ITEM(__pyx_t_3, 0, __pyx_t_22); __pyx_t_22 = NULL;
        __Pyx_INCREF(__pyx_v_ylab);
        __Pyx_GIVEREF(__pyx_v_ylab);
        PyTuple_SET_ITEM(__pyx_t_3, 0+1, __pyx_v_ylab);
        __pyx_t_4 = __Pyx_PyObject_Call(__pyx_t_5, __pyx_t_3, NULL); if (unlikely(!__pyx_t_4)) __PYX_ERR(0, 367, __pyx_L1_error)
        __Pyx_GOTREF(__pyx_t_4);
        __Pyx_DECREF(__pyx_t_3); __pyx_t_3 = 0;
      }
    }
    __Pyx_DECREF(__pyx_t_5); __pyx_t_5 = 0;
    __Pyx_DECREF(__pyx_t_4); __pyx_t_4 = 0;
```

```
+368:         plt.title(title)
```

```
    __pyx_t_5 = __Pyx_GetModuleGlobalName(__pyx_n_s_plt); if (unlikely(!__pyx_t_5)) __PYX_ERR(0, 368, __pyx_L1_error)
    __Pyx_GOTREF(__pyx_t_5);
    __pyx_t_3 = __Pyx_PyObject_GetAttrStr(__pyx_t_5, __pyx_n_s_title); if (unlikely(!__pyx_t_3)) __PYX_ERR(0, 368, __pyx_L1_error)
    __Pyx_GOTREF(__pyx_t_3);
    __Pyx_DECREF(__pyx_t_5); __pyx_t_5 = 0;
    __pyx_t_5 = NULL;
    if (CYTHON_UNPACK_METHODS && unlikely(PyMethod_Check(__pyx_t_3))) {
      __pyx_t_5 = PyMethod_GET_SELF(__pyx_t_3);
      if (likely(__pyx_t_5)) {
        PyObject* function = PyMethod_GET_FUNCTION(__pyx_t_3);
        __Pyx_INCREF(__pyx_t_5);
        __Pyx_INCREF(function);
        __Pyx_DECREF_SET(__pyx_t_3, function);
      }
    }
    if (!__pyx_t_5) {
      __pyx_t_4 = __Pyx_PyObject_CallOneArg(__pyx_t_3, __pyx_v_title); if (unlikely(!__pyx_t_4)) __PYX_ERR(0, 368, __pyx_L1_error)
      __Pyx_GOTREF(__pyx_t_4);
    } else {
      #if CYTHON_FAST_PYCALL
      if (PyFunction_Check(__pyx_t_3)) {
        PyObject *__pyx_temp[2] = {__pyx_t_5, __pyx_v_title};
        __pyx_t_4 = __Pyx_PyFunction_FastCall(__pyx_t_3, __pyx_temp+1-1, 1+1); if (unlikely(!__pyx_t_4)) __PYX_ERR(0, 368, __pyx_L1_error)
        __Pyx_XDECREF(__pyx_t_5); __pyx_t_5 = 0;
        __Pyx_GOTREF(__pyx_t_4);
      } else
      #endif
      #if CYTHON_FAST_PYCCALL
      if (__Pyx_PyFastCFunction_Check(__pyx_t_3)) {
        PyObject *__pyx_temp[2] = {__pyx_t_5, __pyx_v_title};
        __pyx_t_4 = __Pyx_PyCFunction_FastCall(__pyx_t_3, __pyx_temp+1-1, 1+1); if (unlikely(!__pyx_t_4)) __PYX_ERR(0, 368, __pyx_L1_error)
        __Pyx_XDECREF(__pyx_t_5); __pyx_t_5 = 0;
        __Pyx_GOTREF(__pyx_t_4);
      } else
      #endif
      {
        __pyx_t_22 = PyTuple_New(1+1); if (unlikely(!__pyx_t_22)) __PYX_ERR(0, 368, __pyx_L1_error)
        __Pyx_GOTREF(__pyx_t_22);
        __Pyx_GIVEREF(__pyx_t_5); PyTuple_SET_ITEM(__pyx_t_22, 0, __pyx_t_5); __pyx_t_5 = NULL;
        __Pyx_INCREF(__pyx_v_title);
        __Pyx_GIVEREF(__pyx_v_title);
        PyTuple_SET_ITEM(__pyx_t_22, 0+1, __pyx_v_title);
        __pyx_t_4 = __Pyx_PyObject_Call(__pyx_t_3, __pyx_t_22, NULL); if (unlikely(!__pyx_t_4)) __PYX_ERR(0, 368, __pyx_L1_error)
        __Pyx_GOTREF(__pyx_t_4);
        __Pyx_DECREF(__pyx_t_22); __pyx_t_22 = 0;
      }
    }
    __Pyx_DECREF(__pyx_t_3); __pyx_t_3 = 0;
    __Pyx_DECREF(__pyx_t_4); __pyx_t_4 = 0;
```

```
+369:         plt.show()
```

```
    __pyx_t_3 = __Pyx_GetModuleGlobalName(__pyx_n_s_plt); if (unlikely(!__pyx_t_3)) __PYX_ERR(0, 369, __pyx_L1_error)
    __Pyx_GOTREF(__pyx_t_3);
    __pyx_t_22 = __Pyx_PyObject_GetAttrStr(__pyx_t_3, __pyx_n_s_show); if (unlikely(!__pyx_t_22)) __PYX_ERR(0, 369, __pyx_L1_error)
    __Pyx_GOTREF(__pyx_t_22);
    __Pyx_DECREF(__pyx_t_3); __pyx_t_3 = 0;
    __pyx_t_3 = NULL;
    if (CYTHON_UNPACK_METHODS && unlikely(PyMethod_Check(__pyx_t_22))) {
      __pyx_t_3 = PyMethod_GET_SELF(__pyx_t_22);
      if (likely(__pyx_t_3)) {
        PyObject* function = PyMethod_GET_FUNCTION(__pyx_t_22);
        __Pyx_INCREF(__pyx_t_3);
        __Pyx_INCREF(function);
        __Pyx_DECREF_SET(__pyx_t_22, function);
      }
    }
    if (__pyx_t_3) {
      __pyx_t_4 = __Pyx_PyObject_CallOneArg(__pyx_t_22, __pyx_t_3); if (unlikely(!__pyx_t_4)) __PYX_ERR(0, 369, __pyx_L1_error)
      __Pyx_DECREF(__pyx_t_3); __pyx_t_3 = 0;
    } else {
      __pyx_t_4 = __Pyx_PyObject_CallNoArg(__pyx_t_22); if (unlikely(!__pyx_t_4)) __PYX_ERR(0, 369, __pyx_L1_error)
    }
    __Pyx_GOTREF(__pyx_t_4);
    __Pyx_DECREF(__pyx_t_22); __pyx_t_22 = 0;
    __Pyx_DECREF(__pyx_t_4); __pyx_t_4 = 0;
```

```
 370:
```

```
+371:     return x, ccdf
```

```
  __Pyx_XDECREF(__pyx_r);
  __pyx_t_4 = __pyx_memoryview_fromslice(__pyx_v_x, 1, (PyObject *(*)(char *)) __pyx_memview_get_long, (int (*)(char *, PyObject *)) __pyx_memview_set_long, 0);; if (unlikely(!__pyx_t_4)) __PYX_ERR(0, 371, __pyx_L1_error)
  __Pyx_GOTREF(__pyx_t_4);
  __pyx_t_22 = __pyx_memoryview_fromslice(__pyx_v_ccdf, 1, (PyObject *(*)(char *)) __pyx_memview_get_double, (int (*)(char *, PyObject *)) __pyx_memview_set_double, 0);; if (unlikely(!__pyx_t_22)) __PYX_ERR(0, 371, __pyx_L1_error)
  __Pyx_GOTREF(__pyx_t_22);
  __pyx_t_3 = PyTuple_New(2); if (unlikely(!__pyx_t_3)) __PYX_ERR(0, 371, __pyx_L1_error)
  __Pyx_GOTREF(__pyx_t_3);
  __Pyx_GIVEREF(__pyx_t_4);
  PyTuple_SET_ITEM(__pyx_t_3, 0, __pyx_t_4);
  __Pyx_GIVEREF(__pyx_t_22);
  PyTuple_SET_ITEM(__pyx_t_3, 1, __pyx_t_22);
  __pyx_t_4 = 0;
  __pyx_t_22 = 0;
  __pyx_r = __pyx_t_3;
  __pyx_t_3 = 0;
  goto __pyx_L0;
```

```
 372:
```

```
 373: @cython.wraparound(False)
```

```
 374: @cython.boundscheck(False)
```

```
 375: @cython.cdivision(True)
```

```
+376: def exponent_relation(int[:] lengths, int[:] sizes, bint plot = False):
```

```
/* Python wrapper */
static PyObject *__pyx_pw_10avalanches_13exponent_relation(PyObject *__pyx_self, PyObject *__pyx_args, PyObject *__pyx_kwds); /*proto*/
static char __pyx_doc_10avalanches_12exponent_relation[] = "\n    Returns the average size for a given length. \n    ";
static PyMethodDef __pyx_mdef_10avalanches_13exponent_relation = {"exponent_relation", (PyCFunction)__pyx_pw_10avalanches_13exponent_relation, METH_VARARGS|METH_KEYWORDS, __pyx_doc_10avalanches_12exponent_relation};
static PyObject *__pyx_pw_10avalanches_13exponent_relation(PyObject *__pyx_self, PyObject *__pyx_args, PyObject *__pyx_kwds) {
  __Pyx_memviewslice __pyx_v_lengths = { 0, 0, { 0 }, { 0 }, { 0 } };
  __Pyx_memviewslice __pyx_v_sizes = { 0, 0, { 0 }, { 0 }, { 0 } };
  int __pyx_v_plot;
  PyObject *__pyx_r = 0;
  __Pyx_RefNannyDeclarations
  __Pyx_RefNannySetupContext("exponent_relation (wrapper)", 0);
  {
    static PyObject **__pyx_pyargnames[] = {&__pyx_n_s_lengths,&__pyx_n_s_sizes,&__pyx_n_s_plot,0};
    PyObject* values[3] = {0,0,0};
    if (unlikely(__pyx_kwds)) {
      Py_ssize_t kw_args;
      const Py_ssize_t pos_args = PyTuple_GET_SIZE(__pyx_args);
      switch (pos_args) {
        case  3: values[2] = PyTuple_GET_ITEM(__pyx_args, 2);
        CYTHON_FALLTHROUGH;
        case  2: values[1] = PyTuple_GET_ITEM(__pyx_args, 1);
        CYTHON_FALLTHROUGH;
        case  1: values[0] = PyTuple_GET_ITEM(__pyx_args, 0);
        CYTHON_FALLTHROUGH;
        case  0: break;
        default: goto __pyx_L5_argtuple_error;
      }
      kw_args = PyDict_Size(__pyx_kwds);
      switch (pos_args) {
        case  0:
        if (likely((values[0] = __Pyx_PyDict_GetItemStr(__pyx_kwds, __pyx_n_s_lengths)) != 0)) kw_args--;
        else goto __pyx_L5_argtuple_error;
        CYTHON_FALLTHROUGH;
        case  1:
        if (likely((values[1] = __Pyx_PyDict_GetItemStr(__pyx_kwds, __pyx_n_s_sizes)) != 0)) kw_args--;
        else {
          __Pyx_RaiseArgtupleInvalid("exponent_relation", 0, 2, 3, 1); __PYX_ERR(0, 376, __pyx_L3_error)
        }
        CYTHON_FALLTHROUGH;
        case  2:
        if (kw_args > 0) {
          PyObject* value = __Pyx_PyDict_GetItemStr(__pyx_kwds, __pyx_n_s_plot);
          if (value) { values[2] = value; kw_args--; }
        }
      }
      if (unlikely(kw_args > 0)) {
        if (unlikely(__Pyx_ParseOptionalKeywords(__pyx_kwds, __pyx_pyargnames, 0, values, pos_args, "exponent_relation") < 0)) __PYX_ERR(0, 376, __pyx_L3_error)
      }
    } else {
      switch (PyTuple_GET_SIZE(__pyx_args)) {
        case  3: values[2] = PyTuple_GET_ITEM(__pyx_args, 2);
        CYTHON_FALLTHROUGH;
        case  2: values[1] = PyTuple_GET_ITEM(__pyx_args, 1);
        values[0] = PyTuple_GET_ITEM(__pyx_args, 0);
        break;
        default: goto __pyx_L5_argtuple_error;
      }
    }
    __pyx_v_lengths = __Pyx_PyObject_to_MemoryviewSlice_ds_int(values[0], PyBUF_WRITABLE); if (unlikely(!__pyx_v_lengths.memview)) __PYX_ERR(0, 376, __pyx_L3_error)
    __pyx_v_sizes = __Pyx_PyObject_to_MemoryviewSlice_ds_int(values[1], PyBUF_WRITABLE); if (unlikely(!__pyx_v_sizes.memview)) __PYX_ERR(0, 376, __pyx_L3_error)
    if (values[2]) {
      __pyx_v_plot = __Pyx_PyObject_IsTrue(values[2]); if (unlikely((__pyx_v_plot == (int)-1) && PyErr_Occurred())) __PYX_ERR(0, 376, __pyx_L3_error)
    } else {
      __pyx_v_plot = ((int)0);
    }
  }
  goto __pyx_L4_argument_unpacking_done;
  __pyx_L5_argtuple_error:;
  __Pyx_RaiseArgtupleInvalid("exponent_relation", 0, 2, 3, PyTuple_GET_SIZE(__pyx_args)); __PYX_ERR(0, 376, __pyx_L3_error)
  __pyx_L3_error:;
  __Pyx_AddTraceback("avalanches.exponent_relation", __pyx_clineno, __pyx_lineno, __pyx_filename);
  __Pyx_RefNannyFinishContext();
  return NULL;
  __pyx_L4_argument_unpacking_done:;
  __pyx_r = __pyx_pf_10avalanches_12exponent_relation(__pyx_self, __pyx_v_lengths, __pyx_v_sizes, __pyx_v_plot);

  /* function exit code */
  __Pyx_RefNannyFinishContext();
  return __pyx_r;
}

static PyObject *__pyx_pf_10avalanches_12exponent_relation(CYTHON_UNUSED PyObject *__pyx_self, __Pyx_memviewslice __pyx_v_lengths, __Pyx_memviewslice __pyx_v_sizes, int __pyx_v_plot) {
  int __pyx_v_N_lens;
  PyObject *__pyx_v_length_dict = 0;
  int __pyx_v_num_lengths;
  int __pyx_v_i;
  __Pyx_memviewslice __pyx_v_avg_sizes = { 0, 0, { 0 }, { 0 }, { 0 } };
  __Pyx_memviewslice __pyx_v_log_avg_sizes = { 0, 0, { 0 }, { 0 }, { 0 } };
  __Pyx_memviewslice __pyx_v_given_lengths = { 0, 0, { 0 }, { 0 }, { 0 } };
  __Pyx_memviewslice __pyx_v_log_given_lengths = { 0, 0, { 0 }, { 0 }, { 0 } };
  int __pyx_v_counter;
  PyObject *__pyx_v_regression = NULL;
  double __pyx_v_slope;
  double __pyx_v_intercept;
  double __pyx_v_pval;
  __Pyx_memviewslice __pyx_v_x_axis = { 0, 0, { 0 }, { 0 }, { 0 } };
  int __pyx_v_N_x;
  __Pyx_memviewslice __pyx_v_y_axis = { 0, 0, { 0 }, { 0 }, { 0 } };
  PyObject *__pyx_r = NULL;
  __Pyx_RefNannyDeclarations
  __Pyx_RefNannySetupContext("exponent_relation", 0);
/* … */
  /* function exit code */
  __pyx_L1_error:;
  __Pyx_XDECREF(__pyx_t_1);
  __Pyx_XDECREF(__pyx_t_9);
  __Pyx_XDECREF(__pyx_t_15);
  __Pyx_XDECREF(__pyx_t_16);
  __Pyx_XDECREF(__pyx_t_17);
  __PYX_XDEC_MEMVIEW(&__pyx_t_18, 1);
  __Pyx_XDECREF(__pyx_t_26);
  __Pyx_XDECREF(__pyx_t_29);
  __Pyx_XDECREF(__pyx_t_30);
  __Pyx_AddTraceback("avalanches.exponent_relation", __pyx_clineno, __pyx_lineno, __pyx_filename);
  __pyx_r = NULL;
  __pyx_L0:;
  __Pyx_XDECREF(__pyx_v_length_dict);
  __PYX_XDEC_MEMVIEW(&__pyx_v_avg_sizes, 1);
  __PYX_XDEC_MEMVIEW(&__pyx_v_log_avg_sizes, 1);
  __PYX_XDEC_MEMVIEW(&__pyx_v_given_lengths, 1);
  __PYX_XDEC_MEMVIEW(&__pyx_v_log_given_lengths, 1);
  __Pyx_XDECREF(__pyx_v_regression);
  __PYX_XDEC_MEMVIEW(&__pyx_v_x_axis, 1);
  __PYX_XDEC_MEMVIEW(&__pyx_v_y_axis, 1);
  __PYX_XDEC_MEMVIEW(&__pyx_v_lengths, 1);
  __PYX_XDEC_MEMVIEW(&__pyx_v_sizes, 1);
  __Pyx_XGIVEREF(__pyx_r);
  __Pyx_RefNannyFinishContext();
  return __pyx_r;
}
/* … */
  __pyx_tuple__51 = PyTuple_Pack(19, __pyx_n_s_lengths, __pyx_n_s_sizes, __pyx_n_s_plot, __pyx_n_s_N_lens, __pyx_n_s_length_dict, __pyx_n_s_num_lengths, __pyx_n_s_i, __pyx_n_s_avg_sizes, __pyx_n_s_log_avg_sizes, __pyx_n_s_given_lengths, __pyx_n_s_log_given_lengths, __pyx_n_s_counter, __pyx_n_s_regression, __pyx_n_s_slope, __pyx_n_s_intercept, __pyx_n_s_pval, __pyx_n_s_x_axis, __pyx_n_s_N_x, __pyx_n_s_y_axis); if (unlikely(!__pyx_tuple__51)) __PYX_ERR(0, 376, __pyx_L1_error)
  __Pyx_GOTREF(__pyx_tuple__51);
  __Pyx_GIVEREF(__pyx_tuple__51);
/* … */
  __pyx_t_2 = PyCFunction_NewEx(&__pyx_mdef_10avalanches_13exponent_relation, NULL, __pyx_n_s_avalanches); if (unlikely(!__pyx_t_2)) __PYX_ERR(0, 376, __pyx_L1_error)
  __Pyx_GOTREF(__pyx_t_2);
  if (PyDict_SetItem(__pyx_d, __pyx_n_s_exponent_relation, __pyx_t_2) < 0) __PYX_ERR(0, 376, __pyx_L1_error)
  __Pyx_DECREF(__pyx_t_2); __pyx_t_2 = 0;
  __pyx_codeobj__52 = (PyObject*)__Pyx_PyCode_New(3, 0, 19, 0, CO_OPTIMIZED|CO_NEWLOCALS, __pyx_empty_bytes, __pyx_empty_tuple, __pyx_empty_tuple, __pyx_tuple__51, __pyx_empty_tuple, __pyx_empty_tuple, __pyx_kp_s_avalanches_pyx, __pyx_n_s_exponent_relation, 376, __pyx_empty_bytes); if (unlikely(!__pyx_codeobj__52)) __PYX_ERR(0, 376, __pyx_L1_error)
```

```
 377:     """
```

```
 378:     Returns the average size for a given length.
```

```
 379:     """
```

```
+380:     cdef int N_lens = lengths.shape[0]
```

```
  __pyx_v_N_lens = (__pyx_v_lengths.shape[0]);
```

```
+381:     cdef dict length_dict = {}
```

```
  __pyx_t_1 = __Pyx_PyDict_NewPresized(0); if (unlikely(!__pyx_t_1)) __PYX_ERR(0, 381, __pyx_L1_error)
  __Pyx_GOTREF(__pyx_t_1);
  __pyx_v_length_dict = ((PyObject*)__pyx_t_1);
  __pyx_t_1 = 0;
```

```
+382:     cdef int num_lengths = 0
```

```
  __pyx_v_num_lengths = 0;
```

```
 383:     cdef int i
```

```
 384:
```

```
+385:     for i in range(N_lens):
```

```
  __pyx_t_2 = __pyx_v_N_lens;
  __pyx_t_3 = __pyx_t_2;
  for (__pyx_t_4 = 0; __pyx_t_4 < __pyx_t_3; __pyx_t_4+=1) {
    __pyx_v_i = __pyx_t_4;
```

```
+386:         if lengths[i] not in length_dict:
```

```
    __pyx_t_5 = __pyx_v_i;
    __pyx_t_1 = __Pyx_PyInt_From_int((*((int *) ( /* dim=0 */ (__pyx_v_lengths.data + __pyx_t_5 * __pyx_v_lengths.strides[0]) )))); if (unlikely(!__pyx_t_1)) __PYX_ERR(0, 386, __pyx_L1_error)
    __Pyx_GOTREF(__pyx_t_1);
    __pyx_t_6 = (__Pyx_PyDict_ContainsTF(__pyx_t_1, __pyx_v_length_dict, Py_NE)); if (unlikely(__pyx_t_6 < 0)) __PYX_ERR(0, 386, __pyx_L1_error)
    __Pyx_DECREF(__pyx_t_1); __pyx_t_1 = 0;
    __pyx_t_7 = (__pyx_t_6 != 0);
    if (__pyx_t_7) {
/* … */
      goto __pyx_L5;
    }
```

```
+387:             length_dict[lengths[i]] = [sizes[i]]
```

```
      __pyx_t_8 = __pyx_v_i;
      __pyx_t_1 = __Pyx_PyInt_From_int((*((int *) ( /* dim=0 */ (__pyx_v_sizes.data + __pyx_t_8 * __pyx_v_sizes.strides[0]) )))); if (unlikely(!__pyx_t_1)) __PYX_ERR(0, 387, __pyx_L1_error)
      __Pyx_GOTREF(__pyx_t_1);
      __pyx_t_9 = PyList_New(1); if (unlikely(!__pyx_t_9)) __PYX_ERR(0, 387, __pyx_L1_error)
      __Pyx_GOTREF(__pyx_t_9);
      __Pyx_GIVEREF(__pyx_t_1);
      PyList_SET_ITEM(__pyx_t_9, 0, __pyx_t_1);
      __pyx_t_1 = 0;
      __pyx_t_10 = __pyx_v_i;
      __pyx_t_1 = __Pyx_PyInt_From_int((*((int *) ( /* dim=0 */ (__pyx_v_lengths.data + __pyx_t_10 * __pyx_v_lengths.strides[0]) )))); if (unlikely(!__pyx_t_1)) __PYX_ERR(0, 387, __pyx_L1_error)
      __Pyx_GOTREF(__pyx_t_1);
      if (unlikely(PyDict_SetItem(__pyx_v_length_dict, __pyx_t_1, __pyx_t_9) < 0)) __PYX_ERR(0, 387, __pyx_L1_error)
      __Pyx_DECREF(__pyx_t_1); __pyx_t_1 = 0;
      __Pyx_DECREF(__pyx_t_9); __pyx_t_9 = 0;
```

```
+388:             num_lengths += 1
```

```
      __pyx_v_num_lengths = (__pyx_v_num_lengths + 1);
```

```
+389:         elif lengths[i] in length_dict:
```

```
    __pyx_t_11 = __pyx_v_i;
    __pyx_t_9 = __Pyx_PyInt_From_int((*((int *) ( /* dim=0 */ (__pyx_v_lengths.data + __pyx_t_11 * __pyx_v_lengths.strides[0]) )))); if (unlikely(!__pyx_t_9)) __PYX_ERR(0, 389, __pyx_L1_error)
    __Pyx_GOTREF(__pyx_t_9);
    __pyx_t_7 = (__Pyx_PyDict_ContainsTF(__pyx_t_9, __pyx_v_length_dict, Py_EQ)); if (unlikely(__pyx_t_7 < 0)) __PYX_ERR(0, 389, __pyx_L1_error)
    __Pyx_DECREF(__pyx_t_9); __pyx_t_9 = 0;
    __pyx_t_6 = (__pyx_t_7 != 0);
    if (__pyx_t_6) {
/* … */
    }
    __pyx_L5:;
  }
```

```
+390:             length_dict[lengths[i]].append(sizes[i])
```

```
      __pyx_t_12 = __pyx_v_i;
      __pyx_t_9 = __Pyx_PyInt_From_int((*((int *) ( /* dim=0 */ (__pyx_v_lengths.data + __pyx_t_12 * __pyx_v_lengths.strides[0]) )))); if (unlikely(!__pyx_t_9)) __PYX_ERR(0, 390, __pyx_L1_error)
      __Pyx_GOTREF(__pyx_t_9);
      __pyx_t_1 = __Pyx_PyDict_GetItem(__pyx_v_length_dict, __pyx_t_9); if (unlikely(!__pyx_t_1)) __PYX_ERR(0, 390, __pyx_L1_error)
      __Pyx_GOTREF(__pyx_t_1);
      __Pyx_DECREF(__pyx_t_9); __pyx_t_9 = 0;
      __pyx_t_13 = __pyx_v_i;
      __pyx_t_9 = __Pyx_PyInt_From_int((*((int *) ( /* dim=0 */ (__pyx_v_sizes.data + __pyx_t_13 * __pyx_v_sizes.strides[0]) )))); if (unlikely(!__pyx_t_9)) __PYX_ERR(0, 390, __pyx_L1_error)
      __Pyx_GOTREF(__pyx_t_9);
      __pyx_t_14 = __Pyx_PyObject_Append(__pyx_t_1, __pyx_t_9); if (unlikely(__pyx_t_14 == ((int)-1))) __PYX_ERR(0, 390, __pyx_L1_error)
      __Pyx_DECREF(__pyx_t_1); __pyx_t_1 = 0;
      __Pyx_DECREF(__pyx_t_9); __pyx_t_9 = 0;
```

```
 391:
```

```
+392:     cdef double[:] avg_sizes = np.zeros(num_lengths)
```

```
  __pyx_t_1 = __Pyx_GetModuleGlobalName(__pyx_n_s_np); if (unlikely(!__pyx_t_1)) __PYX_ERR(0, 392, __pyx_L1_error)
  __Pyx_GOTREF(__pyx_t_1);
  __pyx_t_15 = __Pyx_PyObject_GetAttrStr(__pyx_t_1, __pyx_n_s_zeros); if (unlikely(!__pyx_t_15)) __PYX_ERR(0, 392, __pyx_L1_error)
  __Pyx_GOTREF(__pyx_t_15);
  __Pyx_DECREF(__pyx_t_1); __pyx_t_1 = 0;
  __pyx_t_1 = __Pyx_PyInt_From_int(__pyx_v_num_lengths); if (unlikely(!__pyx_t_1)) __PYX_ERR(0, 392, __pyx_L1_error)
  __Pyx_GOTREF(__pyx_t_1);
  __pyx_t_16 = NULL;
  if (CYTHON_UNPACK_METHODS && unlikely(PyMethod_Check(__pyx_t_15))) {
    __pyx_t_16 = PyMethod_GET_SELF(__pyx_t_15);
    if (likely(__pyx_t_16)) {
      PyObject* function = PyMethod_GET_FUNCTION(__pyx_t_15);
      __Pyx_INCREF(__pyx_t_16);
      __Pyx_INCREF(function);
      __Pyx_DECREF_SET(__pyx_t_15, function);
    }
  }
  if (!__pyx_t_16) {
    __pyx_t_9 = __Pyx_PyObject_CallOneArg(__pyx_t_15, __pyx_t_1); if (unlikely(!__pyx_t_9)) __PYX_ERR(0, 392, __pyx_L1_error)
    __Pyx_DECREF(__pyx_t_1); __pyx_t_1 = 0;
    __Pyx_GOTREF(__pyx_t_9);
  } else {
    #if CYTHON_FAST_PYCALL
    if (PyFunction_Check(__pyx_t_15)) {
      PyObject *__pyx_temp[2] = {__pyx_t_16, __pyx_t_1};
      __pyx_t_9 = __Pyx_PyFunction_FastCall(__pyx_t_15, __pyx_temp+1-1, 1+1); if (unlikely(!__pyx_t_9)) __PYX_ERR(0, 392, __pyx_L1_error)
      __Pyx_XDECREF(__pyx_t_16); __pyx_t_16 = 0;
      __Pyx_GOTREF(__pyx_t_9);
      __Pyx_DECREF(__pyx_t_1); __pyx_t_1 = 0;
    } else
    #endif
    #if CYTHON_FAST_PYCCALL
    if (__Pyx_PyFastCFunction_Check(__pyx_t_15)) {
      PyObject *__pyx_temp[2] = {__pyx_t_16, __pyx_t_1};
      __pyx_t_9 = __Pyx_PyCFunction_FastCall(__pyx_t_15, __pyx_temp+1-1, 1+1); if (unlikely(!__pyx_t_9)) __PYX_ERR(0, 392, __pyx_L1_error)
      __Pyx_XDECREF(__pyx_t_16); __pyx_t_16 = 0;
      __Pyx_GOTREF(__pyx_t_9);
      __Pyx_DECREF(__pyx_t_1); __pyx_t_1 = 0;
    } else
    #endif
    {
      __pyx_t_17 = PyTuple_New(1+1); if (unlikely(!__pyx_t_17)) __PYX_ERR(0, 392, __pyx_L1_error)
      __Pyx_GOTREF(__pyx_t_17);
      __Pyx_GIVEREF(__pyx_t_16); PyTuple_SET_ITEM(__pyx_t_17, 0, __pyx_t_16); __pyx_t_16 = NULL;
      __Pyx_GIVEREF(__pyx_t_1);
      PyTuple_SET_ITEM(__pyx_t_17, 0+1, __pyx_t_1);
      __pyx_t_1 = 0;
      __pyx_t_9 = __Pyx_PyObject_Call(__pyx_t_15, __pyx_t_17, NULL); if (unlikely(!__pyx_t_9)) __PYX_ERR(0, 392, __pyx_L1_error)
      __Pyx_GOTREF(__pyx_t_9);
      __Pyx_DECREF(__pyx_t_17); __pyx_t_17 = 0;
    }
  }
  __Pyx_DECREF(__pyx_t_15); __pyx_t_15 = 0;
  __pyx_t_18 = __Pyx_PyObject_to_MemoryviewSlice_ds_double(__pyx_t_9, PyBUF_WRITABLE); if (unlikely(!__pyx_t_18.memview)) __PYX_ERR(0, 392, __pyx_L1_error)
  __Pyx_DECREF(__pyx_t_9); __pyx_t_9 = 0;
  __pyx_v_avg_sizes = __pyx_t_18;
  __pyx_t_18.memview = NULL;
  __pyx_t_18.data = NULL;
```

```
+393:     cdef double[:] log_avg_sizes = np.zeros(num_lengths)
```

```
  __pyx_t_15 = __Pyx_GetModuleGlobalName(__pyx_n_s_np); if (unlikely(!__pyx_t_15)) __PYX_ERR(0, 393, __pyx_L1_error)
  __Pyx_GOTREF(__pyx_t_15);
  __pyx_t_17 = __Pyx_PyObject_GetAttrStr(__pyx_t_15, __pyx_n_s_zeros); if (unlikely(!__pyx_t_17)) __PYX_ERR(0, 393, __pyx_L1_error)
  __Pyx_GOTREF(__pyx_t_17);
  __Pyx_DECREF(__pyx_t_15); __pyx_t_15 = 0;
  __pyx_t_15 = __Pyx_PyInt_From_int(__pyx_v_num_lengths); if (unlikely(!__pyx_t_15)) __PYX_ERR(0, 393, __pyx_L1_error)
  __Pyx_GOTREF(__pyx_t_15);
  __pyx_t_1 = NULL;
  if (CYTHON_UNPACK_METHODS && unlikely(PyMethod_Check(__pyx_t_17))) {
    __pyx_t_1 = PyMethod_GET_SELF(__pyx_t_17);
    if (likely(__pyx_t_1)) {
      PyObject* function = PyMethod_GET_FUNCTION(__pyx_t_17);
      __Pyx_INCREF(__pyx_t_1);
      __Pyx_INCREF(function);
      __Pyx_DECREF_SET(__pyx_t_17, function);
    }
  }
  if (!__pyx_t_1) {
    __pyx_t_9 = __Pyx_PyObject_CallOneArg(__pyx_t_17, __pyx_t_15); if (unlikely(!__pyx_t_9)) __PYX_ERR(0, 393, __pyx_L1_error)
    __Pyx_DECREF(__pyx_t_15); __pyx_t_15 = 0;
    __Pyx_GOTREF(__pyx_t_9);
  } else {
    #if CYTHON_FAST_PYCALL
    if (PyFunction_Check(__pyx_t_17)) {
      PyObject *__pyx_temp[2] = {__pyx_t_1, __pyx_t_15};
      __pyx_t_9 = __Pyx_PyFunction_FastCall(__pyx_t_17, __pyx_temp+1-1, 1+1); if (unlikely(!__pyx_t_9)) __PYX_ERR(0, 393, __pyx_L1_error)
      __Pyx_XDECREF(__pyx_t_1); __pyx_t_1 = 0;
      __Pyx_GOTREF(__pyx_t_9);
      __Pyx_DECREF(__pyx_t_15); __pyx_t_15 = 0;
    } else
    #endif
    #if CYTHON_FAST_PYCCALL
    if (__Pyx_PyFastCFunction_Check(__pyx_t_17)) {
      PyObject *__pyx_temp[2] = {__pyx_t_1, __pyx_t_15};
      __pyx_t_9 = __Pyx_PyCFunction_FastCall(__pyx_t_17, __pyx_temp+1-1, 1+1); if (unlikely(!__pyx_t_9)) __PYX_ERR(0, 393, __pyx_L1_error)
      __Pyx_XDECREF(__pyx_t_1); __pyx_t_1 = 0;
      __Pyx_GOTREF(__pyx_t_9);
      __Pyx_DECREF(__pyx_t_15); __pyx_t_15 = 0;
    } else
    #endif
    {
      __pyx_t_16 = PyTuple_New(1+1); if (unlikely(!__pyx_t_16)) __PYX_ERR(0, 393, __pyx_L1_error)
      __Pyx_GOTREF(__pyx_t_16);
      __Pyx_GIVEREF(__pyx_t_1); PyTuple_SET_ITEM(__pyx_t_16, 0, __pyx_t_1); __pyx_t_1 = NULL;
      __Pyx_GIVEREF(__pyx_t_15);
      PyTuple_SET_ITEM(__pyx_t_16, 0+1, __pyx_t_15);
      __pyx_t_15 = 0;
      __pyx_t_9 = __Pyx_PyObject_Call(__pyx_t_17, __pyx_t_16, NULL); if (unlikely(!__pyx_t_9)) __PYX_ERR(0, 393, __pyx_L1_error)
      __Pyx_GOTREF(__pyx_t_9);
      __Pyx_DECREF(__pyx_t_16); __pyx_t_16 = 0;
    }
  }
  __Pyx_DECREF(__pyx_t_17); __pyx_t_17 = 0;
  __pyx_t_18 = __Pyx_PyObject_to_MemoryviewSlice_ds_double(__pyx_t_9, PyBUF_WRITABLE); if (unlikely(!__pyx_t_18.memview)) __PYX_ERR(0, 393, __pyx_L1_error)
  __Pyx_DECREF(__pyx_t_9); __pyx_t_9 = 0;
  __pyx_v_log_avg_sizes = __pyx_t_18;
  __pyx_t_18.memview = NULL;
  __pyx_t_18.data = NULL;
```

```
+394:     cdef double[:] given_lengths = np.zeros(num_lengths)
```

```
  __pyx_t_17 = __Pyx_GetModuleGlobalName(__pyx_n_s_np); if (unlikely(!__pyx_t_17)) __PYX_ERR(0, 394, __pyx_L1_error)
  __Pyx_GOTREF(__pyx_t_17);
  __pyx_t_16 = __Pyx_PyObject_GetAttrStr(__pyx_t_17, __pyx_n_s_zeros); if (unlikely(!__pyx_t_16)) __PYX_ERR(0, 394, __pyx_L1_error)
  __Pyx_GOTREF(__pyx_t_16);
  __Pyx_DECREF(__pyx_t_17); __pyx_t_17 = 0;
  __pyx_t_17 = __Pyx_PyInt_From_int(__pyx_v_num_lengths); if (unlikely(!__pyx_t_17)) __PYX_ERR(0, 394, __pyx_L1_error)
  __Pyx_GOTREF(__pyx_t_17);
  __pyx_t_15 = NULL;
  if (CYTHON_UNPACK_METHODS && unlikely(PyMethod_Check(__pyx_t_16))) {
    __pyx_t_15 = PyMethod_GET_SELF(__pyx_t_16);
    if (likely(__pyx_t_15)) {
      PyObject* function = PyMethod_GET_FUNCTION(__pyx_t_16);
      __Pyx_INCREF(__pyx_t_15);
      __Pyx_INCREF(function);
      __Pyx_DECREF_SET(__pyx_t_16, function);
    }
  }
  if (!__pyx_t_15) {
    __pyx_t_9 = __Pyx_PyObject_CallOneArg(__pyx_t_16, __pyx_t_17); if (unlikely(!__pyx_t_9)) __PYX_ERR(0, 394, __pyx_L1_error)
    __Pyx_DECREF(__pyx_t_17); __pyx_t_17 = 0;
    __Pyx_GOTREF(__pyx_t_9);
  } else {
    #if CYTHON_FAST_PYCALL
    if (PyFunction_Check(__pyx_t_16)) {
      PyObject *__pyx_temp[2] = {__pyx_t_15, __pyx_t_17};
      __pyx_t_9 = __Pyx_PyFunction_FastCall(__pyx_t_16, __pyx_temp+1-1, 1+1); if (unlikely(!__pyx_t_9)) __PYX_ERR(0, 394, __pyx_L1_error)
      __Pyx_XDECREF(__pyx_t_15); __pyx_t_15 = 0;
      __Pyx_GOTREF(__pyx_t_9);
      __Pyx_DECREF(__pyx_t_17); __pyx_t_17 = 0;
    } else
    #endif
    #if CYTHON_FAST_PYCCALL
    if (__Pyx_PyFastCFunction_Check(__pyx_t_16)) {
      PyObject *__pyx_temp[2] = {__pyx_t_15, __pyx_t_17};
      __pyx_t_9 = __Pyx_PyCFunction_FastCall(__pyx_t_16, __pyx_temp+1-1, 1+1); if (unlikely(!__pyx_t_9)) __PYX_ERR(0, 394, __pyx_L1_error)
      __Pyx_XDECREF(__pyx_t_15); __pyx_t_15 = 0;
      __Pyx_GOTREF(__pyx_t_9);
      __Pyx_DECREF(__pyx_t_17); __pyx_t_17 = 0;
    } else
    #endif
    {
      __pyx_t_1 = PyTuple_New(1+1); if (unlikely(!__pyx_t_1)) __PYX_ERR(0, 394, __pyx_L1_error)
      __Pyx_GOTREF(__pyx_t_1);
      __Pyx_GIVEREF(__pyx_t_15); PyTuple_SET_ITEM(__pyx_t_1, 0, __pyx_t_15); __pyx_t_15 = NULL;
      __Pyx_GIVEREF(__pyx_t_17);
      PyTuple_SET_ITEM(__pyx_t_1, 0+1, __pyx_t_17);
      __pyx_t_17 = 0;
      __pyx_t_9 = __Pyx_PyObject_Call(__pyx_t_16, __pyx_t_1, NULL); if (unlikely(!__pyx_t_9)) __PYX_ERR(0, 394, __pyx_L1_error)
      __Pyx_GOTREF(__pyx_t_9);
      __Pyx_DECREF(__pyx_t_1); __pyx_t_1 = 0;
    }
  }
  __Pyx_DECREF(__pyx_t_16); __pyx_t_16 = 0;
  __pyx_t_18 = __Pyx_PyObject_to_MemoryviewSlice_ds_double(__pyx_t_9, PyBUF_WRITABLE); if (unlikely(!__pyx_t_18.memview)) __PYX_ERR(0, 394, __pyx_L1_error)
  __Pyx_DECREF(__pyx_t_9); __pyx_t_9 = 0;
  __pyx_v_given_lengths = __pyx_t_18;
  __pyx_t_18.memview = NULL;
  __pyx_t_18.data = NULL;
```

```
+395:     cdef double[:]log_given_lengths = np.zeros(num_lengths)
```

```
  __pyx_t_16 = __Pyx_GetModuleGlobalName(__pyx_n_s_np); if (unlikely(!__pyx_t_16)) __PYX_ERR(0, 395, __pyx_L1_error)
  __Pyx_GOTREF(__pyx_t_16);
  __pyx_t_1 = __Pyx_PyObject_GetAttrStr(__pyx_t_16, __pyx_n_s_zeros); if (unlikely(!__pyx_t_1)) __PYX_ERR(0, 395, __pyx_L1_error)
  __Pyx_GOTREF(__pyx_t_1);
  __Pyx_DECREF(__pyx_t_16); __pyx_t_16 = 0;
  __pyx_t_16 = __Pyx_PyInt_From_int(__pyx_v_num_lengths); if (unlikely(!__pyx_t_16)) __PYX_ERR(0, 395, __pyx_L1_error)
  __Pyx_GOTREF(__pyx_t_16);
  __pyx_t_17 = NULL;
  if (CYTHON_UNPACK_METHODS && unlikely(PyMethod_Check(__pyx_t_1))) {
    __pyx_t_17 = PyMethod_GET_SELF(__pyx_t_1);
    if (likely(__pyx_t_17)) {
      PyObject* function = PyMethod_GET_FUNCTION(__pyx_t_1);
      __Pyx_INCREF(__pyx_t_17);
      __Pyx_INCREF(function);
      __Pyx_DECREF_SET(__pyx_t_1, function);
    }
  }
  if (!__pyx_t_17) {
    __pyx_t_9 = __Pyx_PyObject_CallOneArg(__pyx_t_1, __pyx_t_16); if (unlikely(!__pyx_t_9)) __PYX_ERR(0, 395, __pyx_L1_error)
    __Pyx_DECREF(__pyx_t_16); __pyx_t_16 = 0;
    __Pyx_GOTREF(__pyx_t_9);
  } else {
    #if CYTHON_FAST_PYCALL
    if (PyFunction_Check(__pyx_t_1)) {
      PyObject *__pyx_temp[2] = {__pyx_t_17, __pyx_t_16};
      __pyx_t_9 = __Pyx_PyFunction_FastCall(__pyx_t_1, __pyx_temp+1-1, 1+1); if (unlikely(!__pyx_t_9)) __PYX_ERR(0, 395, __pyx_L1_error)
      __Pyx_XDECREF(__pyx_t_17); __pyx_t_17 = 0;
      __Pyx_GOTREF(__pyx_t_9);
      __Pyx_DECREF(__pyx_t_16); __pyx_t_16 = 0;
    } else
    #endif
    #if CYTHON_FAST_PYCCALL
    if (__Pyx_PyFastCFunction_Check(__pyx_t_1)) {
      PyObject *__pyx_temp[2] = {__pyx_t_17, __pyx_t_16};
      __pyx_t_9 = __Pyx_PyCFunction_FastCall(__pyx_t_1, __pyx_temp+1-1, 1+1); if (unlikely(!__pyx_t_9)) __PYX_ERR(0, 395, __pyx_L1_error)
      __Pyx_XDECREF(__pyx_t_17); __pyx_t_17 = 0;
      __Pyx_GOTREF(__pyx_t_9);
      __Pyx_DECREF(__pyx_t_16); __pyx_t_16 = 0;
    } else
    #endif
    {
      __pyx_t_15 = PyTuple_New(1+1); if (unlikely(!__pyx_t_15)) __PYX_ERR(0, 395, __pyx_L1_error)
      __Pyx_GOTREF(__pyx_t_15);
      __Pyx_GIVEREF(__pyx_t_17); PyTuple_SET_ITEM(__pyx_t_15, 0, __pyx_t_17); __pyx_t_17 = NULL;
      __Pyx_GIVEREF(__pyx_t_16);
      PyTuple_SET_ITEM(__pyx_t_15, 0+1, __pyx_t_16);
      __pyx_t_16 = 0;
      __pyx_t_9 = __Pyx_PyObject_Call(__pyx_t_1, __pyx_t_15, NULL); if (unlikely(!__pyx_t_9)) __PYX_ERR(0, 395, __pyx_L1_error)
      __Pyx_GOTREF(__pyx_t_9);
      __Pyx_DECREF(__pyx_t_15); __pyx_t_15 = 0;
    }
  }
  __Pyx_DECREF(__pyx_t_1); __pyx_t_1 = 0;
  __pyx_t_18 = __Pyx_PyObject_to_MemoryviewSlice_ds_double(__pyx_t_9, PyBUF_WRITABLE); if (unlikely(!__pyx_t_18.memview)) __PYX_ERR(0, 395, __pyx_L1_error)
  __Pyx_DECREF(__pyx_t_9); __pyx_t_9 = 0;
  __pyx_v_log_given_lengths = __pyx_t_18;
  __pyx_t_18.memview = NULL;
  __pyx_t_18.data = NULL;
```

```
 396:
```

```
+397:     cdef int counter = 0
```

```
  __pyx_v_counter = 0;
```

```
+398:     for i in list(length_dict.keys()):
```

```
  __pyx_t_9 = __Pyx_PyDict_Keys(__pyx_v_length_dict); if (unlikely(!__pyx_t_9)) __PYX_ERR(0, 398, __pyx_L1_error)
  __Pyx_GOTREF(__pyx_t_9);
  __pyx_t_1 = PySequence_List(__pyx_t_9); if (unlikely(!__pyx_t_1)) __PYX_ERR(0, 398, __pyx_L1_error)
  __Pyx_GOTREF(__pyx_t_1);
  __Pyx_DECREF(__pyx_t_9); __pyx_t_9 = 0;
  __pyx_t_9 = __pyx_t_1; __Pyx_INCREF(__pyx_t_9); __pyx_t_19 = 0;
  __Pyx_DECREF(__pyx_t_1); __pyx_t_1 = 0;
  for (;;) {
    if (__pyx_t_19 >= PyList_GET_SIZE(__pyx_t_9)) break;
    #if CYTHON_ASSUME_SAFE_MACROS && !CYTHON_AVOID_BORROWED_REFS
    __pyx_t_1 = PyList_GET_ITEM(__pyx_t_9, __pyx_t_19); __Pyx_INCREF(__pyx_t_1); __pyx_t_19++; if (unlikely(0 < 0)) __PYX_ERR(0, 398, __pyx_L1_error)
    #else
    __pyx_t_1 = PySequence_ITEM(__pyx_t_9, __pyx_t_19); __pyx_t_19++; if (unlikely(!__pyx_t_1)) __PYX_ERR(0, 398, __pyx_L1_error)
    __Pyx_GOTREF(__pyx_t_1);
    #endif
    __pyx_t_2 = __Pyx_PyInt_As_int(__pyx_t_1); if (unlikely((__pyx_t_2 == (int)-1) && PyErr_Occurred())) __PYX_ERR(0, 398, __pyx_L1_error)
    __Pyx_DECREF(__pyx_t_1); __pyx_t_1 = 0;
    __pyx_v_i = __pyx_t_2;
/* … */
  }
  __Pyx_DECREF(__pyx_t_9); __pyx_t_9 = 0;
```

```
+399:         given_lengths[counter] = i
```

```
    __pyx_t_20 = __pyx_v_counter;
    *((double *) ( /* dim=0 */ (__pyx_v_given_lengths.data + __pyx_t_20 * __pyx_v_given_lengths.strides[0]) )) = __pyx_v_i;
```

```
+400:         log_given_lengths[counter] = log10(i)
```

```
    __pyx_t_21 = __pyx_v_counter;
    *((double *) ( /* dim=0 */ (__pyx_v_log_given_lengths.data + __pyx_t_21 * __pyx_v_log_given_lengths.strides[0]) )) = log10(__pyx_v_i);
```

```
 401:
```

```
+402:         avg_sizes[counter] = sum(length_dict[i])/len(length_dict[i])
```

```
    __pyx_t_1 = __Pyx_PyInt_From_int(__pyx_v_i); if (unlikely(!__pyx_t_1)) __PYX_ERR(0, 402, __pyx_L1_error)
    __Pyx_GOTREF(__pyx_t_1);
    __pyx_t_15 = __Pyx_PyDict_GetItem(__pyx_v_length_dict, __pyx_t_1); if (unlikely(!__pyx_t_15)) __PYX_ERR(0, 402, __pyx_L1_error)
    __Pyx_GOTREF(__pyx_t_15);
    __Pyx_DECREF(__pyx_t_1); __pyx_t_1 = 0;
    __pyx_t_1 = __Pyx_PyObject_CallOneArg(__pyx_builtin_sum, __pyx_t_15); if (unlikely(!__pyx_t_1)) __PYX_ERR(0, 402, __pyx_L1_error)
    __Pyx_GOTREF(__pyx_t_1);
    __Pyx_DECREF(__pyx_t_15); __pyx_t_15 = 0;
    __pyx_t_15 = __Pyx_PyInt_From_int(__pyx_v_i); if (unlikely(!__pyx_t_15)) __PYX_ERR(0, 402, __pyx_L1_error)
    __Pyx_GOTREF(__pyx_t_15);
    __pyx_t_16 = __Pyx_PyDict_GetItem(__pyx_v_length_dict, __pyx_t_15); if (unlikely(!__pyx_t_16)) __PYX_ERR(0, 402, __pyx_L1_error)
    __Pyx_GOTREF(__pyx_t_16);
    __Pyx_DECREF(__pyx_t_15); __pyx_t_15 = 0;
    __pyx_t_22 = PyObject_Length(__pyx_t_16); if (unlikely(__pyx_t_22 == ((Py_ssize_t)-1))) __PYX_ERR(0, 402, __pyx_L1_error)
    __Pyx_DECREF(__pyx_t_16); __pyx_t_16 = 0;
    __pyx_t_16 = PyInt_FromSsize_t(__pyx_t_22); if (unlikely(!__pyx_t_16)) __PYX_ERR(0, 402, __pyx_L1_error)
    __Pyx_GOTREF(__pyx_t_16);
    __pyx_t_15 = __Pyx_PyNumber_Divide(__pyx_t_1, __pyx_t_16); if (unlikely(!__pyx_t_15)) __PYX_ERR(0, 402, __pyx_L1_error)
    __Pyx_GOTREF(__pyx_t_15);
    __Pyx_DECREF(__pyx_t_1); __pyx_t_1 = 0;
    __Pyx_DECREF(__pyx_t_16); __pyx_t_16 = 0;
    __pyx_t_23 = __pyx_PyFloat_AsDouble(__pyx_t_15); if (unlikely((__pyx_t_23 == (double)-1) && PyErr_Occurred())) __PYX_ERR(0, 402, __pyx_L1_error)
    __Pyx_DECREF(__pyx_t_15); __pyx_t_15 = 0;
    __pyx_t_24 = __pyx_v_counter;
    *((double *) ( /* dim=0 */ (__pyx_v_avg_sizes.data + __pyx_t_24 * __pyx_v_avg_sizes.strides[0]) )) = __pyx_t_23;
```

```
+403:         log_avg_sizes[counter] = log10(sum(length_dict[i])/len(length_dict[i]))
```

```
    __pyx_t_15 = __Pyx_PyInt_From_int(__pyx_v_i); if (unlikely(!__pyx_t_15)) __PYX_ERR(0, 403, __pyx_L1_error)
    __Pyx_GOTREF(__pyx_t_15);
    __pyx_t_16 = __Pyx_PyDict_GetItem(__pyx_v_length_dict, __pyx_t_15); if (unlikely(!__pyx_t_16)) __PYX_ERR(0, 403, __pyx_L1_error)
    __Pyx_GOTREF(__pyx_t_16);
    __Pyx_DECREF(__pyx_t_15); __pyx_t_15 = 0;
    __pyx_t_15 = __Pyx_PyObject_CallOneArg(__pyx_builtin_sum, __pyx_t_16); if (unlikely(!__pyx_t_15)) __PYX_ERR(0, 403, __pyx_L1_error)
    __Pyx_GOTREF(__pyx_t_15);
    __Pyx_DECREF(__pyx_t_16); __pyx_t_16 = 0;
    __pyx_t_16 = __Pyx_PyInt_From_int(__pyx_v_i); if (unlikely(!__pyx_t_16)) __PYX_ERR(0, 403, __pyx_L1_error)
    __Pyx_GOTREF(__pyx_t_16);
    __pyx_t_1 = __Pyx_PyDict_GetItem(__pyx_v_length_dict, __pyx_t_16); if (unlikely(!__pyx_t_1)) __PYX_ERR(0, 403, __pyx_L1_error)
    __Pyx_GOTREF(__pyx_t_1);
    __Pyx_DECREF(__pyx_t_16); __pyx_t_16 = 0;
    __pyx_t_22 = PyObject_Length(__pyx_t_1); if (unlikely(__pyx_t_22 == ((Py_ssize_t)-1))) __PYX_ERR(0, 403, __pyx_L1_error)
    __Pyx_DECREF(__pyx_t_1); __pyx_t_1 = 0;
    __pyx_t_1 = PyInt_FromSsize_t(__pyx_t_22); if (unlikely(!__pyx_t_1)) __PYX_ERR(0, 403, __pyx_L1_error)
    __Pyx_GOTREF(__pyx_t_1);
    __pyx_t_16 = __Pyx_PyNumber_Divide(__pyx_t_15, __pyx_t_1); if (unlikely(!__pyx_t_16)) __PYX_ERR(0, 403, __pyx_L1_error)
    __Pyx_GOTREF(__pyx_t_16);
    __Pyx_DECREF(__pyx_t_15); __pyx_t_15 = 0;
    __Pyx_DECREF(__pyx_t_1); __pyx_t_1 = 0;
    __pyx_t_23 = __pyx_PyFloat_AsDouble(__pyx_t_16); if (unlikely((__pyx_t_23 == (double)-1) && PyErr_Occurred())) __PYX_ERR(0, 403, __pyx_L1_error)
    __Pyx_DECREF(__pyx_t_16); __pyx_t_16 = 0;
    __pyx_t_25 = __pyx_v_counter;
    *((double *) ( /* dim=0 */ (__pyx_v_log_avg_sizes.data + __pyx_t_25 * __pyx_v_log_avg_sizes.strides[0]) )) = log10(__pyx_t_23);
```

```
+404:         counter += 1
```

```
    __pyx_v_counter = (__pyx_v_counter + 1);
```

```
 405:
```

```
+406:     regression = linregress(log_given_lengths, log_avg_sizes)
```

```
  __pyx_t_16 = __Pyx_GetModuleGlobalName(__pyx_n_s_linregress); if (unlikely(!__pyx_t_16)) __PYX_ERR(0, 406, __pyx_L1_error)
  __Pyx_GOTREF(__pyx_t_16);
  __pyx_t_1 = __pyx_memoryview_fromslice(__pyx_v_log_given_lengths, 1, (PyObject *(*)(char *)) __pyx_memview_get_double, (int (*)(char *, PyObject *)) __pyx_memview_set_double, 0);; if (unlikely(!__pyx_t_1)) __PYX_ERR(0, 406, __pyx_L1_error)
  __Pyx_GOTREF(__pyx_t_1);
  __pyx_t_15 = __pyx_memoryview_fromslice(__pyx_v_log_avg_sizes, 1, (PyObject *(*)(char *)) __pyx_memview_get_double, (int (*)(char *, PyObject *)) __pyx_memview_set_double, 0);; if (unlikely(!__pyx_t_15)) __PYX_ERR(0, 406, __pyx_L1_error)
  __Pyx_GOTREF(__pyx_t_15);
  __pyx_t_17 = NULL;
  __pyx_t_2 = 0;
  if (CYTHON_UNPACK_METHODS && unlikely(PyMethod_Check(__pyx_t_16))) {
    __pyx_t_17 = PyMethod_GET_SELF(__pyx_t_16);
    if (likely(__pyx_t_17)) {
      PyObject* function = PyMethod_GET_FUNCTION(__pyx_t_16);
      __Pyx_INCREF(__pyx_t_17);
      __Pyx_INCREF(function);
      __Pyx_DECREF_SET(__pyx_t_16, function);
      __pyx_t_2 = 1;
    }
  }
  #if CYTHON_FAST_PYCALL
  if (PyFunction_Check(__pyx_t_16)) {
    PyObject *__pyx_temp[3] = {__pyx_t_17, __pyx_t_1, __pyx_t_15};
    __pyx_t_9 = __Pyx_PyFunction_FastCall(__pyx_t_16, __pyx_temp+1-__pyx_t_2, 2+__pyx_t_2); if (unlikely(!__pyx_t_9)) __PYX_ERR(0, 406, __pyx_L1_error)
    __Pyx_XDECREF(__pyx_t_17); __pyx_t_17 = 0;
    __Pyx_GOTREF(__pyx_t_9);
    __Pyx_DECREF(__pyx_t_1); __pyx_t_1 = 0;
    __Pyx_DECREF(__pyx_t_15); __pyx_t_15 = 0;
  } else
  #endif
  #if CYTHON_FAST_PYCCALL
  if (__Pyx_PyFastCFunction_Check(__pyx_t_16)) {
    PyObject *__pyx_temp[3] = {__pyx_t_17, __pyx_t_1, __pyx_t_15};
    __pyx_t_9 = __Pyx_PyCFunction_FastCall(__pyx_t_16, __pyx_temp+1-__pyx_t_2, 2+__pyx_t_2); if (unlikely(!__pyx_t_9)) __PYX_ERR(0, 406, __pyx_L1_error)
    __Pyx_XDECREF(__pyx_t_17); __pyx_t_17 = 0;
    __Pyx_GOTREF(__pyx_t_9);
    __Pyx_DECREF(__pyx_t_1); __pyx_t_1 = 0;
    __Pyx_DECREF(__pyx_t_15); __pyx_t_15 = 0;
  } else
  #endif
  {
    __pyx_t_26 = PyTuple_New(2+__pyx_t_2); if (unlikely(!__pyx_t_26)) __PYX_ERR(0, 406, __pyx_L1_error)
    __Pyx_GOTREF(__pyx_t_26);
    if (__pyx_t_17) {
      __Pyx_GIVEREF(__pyx_t_17); PyTuple_SET_ITEM(__pyx_t_26, 0, __pyx_t_17); __pyx_t_17 = NULL;
    }
    __Pyx_GIVEREF(__pyx_t_1);
    PyTuple_SET_ITEM(__pyx_t_26, 0+__pyx_t_2, __pyx_t_1);
    __Pyx_GIVEREF(__pyx_t_15);
    PyTuple_SET_ITEM(__pyx_t_26, 1+__pyx_t_2, __pyx_t_15);
    __pyx_t_1 = 0;
    __pyx_t_15 = 0;
    __pyx_t_9 = __Pyx_PyObject_Call(__pyx_t_16, __pyx_t_26, NULL); if (unlikely(!__pyx_t_9)) __PYX_ERR(0, 406, __pyx_L1_error)
    __Pyx_GOTREF(__pyx_t_9);
    __Pyx_DECREF(__pyx_t_26); __pyx_t_26 = 0;
  }
  __Pyx_DECREF(__pyx_t_16); __pyx_t_16 = 0;
  __pyx_v_regression = __pyx_t_9;
  __pyx_t_9 = 0;
```

```
 407:
```

```
+408:     cdef double slope = regression[0]
```

```
  __pyx_t_9 = __Pyx_GetItemInt(__pyx_v_regression, 0, long, 1, __Pyx_PyInt_From_long, 0, 0, 0); if (unlikely(!__pyx_t_9)) __PYX_ERR(0, 408, __pyx_L1_error)
  __Pyx_GOTREF(__pyx_t_9);
  __pyx_t_23 = __pyx_PyFloat_AsDouble(__pyx_t_9); if (unlikely((__pyx_t_23 == (double)-1) && PyErr_Occurred())) __PYX_ERR(0, 408, __pyx_L1_error)
  __Pyx_DECREF(__pyx_t_9); __pyx_t_9 = 0;
  __pyx_v_slope = __pyx_t_23;
```

```
+409:     cdef double intercept = regression[1]
```

```
  __pyx_t_9 = __Pyx_GetItemInt(__pyx_v_regression, 1, long, 1, __Pyx_PyInt_From_long, 0, 0, 0); if (unlikely(!__pyx_t_9)) __PYX_ERR(0, 409, __pyx_L1_error)
  __Pyx_GOTREF(__pyx_t_9);
  __pyx_t_23 = __pyx_PyFloat_AsDouble(__pyx_t_9); if (unlikely((__pyx_t_23 == (double)-1) && PyErr_Occurred())) __PYX_ERR(0, 409, __pyx_L1_error)
  __Pyx_DECREF(__pyx_t_9); __pyx_t_9 = 0;
  __pyx_v_intercept = __pyx_t_23;
```

```
+410:     cdef double pval = regression[3]
```

```
  __pyx_t_9 = __Pyx_GetItemInt(__pyx_v_regression, 3, long, 1, __Pyx_PyInt_From_long, 0, 0, 0); if (unlikely(!__pyx_t_9)) __PYX_ERR(0, 410, __pyx_L1_error)
  __Pyx_GOTREF(__pyx_t_9);
  __pyx_t_23 = __pyx_PyFloat_AsDouble(__pyx_t_9); if (unlikely((__pyx_t_23 == (double)-1) && PyErr_Occurred())) __PYX_ERR(0, 410, __pyx_L1_error)
  __Pyx_DECREF(__pyx_t_9); __pyx_t_9 = 0;
  __pyx_v_pval = __pyx_t_23;
```

```
 411:
```

```
 412:     cdef double[:] x_axis
```

```
 413:     cdef int N_x
```

```
 414:     cdef double[:] y_axis
```

```
 415:
```

```
 416:
```

```
+417:     x_axis = np.arange(min(given_lengths), max(given_lengths), 0.01)
```

```
  __pyx_t_16 = __Pyx_GetModuleGlobalName(__pyx_n_s_np); if (unlikely(!__pyx_t_16)) __PYX_ERR(0, 417, __pyx_L1_error)
  __Pyx_GOTREF(__pyx_t_16);
  __pyx_t_26 = __Pyx_PyObject_GetAttrStr(__pyx_t_16, __pyx_n_s_arange); if (unlikely(!__pyx_t_26)) __PYX_ERR(0, 417, __pyx_L1_error)
  __Pyx_GOTREF(__pyx_t_26);
  __Pyx_DECREF(__pyx_t_16); __pyx_t_16 = 0;
  __pyx_t_16 = __pyx_memoryview_fromslice(__pyx_v_given_lengths, 1, (PyObject *(*)(char *)) __pyx_memview_get_double, (int (*)(char *, PyObject *)) __pyx_memview_set_double, 0);; if (unlikely(!__pyx_t_16)) __PYX_ERR(0, 417, __pyx_L1_error)
  __Pyx_GOTREF(__pyx_t_16);
  __pyx_t_15 = __Pyx_PyObject_CallOneArg(__pyx_builtin_min, __pyx_t_16); if (unlikely(!__pyx_t_15)) __PYX_ERR(0, 417, __pyx_L1_error)
  __Pyx_GOTREF(__pyx_t_15);
  __Pyx_DECREF(__pyx_t_16); __pyx_t_16 = 0;
  __pyx_t_16 = __pyx_memoryview_fromslice(__pyx_v_given_lengths, 1, (PyObject *(*)(char *)) __pyx_memview_get_double, (int (*)(char *, PyObject *)) __pyx_memview_set_double, 0);; if (unlikely(!__pyx_t_16)) __PYX_ERR(0, 417, __pyx_L1_error)
  __Pyx_GOTREF(__pyx_t_16);
  __pyx_t_1 = __Pyx_PyObject_CallOneArg(__pyx_builtin_max, __pyx_t_16); if (unlikely(!__pyx_t_1)) __PYX_ERR(0, 417, __pyx_L1_error)
  __Pyx_GOTREF(__pyx_t_1);
  __Pyx_DECREF(__pyx_t_16); __pyx_t_16 = 0;
  __pyx_t_16 = NULL;
  __pyx_t_2 = 0;
  if (CYTHON_UNPACK_METHODS && unlikely(PyMethod_Check(__pyx_t_26))) {
    __pyx_t_16 = PyMethod_GET_SELF(__pyx_t_26);
    if (likely(__pyx_t_16)) {
      PyObject* function = PyMethod_GET_FUNCTION(__pyx_t_26);
      __Pyx_INCREF(__pyx_t_16);
      __Pyx_INCREF(function);
      __Pyx_DECREF_SET(__pyx_t_26, function);
      __pyx_t_2 = 1;
    }
  }
  #if CYTHON_FAST_PYCALL
  if (PyFunction_Check(__pyx_t_26)) {
    PyObject *__pyx_temp[4] = {__pyx_t_16, __pyx_t_15, __pyx_t_1, __pyx_float_0_01};
    __pyx_t_9 = __Pyx_PyFunction_FastCall(__pyx_t_26, __pyx_temp+1-__pyx_t_2, 3+__pyx_t_2); if (unlikely(!__pyx_t_9)) __PYX_ERR(0, 417, __pyx_L1_error)
    __Pyx_XDECREF(__pyx_t_16); __pyx_t_16 = 0;
    __Pyx_GOTREF(__pyx_t_9);
    __Pyx_DECREF(__pyx_t_15); __pyx_t_15 = 0;
    __Pyx_DECREF(__pyx_t_1); __pyx_t_1 = 0;
  } else
  #endif
  #if CYTHON_FAST_PYCCALL
  if (__Pyx_PyFastCFunction_Check(__pyx_t_26)) {
    PyObject *__pyx_temp[4] = {__pyx_t_16, __pyx_t_15, __pyx_t_1, __pyx_float_0_01};
    __pyx_t_9 = __Pyx_PyCFunction_FastCall(__pyx_t_26, __pyx_temp+1-__pyx_t_2, 3+__pyx_t_2); if (unlikely(!__pyx_t_9)) __PYX_ERR(0, 417, __pyx_L1_error)
    __Pyx_XDECREF(__pyx_t_16); __pyx_t_16 = 0;
    __Pyx_GOTREF(__pyx_t_9);
    __Pyx_DECREF(__pyx_t_15); __pyx_t_15 = 0;
    __Pyx_DECREF(__pyx_t_1); __pyx_t_1 = 0;
  } else
  #endif
  {
    __pyx_t_17 = PyTuple_New(3+__pyx_t_2); if (unlikely(!__pyx_t_17)) __PYX_ERR(0, 417, __pyx_L1_error)
    __Pyx_GOTREF(__pyx_t_17);
    if (__pyx_t_16) {
      __Pyx_GIVEREF(__pyx_t_16); PyTuple_SET_ITEM(__pyx_t_17, 0, __pyx_t_16); __pyx_t_16 = NULL;
    }
    __Pyx_GIVEREF(__pyx_t_15);
    PyTuple_SET_ITEM(__pyx_t_17, 0+__pyx_t_2, __pyx_t_15);
    __Pyx_GIVEREF(__pyx_t_1);
    PyTuple_SET_ITEM(__pyx_t_17, 1+__pyx_t_2, __pyx_t_1);
    __Pyx_INCREF(__pyx_float_0_01);
    __Pyx_GIVEREF(__pyx_float_0_01);
    PyTuple_SET_ITEM(__pyx_t_17, 2+__pyx_t_2, __pyx_float_0_01);
    __pyx_t_15 = 0;
    __pyx_t_1 = 0;
    __pyx_t_9 = __Pyx_PyObject_Call(__pyx_t_26, __pyx_t_17, NULL); if (unlikely(!__pyx_t_9)) __PYX_ERR(0, 417, __pyx_L1_error)
    __Pyx_GOTREF(__pyx_t_9);
    __Pyx_DECREF(__pyx_t_17); __pyx_t_17 = 0;
  }
  __Pyx_DECREF(__pyx_t_26); __pyx_t_26 = 0;
  __pyx_t_18 = __Pyx_PyObject_to_MemoryviewSlice_ds_double(__pyx_t_9, PyBUF_WRITABLE); if (unlikely(!__pyx_t_18.memview)) __PYX_ERR(0, 417, __pyx_L1_error)
  __Pyx_DECREF(__pyx_t_9); __pyx_t_9 = 0;
  __pyx_v_x_axis = __pyx_t_18;
  __pyx_t_18.memview = NULL;
  __pyx_t_18.data = NULL;
```

```
+418:     N_x = x_axis.shape[0]
```

```
  __pyx_v_N_x = (__pyx_v_x_axis.shape[0]);
```

```
+419:     y_axis = np.zeros(N_x)
```

```
  __pyx_t_26 = __Pyx_GetModuleGlobalName(__pyx_n_s_np); if (unlikely(!__pyx_t_26)) __PYX_ERR(0, 419, __pyx_L1_error)
  __Pyx_GOTREF(__pyx_t_26);
  __pyx_t_17 = __Pyx_PyObject_GetAttrStr(__pyx_t_26, __pyx_n_s_zeros); if (unlikely(!__pyx_t_17)) __PYX_ERR(0, 419, __pyx_L1_error)
  __Pyx_GOTREF(__pyx_t_17);
  __Pyx_DECREF(__pyx_t_26); __pyx_t_26 = 0;
  __pyx_t_26 = __Pyx_PyInt_From_int(__pyx_v_N_x); if (unlikely(!__pyx_t_26)) __PYX_ERR(0, 419, __pyx_L1_error)
  __Pyx_GOTREF(__pyx_t_26);
  __pyx_t_1 = NULL;
  if (CYTHON_UNPACK_METHODS && unlikely(PyMethod_Check(__pyx_t_17))) {
    __pyx_t_1 = PyMethod_GET_SELF(__pyx_t_17);
    if (likely(__pyx_t_1)) {
      PyObject* function = PyMethod_GET_FUNCTION(__pyx_t_17);
      __Pyx_INCREF(__pyx_t_1);
      __Pyx_INCREF(function);
      __Pyx_DECREF_SET(__pyx_t_17, function);
    }
  }
  if (!__pyx_t_1) {
    __pyx_t_9 = __Pyx_PyObject_CallOneArg(__pyx_t_17, __pyx_t_26); if (unlikely(!__pyx_t_9)) __PYX_ERR(0, 419, __pyx_L1_error)
    __Pyx_DECREF(__pyx_t_26); __pyx_t_26 = 0;
    __Pyx_GOTREF(__pyx_t_9);
  } else {
    #if CYTHON_FAST_PYCALL
    if (PyFunction_Check(__pyx_t_17)) {
      PyObject *__pyx_temp[2] = {__pyx_t_1, __pyx_t_26};
      __pyx_t_9 = __Pyx_PyFunction_FastCall(__pyx_t_17, __pyx_temp+1-1, 1+1); if (unlikely(!__pyx_t_9)) __PYX_ERR(0, 419, __pyx_L1_error)
      __Pyx_XDECREF(__pyx_t_1); __pyx_t_1 = 0;
      __Pyx_GOTREF(__pyx_t_9);
      __Pyx_DECREF(__pyx_t_26); __pyx_t_26 = 0;
    } else
    #endif
    #if CYTHON_FAST_PYCCALL
    if (__Pyx_PyFastCFunction_Check(__pyx_t_17)) {
      PyObject *__pyx_temp[2] = {__pyx_t_1, __pyx_t_26};
      __pyx_t_9 = __Pyx_PyCFunction_FastCall(__pyx_t_17, __pyx_temp+1-1, 1+1); if (unlikely(!__pyx_t_9)) __PYX_ERR(0, 419, __pyx_L1_error)
      __Pyx_XDECREF(__pyx_t_1); __pyx_t_1 = 0;
      __Pyx_GOTREF(__pyx_t_9);
      __Pyx_DECREF(__pyx_t_26); __pyx_t_26 = 0;
    } else
    #endif
    {
      __pyx_t_15 = PyTuple_New(1+1); if (unlikely(!__pyx_t_15)) __PYX_ERR(0, 419, __pyx_L1_error)
      __Pyx_GOTREF(__pyx_t_15);
      __Pyx_GIVEREF(__pyx_t_1); PyTuple_SET_ITEM(__pyx_t_15, 0, __pyx_t_1); __pyx_t_1 = NULL;
      __Pyx_GIVEREF(__pyx_t_26);
      PyTuple_SET_ITEM(__pyx_t_15, 0+1, __pyx_t_26);
      __pyx_t_26 = 0;
      __pyx_t_9 = __Pyx_PyObject_Call(__pyx_t_17, __pyx_t_15, NULL); if (unlikely(!__pyx_t_9)) __PYX_ERR(0, 419, __pyx_L1_error)
      __Pyx_GOTREF(__pyx_t_9);
      __Pyx_DECREF(__pyx_t_15); __pyx_t_15 = 0;
    }
  }
  __Pyx_DECREF(__pyx_t_17); __pyx_t_17 = 0;
  __pyx_t_18 = __Pyx_PyObject_to_MemoryviewSlice_ds_double(__pyx_t_9, PyBUF_WRITABLE); if (unlikely(!__pyx_t_18.memview)) __PYX_ERR(0, 419, __pyx_L1_error)
  __Pyx_DECREF(__pyx_t_9); __pyx_t_9 = 0;
  __pyx_v_y_axis = __pyx_t_18;
  __pyx_t_18.memview = NULL;
  __pyx_t_18.data = NULL;
```

```
 420:
```

```
+421:     for i in range(N_x):
```

```
  __pyx_t_2 = __pyx_v_N_x;
  __pyx_t_3 = __pyx_t_2;
  for (__pyx_t_4 = 0; __pyx_t_4 < __pyx_t_3; __pyx_t_4+=1) {
    __pyx_v_i = __pyx_t_4;
```

```
+422:         y_axis[i] = 10**intercept * (x_axis[i]**slope)
```

```
    __pyx_t_27 = __pyx_v_i;
    __pyx_t_28 = __pyx_v_i;
    *((double *) ( /* dim=0 */ (__pyx_v_y_axis.data + __pyx_t_28 * __pyx_v_y_axis.strides[0]) )) = (pow(10.0, __pyx_v_intercept) * pow((*((double *) ( /* dim=0 */ (__pyx_v_x_axis.data + __pyx_t_27 * __pyx_v_x_axis.strides[0]) ))), __pyx_v_slope));
  }
```

```
 423:
```

```
+424:     if plot == True:
```

```
  __pyx_t_6 = ((__pyx_v_plot == 1) != 0);
  if (__pyx_t_6) {
/* … */
  }
```

```
+425:         plt.subplots()
```

```
    __pyx_t_17 = __Pyx_GetModuleGlobalName(__pyx_n_s_plt); if (unlikely(!__pyx_t_17)) __PYX_ERR(0, 425, __pyx_L1_error)
    __Pyx_GOTREF(__pyx_t_17);
    __pyx_t_15 = __Pyx_PyObject_GetAttrStr(__pyx_t_17, __pyx_n_s_subplots); if (unlikely(!__pyx_t_15)) __PYX_ERR(0, 425, __pyx_L1_error)
    __Pyx_GOTREF(__pyx_t_15);
    __Pyx_DECREF(__pyx_t_17); __pyx_t_17 = 0;
    __pyx_t_17 = NULL;
    if (CYTHON_UNPACK_METHODS && unlikely(PyMethod_Check(__pyx_t_15))) {
      __pyx_t_17 = PyMethod_GET_SELF(__pyx_t_15);
      if (likely(__pyx_t_17)) {
        PyObject* function = PyMethod_GET_FUNCTION(__pyx_t_15);
        __Pyx_INCREF(__pyx_t_17);
        __Pyx_INCREF(function);
        __Pyx_DECREF_SET(__pyx_t_15, function);
      }
    }
    if (__pyx_t_17) {
      __pyx_t_9 = __Pyx_PyObject_CallOneArg(__pyx_t_15, __pyx_t_17); if (unlikely(!__pyx_t_9)) __PYX_ERR(0, 425, __pyx_L1_error)
      __Pyx_DECREF(__pyx_t_17); __pyx_t_17 = 0;
    } else {
      __pyx_t_9 = __Pyx_PyObject_CallNoArg(__pyx_t_15); if (unlikely(!__pyx_t_9)) __PYX_ERR(0, 425, __pyx_L1_error)
    }
    __Pyx_GOTREF(__pyx_t_9);
    __Pyx_DECREF(__pyx_t_15); __pyx_t_15 = 0;
    __Pyx_DECREF(__pyx_t_9); __pyx_t_9 = 0;
```

```
+426:         plt.loglog(given_lengths, avg_sizes, ".")
```

```
    __pyx_t_15 = __Pyx_GetModuleGlobalName(__pyx_n_s_plt); if (unlikely(!__pyx_t_15)) __PYX_ERR(0, 426, __pyx_L1_error)
    __Pyx_GOTREF(__pyx_t_15);
    __pyx_t_17 = __Pyx_PyObject_GetAttrStr(__pyx_t_15, __pyx_n_s_loglog); if (unlikely(!__pyx_t_17)) __PYX_ERR(0, 426, __pyx_L1_error)
    __Pyx_GOTREF(__pyx_t_17);
    __Pyx_DECREF(__pyx_t_15); __pyx_t_15 = 0;
    __pyx_t_15 = __pyx_memoryview_fromslice(__pyx_v_given_lengths, 1, (PyObject *(*)(char *)) __pyx_memview_get_double, (int (*)(char *, PyObject *)) __pyx_memview_set_double, 0);; if (unlikely(!__pyx_t_15)) __PYX_ERR(0, 426, __pyx_L1_error)
    __Pyx_GOTREF(__pyx_t_15);
    __pyx_t_26 = __pyx_memoryview_fromslice(__pyx_v_avg_sizes, 1, (PyObject *(*)(char *)) __pyx_memview_get_double, (int (*)(char *, PyObject *)) __pyx_memview_set_double, 0);; if (unlikely(!__pyx_t_26)) __PYX_ERR(0, 426, __pyx_L1_error)
    __Pyx_GOTREF(__pyx_t_26);
    __pyx_t_1 = NULL;
    __pyx_t_2 = 0;
    if (CYTHON_UNPACK_METHODS && unlikely(PyMethod_Check(__pyx_t_17))) {
      __pyx_t_1 = PyMethod_GET_SELF(__pyx_t_17);
      if (likely(__pyx_t_1)) {
        PyObject* function = PyMethod_GET_FUNCTION(__pyx_t_17);
        __Pyx_INCREF(__pyx_t_1);
        __Pyx_INCREF(function);
        __Pyx_DECREF_SET(__pyx_t_17, function);
        __pyx_t_2 = 1;
      }
    }
    #if CYTHON_FAST_PYCALL
    if (PyFunction_Check(__pyx_t_17)) {
      PyObject *__pyx_temp[4] = {__pyx_t_1, __pyx_t_15, __pyx_t_26, __pyx_kp_s__3};
      __pyx_t_9 = __Pyx_PyFunction_FastCall(__pyx_t_17, __pyx_temp+1-__pyx_t_2, 3+__pyx_t_2); if (unlikely(!__pyx_t_9)) __PYX_ERR(0, 426, __pyx_L1_error)
      __Pyx_XDECREF(__pyx_t_1); __pyx_t_1 = 0;
      __Pyx_GOTREF(__pyx_t_9);
      __Pyx_DECREF(__pyx_t_15); __pyx_t_15 = 0;
      __Pyx_DECREF(__pyx_t_26); __pyx_t_26 = 0;
    } else
    #endif
    #if CYTHON_FAST_PYCCALL
    if (__Pyx_PyFastCFunction_Check(__pyx_t_17)) {
      PyObject *__pyx_temp[4] = {__pyx_t_1, __pyx_t_15, __pyx_t_26, __pyx_kp_s__3};
      __pyx_t_9 = __Pyx_PyCFunction_FastCall(__pyx_t_17, __pyx_temp+1-__pyx_t_2, 3+__pyx_t_2); if (unlikely(!__pyx_t_9)) __PYX_ERR(0, 426, __pyx_L1_error)
      __Pyx_XDECREF(__pyx_t_1); __pyx_t_1 = 0;
      __Pyx_GOTREF(__pyx_t_9);
      __Pyx_DECREF(__pyx_t_15); __pyx_t_15 = 0;
      __Pyx_DECREF(__pyx_t_26); __pyx_t_26 = 0;
    } else
    #endif
    {
      __pyx_t_16 = PyTuple_New(3+__pyx_t_2); if (unlikely(!__pyx_t_16)) __PYX_ERR(0, 426, __pyx_L1_error)
      __Pyx_GOTREF(__pyx_t_16);
      if (__pyx_t_1) {
        __Pyx_GIVEREF(__pyx_t_1); PyTuple_SET_ITEM(__pyx_t_16, 0, __pyx_t_1); __pyx_t_1 = NULL;
      }
      __Pyx_GIVEREF(__pyx_t_15);
      PyTuple_SET_ITEM(__pyx_t_16, 0+__pyx_t_2, __pyx_t_15);
      __Pyx_GIVEREF(__pyx_t_26);
      PyTuple_SET_ITEM(__pyx_t_16, 1+__pyx_t_2, __pyx_t_26);
      __Pyx_INCREF(__pyx_kp_s__3);
      __Pyx_GIVEREF(__pyx_kp_s__3);
      PyTuple_SET_ITEM(__pyx_t_16, 2+__pyx_t_2, __pyx_kp_s__3);
      __pyx_t_15 = 0;
      __pyx_t_26 = 0;
      __pyx_t_9 = __Pyx_PyObject_Call(__pyx_t_17, __pyx_t_16, NULL); if (unlikely(!__pyx_t_9)) __PYX_ERR(0, 426, __pyx_L1_error)
      __Pyx_GOTREF(__pyx_t_9);
      __Pyx_DECREF(__pyx_t_16); __pyx_t_16 = 0;
    }
    __Pyx_DECREF(__pyx_t_17); __pyx_t_17 = 0;
    __Pyx_DECREF(__pyx_t_9); __pyx_t_9 = 0;
```

```
+427:         plt.loglog(x_axis, y_axis, label = r"$1 / \sigma v z$ = {0}".format(round(slope, 2)))
```

```
    __pyx_t_9 = __Pyx_GetModuleGlobalName(__pyx_n_s_plt); if (unlikely(!__pyx_t_9)) __PYX_ERR(0, 427, __pyx_L1_error)
    __Pyx_GOTREF(__pyx_t_9);
    __pyx_t_17 = __Pyx_PyObject_GetAttrStr(__pyx_t_9, __pyx_n_s_loglog); if (unlikely(!__pyx_t_17)) __PYX_ERR(0, 427, __pyx_L1_error)
    __Pyx_GOTREF(__pyx_t_17);
    __Pyx_DECREF(__pyx_t_9); __pyx_t_9 = 0;
    __pyx_t_9 = __pyx_memoryview_fromslice(__pyx_v_x_axis, 1, (PyObject *(*)(char *)) __pyx_memview_get_double, (int (*)(char *, PyObject *)) __pyx_memview_set_double, 0);; if (unlikely(!__pyx_t_9)) __PYX_ERR(0, 427, __pyx_L1_error)
    __Pyx_GOTREF(__pyx_t_9);
    __pyx_t_16 = __pyx_memoryview_fromslice(__pyx_v_y_axis, 1, (PyObject *(*)(char *)) __pyx_memview_get_double, (int (*)(char *, PyObject *)) __pyx_memview_set_double, 0);; if (unlikely(!__pyx_t_16)) __PYX_ERR(0, 427, __pyx_L1_error)
    __Pyx_GOTREF(__pyx_t_16);
    __pyx_t_26 = PyTuple_New(2); if (unlikely(!__pyx_t_26)) __PYX_ERR(0, 427, __pyx_L1_error)
    __Pyx_GOTREF(__pyx_t_26);
    __Pyx_GIVEREF(__pyx_t_9);
    PyTuple_SET_ITEM(__pyx_t_26, 0, __pyx_t_9);
    __Pyx_GIVEREF(__pyx_t_16);
    PyTuple_SET_ITEM(__pyx_t_26, 1, __pyx_t_16);
    __pyx_t_9 = 0;
    __pyx_t_16 = 0;
    __pyx_t_16 = __Pyx_PyDict_NewPresized(1); if (unlikely(!__pyx_t_16)) __PYX_ERR(0, 427, __pyx_L1_error)
    __Pyx_GOTREF(__pyx_t_16);
    __pyx_t_15 = __Pyx_PyObject_GetAttrStr(__pyx_kp_s_1_sigma_v_z_0, __pyx_n_s_format); if (unlikely(!__pyx_t_15)) __PYX_ERR(0, 427, __pyx_L1_error)
    __Pyx_GOTREF(__pyx_t_15);
    __pyx_t_1 = PyFloat_FromDouble(__pyx_v_slope); if (unlikely(!__pyx_t_1)) __PYX_ERR(0, 427, __pyx_L1_error)
    __Pyx_GOTREF(__pyx_t_1);
    __pyx_t_29 = PyTuple_New(2); if (unlikely(!__pyx_t_29)) __PYX_ERR(0, 427, __pyx_L1_error)
    __Pyx_GOTREF(__pyx_t_29);
    __Pyx_GIVEREF(__pyx_t_1);
    PyTuple_SET_ITEM(__pyx_t_29, 0, __pyx_t_1);
    __Pyx_INCREF(__pyx_int_2);
    __Pyx_GIVEREF(__pyx_int_2);
    PyTuple_SET_ITEM(__pyx_t_29, 1, __pyx_int_2);
    __pyx_t_1 = 0;
    __pyx_t_1 = __Pyx_PyObject_Call(__pyx_builtin_round, __pyx_t_29, NULL); if (unlikely(!__pyx_t_1)) __PYX_ERR(0, 427, __pyx_L1_error)
    __Pyx_GOTREF(__pyx_t_1);
    __Pyx_DECREF(__pyx_t_29); __pyx_t_29 = 0;
    __pyx_t_29 = NULL;
    if (CYTHON_UNPACK_METHODS && likely(PyMethod_Check(__pyx_t_15))) {
      __pyx_t_29 = PyMethod_GET_SELF(__pyx_t_15);
      if (likely(__pyx_t_29)) {
        PyObject* function = PyMethod_GET_FUNCTION(__pyx_t_15);
        __Pyx_INCREF(__pyx_t_29);
        __Pyx_INCREF(function);
        __Pyx_DECREF_SET(__pyx_t_15, function);
      }
    }
    if (!__pyx_t_29) {
      __pyx_t_9 = __Pyx_PyObject_CallOneArg(__pyx_t_15, __pyx_t_1); if (unlikely(!__pyx_t_9)) __PYX_ERR(0, 427, __pyx_L1_error)
      __Pyx_DECREF(__pyx_t_1); __pyx_t_1 = 0;
      __Pyx_GOTREF(__pyx_t_9);
    } else {
      #if CYTHON_FAST_PYCALL
      if (PyFunction_Check(__pyx_t_15)) {
        PyObject *__pyx_temp[2] = {__pyx_t_29, __pyx_t_1};
        __pyx_t_9 = __Pyx_PyFunction_FastCall(__pyx_t_15, __pyx_temp+1-1, 1+1); if (unlikely(!__pyx_t_9)) __PYX_ERR(0, 427, __pyx_L1_error)
        __Pyx_XDECREF(__pyx_t_29); __pyx_t_29 = 0;
        __Pyx_GOTREF(__pyx_t_9);
        __Pyx_DECREF(__pyx_t_1); __pyx_t_1 = 0;
      } else
      #endif
      #if CYTHON_FAST_PYCCALL
      if (__Pyx_PyFastCFunction_Check(__pyx_t_15)) {
        PyObject *__pyx_temp[2] = {__pyx_t_29, __pyx_t_1};
        __pyx_t_9 = __Pyx_PyCFunction_FastCall(__pyx_t_15, __pyx_temp+1-1, 1+1); if (unlikely(!__pyx_t_9)) __PYX_ERR(0, 427, __pyx_L1_error)
        __Pyx_XDECREF(__pyx_t_29); __pyx_t_29 = 0;
        __Pyx_GOTREF(__pyx_t_9);
        __Pyx_DECREF(__pyx_t_1); __pyx_t_1 = 0;
      } else
      #endif
      {
        __pyx_t_30 = PyTuple_New(1+1); if (unlikely(!__pyx_t_30)) __PYX_ERR(0, 427, __pyx_L1_error)
        __Pyx_GOTREF(__pyx_t_30);
        __Pyx_GIVEREF(__pyx_t_29); PyTuple_SET_ITEM(__pyx_t_30, 0, __pyx_t_29); __pyx_t_29 = NULL;
        __Pyx_GIVEREF(__pyx_t_1);
        PyTuple_SET_ITEM(__pyx_t_30, 0+1, __pyx_t_1);
        __pyx_t_1 = 0;
        __pyx_t_9 = __Pyx_PyObject_Call(__pyx_t_15, __pyx_t_30, NULL); if (unlikely(!__pyx_t_9)) __PYX_ERR(0, 427, __pyx_L1_error)
        __Pyx_GOTREF(__pyx_t_9);
        __Pyx_DECREF(__pyx_t_30); __pyx_t_30 = 0;
      }
    }
    __Pyx_DECREF(__pyx_t_15); __pyx_t_15 = 0;
    if (PyDict_SetItem(__pyx_t_16, __pyx_n_s_label, __pyx_t_9) < 0) __PYX_ERR(0, 427, __pyx_L1_error)
    __Pyx_DECREF(__pyx_t_9); __pyx_t_9 = 0;
    __pyx_t_9 = __Pyx_PyObject_Call(__pyx_t_17, __pyx_t_26, __pyx_t_16); if (unlikely(!__pyx_t_9)) __PYX_ERR(0, 427, __pyx_L1_error)
    __Pyx_GOTREF(__pyx_t_9);
    __Pyx_DECREF(__pyx_t_17); __pyx_t_17 = 0;
    __Pyx_DECREF(__pyx_t_26); __pyx_t_26 = 0;
    __Pyx_DECREF(__pyx_t_16); __pyx_t_16 = 0;
    __Pyx_DECREF(__pyx_t_9); __pyx_t_9 = 0;
```

```
+428:         plt.xlabel("Given Lengths")
```

```
    __pyx_t_9 = __Pyx_GetModuleGlobalName(__pyx_n_s_plt); if (unlikely(!__pyx_t_9)) __PYX_ERR(0, 428, __pyx_L1_error)
    __Pyx_GOTREF(__pyx_t_9);
    __pyx_t_16 = __Pyx_PyObject_GetAttrStr(__pyx_t_9, __pyx_n_s_xlabel); if (unlikely(!__pyx_t_16)) __PYX_ERR(0, 428, __pyx_L1_error)
    __Pyx_GOTREF(__pyx_t_16);
    __Pyx_DECREF(__pyx_t_9); __pyx_t_9 = 0;
    __pyx_t_9 = __Pyx_PyObject_Call(__pyx_t_16, __pyx_tuple__4, NULL); if (unlikely(!__pyx_t_9)) __PYX_ERR(0, 428, __pyx_L1_error)
    __Pyx_GOTREF(__pyx_t_9);
    __Pyx_DECREF(__pyx_t_16); __pyx_t_16 = 0;
    __Pyx_DECREF(__pyx_t_9); __pyx_t_9 = 0;
/* … */
  __pyx_tuple__4 = PyTuple_Pack(1, __pyx_kp_s_Given_Lengths); if (unlikely(!__pyx_tuple__4)) __PYX_ERR(0, 428, __pyx_L1_error)
  __Pyx_GOTREF(__pyx_tuple__4);
  __Pyx_GIVEREF(__pyx_tuple__4);
```

```
+429:         plt.ylabel("Average Sizes")
```

```
    __pyx_t_9 = __Pyx_GetModuleGlobalName(__pyx_n_s_plt); if (unlikely(!__pyx_t_9)) __PYX_ERR(0, 429, __pyx_L1_error)
    __Pyx_GOTREF(__pyx_t_9);
    __pyx_t_16 = __Pyx_PyObject_GetAttrStr(__pyx_t_9, __pyx_n_s_ylabel); if (unlikely(!__pyx_t_16)) __PYX_ERR(0, 429, __pyx_L1_error)
    __Pyx_GOTREF(__pyx_t_16);
    __Pyx_DECREF(__pyx_t_9); __pyx_t_9 = 0;
    __pyx_t_9 = __Pyx_PyObject_Call(__pyx_t_16, __pyx_tuple__5, NULL); if (unlikely(!__pyx_t_9)) __PYX_ERR(0, 429, __pyx_L1_error)
    __Pyx_GOTREF(__pyx_t_9);
    __Pyx_DECREF(__pyx_t_16); __pyx_t_16 = 0;
    __Pyx_DECREF(__pyx_t_9); __pyx_t_9 = 0;
/* … */
  __pyx_tuple__5 = PyTuple_Pack(1, __pyx_kp_s_Average_Sizes); if (unlikely(!__pyx_tuple__5)) __PYX_ERR(0, 429, __pyx_L1_error)
  __Pyx_GOTREF(__pyx_tuple__5);
  __Pyx_GIVEREF(__pyx_tuple__5);
```

```
+430:         plt.title("Exponent Relation")
```

```
    __pyx_t_9 = __Pyx_GetModuleGlobalName(__pyx_n_s_plt); if (unlikely(!__pyx_t_9)) __PYX_ERR(0, 430, __pyx_L1_error)
    __Pyx_GOTREF(__pyx_t_9);
    __pyx_t_16 = __Pyx_PyObject_GetAttrStr(__pyx_t_9, __pyx_n_s_title); if (unlikely(!__pyx_t_16)) __PYX_ERR(0, 430, __pyx_L1_error)
    __Pyx_GOTREF(__pyx_t_16);
    __Pyx_DECREF(__pyx_t_9); __pyx_t_9 = 0;
    __pyx_t_9 = __Pyx_PyObject_Call(__pyx_t_16, __pyx_tuple__6, NULL); if (unlikely(!__pyx_t_9)) __PYX_ERR(0, 430, __pyx_L1_error)
    __Pyx_GOTREF(__pyx_t_9);
    __Pyx_DECREF(__pyx_t_16); __pyx_t_16 = 0;
    __Pyx_DECREF(__pyx_t_9); __pyx_t_9 = 0;
/* … */
  __pyx_tuple__6 = PyTuple_Pack(1, __pyx_kp_s_Exponent_Relation); if (unlikely(!__pyx_tuple__6)) __PYX_ERR(0, 430, __pyx_L1_error)
  __Pyx_GOTREF(__pyx_tuple__6);
  __Pyx_GIVEREF(__pyx_tuple__6);
```

```
+431:         plt.legend()
```

```
    __pyx_t_16 = __Pyx_GetModuleGlobalName(__pyx_n_s_plt); if (unlikely(!__pyx_t_16)) __PYX_ERR(0, 431, __pyx_L1_error)
    __Pyx_GOTREF(__pyx_t_16);
    __pyx_t_26 = __Pyx_PyObject_GetAttrStr(__pyx_t_16, __pyx_n_s_legend); if (unlikely(!__pyx_t_26)) __PYX_ERR(0, 431, __pyx_L1_error)
    __Pyx_GOTREF(__pyx_t_26);
    __Pyx_DECREF(__pyx_t_16); __pyx_t_16 = 0;
    __pyx_t_16 = NULL;
    if (CYTHON_UNPACK_METHODS && unlikely(PyMethod_Check(__pyx_t_26))) {
      __pyx_t_16 = PyMethod_GET_SELF(__pyx_t_26);
      if (likely(__pyx_t_16)) {
        PyObject* function = PyMethod_GET_FUNCTION(__pyx_t_26);
        __Pyx_INCREF(__pyx_t_16);
        __Pyx_INCREF(function);
        __Pyx_DECREF_SET(__pyx_t_26, function);
      }
    }
    if (__pyx_t_16) {
      __pyx_t_9 = __Pyx_PyObject_CallOneArg(__pyx_t_26, __pyx_t_16); if (unlikely(!__pyx_t_9)) __PYX_ERR(0, 431, __pyx_L1_error)
      __Pyx_DECREF(__pyx_t_16); __pyx_t_16 = 0;
    } else {
      __pyx_t_9 = __Pyx_PyObject_CallNoArg(__pyx_t_26); if (unlikely(!__pyx_t_9)) __PYX_ERR(0, 431, __pyx_L1_error)
    }
    __Pyx_GOTREF(__pyx_t_9);
    __Pyx_DECREF(__pyx_t_26); __pyx_t_26 = 0;
    __Pyx_DECREF(__pyx_t_9); __pyx_t_9 = 0;
```

```
 432:
```

```
+433:     return (slope, intercept, pval), given_lengths, avg_sizes
```

```
  __Pyx_XDECREF(__pyx_r);
  __pyx_t_9 = PyFloat_FromDouble(__pyx_v_slope); if (unlikely(!__pyx_t_9)) __PYX_ERR(0, 433, __pyx_L1_error)
  __Pyx_GOTREF(__pyx_t_9);
  __pyx_t_26 = PyFloat_FromDouble(__pyx_v_intercept); if (unlikely(!__pyx_t_26)) __PYX_ERR(0, 433, __pyx_L1_error)
  __Pyx_GOTREF(__pyx_t_26);
  __pyx_t_16 = PyFloat_FromDouble(__pyx_v_pval); if (unlikely(!__pyx_t_16)) __PYX_ERR(0, 433, __pyx_L1_error)
  __Pyx_GOTREF(__pyx_t_16);
  __pyx_t_17 = PyTuple_New(3); if (unlikely(!__pyx_t_17)) __PYX_ERR(0, 433, __pyx_L1_error)
  __Pyx_GOTREF(__pyx_t_17);
  __Pyx_GIVEREF(__pyx_t_9);
  PyTuple_SET_ITEM(__pyx_t_17, 0, __pyx_t_9);
  __Pyx_GIVEREF(__pyx_t_26);
  PyTuple_SET_ITEM(__pyx_t_17, 1, __pyx_t_26);
  __Pyx_GIVEREF(__pyx_t_16);
  PyTuple_SET_ITEM(__pyx_t_17, 2, __pyx_t_16);
  __pyx_t_9 = 0;
  __pyx_t_26 = 0;
  __pyx_t_16 = 0;
  __pyx_t_16 = __pyx_memoryview_fromslice(__pyx_v_given_lengths, 1, (PyObject *(*)(char *)) __pyx_memview_get_double, (int (*)(char *, PyObject *)) __pyx_memview_set_double, 0);; if (unlikely(!__pyx_t_16)) __PYX_ERR(0, 433, __pyx_L1_error)
  __Pyx_GOTREF(__pyx_t_16);
  __pyx_t_26 = __pyx_memoryview_fromslice(__pyx_v_avg_sizes, 1, (PyObject *(*)(char *)) __pyx_memview_get_double, (int (*)(char *, PyObject *)) __pyx_memview_set_double, 0);; if (unlikely(!__pyx_t_26)) __PYX_ERR(0, 433, __pyx_L1_error)
  __Pyx_GOTREF(__pyx_t_26);
  __pyx_t_9 = PyTuple_New(3); if (unlikely(!__pyx_t_9)) __PYX_ERR(0, 433, __pyx_L1_error)
  __Pyx_GOTREF(__pyx_t_9);
  __Pyx_GIVEREF(__pyx_t_17);
  PyTuple_SET_ITEM(__pyx_t_9, 0, __pyx_t_17);
  __Pyx_GIVEREF(__pyx_t_16);
  PyTuple_SET_ITEM(__pyx_t_9, 1, __pyx_t_16);
  __Pyx_GIVEREF(__pyx_t_26);
  PyTuple_SET_ITEM(__pyx_t_9, 2, __pyx_t_26);
  __pyx_t_17 = 0;
  __pyx_t_16 = 0;
  __pyx_t_26 = 0;
  __pyx_r = __pyx_t_9;
  __pyx_t_9 = 0;
  goto __pyx_L0;
```

```
 434:
```

```
 435: @cython.wraparound(False)
```

```
 436: @cython.boundscheck(False)
```

```
 437: @cython.cdivision(True)
```

```
+438: def branching_ratio(object[:] X):
```

```
/* Python wrapper */
static PyObject *__pyx_pw_10avalanches_15branching_ratio(PyObject *__pyx_self, PyObject *__pyx_arg_X); /*proto*/
static char __pyx_doc_10avalanches_14branching_ratio[] = "\n    Returns the branching ratio of a series of avalanches. \n    Found by averaging the ratio of the first to second events in all avalanches\n    ";
static PyMethodDef __pyx_mdef_10avalanches_15branching_ratio = {"branching_ratio", (PyCFunction)__pyx_pw_10avalanches_15branching_ratio, METH_O, __pyx_doc_10avalanches_14branching_ratio};
static PyObject *__pyx_pw_10avalanches_15branching_ratio(PyObject *__pyx_self, PyObject *__pyx_arg_X) {
  __Pyx_memviewslice __pyx_v_X = { 0, 0, { 0 }, { 0 }, { 0 } };
  PyObject *__pyx_r = 0;
  __Pyx_RefNannyDeclarations
  __Pyx_RefNannySetupContext("branching_ratio (wrapper)", 0);
  assert(__pyx_arg_X); {
    __pyx_v_X = __Pyx_PyObject_to_MemoryviewSlice_ds_object(__pyx_arg_X, PyBUF_WRITABLE); if (unlikely(!__pyx_v_X.memview)) __PYX_ERR(0, 438, __pyx_L3_error)
  }
  goto __pyx_L4_argument_unpacking_done;
  __pyx_L3_error:;
  __Pyx_AddTraceback("avalanches.branching_ratio", __pyx_clineno, __pyx_lineno, __pyx_filename);
  __Pyx_RefNannyFinishContext();
  return NULL;
  __pyx_L4_argument_unpacking_done:;
  __pyx_r = __pyx_pf_10avalanches_14branching_ratio(__pyx_self, __pyx_v_X);

  /* function exit code */
  __Pyx_RefNannyFinishContext();
  return __pyx_r;
}

static PyObject *__pyx_pf_10avalanches_14branching_ratio(CYTHON_UNUSED PyObject *__pyx_self, __Pyx_memviewslice __pyx_v_X) {
  int __pyx_v_N;
  double __pyx_v_ratio;
  double __pyx_v_event_0;
  double __pyx_v_event_1;
  __Pyx_memviewslice __pyx_v_avalanche = { 0, 0, { 0 }, { 0 }, { 0 } };
  int __pyx_v_i;
  double __pyx_v_sigma;
  PyObject *__pyx_r = NULL;
  __Pyx_RefNannyDeclarations
  __Pyx_RefNannySetupContext("branching_ratio", 0);
/* … */
  /* function exit code */
  __pyx_L1_error:;
  __Pyx_XDECREF(__pyx_t_4);
  __Pyx_XDECREF(__pyx_t_6);
  __PYX_XDEC_MEMVIEW(&__pyx_t_7, 1);
  __Pyx_AddTraceback("avalanches.branching_ratio", __pyx_clineno, __pyx_lineno, __pyx_filename);
  __pyx_r = NULL;
  __pyx_L0:;
  __PYX_XDEC_MEMVIEW(&__pyx_v_X, 1);
  __PYX_XDEC_MEMVIEW(&__pyx_v_avalanche, 1);
  __Pyx_XGIVEREF(__pyx_r);
  __Pyx_RefNannyFinishContext();
  return __pyx_r;
}
/* … */
  __pyx_tuple__53 = PyTuple_Pack(9, __pyx_n_s_X, __pyx_n_s_X, __pyx_n_s_N, __pyx_n_s_ratio, __pyx_n_s_event_0, __pyx_n_s_event_1, __pyx_n_s_avalanche, __pyx_n_s_i, __pyx_n_s_sigma); if (unlikely(!__pyx_tuple__53)) __PYX_ERR(0, 438, __pyx_L1_error)
  __Pyx_GOTREF(__pyx_tuple__53);
  __Pyx_GIVEREF(__pyx_tuple__53);
/* … */
  __pyx_t_2 = PyCFunction_NewEx(&__pyx_mdef_10avalanches_15branching_ratio, NULL, __pyx_n_s_avalanches); if (unlikely(!__pyx_t_2)) __PYX_ERR(0, 438, __pyx_L1_error)
  __Pyx_GOTREF(__pyx_t_2);
  if (PyDict_SetItem(__pyx_d, __pyx_n_s_branching_ratio, __pyx_t_2) < 0) __PYX_ERR(0, 438, __pyx_L1_error)
  __Pyx_DECREF(__pyx_t_2); __pyx_t_2 = 0;
  __pyx_codeobj__54 = (PyObject*)__Pyx_PyCode_New(1, 0, 9, 0, CO_OPTIMIZED|CO_NEWLOCALS, __pyx_empty_bytes, __pyx_empty_tuple, __pyx_empty_tuple, __pyx_tuple__53, __pyx_empty_tuple, __pyx_empty_tuple, __pyx_kp_s_avalanches_pyx, __pyx_n_s_branching_ratio, 438, __pyx_empty_bytes); if (unlikely(!__pyx_codeobj__54)) __PYX_ERR(0, 438, __pyx_L1_error)
```

```
 439:     """
```

```
 440:     Returns the branching ratio of a series of avalanches.
```

```
 441:     Found by averaging the ratio of the first to second events in all avalanches
```

```
 442:     """
```

```
+443:     cdef int N = X.shape[0]
```

```
  __pyx_v_N = (__pyx_v_X.shape[0]);
```

```
+444:     cdef double ratio = 0
```

```
  __pyx_v_ratio = 0.0;
```

```
 445:     cdef double event_0, event_1
```

```
 446:     cdef double[:] avalanche
```

```
 447:     cdef int i
```

```
 448:
```

```
+449:     for i in range(N):
```

```
  __pyx_t_1 = __pyx_v_N;
  __pyx_t_2 = __pyx_t_1;
  for (__pyx_t_3 = 0; __pyx_t_3 < __pyx_t_2; __pyx_t_3+=1) {
    __pyx_v_i = __pyx_t_3;
```

```
+450:         avalanche = X[i].astype("double")
```

```
    __pyx_t_5 = __pyx_v_i;
    __pyx_t_4 = (PyObject *) *((PyObject * *) ( /* dim=0 */ (__pyx_v_X.data + __pyx_t_5 * __pyx_v_X.strides[0]) ));
    __Pyx_INCREF((PyObject*)__pyx_t_4);
    __pyx_t_6 = __Pyx_PyObject_GetAttrStr(__pyx_t_4, __pyx_n_s_astype); if (unlikely(!__pyx_t_6)) __PYX_ERR(0, 450, __pyx_L1_error)
    __Pyx_GOTREF(__pyx_t_6);
    __Pyx_DECREF(__pyx_t_4); __pyx_t_4 = 0;
    __pyx_t_4 = __Pyx_PyObject_Call(__pyx_t_6, __pyx_tuple__7, NULL); if (unlikely(!__pyx_t_4)) __PYX_ERR(0, 450, __pyx_L1_error)
    __Pyx_GOTREF(__pyx_t_4);
    __Pyx_DECREF(__pyx_t_6); __pyx_t_6 = 0;
    __pyx_t_7 = __Pyx_PyObject_to_MemoryviewSlice_ds_double(__pyx_t_4, PyBUF_WRITABLE); if (unlikely(!__pyx_t_7.memview)) __PYX_ERR(0, 450, __pyx_L1_error)
    __Pyx_DECREF(__pyx_t_4); __pyx_t_4 = 0;
    __PYX_XDEC_MEMVIEW(&__pyx_v_avalanche, 1);
    __pyx_v_avalanche = __pyx_t_7;
    __pyx_t_7.memview = NULL;
    __pyx_t_7.data = NULL;
/* … */
  __pyx_tuple__7 = PyTuple_Pack(1, __pyx_n_s_double); if (unlikely(!__pyx_tuple__7)) __PYX_ERR(0, 450, __pyx_L1_error)
  __Pyx_GOTREF(__pyx_tuple__7);
  __Pyx_GIVEREF(__pyx_tuple__7);
```

```
+451:         event_0 = avalanche[0]
```

```
    __pyx_t_8 = 0;
    __pyx_v_event_0 = (*((double *) ( /* dim=0 */ (__pyx_v_avalanche.data + __pyx_t_8 * __pyx_v_avalanche.strides[0]) )));
```

```
+452:         event_1 = avalanche[1]
```

```
    __pyx_t_9 = 1;
    __pyx_v_event_1 = (*((double *) ( /* dim=0 */ (__pyx_v_avalanche.data + __pyx_t_9 * __pyx_v_avalanche.strides[0]) )));
```

```
+453:         if event_1 != 0:
```

```
    __pyx_t_10 = ((__pyx_v_event_1 != 0.0) != 0);
    if (__pyx_t_10) {
/* … */
    }
  }
```

```
+454:             ratio += (event_0 / event_1)
```

```
      __pyx_v_ratio = (__pyx_v_ratio + (__pyx_v_event_0 / __pyx_v_event_1));
```

```
 455:
```

```
+456:     cdef double sigma = ratio / N
```

```
  __pyx_v_sigma = (__pyx_v_ratio / __pyx_v_N);
```

```
 457:
```

```
+458:     return sigma
```

```
  __Pyx_XDECREF(__pyx_r);
  __pyx_t_4 = PyFloat_FromDouble(__pyx_v_sigma); if (unlikely(!__pyx_t_4)) __PYX_ERR(0, 458, __pyx_L1_error)
  __Pyx_GOTREF(__pyx_t_4);
  __pyx_r = __pyx_t_4;
  __pyx_t_4 = 0;
  goto __pyx_L0;
```
