# Supplementary material for: Differential effects of propofol and ketamine on critical brain dynamics
Source: PLoS Comput Biol. 2020 Dec 21;16(12):e1008418. doi: 10.1371/journal.pcbi.1008418 (PMC7785236; doi:10.1371/journal.pcbi.1008418)
Supplement: Attachment — Submitted filename: response_to_reviewers_v3.pdf [file pcbi.1008418.s003.pdf]

To the Editors and Reviewers,
We sincerely thank you for taking the time to go through our submission so carefully, and for giving us your thoughtful and insightful feedback. Please find our responses below describing the revisions we have made to our manuscript. Our responses to specific comments appear in blue italics, wheras the original reviewer comments are presented in un-italicized black font. On behalf of all Authors,

We send our best wishes to you to remain safe and well during the Covid-19 pandemic.
~Thomas Varley

Dr. Jbabdi:
The three reviewers raise a number of concerns, but two of them are particularly salient: (1) is the work sufficiently novel.

We believe that the work presented here is novel in several respects. First, the most significant is the combination of criticality analysis and information dynamics in the context of consciousness research. While both dimensions (criticality and information theory) have been applied to questions about anesthesia previously, the intersection of the two is novel, allowing us to present a more complete interpretation of the issue. Empirical evidence that criticality maximizes complexity in neural tissue was first shown in 2016 (Timme et al., 2016 Front. Physiol.) and work in this vein represents a potentially useful bridge between information-theoretic analysis of brain activity, and dynamical systems analysis of brain activity. Consistent with Reviewer 3's favorable comments, while previous work has shown increases in the "complexity" of brain activity following ketamine administration, to the best of our knowledge, we are the first group to bring criticality analysis to bear on this question. As discussed below, previous work has typically relied on univariate, scalar measures, such as Lempel-Ziv complexity, which while informative, do not provide as rich or detailed an understanding of the different dynamical regimes as the criticality analysis does. An example of this was picked up by Reviewer 2, who noted that one novel finding in this paper is the restricted range over which the powerlaw distribution holds, which in turn informs on how coordinated activity across the cortex is behaving. This provides dynamical, spatial, and temporal insights beyond what could be extracted from something like a channel-wise Lempel-Ziv analysis.

Second, while previous studies have shown changes to critical dynamics following propofol and isoflurane anesthesia, this study applies a novel, and more robust, pipeline for criticality inference than has been used in the past. By applying multiple tests, such as exponent relations, shape collapse, and renormalization across temporal bins, we are able to infer a much more robust picture of where our data maintains critical dynamics, and where it deviates from them. We believe that our battery represents the current gold-standard in terms of criticality inference.

*Finally, we hold that the measure of information complexity we use (TSE complexity) is unique in several respects, when compared to other commonly-used measures of complexity in consciousness research. Typical measures used include Lempel-Ziv complexity and Shannon entropy which, while informative, are distinct from TSE complexity. While measures like LZ complexity and Shannon entropy are best understood as measures of "randomness" or "incompressibility", TSE complexity provides information about **the relative balance** of integration and segregation in the system under study – a topic of key interest in the field of consciousness research. While Shannon entropy and LZ complexity are high when the data are*

incompressible, TSE complexity is high when the system shows both integration and segregation. Consequently, while the result that the Awake condition is high relative to anesthesia may appear to just be a replication of earlier work (e.g. Schartner et al., 2015., PLOS ONE), we feel that the insights gleaned from using TSE complexity provides additional information above-and-beyond what has been reported previously relevant to the specific information-dynamics at play in the system (e.g. integrated activity vs. segregated activity).

(2) can conclusions be drawn from such a small sample size?

The Reviewers are correct that the small sample size represents a significant limitation of this research, and not one that can be easily addressed, unfortunately, due the inherent limitations in the dataset we are using. That said, despite this issue, we believe several arguments can be made in favor of the validity of these results. First, while our methods are novel (as described above), our results are broadly consistent with previous work in the field – if the results had been wildly inconsistent with previous work, that would be a major concern, however given previous work, we feel it is appropriate to leverage reasonably strong priors when assessing these results.

Second, results from the NeuroTycho project using this small number of macaques have been published in a number of journals, including PLoS Computational Biology. For example, Toker & Sommer (2019) published work using these two monkeys. Other examples using the 2 macaque NeuroTycho dataset include Ma et al., (2019, Anesthesiology), and Muthukamaraswamy & Liley (2018 – NeuroImage). The literature around this kind of invasive primate recording typically has smaller sample sizes than would be expected in other animal models or in non-invasive human neuroimaging studies (for an example not from NeuroTycho, see also Hu et al., (2018, Scientific Reports) who similarly had an N of 2).

Finally, while the N is low, in terms of the absolute number of subjects, the recordings that we do have have been very densely sampled, and span a considerable timeframe. For each condition the recordings were performed at a very high sampling rate (1KHz) and lasted for multiple minutes, resulting in several million individual samples of cortical activity across the array. This provides a rich statistical dataset to extract data from and allows us to be very confident in the particular distributions we construct.

In our paper, we have already highlighted this specific limitation, particularly stressing the need for further replication. Despite these limitations, however, we still feel confident in asserting that these results represent a meaningful addition to the scientific literature around the issues of consciousness, criticality, and information dynamics.

Toker, D., & Sommer, F. T. (2019). Information integration in large brain networks. PLOS Computational Biology, 15(2), e1006807. https://doi.org/10.1371/journal.pcbi.1006807

Ma, L., Liu, W., & Hudson, A. E. (2019). Propofol Anesthesia Increases Long-range Frontoparietal Corticocortical Interaction in the Oculomotor Circuit in Macaque Monkeys. Anesthesiology: The Journal of the American Society of Anesthesiologists, 130(4), 560–571. https://doi.org/10.1097/ALN.0000000000002637

Muthukumaraswamy, S. D., & Liley, D. TJ. (2018). 1/f electrophysiological spectra in resting and drug-induced states can be explained by the dynamics of multiple oscillatory relaxation processes. NeuroImage, 179, 582–595. https://doi.org/10.1016/j.neuroimage.2018.06.068

Hu, K., Jamali, M., Moses, Z. B., Ortega, C. A., Friedman, G. N., Xu, W., & Williams, Z. M. (2018). Decoding unconstrained arm movements in primates using high-density electrocorticography signals for brain-machine interface use. Scientific Reports, 8(1), 10583. https://doi.org/10.1038/s41598-018-28940-7

We were also requested to note that, where we had a reference of "data not shown", we now have expanded on the results in question, removing the dissallowed reference.

Reviewer #1:

Using ECoG recordings in a macaque brain, this study investigated the effect of different anesthetics (propofol and ketamine) on critical brain dynamics. Consistent with different mechanisms of the two anesthetics, they found propofol, but not ketamine, dramatically restricted the size and duration of avalanches, as well as a large reduction in the complexity of brain dynamics. Overall, I think this is a well-conducted study. The methodology is sound, the conclusion helps to improve our understanding of altered states of consciousness and brain dynamics.

My main concern is about the small sample size. I agree with the authors about the rarity of the dataset, but with only N=4 for wake sessions and N=2 for propofol and ketamine, I think the results with mean+/-SD is inappropriate and somewhat misleading. I would suggest changing the descriptive statistics in the main text (perhaps only mean, or the range like minimum-maximum?), and moving the individual session results in supplemental table 1 to main text for clarity.

In With regard to the small number of animals, we agree with the reviewer that is a fundamental limitation, that we cannot control. That said, we have already noted above that the previous work that has been published with the 2 macaque NeuroTycho dataset.

Ma, L., Liu, W., & Hudson, A.E. (2019). Propfol Anesthesia Increases Long-range Frotoparietal Corticocortical Interaction in the Oculomotor Circuit in Macaque Monkeys. Anesthesiology,

Due to the resource constraints on this kind of invasive primate work, low Ns are more common than is expected relative to other animal models.

As we have also already mentioned above, the recordings have are very densely sampled (using 1 KHz), and span a considerable timeframe.

Changing the reporting of results is a good suggestion – we now show just the means (with no standard deviations), and have constructed a Table with all results from each scan.

Line 122. Please clearly specify the number of scans for each condition (awake, propofol, ketamine).

Thank you for catching this oversight! We have added the following text, starting on line 123:

> *“There were a total of four scans in the Awake condition, and two each in the Propofol and Ketamine conditions (each anaesthesia condition having it's own associated Awake scan).”*

Line 132. Please specify the sampling frequency of the data.

We have added text lines regarding the sample rate (1 KHz recording, with no downsampling during preprocessing) at the relevant line number and in the Data Acquisition section of Methods.

Line 156-157. The description of the method is not clear. For example, which correlation function was used? Is ρ correlation coefficient? What do t_min and t_max mean?

We have clarified the notation starting on line 156 to now read:

> “For all excursions above σ, the global maxima of the excursion was set to 1 and all other moments set to 0. We calculated the Pearson correlation coefficient ρ from the beginning of the excursion (t_{min}) to the end point of the excursion (t_{max}) against the same range in every other channel, and if $\rho \ge 0.75$, we also set the local maximum of the interval in the associated channel to 1 (even if it did not cross our threshold σ)}”

We hope that this now makes clear that t_min and t_max refer to respective beginning and endpoints of the excursion above sigma, while rho is specifically the Pearson correlation coefficient between the two slices of time-series.

Line 192. So, x denotes avalanche size or duration, and minimum and maximum values of x, which were named as x_min and x_max in line 234? Please clarify.

Here, x, x_min, and x_max refer to any elements in a distribution. We have added a line stating that x is a general variable and can refer either to avalanche sizes or durations, depending on the context. Line 202:

> “...the MLE value of the exponent, the minimum and maximum values of \textit{x} for which the power law estimate holds \added{(x_{min} and x_{max}, where \textit{x} can refer either to avalanche sizes or durations)}, and the \textit{p}-value. \added{It is crucial to note that x_{min} and x_{max} do \textit{not} refer to the smallest and largest values of x in the empirical distribution, but rather, the minimum and maximum values between which a powerlaw fit plausibly holds.”

Line 319. For avalanche size distribution, with only 128 channels of ECoG, how to get the maximum values of 378? Was this derived from fitted data or empirical data?

An avalanche size can exceed the number of channels if some channels are activated multiple times during a single avalanche. This is relatively rare, but occurs almost always in large data sets. See for example (Beggs and Plenz, 2003) where avalanches for local field potentials were first presented. The electrode array size was 60, but some avalanches were as large as 100.

Beggs, J. M., & Plenz, D. (2003). Neuronal Avalanches in Neocortical Circuits. Journal of Neuroscience, 23(35), 11167–11177. https://doi.org/10.1523/JNEUROSCI.23-35-11167.2003

Figures 7, 8 and 10 showed the results from two scans? Please appropriately describe this in the legend. To the plot legends of each of the three Figures, we have now added the explicit line:

> “Each plot includes two recordings: the anaesthesia condition (Propofol or Ketamine) and the associated pre-anaesthesia Awake condition from the same monkey during the same session.”

Line 516. I would suggest a brief discussion on the effect of eyes open on awake results, and on the observed difference between awake and unresponsive conditions (propofol/ketamine).

This is an interesting question. Thank you for this suggestion. We do not necessarily have strong intuitions about this issue, but we have included some speculation in the Discussion addressing this as an issue worthy of further study (Line 592):

> *“There is also a question about how the difference between the open eyes in the awake condition, compared to the closed eyes in the anaesthesia conditions, may be affecting neural dynamics. It could be argued that the awake state may be less stationary than either anaesthesia states, as the monkey is still able to engage with it's environment despite the restraints. The documentation does not make it clear how much potentially stimulating activity was taking place during the period of awake recording, although the monkey is described as "calmly sitting for periods up to 20 minutes" (http://wiki.neurotycho.org/Anesthesia_and_Sleep_Task_Details). While this may be considered a limitation, we argue that alertness and responsiveness to the environment is actually a key component to understanding ``normal" waking consciousness. Awareness of, and responsiveness to, the environment is a key element of what it means to be conscious, and so while the effects of incoming stimuli on neural data remain a fascinating outstanding question, we do not think that their presence is incompatible with our goal of understanding the differences between these three states of consciousness.”*

Reviewer #2:

The authors studied the differential effects of propofol and ketamine on the critical brain dynamics of a single macaque. A previously validated test of criticality, avalanche dynamics, was applied to analyze ECoG data of propofol and ketamine that induce differentiable effects on consciousness. Many previous studies with human and animal subjects suggested that maintenance of critical dynamics is necessary for the emergence of consciousness. However, controversially, some studies also demonstrated criticality in unconscious states. Thus, the authors tried to fill the knowledge gap and showed that propofol dramatically restricted the size and duration of avalanches, while ketamine allowed for more awake-like dynamics to persist. And propofol produces a dramatic reduction in the complexity but all states show some signs of persistent criticality. The author concluded that maintenance of critical brain dynamics may be important for regulation and control of conscious awareness.

The paper was well written and published timely. The authors provided proper background and relevant knowledge. The novelty and the need for this study appear clearly. I think this study may provide many insights to the researchers in this research field especially on the controversial findings of criticality in conscious and unconscious states.

Despite this paper was well written, I feel this study has a serious limitation that has already been mentioned by the authors. That is, all the results were drawn only from a single monkey. Even though several ECoG recordings were analyzed for two different anesthetics, still I doubt whether the results could be reproducible with other subjects.

Thank you for recognizing our manuscript as constituting a novel contribution to the literature.

We have already noted that using the data from a single monkey is a legitimate concern shared by other Reviewers and have responded above to these criticisms in detail, as well as tackling this issue explicitly in the manuscript itself. In our careful scrutiny of the datasets, we felt that there were artifacts in the second dataset that would have undermined the types of analyses that we performed in the current manuscript.

Considering the ambiguity of determining consciousness which mainly depends on the subject's responsiveness, in particular, with monkey it is worse than human subject. The authors cannot completely get rid of the possibility that the macaque was in covert consciousness. The subject could have covert consciousness if the dose of propofol was not enough to induce deep anesthesia or the anesthetic concentration was not maintained during the ECoG recording (in this study, the concentration was not maintained), or if the level of consciousness fluctuated after the initial induction, the subject could have woken up for a moment. Because of these possibilities, the authors did not remove, it seems difficult the authors can conclude that the signs of criticality were observed in the unconscious state. Without gathering more subjects, it would be difficult to improve this critical limitation.

With regard to the issue of "covert consciousness", we agree that this is potentially problematic. That said, however, it is not unique to our study and is an inherent limitation of the 2 macaque NeuroTycho dataset itself. The data themselves come timestamped to note the beginning of anesthetic injection, onset of surgical anesthesia, and onset of emergence. Hence, we are dependent on the expertise of the original researchers for epoching the data. (This problem is not unique to monkey anaesthesia – human surgical anaesthesia has had the perennial

issue of potential patient intraoperative awareness, where a conscious individual cannot adequately signal that they are awake, because they are routinely given a muscle paralysis agent as part of the anaesthesia protocol.)

Reviewer #3:

Varley and colleagues investigated the properties of non-human primate ECoG data from the perspective of critical dynamics, with the hypothesis that consciousness (perhaps more adequately, responsiveness) would correlate with critical behaviour. They found some evidence supporting that propofol (but not ketamine) disrupts critical dynamics.

While this direction of research is interesting, the results are hardly novel at this stage. The authors cited several papers showing a departure from critical dynamics induced by general anesthetics, including propofol. This extends to other states of reduced consciousness, such as slow wave sleep or epileptic seizures. Perhaps the most interesting finding is that ketamine resembled wakefulness; although this could also be expected from previous work, I'm not aware it has been shown from the perspective of criticality.

We have already commented above in detail to Reviewer 1 regarding the issue of novelty, so will not repeat our arguments here.

Besides this potential novelty issue, I have the following comments for the authors:

- The use of the word "exotic" to refer to non-ordinary/altered states of consciousness is somewhat strange... is there a reason not to stick to the common nomenclature?

In the past, we have encountered “exotic states of consciousness” in the literature referring to those altered states of consciousness which are not typically experienced in the course of normal life. That said, the Reviewer’s point is well-taken. We have replaced the term with a more standard expression such as “altered states of consciousness”.

- I checked ref. 25 and it does not include analysis of ECoG recordings acquired under the effect of psychedelics - this would have been somewhat strange, I think, since invasive recordings tend to be acquired in neurological patients only.

Thank you for finding this ambiguity in our manuscript. Muthukamaraswamy et al., do include EcoG from the NeuroTycho dataset, but not for any serotonergic psychedelics. We have updated the text accordingly.

- The NeuroTycho dataset has ECoG data acquired during other potentially interesting states of reduced awareness, which, if analyzed by the authors, could contribute to broaden the scope of their conclusions. I'm thinking of the sleep and ketamine plus medetomidine datasets. Is there a reason to exclude these datasets and to focus on the ketamine and propofol recordings only?

This is an interesting suggestion, which we considered quite a bit at the beginning of the project. Ultimately, however, we decided to select the data relating to only propofol and ketamine for two reasons: 1) both conditions represent a single drug intervention, in contrast to the ketamine and medetomidine conditions, where we would have had to address the added complexity of a second, adrenergic compound, as well as any interactions that could occur between the two of them. 2) The ketamine and propofol conditions were accompanied by a well-defined, behavioral test of unresponsiveness (nose-tickle. In contrast, during the sleep ‘task’, the monkeys moved in and out of slow-wave sleep throughout that ‘task’, making it difficult to discretize the two conditions. Critically, the

avalanche inference we used requires a long period of continuous, ideally stationary dynamics to robustly infer avalanche dynamics. We would not in the sleep condition the monkeys were moving in and out of slow-wave sleep.

- The following hypothesis "propofol would dramatically reduce signs of critical dynamics, but that criticality would persist under the influence of ketamine" is reasonable for sub-anesthetic doses of ketamine, but that is not so clear for anesthetic doses (at least not from the previous paragraphs of the introduction)

  This hypothesis was based on the phenomenon of "ketamine dreams", or the persistence of dream-like, or hallucinatory states of consciousness under the influence of ketamine reported by patients (and discussed in the Introduction). We have expanded the enumerated hypothesis in the Introduction to make this more explicit (Line 97):

  > *"This is based on the phenomena of "ketamine dreams" discussed above: based on the persistence of phenomenological consciousness under ketamine, we would signs of consciousness-like dynamics to persist under ketamine but not necessarily under propofol."*

- Perhaps the authors should invest additional efforts in model comparison, for instance, how do log normal, exponential, and exponential cutoff models reproduce the avalanche distributions, and how do the goodness of fit compare to those seen for power laws?

  Thank you for this practical suggestion. We did consider more detailed model comparisons during the project, but we ran into some technical limitations in that the NCC Toolbox we used does not directly support this kind of model comparison. On the other hand, other tools had their own issues (for instance, other tools we looked at did not support variable values of x_min and x_max), which made comparison almost impossible (since different criteria are being used to assess the power-law fit).

- "To avoid interminable run-times, the NCC Toolbox [49] includes several corrections for sub-sampling and heuristics for estimating integration in large systems" -> Maybe some information concerning those heuristics, to make the manuscript more self-contained?

  We have added the following text to the relevant Methods section (Line 292):

  > *"One correction is to only consider those bins where at least one "event" occurs, consequently calculating the complexity of the avalanches themselves, which controls for variable numbers and distances between avalanches. Furthermore, the NCC Toolbox corrects for sub-sampling biases in the joint probability distribution by comparing the empirical integrations to an ensemble of time-randomized null models and subtracting the expected value of the distribution of null integrations and optimizing on the subsets of size k that are most informative about the structure of the system. By implementing these corrections, the toolbox is able to infer the multi-scale integration/segregation structure without having to brute-force all possible bipartitions of a sparse multi-dimensional time-series."*

- "Visual inspection shows that the ketamine condition tracks the Awake condition much more closely than the propofol condition does" -> Sorry, I really can't see this by visual inspection. I know the authors checked the stats on the manuscript text, but perhaps they should make one or more new figures where these are visualized. For example, the values of the exponents are scattered throughout the

text and this makes it difficult to draw quick comparisons. Here a figure could help.

We apologize for the lack of clarity here. Our writing was unclear. By using the word "tracks" in this context, we were speaking specifically of the CCDF plots (Figure 7), where (we hope) it was clear that the Propofol condition dropped below the Awake condition almost immediately, while the Ketamine distributions remained close to their associated Awake distributions through the first order of magnitude. We have elaborated and clarified this in the associated figure legend. We hope that this revision now resolves this issue.

The point about values being scattered around is well-taken, however! To this end, we moved the individual results into the main text from their original place in Supplementary Information (.csv table). Now all the results are accessible in one location, and should be able to be visually compared more easily.

- "We had hypothesized that the Awake and/or ketamine conditions would show the highest degree of concurrence between the measures (reflecting a greater degree of criticality), but instead, the Awake condition has the lowest degree of concurrence" -> I think this deserves more discussion.

  We agree that is certainly one of the more interesting results of the study. We appreciate the opportunity to expund on it further, and have included the following discussion in the Scaling Exponents subsection of the Results:

  > *"The significance of this is difficult to explain. One possibility is that the Awake condition is noisier than either anaesthesia conditions, which would reduce the critical fitness. Alternately, the Awake state may be less stationary than either anaesthesia states, as the monkey is still able to engage with it's environment despite the restraints. Finally, we note that, while concurrence is highest in the Propofol condition, it is over a much narrower range of x values for both avalanche sizes and durations, as opposed to the Awake and Ketamine conditions and so the higher concurrence may be reflective of the more restricted range with fewer degrees of freedom (for further discussion of this, see the Discussion)."*

- "Based on these, we propose that the brain is able to support critical dynamics in all three states, but that propofol (but not ketamine) reduces the scale over which critical dynamics can occur." -> At this point, I think you need to delve deeper into how this "partial" scale-free dynamics relate to known results from statistical physics. What kind of system would show critical dynamics over a restricted range, and is the brain such a system?

  We are glad to further elaborate on this issue and have added the following to the Discussion on this point:

  > *"It is worth unpacking how this restriction might play out in the brain. Two dynamical changes might restrict the range over which the power-law held. An increase in high-frequency noise will have the effect of driving a deviation from power-law scaling at the upper end of the distribution, while limiting the diverging correlation length will drive a deviation from power-laws at the lower end of the tail. By examining how the x_{min} and x_{max} values change between conditions, we can better understand the changing dynamics. In the Propofol condition, for both avalanche sizes and durations, the values of x_{min} and x_{max} are shifted up the distribution, so the power-law fit begins and ends with smaller, shorter lived avalanches. This could be consistent with both a reduction in high-frequency noise, as well as a decreasing correlation length, both of which are consistent with other, well*

established elements of propofol anaesthesia. As previously mentioned, a leading hypothesis is that anaesthetics like propofol ``fragment" brain networks \cite{lee_frontoparietal_2009,hudetz_disconnecting_2016,lewis_rapid_2012}, or alternately ``mute" the flow of information \cite{areshenkoff_muting_2020} between regions. In the context of functional connectivity analysis (a core element of many of these analysis), decreased connectivity can be directly related to a decrease in correlation length, which is consistent with the loss of power-law behaviour in the tails of the distributions. The decrease in highfrequency noise is likely associated with the increase in high-power, lowfrequency oscillations that characterize the state induced by propofol \cite{hutt_anesthetic_2013,purdon_electroencephalogram_2013}.”
